# The Effects of Circumcision on the Penis Microbiome

**DOI:** 10.1371/journal.pone.0008422

**Published:** 2010-01-06

**Authors:** Lance B. Price, Cindy M. Liu, Kristine E. Johnson, Maliha Aziz, Matthew K. Lau, Jolene Bowers, Jacques Ravel, Paul S. Keim, David Serwadda, Maria J. Wawer, Ronald H. Gray

**Affiliations:** 1 Translational Genomics Research Institute (TGen), Flagstaff, Arizona, United States of America; 2 Northern Arizona University, Flagstaff, Arizona, United States of America; 3 Johns Hopkins Medical Institutions, Baltimore, Maryland, United States of America; 4 University of Maryland School of Medicine, Baltimore, Maryland, United States of America; 5 School of Public Health, Makerere University, Kampala, Uganda; 6 Bloomberg School of Public Health, Johns Hopkins University, Baltimore, Maryland, United States of America; Charité-Universitätsmedizin Berlin, Germany

## Abstract

**Background:**

Circumcision is associated with significant reductions in HIV, HSV-2 and HPV infections among men and significant reductions in bacterial vaginosis among their female partners.

**Methodology/Principal Findings:**

We assessed the penile (coronal sulci) microbiota in 12 HIV-negative Ugandan men before and after circumcision. Microbiota were characterized using sequence-tagged 16S rRNA gene pyrosequencing targeting the V3–V4 hypervariable regions. Taxonomic classification was performed using the RDP Naïve Bayesian Classifier. Among the 42 unique bacterial families identified, Pseudomonadaceae and Oxalobactericeae were the most abundant irrespective of circumcision status. Circumcision was associated with a significant change in the overall microbiota (PerMANOVA *p* = 0.007) and with a significant decrease in putative anaerobic bacterial families (Wilcoxon Signed-Rank test *p* = 0.014). Specifically, two families—Clostridiales Family XI (*p* = 0.006) and Prevotellaceae (*p* = 0.006)—were uniquely abundant before circumcision. Within these families we identified a number of anaerobic genera previously associated with bacterial vaginosis including: *Anaerococcus spp.*, *Finegoldia spp.*, *Peptoniphilus spp.*, and *Prevotella spp.*

**Conclusions/Significance:**

The anoxic microenvironment of the subpreputial space may support pro-inflammatory anaerobes that can activate Langerhans cells to present HIV to CD4 cells in draining lymph nodes. Thus, the reduction in putative anaerobic bacteria after circumcision may play a role in protection from HIV and other sexually transmitted diseases.

## Introduction

Randomized trials have shown that male circumcision decreased risk of HIV, HSV-2, and HPV infections in men [1]–[4]. One these trials also demonstrated decreased risk of trichomoniasis and bacterial vaginosis (BV) in the female sexual partners of circumcised men [5] as well as decreased symptomatic genital ulceration in both men and their female sexual partners [4], [6]. On the basis of the HIV findings, WHO/UNAIDS have recommended that circumcision be provided as part of a strategy for HIV prevention in men [7]. However, large-scale population-based male circumcision programs may not always be feasible due to cultural, logistical, and financial barriers. Thus, it is important to better understand the biological mechanisms by which male circumcision reduces the risk of HIV infection as this may lead to the development of novel, non-surgical prevention strategies.

Genital microbial communities may play an important role in modulating HIV risk [8]. Genital mucosal inflammation induced by microbes leads to the activation of HIV target cells and an increase in HIV susceptibility [9]. The dominant HIV target cells in the genital mucosa are two dendritic cell types, langerin^+^ Langerhans' cells and DC-SIGN^+^ dendritic cells [10], [11]. While immature DC-SIGN^+^ dendritic cells are capable of mediating HIV-1 *trans*-infection of the CD4^+^ T-cells [12], Langerhans' cells could play a dual role depending on their activation state. Immature Langerhans' cells protect against HIV-1 infection by internalizing and degrading HIV-1 viral particles and inhibiting T-cell infection [13], but Langerhans' cells activated through exposure to microbial-associated inflammatory molecules such as lipopolysaccharide (LPS), TNF-α, and Toll-like receptor 2 (TLR2) agonists are efficient mediators of HIV-1 *trans*-infection [14]–[16]. Experiments using tissue explants have shown that gram-positive bacteria such as *Staphylococcus aureus*, Group B *Streptococcus* and genital pathogens such as *Candida albicans* and *Neisseria gonorrhea* increase Langerhans' cells susceptibility to HIV-1 infection via TLR2 agonists and TNF-α production [17], [18]. These findings further support the link between genital microbial communities and HIV susceptibility and the importance of genital mucosal inflammation in HIV sexual transmission.

The biological mechanism underlying circumcision-conferred protection against HIV is likely to be multifactorial. Post-circumcision anatomical, immunological, and microbiological changes have all been hypothesized to contribute to the reduction in HIV risk. From the anatomical and immunological perspective, the inner surface of the foreskin is lightly keratinized and contains abundant Langerhans cells close to the mucosal surface resulting in a large number of exposed HIV target cells in the erect uncircumcised penis [19], [20]. From a microbiological perspective, the intact foreskin may support the survival of genital microbes associated with increased foreskin mucosal inflammation and Langerhans' cell activation. Of note, the protection against sexually transmitted infections and BV conferred to the female partners of circumcised men [1], [3]–[6] strongly suggests circumcision-associated microbiological changes in the male genital mucosa.

To assess the impact of circumcision on the penis microbiota (i.e., the microbial community), we characterized samples from the coronal sulcus—the junction between the shaft and glans of the penis—before and after circumcision and evaluated the circumcision-associated changes using culture independent methods and ecological analyses. To our knowledge, no comprehensive, culture-independent evaluations of the male genital mucosa have been published. In contrast, culture-independent molecular analyses methods have been used to characterize the vaginal microbiota, revealing distinct bacterial core community types [21] and previously uncharacterized bacteria associated with BV—many of which are anaerobic [22]. We hypothesized that removing the foreskin would decrease the proportional abundance of anaerobic bacteria in the coronal sulci due to the elimination of anoxic subpreputial microenvironments. This hypothesis was assessed by analyzing paired pre- and post-circumcision swab samples collected from HIV-negative participants during a randomized trial of male circumcision for HIV prevention in Rakai, Uganda [2].

## Results

### 16S rRNA Gene-Based Pyrosequencing Analysis of Penis Coronal Sulci Microbiota Revealed Diverse Array of Bacterial Families

Pyrosequencing analysis of V3–V4 segment of the 16S rRNA genes [23] generated a total of 75,005 sequences from 24 coronal sulci swab samples. To facilitate abundance-based bacterial community comparison, we equilibrated sequence coverage by generating sequence subsets (n = 387 sequences per sample) using random sampling without replacement and performed taxonomic classification of sequence subsets using the Ribosomal Database Project (RDP) Naïve Bayesian Classifier [24]. While nearly all (97.4%) of the sequences analyzed were identified to the phylum level at ≥95% confidence, the proportion of sequences successfully assigned to lower taxonomic levels decreased to 96.44% at the class level, 93.3% at the order level, 90.1% at the family level and 65.9% at the genus level. Of note, only 65.6% of the Proteobacteria—a phylum including many medically important bacterial pathogens—could be classified to the genus-level, which led us to perform subsequent analyses on the family-level (unless otherwise specified).

We found a total of 42 unique bacterial families in the coronal sulci microbiota, with 38 bacterial families among pre-circumcision samples versus 36 detected among post-circumcision samples (Table 1). Pseudomonadaceae was the most abundant family irrespective of circumcision status, constituting over 50% of the coronal sulci microbiota, followed by Clostridiales Family XI, Oxalobacteraceae, and Prevotellaceae for pre-circumcision and Corynebacteriaceae, Oxalobacteraceae, and Staphylococcaceae for post-circumcision (Table 1). As the inter-sample variation for the bacterial abundances was high and the microbiota data lacked normality, subsequent analyses employed ecological methods including non-metric multi-dimensional scaling (nMDS) and permutational multivariate analysis of variance (PerMANOVA) to assess the significance of circumcision on the full community dataset.

**Table 1:** Pre- and post-circumcision abundances of bacterial phylotypes detected in the coronal sulci swabs (n = 387 sequences analyzed per swab sample).

Bacterial family	Pre-circumcision	Post-circumcision	Oxygen Requirement
	Mean (SD)	Mean (SD)	
Pseudomonadaceae	178.83 (87.23)	201.83 (47.86)	A
Oxalobacteraceae	31.42 (18.09)	37.92 (12.96)	A
Corynebacteriaceae	5.25 (7.35)	39.83 (40.81)	F
Clostridiales Family XI	33.58 (37.09)	3.58 (5.32)	AN
Staphylococcaceae	6.08 (16.70)	28.42 (42.80)	F
Prevotellaceae	21.50 (32.96)	0.17 (0.39)	AN
Moraxellaceae	8.08 (5.07)	9.25 (3.31)	A
Comamonadaceae	6.75 (9.38)	7.58 (4.60)	A
Bifidobacteriaceae	11.92 (37.58)	0.17 (0.39)	F/AN
Xanthomonadaceae	4.67 (3.28)	5.83 (3.51)	A
Enterobacteriaceae	4.08 (2.84)	4.83 (1.75)	F
Fusobacteriaceae	7.92 (20.33)	0.17 (0.58)	AN
Aeromonadaceae	3.25 (1.76)	2.42 (1.68)	F
Veillonellaceae	3.67 (6.75)	0.75 (1.22)	AN
Sphingomonadaceae	2.50 (5.04)	1.00 (0.85)	A
Aerococcaceae	1.08 (1.98)	1.42 (2.97)	F
Peptostreptococcaceae	2.42 (5.00)	0.08 (0.29)	AN
Carnobacteriaceae	1.42 (3.37)	0.25 (0.62)	F
Streptococcaceae	1.25 (1.66)	0.42 (0.90)	F
Micrococcaceae	0.75 (0.97)	0.83 (1.19)	A
Flavobacteriaceae	0.50 (0.90)	1.08 (1.31)	A
Burkholderiales Family V	0.58 (1.44)	0.67 (1.15)	A
Porphyromonadaceae	1.17 (1.95)	0.00 (0)	AN
Bacillaceae	0.00 (0)	1.08 (2.84)	A
Caulobacteraceae	0.67 (2.02)	0.42 (0.51)	A
Enterococcaceae	0.08 (0.29)	0.83 (1.19)	F
Lachnospiraceae	0.92 (2.31)	0.00 (0)	AN
Burkholderiaceae	0.50 (0.90)	0.33 (0.89)	A
Campylobacteraceae	0.83 (2.04)	0.00 (0)	M
Coriobacteriaceae	0.58 (1.16)	0.00 (0)	AN
Rhodocyclaceae	0.25 (0.87)	0.33 (0.89)	A
Actinomycetaceae	0.25 (0.45)	0.25 (0.87)	F/AN
Intrasporangiaceae	0.08 (0.29)	0.42 (1.44)	A
Planctomycetaceae	0.42 (0.79)	0.08 (0.29)	A
Halomonadaceae	0.33 (0.65)	0.17 (0.39)	A
Brevibacteriaceae	0.08 (0.29)	0.25 (0.62)	A
Neisseriaceae	0.00 (0)	0.33 (0.49)	F
Bradyrhizobiaceae	0.17 (0.39)	0.08 (0.29)	F/A
Mycoplasmataceae	0.25 (0.62)	0.00 (0)	F
Dermabacteraceae	0.00 (0)	0.17 (0.58)	F
Rhodobacteraceae	0.00 (0)	0.17 (0.58)	A
Pseudomonadales Family VI	0.17 (0.58)	0.00 (0)	U
	Pre-circumcision	Post-circumcision	
Bacterial family by oxygen req.	Total No. (%)	Total No. (%)	
Aerobic	16 (41.1)	18 (50.0)	A
Anaerobic	8 (21.1)	5 (13.9)	AN
Facultative anaerobic	9 (23.7)	10 (27.8)	F
Microaerophilic	1 (2.6)	0 (0)	M
Mixed	3 (7.9)	3 (7.9)	F/A or F/AN
Unclassified	1 (2.6)	0 (0)	U
Total	38	36	

The oxygen requirement of the each detected bacterial phylotype is also presented: *A* = *aerobic*, *AN* = *anaerobic*, *F* = *facultative anaerobic*, *M* = *microaerophilic*. *U* = *unidentified*.

### Male Circumcision Is Associated with a Decrease in Putative Anaerobic Bacteria in the Penis Microbiota

Bacterial families consisting of predominantly anaerobic genera decreased substantially after circumcision, whereas predominantly facultative anaerobic genera increased. Aerobic and microaerophilic bacterial families exhibited less obvious trends. Of the 38 bacterial families detected in pre-circumcision swabs, 16 (41.1%) were aerobic, 8 (21.1%) were anaerobic, 9 (23.7%) were facultative anaerobic, and one (2.6%) was microaerophilic, and 4 (10.5%) were mixed or unclassified. In comparison, in post-circumcision swabs, 18 (50.0%) were aerobic, 5 (13.9%) were anaerobic, 10 (27.8%) were facultative anaerobic, and 3 (8.3%) were mixed (Table 1). The decrease in putative anaerobic bacterial abundance after circumcision was statistically significant (Wilcoxon Signed-Rank test *p* = 0.014), which was accompanied by a statistically significant increase in facultative anearobes (*p* = 0.006) (Table 2). Two anaerobic bacterial families, Clostridiales Family XI (*p* = 0.006) and Prevotellaceae (*p* = 0.006) were uniquely abundant in pre-circumcision coronal sulci samples, whereas an aerobic/facultative anaerobic family—Corynebacteriaceae (*p* = 0.003)—and a facultative anaerobic family—Staphylococcaceae (*p* = 0.04)—were uniquely abundant in post-circumcision coronal sulci samples (Table S4).

**Table 2:** Pre- and post-circumcision abundances of bacterial phylotypes detected in the coronal sulci swabs summarized by oxygen requirement.

Bacterial family by oxygen req.	Pre-circumcision	Post-circumcision	Wilcoxon Signed-Rank Test
	Mean (SD)	Mean (SD)	
Aerobic	236.4 (119.1)	466.8 (65.6)	*p* = 0.62
Anaerobic	71.8 (69.6)	4.8 (6.2)	*p* = 0.014
Facultative anaerobic	22.8 (19.8)	78.9 (53.4)	*p* = 0.006

### Classification on the Genus-Level

We examined the genus-level data of the most abundant bacterial families, as well as specific anaerobic families (Table 3; Table S1). At the ≥95% bootstrap confidence level, we identified 77.25% of the Pseudomonadaceae sequences as *Pseudomonas spp.*, 29.7% of the Oxalobacteraceae sequences as *Janthinobacterium spp.*, 86.8% of the Clostridiales Family XI sequences as *Anaerococcus spp.*, *Finegoldia spp.*, *Peptoniphilus spp.*, *Helcococcus spp.*, *or Parvimonas*, 58.5% of the Prevotellaceae as *Prevotella spp*, 99.5% of the Staphylococcaceae sequences as *Staphylococcus spp.*, and 100.0% of the Corynebacteriaceae sequences as *Corynebacterium spp.* When we assessed two bacterial families previously identified in the vaginal microbiota—Bifidobacteraciaceae and Fusobacteriaceae—we identified 98.6% of the Bifidobacteraciaceae sequences to be *Gardnerella spp.*, with the remainder being *Bifidobacterium spp.* In Fusobacteriaceae, we identified 77.3% of the sequences to be *Sneathia spp.* and 14.4% to be *Fusobacterium spp.* Additional anaerobic genera detected in the penis microbiota included *Dialister spp.*, *Veillonella spp.*, *Peptostreptococcus spp.*, *and Porphyromonas spp.*

**Table 3:** Genus-level taxonomic classifications of the most abundant bacterial families, including select anaerobic bacterial families.

	Pre-Circumcision	Post-Circumcision
	# of sequences detected (%)	# of sequences detected (%)
Pseudomonadaceae	2146 (100.00)	2422 (100.00)
*Pseudomonas spp.*	1667 (77.68)	1862 (76.88)
*Unclassified*	479 (21.32)	560 (23.12)
Oxalobacteraceae	377 (100.00)	455 (100.00)
*Janthinobacterium spp.*	121 (32.10)	126 (27.69)
*Unclassified*	256 (67.90)	329 (71.31)
Clostridiales Family XI	403 (100.00)	43 (100.00)
*Anaerococcus spp.*	104 (25.80)	25 (58.14)
*Finegoldia spp.*	118 (29.28)	16 (37.21)
*Peptoniphilus spp.*	113 (28.04)	2 (4.65)
*Helcococcus spp.*	1 (0.25)	0 (0)
*Parvimonas spp.*	8 (1.99)	0 (0)
*Unclassified*	59 (14.64)	0 (0)
Prevotellaceae	258 (100.00)	2 (100.00)
*Prevotella spp.*	150 (58.14)	2 (100)
*Unclassified*	108 (41.86)	0 (0)
Staphylococcaceae	73 (100.00)	341 (100.00)
*Macrococcus spp.*	0 (0)	1 (0.29)
*Staphylococcus spp.*	73 (100)	339 (99.41)
*Unclassified*	0 (0)	1 (0.29)
Corynebacteriaceae	63 (100.00)	478 (100.00)
*Corynebacterium spp.*	63 (100)	478 (100)
*Unclassified*	0 (0)	0 (0)
Moraxellaceae	97 (100.00)	111 (100.00)
*Acinetobacter spp.*	86 (88.66)	107 (96.40)
*Unclassified*	11 (11.34)	4 (3.60)
Comamonadaceae	81 (100.00)	91 (100.00)
*Acidovorax spp.*	25 (30.86)	13 (14.29)
*Polaromonas spp.*	3 (3.70)	2 (2.20)
*Delftia spp.*	3 (3.70)	1 (1.10)
*Unclassified*	50 (61.74)	75 (82.40)
Bifidobacteriaceae	143 (100.00)	2 (100.00)
*Gardnerella spp.*	141 (98.60)	2 (100.00)
*Bifidobacterium spp.*	2 (1.40)	0 (0)
Xanthomonadaceae	56 (100.00)	70 (100.00)
*Stenotrophomonas spp.*	18 (32.14)	18 (25.71)
*Unclassified*	38 (67.86)	52 (74.29)
Fusobacteriaceae	95 (100.00)	2 (100.00)
*Sneathia spp.*	73 (76.84)	2 (100.00)
*Fusobacterium spp.*	14 (14.74)	0 (0)
*Unclassified*	8 (8.42)	0 (0)
Veillonellaceae	44 (100.00)	9 (100.00)
*Dialister spp.*	36 (81.82)	5 (55.56)
*Veillonella spp.*	1 (2.27)	3 (33.33)
*Unclassified*	7 (15.91)	1 (11.11)
Peptostreptococcaceae	29 (100.00)	1 (100.00)
*Peptostreptococcus spp.*	29 (100.00)	1 (100.00)
Porphyromonadaceae	14 (100.00)	0 (0)
*Porphyromonas spp.*	13 (92.86)	0 (0)
*Unclassified*	1 (7.14)	0 (0)

### Penis Microbiota Heterogeneity after Circumcision

**Figure 1:**
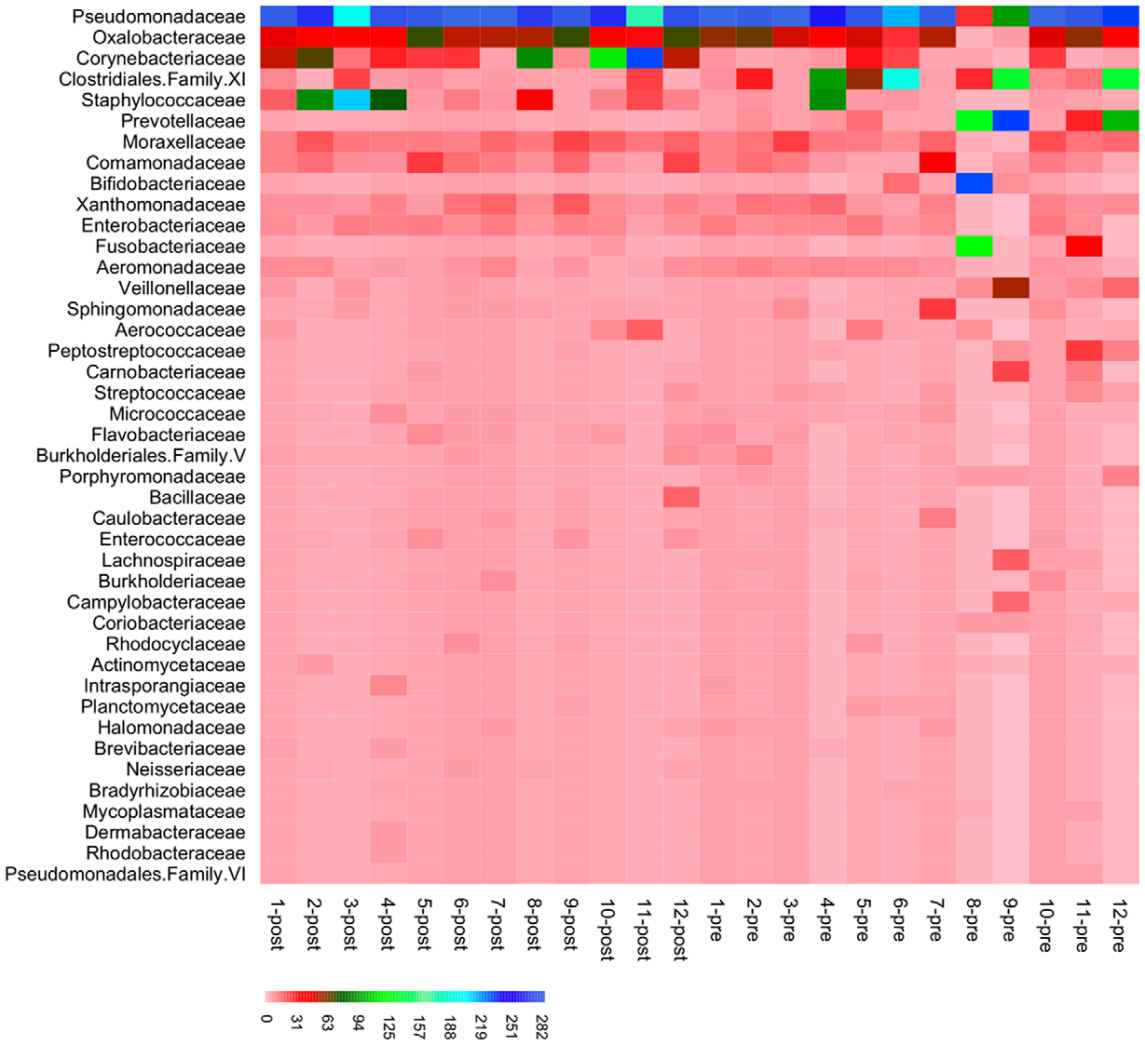
Heatmap display of the bacterial families in the coronal sulci microbiota pre- and post-circumcision. The microbiota from 12 paired coronal sulci samples is presented with the abundance of each phylotype represented by a colored block as specified in the legend.

We assessed shifts in the overall penis microbiota after circumcision using community analysis methods. Heatmap display of bacterial family abundance showed that the coronal sulci microbiota were more homogeneous after circumcision (Figure 1). Before circumcision, a greater number of dominant bacterial families were observed, with Pseudomonadaceae, Clostridiales Family XI, and Prevotellaceae being most commonly dominant and Fusobacteriaceae, Bifidobacteraciaceae, or Staphylococcaceae dominating select samples. In contrast, Pseudomonadaceae, Corynebactericeae, and Staphylococcaceae were the only dominant bacterial families observed after circumcision.

**Figure 2:**
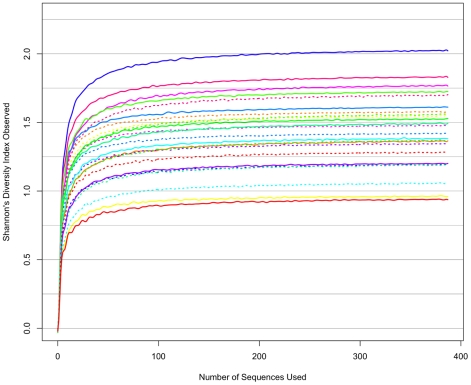
Shannon-Weaver diversity indices generated using the 16S rRNA gene pyrosequencing data. The diversity index is based on the number of unique phylotypes and their frequency, with the rare phylotypes having lower effect on the diversity index. These analyses indicate that diversity was sufficiently described after analyzing 387 sequences from each sample. Pre-circumcision samples appeared to have a greater range of community diversity as compared to post-circumcision. *Solid lines* = *pre-circumcision*; *dotted line* = *post-circumcision*.

This trend in decreased microbiota heterogeneity associated with circumcision was supported by the richness and diversity analyses. A higher number of unique bacterial families were found prior to circumcision (Table 4, 2). The coronal sulci microbiota had a greater range of diversity values before circumcision, which appeared to decrease following circumcision (Figure 2), but the difference was not statistically significant (Wilcoxon Signed-Rank test *p* = 0.47) (Table 4, Table S2). Based upon the diversity analysis, we also concluded that while 387 sequences may have been insufficient to characterize all phylotypes present, they were sufficient to estimate the overall microbiota diversity in all samples.

**Table 4:** Richness and Shannon Diversity Index at ≥95% bootstrap confidence level.

Pre-circ.	Post-circ.	Total	Pre-circ.	Post-circ.	Pre-/Post-circ.
No. unique bacterial families mean (SD)	No. of unique bacterial families mean (SD)	No. unique bacterial families	Shannon index mean (SD)	Shannon index mean (SD)	Shannon index mean ratio
16.5 (3.55)	16 (3.25)	42	1.51 (0.34)	1.41 (0.19)	1.07 (*p* = 0.47)

### Circumcision Is Associated with Significant Shift in the Penis Microbiota

**Figure 3:**
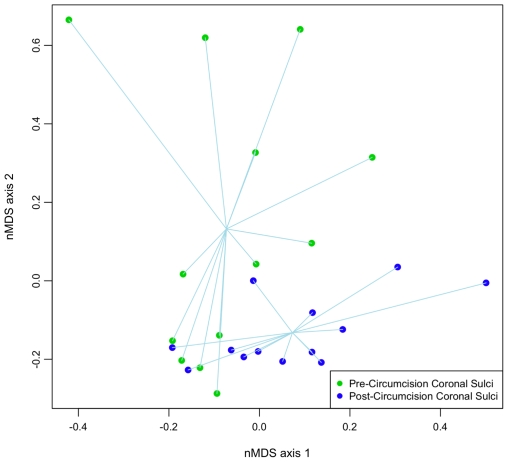
Comparison of pre- and post-circumcision coronal sulci microbiota using nMDS ordination. nMDS is an ordination method that ranks the distances calculated using community data, with each data point representing the community data from a single sample; it is used to reduce data complexity and to extract meaningful relationships amongst communities. Lines connect individual communities to the centroid values for each group.

Using nMDS, the overall composition of the penis microbiota formed two distinct clusters pre- and post-circumcision (Figure 3, Figure S1). While the pre-circumcision samples exhibited more variance along the y-axis, the post-circumcision samples varied mostly along the x-axis, indicating that the pre- and post-circumcision microbiota were composed of different bacterial families. Furthermore, post-circumcision samples formed a smaller cluster than pre-circumcision samples, which supported our earlier observation of the decrease in microbiota heterogeneity after circumcision. When we compared the pre- and post-circumcision microbiota using PerMANOVA, we found that the coronal sulci microbiota was significantly modified after circumcision (*p* = 0.007) (Table S3).

### Potential Positive Interactions between Pseudomonadaceae and Oxalobacteraceae

We assessed the correlations between bacterial families using Kendall's τ, and found a greater number of correlations (potential biological interactions) among pre-circumcision samples than post-circumcision samples (Figure S3). We also observed that the Pseudomonadaceae and Oxalobacteraceae—two of the most abundant phylotypes— were positively correlated irrespective of circumcision state, possibly suggesting a cooperative interaction. In contrast, the four indicator phylotypes—Clostridiales family XI, Prevotellaceae, Corynebacteriaceae, and Staphylococcaceae—were all negatively correlated with Pseudomonadaceae and Oxalobacteraceae, suggesting that these two abundant phylotypes may be the main competitors against the indicator phylotypes.

## Discussion

We found that pre-circumcision microbiota appeared more heterogeneous than post-circumcision microbiota, with several core community types observed. One community type appeared to be dominated by members of the Clostridiales Family XI and Prevotellaceae families (Figure 1). These two families have been identified in the normal human vagina and when present in higher numbers have been associated with BV [22], [25], a condition characterized by a shift in the composition of vaginal microbial communities that results in decreased numbers of lactic acid producing bacteria, increased numbers of strict anaerobes, and elevated vaginal pH. The coronal sulci microbiota observed in pre-circumcision samples in this study were similar to several core community types observed in the vagina. Whether the presence of these specific phylotypes in the male genital microbiota is associated with a diseased versus normal state or with HIV risk is unknown, but should be investigated in future studies. Likewise, it is unclear whether these phylotypes are acquired from the vagina or vice-versa. We previously reported that male circumcision was associated with reduced BV in female sexual partners [5] and we hypothesize that decreased anaerobic bacteria in the order of Clostridiales and the family of Prevotellaceae may be involved in the causal pathway between male circumcision and reduction of BV in sexual partners.

*Pseudomonas* species are ubiquitous and include many non-pathogenic species isolated from soil, water, and other environmental sources [26], [27]. *Pseudomonas aeruginosa* is an important waterborne clinical pathogen associated with bacterial infections [28], [29] and a common nosocomial pathogen in intensive care units [30]–[36]. *Pseudomonas spp.*-dominated penis microbiota was common in this study and may represent a core penis microbiota type that is determined by a combination of environmental exposure, host genetics and mucosal immunity [37]–[40]. Future studies are needed to characterize the potential range of penis microbiota core types and their relationship with disease risk.

We controlled for the immunological changes associated with HIV infection by only including HIV negative participants in this study. HIV infection has been associated with significant changes in host mucosal immunity, including decreased IgA response [41], gut-associated lymphoid tissue CD4(+) T-cells [42], and mucosa-associated lymphoid tissue-based memory CD4(+)CCR5(+) T-cells [43], [44]. Therefore, we hypothesize that the HIV-associated changes in mucosal immunity can lead to a different genital microbiota profile in HIV-positive men. Additional studies will be needed to investigate the impact of HIV status on the male genital microbiota [45].

This study is the first molecular assessment of the bacterial diversity in the male genital mucosa. The observed decrease in anaerobic bacteria after circumcision may be related to the elimination of anoxic microenvironments under the foreskin. Detection of these anaerobic genera in other human infectious [46] and inflammatory pathologies [47]–[50] suggests that they may mediate genital mucosal inflammation or co-infections in the uncircumcised state. Hence, the decrease in these anaerobic bacteria after circumcision may complement the loss of the foreskin inner mucosa to reduce the number of activated Langerhans cells near the genital mucosal surface and possibly the risk of HIV acquisition in circumcised men.

## Materials and Methods

### Ethics Statement

Participants were provided written informed consent for screening, enrollment and follow up, and men receiving circumcision were also provided written consent for surgery. All participants were offered intensive HIV prevention education, access to free HIV voluntary counseling and testing (VCT), and condoms, provided free of charge, and were strongly encouraged at each study visit to practice safe sex behaviors and to avail themselves of VCT and condoms. The trials are registered with ClinicalTrials.gov numbers NCT00425984 for NIH trial and NCT00124878 for the Gates Foundation trial. The protocol (Protocol S1) was reviewed and approved by the Uganda National Council for Science and Technology, and by three Institutional Review Boards (IRBs): the Science and Ethics Committee of the Uganda Virus Research Institute, the Committee for Human Research at Johns Hopkins University, Bloomberg School of Public Health, and the Western Institutional Review Board, Olympia, Washington. The data were analyzed without personal identifiers.

### Sample Collection

We conducted a randomized trial of male circumcision for HIV prevention, in which uncircumcised men aged 15–49 were randomized to either immediate circumcision (intervention) or to circumcision delayed for 24 months (controls) as described elsewhere (Figure S4) [5]. Study participants were followed at 6, 12 and 24 months to assess HIV and STI acquisition. At each visit, participants were asked permission to take a penile swab from the coronal sulcus. During sample collection, saline moistened Dacron swabs were rolled over the coronal sulcus, placed in PCR buffer and stored at −80°C until assayed. A random subset of 12 HIV-negative participants from the study with coronal sulci swabs at enrollment (prior to circumcision) and at 12 months following circumcision was analyzed in the current study.

### DNA Extraction and Purification

Genomic DNA was extracted from swab samples using a bead-beating and enzymatic lysis protocol, followed by purification using a QIAamp DNA Mini Kit (Qiagen, Valencia, USA). Briefly, the 50 µl inoculate from the coronal sulci swabs were thawed on ice and 0.75 ml of TE50 (10mM Tris-HCl+50mM EDTA, pH 8.0) solution added. 500 µl of the solution was transferred to a clean, sterile bead-beating tube (MP Biomedicals, Solon, USA) and kept on ice. A lytic enzyme cocktail was prepared at the time of extraction and added to each sample as follows: 50 µl Lysozyme (450 kU ml-1), 6 µl Mutanolysin (25 kU ml−1), 3 µl Lysostaphin (4 kU ml−1) and 41 µl TE50 for a final volume of 100 ml per sample. Samples were digested by incubating at 37°C for 60 min in a dry heat block before centrifugation at 1200 rpm for 1 min. To each digested sample, 750 mg of sterile 0.1 mm diameter zirconia silica beads (BioSpec Products Inc., Bartlesville, USA) were added. Bead-beating was performed for 1 min at 2100 rpm using a BioSpec Mini-Bead Beater-96. Following bead disruption, the tubes were centrifuged at 1200 rpm for 1 min. Two 200 ml aliquots of crude lysate from each sample were transferred to new, sterile microcentrifuge tubes. To each tube, 25 µl of Proteinase K (20 mg/ml (>600 mAU/ml)) and 200 µl of Qiagen buffer AL were added. Samples were mixed by pulse-vortexing for 15 sec and then incubated at 56°C for 10 min before being centrifuged at 1200 rpm for 1 min. For each 200 µl crude lysate, 20 µl of 3 M sodium acetate, pH 5.5 was added followed by 200 µl of molecular grade ethanol (96–99.5%). Vortexing was repeated for an additional 15 sec before being centrifuged at 1200 rpm for 1 min. From this point onward, purification was carried out using the QIAmp DNA Purification from Blood or Body Fluids as per manufacturer's instructions. Aliquots from the same sample were loaded onto the same column. Purified genomic DNA was stored at −80°C until analysis.

### Pyrosequencing Analysis of the 16S rRNA Gene V3–V4 Region for Parallel Tagged Sequencing on the 454® Platform

The 16S rRNA gene was amplified in two replicate 50 µl reaction volumes. In each 50 µl reaction, 3 µl was added to 47 µl of PCR reaction mix containing 450 nM of each broad range forward (5′-CCTACGGGAGGCAGCA-GT-3′) and reverse primer (5′-GGACTACCAGGGTATCTAATCCTGTT-3′) [51], 1× PCR buffer without MgCl_2_ (Invitrogen, Carlsbad, USA), 3 mM MgCl_2_, 0.2 mM dNTP mix, 1 U platinum *Taq* (Invitrogen) using the following touch-down PCR condition: 90s at 95°C for initial denaturation, 30s at 95°C for denaturation, 30s at 64°C for annealing, 30s at 72°C for extension with the annealing temperature decreasing by 0.3°C for each subsequent cycle for 34 cycles, followed by 5 min at 72°C for final extension. Subsequent purification, blunt-end repair, adapter ligation, amplicon quantification and pooling, restriction digestion, and pyrosequencing library generation were carried according to a previously published protocol [52]. The sample-specific, palindromic, self-hybridizing barcodes used in the tagging reactions were generated using a self-complementary 8-nt barcode and a rare restriction site according the same protocol. The pooled tagged single-stranded pyrosequencing library underwent fusion PCR and pyrosequencing using a Roche 454 FLX Pyrosequencer (454 Life Sciences, Branford, USA) according to the manufacturer instructions at the Institute for Genome Sciences of University of Maryland.

### Pyrosequencing Data Processing

Experimental sequences were processed using a custom PERL script, which performed the following: the script filtered the sequence files and retained only sequences that were 200-nt or longer. Regular expressions were then applied to the remaining sequences to search for a single barcode sequence in each FASTA sequence, binned each sequence accordingly, and scanned each binned sequence for the 16S forward primer sequence. The script then trimmed off the forward primer sequence and oriented the remaining sequence such that all sequences begin with the 5′ end according to standard sense strand conventions. As a result of our processing, sequences that were shorter than 200-nt or had multiple barcode or primer motifs were excluded from the analysis. We included only sequences with the forward primer motif to ensure that the highly informative V3 region was available for taxonomic assignment.

### Subset Generation and Taxonomic Assignment

We evaluated the sequencing coverage by examining the number of 16S rRNA gene sequences generated per swab sample, which showed that the lowest number of sequences per sample to be n = 387. To facilitate subsequent phylotype abundance-based analyses, we standardized/normalized the number of sequences per swab sample to n = 387 sequences by sampling randomly without replacement prior to taxonomic classification to generate a single subset. We repeated this process four more times to generate a total of five subsets for confirmatory data analyses. Samples with less than n = 387 sequences were excluded. We classified the 16S rRNA gene sequences in each subset at each taxonomic level (*i.e.*, phylum, class, order, family, genus) at three bootstrap confidence levels: ≥95%, ≥97%, ≥99% using a web service for the Naïve Bayesian Classifier made available by the Ribosomal Database Project [24]. An SQL database was used to store and query the results. To control for potential errors due to pyrosequencing that may have created rare phylotypes, we removed all family-level phylotypes occurring only once (i.e., singletons) in the dataset, the estimated equivalent of a proportional abundance of 1/387 = 0.26% in a single sample or 1/(387*24) = 0.011% in the full dataset. The phylotype abundance data was converted into a data matrix in R, which we converted to a proportional abundance data matrix calculated by dividing the phylotype abundance by the total number of sequences assigned to the family-level in each sample. We assessed the potential biases of the subset generation approach by analyzing all five subsets classified at ≥95%, ≥97%, and ≥99% bootstrap confidence levels and found the variation among the subsets and different classification cutoffs to be non-significant (Text S1). Subsequently, all data presented in the manuscript were derived from Subset 1 classified at 95% confidence level.

### Putative Oxygen Requirement Classifications

Oxygen requirement (i.e., aerotolerance) classification was generated based on the genus- and family-level taxonomy through extensive literature search. Briefly, we compiled the bacterial genera included in each detected bacterial family using the NCBI taxonomy browser [53], [54]. We used a two-tiered approach to obtain the aerotolerance information for each identified bacterial genera, beginning with the Gideon Online Microbiology Feature from the Global Infectious Disease & Epidemiology Network [55], which contains phenotypic characteristics including oxygen requirements for medically important bacterial genera. For genera unable to be assigned using this approach, we performed literature search on the PubMed database for relevant primary literature. Finally, we assigned oxygen requirement to each bacterial family using the genera-level classifications. In most cases, bacterial families consist of genera with the same aerotolerance profile and an overall oxygen requirement could be assigned. In bacterial families with mixed aerotolerance profiles, we examined the genus-level sequence classification data. For bacterial families with incomplete genus-level classification or mixed genera profiles, a mixed designation was given to the bacterial family. One bacterial family was unclassified using our tiered approach.

### Phylotype and Oxygen Requirement Group Abundance-Based Analyses

Using the phylotype abundance data matrix, we calculated the mean and standard deviation of the bacterial families and oxygen tolerance groups from pre-circumcision (n = 12) and post-circumcision (n = 12) samples in Microsoft Excel (Microsoft Corp., Seattle, USA). Significance test for the change in oxygen requirement groups after circumcision was performed using a two-tailed Wilcoxon Signed-Rank Test with α = 0.05 and was performed in R.

### Visualization of the Penis Microbiota Data

Subsequent data visualization and analyses were performed in R version 2.9.1 unless otherwise specified [56]. We visualized the penis microbioa using two different approaches: 1) a heatmap display and 2) non-metric multi-dimensional scaling (nMDS). Heatmap display. We visually assessed the coronal sulci microbiota using a heatmap display in the vegan package (Figure 1, Figure S2). Non-metric multidimensional scaling (nMDS) analysis. We utilized nMDS ordination to visually assess patterns microbiota compositional differences among our samples. All nMDS plots were generated in R using functions from ecodist [57], ellipse [58], and BiodiversityR [59]. The Bray-Curtis distance was used for its excellent properties when used with community data [60], [61]. To generate each nMDS plot, we began by evaluating the number of dimensions required to appropriately present the bacterial communities using a stress plot, which was generated using n = 10 iterations of nMDS for dimensions n = 1 through n = 5. We applied a conventional cutoff of <0.2 to determine the acceptable number of dimensions. Using the appropriate number of dimensions n = 50 iterations, Using the appropriate number of dimensions, the nMDS procedure was repeated with n = 50 iterations to more fully explore the ordination space at that dimensionality. The minimum stress solution from this was used to produce the nMDS plots in which each data point represents the bacterial community found in one sample. The spatial distance between points in the plot can be interpreted as the relative difference in community composition, hence, points that are closer are more similar than points that are more distant.

### Rarefaction and Shannon Diversity Analyses

Rarefied richness values were generated using the “rarefy” function in the *vegan* package [62]. Equivalent values of Shannon's diversity index across the same range of sample sizes were estimated using the “H.sampler” function in the DiversitySampler package [63]. Richness described as the number of unique phylotypes and the mean and standard deviation values presented in Table 4 were calculated in Microsoft Excel (Microsoft Corp., Seattle, USA). The significance test for change in microbiota diversity was performed using a two-tailed Wilcoxon Signed-Rank Test with α = 0.05 and was performed in R.

### Multivariate Ecological Analyses

Due to the high level of similarity between the microbiota data matrix in the current study to ecological community data matrices, we chose to apply multivariate analysis methods typically used for ecological community data to assess the coronal sulci microbiota. Ecology-type community data, which can be non-normal, zero-rich, and highly skewed—such as our current dataset—often require special analytical methods; this has been investigated and discussed thoroughly in the ecology literature. Permutation Multivariate Analysis of Variance (PerMANOVA). We used PerMANOVA to test the null-hypothesis of no-difference between the bacterial communities found in pre-circumcision versus post-circumcision coronal sulci swabs (α = 0.05). PerMANOVA is a permutation-based version of the multivariate analysis of variance [64]. It uses the distances between samples to partition variance and randomizations or permutations of the data to produce the p-value for the hypothesis test. It is non-parametric (or semi-parametric for multi-factor models) and, therefore, robust to the assumption of multivariate normality making it less prone to Type I errors. All PerMANOVA analyses were performed in R using the “adonis” function from the *vegan* package [62]. Indicator Species Analysis. While the multivariate analysis above allowed us to test for differences in microbiota composition before and after circumcision, it could not determine the phylotypes that contributed to those differences and test our overall hypothesis that anaerobic bacterial colonization decreased after circumcision. To identify the bacterial families that were uniquely present in each circumcision state, we preformed an Indicator Species Analysis, which is commonly used with species data but can be applied equally to family-level data [65]. All indicator analyses were performed in R using the “duleg” function from the labdsv package [66]. Significance level for the indicator analysis was set at 0.05 with an indicator value greater than 0.50.

### Phylotype Correlation Analysis

To assess the potential net competitive and cooperative interactions among the ten most abundant phylotypes, we utilized pair-wise correlation analysis (Figure S3). We evaluated potential interactions between the ten most abundant bacterial families found in pre- and post-circumcision samples using the Kendall's τ—a non-parametric correlation coefficient—for which a positive value (τ>0) indicates positive correlation and vice versa. We tested for significant correlation using a two-tailed Kendall's τ-test (i.e., the significance of the difference of Kendall's τ from τ = 0) at *p* = 0.05 [67]. We performed the analysis separately for pre-circumcision and post-circumcision because of their significantly different microbiota. Kendall's τ was selected in consideration for the likely non-linear relationships between the detected bacterial families. Given the exploratory nature of the analyses, we tested using a 0.05 significance level for each correlation. All correlation analyses were performed in R.

## Supporting Information

Text S1 Inter-subset and inter-classification level variation assessment.
(0.20 MB DOC)

Figure S1 Confirmation of nMDS ordination using multiple subsets. nMDS plots generated using additional subsets at an OTU definition of > = 95%, > = 97%, and > = 99% bootstrap confidence levels, which demonstrated high level of consistency between subsets and among the different levels of OUT definitions. The two-factor PerMANOVA test using the > = 95% bootstrap confidence level dataset (A) comparing the difference between subsets found no difference between subsets (p = 1.00), whereas a significant difference was found between pre-circumcision and post-circumcision coronal sulci microbiota (p = 0.001). Additional PerMANOVA tests using (B) > = 97% and (C) > = 99% bootstrap confidence level datasets showed similar results. (solid dot = pre-circumcision samples; circles = post-circumcision samples).
(9.74 MB TIF)

Figure S2 Heatmap plots generated using all five data subsets. Heatmap plots generated using phylotype abundance data from additional subsets at > = 95%, > = 97%, and > = 99% bootstrap confidence levels. The same trend in phylotype abundances is observed among the subsets and three OTU definitions: (A) > = 95%, (B) > = 97%, and (C) > = 99% bootstrap confidence levels.
(8.93 MB TIF)

Figure S3 Phylotype correlation plots of the ten most abundant phylotypes found in the pre-circumcision and the post-circumcision coronal sulci microbiota. Phylotype correlation plots of the ten most abundant phylotypes found in the (A) pre-circumcision and the (B) post-circumcision coronal sulci microbiota. In these correlation plots, each phylotype is compared to all nine remaining phylotypes graphically (lower half of the correlation plot) and statistically (upper half of the correlation plot). In the graphical comparison, two phylotypes are plotted against each other in each sub-plot, with one phylotype's abundance on the x-axis and another phylotype's abundance on the y-axis. In the statistical comparison, the Kendall's τ is calculated to evaluate the correlation, with the statistically significant τ values highlighted in red. In contrast to the pre-circumcision correlation plot (A), where 26 potential correlations were observed, only 10 potential correlations were observed among the post-circumcision samples (B). Abbreviations: Pseudo = Pseudomonadaceae, Oxalo = Oxalobacteraceae, Coryn = Corynebacteriaceae, Clost = Clostridiales Family XI, Staph = Staphylococcaceae, Prevo = Prevotellaceae, Morax = Moraxellaceae, Comam = Comamonadaceae, Bifid = Bifidobacteriaceae, Xanth = Xanthomonadaceae, Enter = Enterobacteriaceae, Fusob = Fusobacteriaceae.
(10.29 MB TIF)

Figure S4 CONSORT diagram from the randomized control trial
(12.02 MB TIF)

Table S1 Circumcision-associated % change and standard deviations of the six most abundant phylotypes found in coronal sulci microbiota generated from additional subsets at OTU definitions of > = 95% bootstrap confidence level. While the overall inter-subset variations for the mid-abundance phylotpes were small, the inter-subset variations are larger in phylotypes of relatively higher and lower abundance such as Pseudomonaceae and Oxalobacteraceae.
(0.03 MB DOC)

Table S2 Richness and diversity values from additional subsets at OTU definitions of > = 95%, > = 97%, and > = 99% bootstrap confidence level. We assessed the level of variation in richness and diversity values between randomly generated subsets among three bootstrap confidence levels. We found little variation between subsets and between (A) > = 95%, (B) > = 97%, and (C) > = 99% bootstrap confidence levels.
(0.06 MB DOC)

Table S3 PerMANOVA analysis using additional subsets based on both abundance and proportional abundance data matrices generated using OTU definitions of > = 95%, > = 97%, and > = 99% bootstrap confidence levels. In addition, we also compared our PerMANOVA-generated significance-levels with those from the multi-response permutational procedure (MRPP), another permutational method for testing the null-hypothesis of no-difference between community ecological data. (A) PerMANOVA and MRPP results at > = 95% bootstrap confidence level. (B) PerMANOVA and MRPP results at > = 97% bootstrap confidence level. (C) PerMANOVA and MRPP results at > = 99% bootstrap confidence level.
(0.04 MB DOC)

Table S4 Indicator species analysis using additional subsets based on both abundance and proportional abundance data matrices generated using OTU definitions of (A) > = 95%, (B) > = 97%, and (C) > = 99% bootstrap confidence levels.
(0.11 MB DOC)

Protocol S1 Rakai Randomized Control Trial Protocol
(1.37 MB DOC)
