# Supplementary material for: The Effects of Circumcision on the Penis Microbiome
Source: PLoS One. 2010 Jan 6;5(1):e8422. doi: 10.1371/journal.pone.0008422 (PMC2798966; doi:10.1371/journal.pone.0008422)
Supplement: Text S1 — Inter-subset and inter-classification level variation assessment. (0.20 MB DOC) [file pone.0008422.s001.doc]

**SUPPORTING INFORMATION**

**Inter-subset and inter-classification level variation assessment.**

To confirm that our findings did not occur by chance alone during the subset generation step or due to the selection of bootstrap confidence level, we repeated data analyses using all data subsets classified at three bootstrap confidence levels. We first examined the mean phylotype abundance and standard deviations of the six most abundant phylotypes found in the coronal sulci microbiota (Table S1). Collectively, these six phylotypes account for more than 80% of the sequences generated. We found that while minimal inter-subset variations for Corynebacteriaceae, Prevotellaceae, Staphylococcaceae, and Clostridiales Family XI were observed, variations in Pseudomonadaceae (relatively more abundant) and Oxalobacteraceae (relatively less abundant) were larger than in the other phylotypes.

a. Variations in richness and diversity measurements. To better assess the inter-subset and inter-classification level variations in the bacterial community structure, we turned to richness and diversity analyses. Here, we found that while inter-subset and inter-classification level variations are present, conclusions derived based on a single subset classified at the ≥95% boostrap confidence level were appropriate. Specifically, the same decrease in the standard deviation of the diversity index post-circumcision was observed in all subsets and at all confidence levels (Table S2), thus supporting our inference that circumcision may have led to a more homogeneous type of bacterial community.

b. Variations in community analysis outputs. To visualize the community data in each subset and to test the significance of the sequence sampling in contributing to our findings, we performed additional community ecological analyses using all re-classified data subsets. We began by visualizing the phylotype abundance in all subsets using the heatmap plots, which showed minimal difference between subsets and bootstrap confidence levels (Figure S2). We next visualized the bacterial communities using nMDS plots (Figure S1). Tight clustering of each data point (i.e., an individual sample) with its equivalent from other subsets indicates that the subset generation introduced only insignificant variation to the bacterial community structure. In addition, the overall relatedness of the coronal sulci bacterial communities remained consistent among subsets. These findings held true for all three bootstrap confidence levels. We tested the significance of subset number and of circumcision status concurrently using a two-factor PerMANOVA test. We found that while circumcision status remained a statistically significant environmental variable, inter-subset differences were non-significant (Figure S1). The PerMANOVA test generated identical results for all bootstrap confidence levels. Next, we examined each subset separately. Our MRPP and PerMANOVA analyses showed consistent results irrespective of the bootstrap confidence level or the subset tested (Table S3). Furthermore, we observed no difference between analyses based on abundance versus proportional abundance data. Our indicator analyses showed consistent results irrespective of the bootstrap confidence level or the subset tested (Table S4). Two bacterial families—Xanthomonadaceae (in subset 4 only, but at all three bootstrap confidence levels) and Enterobacteriaceae (in subset 2 at ≥95% only)—met the significance test cut-off but did not appear in all subsets, which suggests that more than one subset should be analyzed to confirm the indicator analysis results.

c. Conclusion. In our additional analyses, we found that simple descriptive analyses such in Table S1 demonstrated more noise from the subset sampling than the community analyses methods. We also found that the normalized phylotype abundance data alone is sufficient for community analyses without having to convert to a proportional abundance data matrix. We further found that the bootstrap confidence level chosen did not have a significant impact on the analysis outcomes in this study (i.e., ≥95% alone was sufficient). Based on these analyses, we concluded that our approach of applying random sampling without replacement to the full dataset to generate equilibrized data subset for abundance-based analysis is sound, with the caveat that three to five subsets should be generated and analyzed to minimize the probability of non-reproducible results occurring due to the sampling process.

**Supporting Figure legends**

**Figure S1.** nMDS plots generated using additional subsets at an OTU definition of ≥95%, ≥97%, and ≥99% bootstrap confidence levels, which demonstrated high level of consistency between subsets and among the different levels of OUT definitions. The two-factor PerMANOVA test using the ≥95% bootstrap confidence level dataset (A) comparing the difference between subsets found no difference between subsets (*p* = 1.00), whereas a significant difference was found between pre-circumcision and post-circumcision coronal sulci microbiota (*p* = 0.001). Additional PerMANOVA tests using (B) ≥97% and (C) ≥99% bootstrap confidence level datasets showed similar results. (solid dot = pre-circumcision samples; circles = post-circumcision samples).

**Figure S2.** Heatmap plots generated using phylotype abundance data from additional subsets at ≥95%, ≥97%, and ≥99% bootstrap confidence levels. The same trend in phylotype abundances is observed among the subsets and three levels of OTU definitions: (A) ≥95%, (B) ≥97%, and (C) ≥99% bootstrap confidence levels.

**Figures S3.** Phylotype correlation plots of the ten most abundant phylotypes found in the (A) pre-circumcision and the (B) post-circumcision coronal sulci microbiota. In these correlation plots, each phylotype is compared to all nine remaining phylotypes graphically (lower half of the correlation plot) and statistically (upper half of the correlation plot). In the graphical comparison, two phylotypes are plotted against each other in each sub-plot, with one phylotype’s abundance on the x-axis and another phylotype’s abundance on the y-axis. In the statistical comparison, the Kendall’s  is calculated to evaluate the correlation, with the statistically significant  values highlighted in red. In contrast to the pre-circumcision correlation plot (A), where 26 potential correlations were observed, only 10 potential correlations were observed among the post-circumcision samples (B). Abbreviations: *Pseudo* = Pseudomonadaceae, *Oxalo* = Oxalobacteraceae, *Coryn* = Corynebacteriaceae, *Clost* = Clostridiales Family XI, *Staph* = Staphylococcaceae, *Prevo* = Prevotellaceae, *Morax* = Moraxellaceae, *Comam* = Comamonadaceae, *Bifid* = Bifidobacteriaceae, *Xanth* = Xanthomonadaceae, *Enter* = Enterobacteriaceae, *Fusob* = Fusobacteriaceae.

**Supporting Tables**

**Table S1.** Circumcision-associated % change and standard deviations of the six most abundant phylotypes found in coronal sulci microbiota generated from additional subsets at OTU definitions of ≥95% bootstrap confidence level.

| **95% Conf level** | **Pseudomonadaceae**  (mean % change; SD) | **Corynebacteriaceae** (mean % change; SD) | **Prevotellaceae**  (mean % change; SD) | **Staphylococcaceae**  (mean % change; SD) | **Clostridiales Family XI** (mean % change; SD) | **Oxalobacteraceae** (mean % change; SD) |
| --- | --- | --- | --- | --- | --- | --- |
| Subset 1 | 5.94% (27.91%) | 8.94% (10.73%) | -5.51% (8.54%) | 5.77% (11.18%) | -7.75% (10.31%) | 1.68% (6.98%) |
| Subset 2 | 7.86% (28.90%) | 8.61% (10.63%) | -5.32% (8.54%) | 5.53% (11.34%) | -7.92% (9.94%) | 0.06% (7.22%) |
| Subset 3 | 7.92% (29.64%) | 8.51% (10.86%) | -5.17% (8.44%) | 5.53% (10.52%) | -8.48% (10.62%) | 1.81% (7.05%) |
| Subset 4 | 6.98% (28.57%) | 9.13% (10.57%) | -5.25% (8.30%) | 5.12% (11.14%) | -7.26% (9.70%) | 0.45% (6.80%) |
| Subset 5 | 7.43% (28.28%) | 8.68% (10.62%) | -5.08% (8.18%) | 5.94% (11.05%) | -8.05% (10.52%) | 1.10% (7.08%) |

While the overall inter-subset variations for the mid-abundance phylotpes were small, the inter-subset variations are larger in phylotypes of relatively higher and lower abundance such as Pseudomonaceae and Oxalobacteraceae.

**Table S2.** Richness and diversity values from additional subsets at OTU definitions of ≥95%, ≥97%, and ≥99% bootstrap confidence level.

Table S2. (A)

| **95% Conf level** | **Pre-circ # of unique phylotypes** | **Post-circ # of unique phylotype** | **Total # of unique phylotype** | **Pre-circ Shannon index** | **Post-circ Shannon index** | **Pre-/Post-circ Shannon index ratio** |
| --- | --- | --- | --- | --- | --- | --- |
| **Subset 1** | 10-23; 16.5 (3.55) | 12-21; 16 (3.25) | 42 | 0.96-2.06; 1.51 (0.34) | 1.08-1.73; 1.41 (0.19) | 1.07 |
| **Subset 2** | 12-21; 16.58 (3.15) | 12-21; 15.33 (2.99) | 41 | 0.92-1.97; 1.51 (0.33) | 1.07-1.59; 1.39 (0.17) | 1.09 |
| **Subset 3** | 12-23; 16.58 (3.58) | 11-18; 15.17 (1.90) | 43 | 0.90-2.01; 1.53 (0.33) | 1.05-1.62; 1.39 (0.18) | 1.10 |
| **Subset 4** | 12-21; 16.08 (3.65) | 11-21; 15.83 (3.41) | 44 | 0.96-2.05; 1.50 (0.32) | 1.01-1.60; 1.39 (0.18) | 1.08 |
| **Subset 5** | 12-22; 17.08 (3.26) | 12-21; 16.25 (3.33) | 44 | 0.88-2.08; 1.54 (0.33) | 1.02-1.57; 1.38 (0.17) | 1.12 |

Table S2. (B)

| **97% Conf**  **level** | **Pre-circ # of unique phylotype** | **Post-circ # of unique phylotype** | **Total # of unique phylotype** | **Pre-circ Shannon index** | **Post-circ Shannon index** | **Pre-/Post-circ Shannon index ratio** |
| --- | --- | --- | --- | --- | --- | --- |
| **Subset 1** | 10-23; 16.00 (3.54) | 11-21; 15.58 (3.23) | 41 | 0.89-2.05; 1.49 (0.35) | 1.06-1.67; 1.40 (0.18) | 1.07 |
| **Subset 2** | 12-21; 16.50 (3.03) | 12-21; 14.42 (2.43) | 41 | 0.89-1.95; 1.50 (0.33) | 1.05-1.58; 1.38 (0.17) | 1.09 |
| **Subset 3** | 11-23; 16.17 (3.69) | 11-18; 14.33 (1.87) | 40 | 0.88-1.99; 1.52 (0.34) | 1.03-1.60; 1.37 (0.18) | 1.10 |
| **Subset 4** | 12-20; 15/67 (3.31) | 11-20; 14.67 (2.99) | 42 | 0.92-2.02; 1.49 (0.32) | 1.00-1.61; 1.38 (0.18) | 1.07 |
| **Subset 5** | 11-21; 16.58 (3.18) | 12-20; 15.25 (2.90) | 43 | 0.85-2.06; 1.52 (0.34) | 1.01-1.56; 1.36 (0.17) | 1.12 |

Table S2. (C)

| **99% Conf level** | **Pre-circ # of unique phylotype** | **Post-circ # of unique phylotype** | **Total # of unique phylotype** | **Pre-circ Shannon index** | **Post-circ Shannon index** | **Pre-/Post-circ Shannon index ratio** |
| --- | --- | --- | --- | --- | --- | --- |
| **Subset 1** | 10-22; 15.92 (3.37) | 11-21; 15.25 (3.28) | 41 | 0.89-2.02; 1.49 (0.35) | 1.06-1.66; 1.39 (0.19) | 1.07 |
| **Subset 2** | 10-20; 15.33 (3.03) | 12-20; 14.08 (2.19) | 40 | 0.86-1.95; 1.49 (0.33) | 1.04-1.58; 1.37 (0.17) | 1.09 |
| **Subset 3** | 11-21; 16.00 (3.38) | 11-18; 14.08 (2.15) | 39 | 0.87-1.99; 1.51 (0.34) | 1.05-1.59; 1.37 (0.18) | 1.10 |
| **Subset 4** | 12-20; 15.58 (3.40) | 10-19; 14.33 (2.99) | 42 | 0.92-2.03; 1.48 (0.33) | 1.00-1.67; 1.37 (0.19) | 1.08 |
| **Subset 5** | 11-21; 16.33 (3.03) | 10-20; 14.75 (2.83) | 42 | 0.85-2.05; 1.52 (0.34) | 1.01-1.55; 1.35 (0.16) | 1.12 |

We assessed the level of variation in richness and diversity values between randomly generated subsets among three bootstrap confidence levels. We found little variation between subsets and between (A) ≥95%, (B) ≥97%, and (C) ≥99% bootstrap confidence levels.

**Table S3.** PerMANOVA analysis using additional subsets based on both abundance and proportional abundance data matrices generated using OTU definitions of ≥95%, ≥97%, and ≥99% bootstrap confidence levels.

Table S3. (A)

| **95% Conf level** | **MRPP based on abundance** | **PerMANOVA based on abundance** | **MRPP based on proportional abundance** | **PerMANOVA based on proportional abundance** |
| --- | --- | --- | --- | --- |
| **Subset 1** | p = 0.016 | p = 0.005 | p = 0.010 | p = 0.008 |
| **Subset 2** | p = 0.009 | p = 0.005 | p = 0.011 | p = 0.005 |
| **Subset 3** | p = 0.009 | p = 0.008 | p = 0.017 | p = 0.011 |
| **Subset 4** | p = 0.008 | p = 0.01 | p = 0.012 | p = 0.004 |
| **Subset 5** | p = 0.015 | p = 0.008 | p = 0.01 | p = 0.004 |

Table S3. (B)

| **97% Conf level** | **MRPP based on abundance** | **PerMANOVA based on abundance** | **MRPP based on proportional abundance** | **PerMANOVA based on proportional abundance** |
| --- | --- | --- | --- | --- |
| **Subset 1** | p = 0.017 | p = 0.007 | p = 0.007 | p = 0.007 |
| **Subset 2** | p = 0.015 | p = 0.004 | p = 0.016 | p = 0.006 |
| **Subset 3** | p = 0.019 | p = 0.013 | p = 0.010 | p = 0.009 |
| **Subset 4** | p = 0.011 | p = 0.009 | p = 0.008 | p = 0.014 |
| **Subset 5** | p = 0.012 | p = 0.011 | p = 0.017 | p = 0.007 |

Table S3. (C)

| **99% Conf level** | **MRPP based on abundance** | **PerMANOVA based on abundance** | **MRPP based on proportional abundance** | **PerMANOVA based on proportional abundance** |
| --- | --- | --- | --- | --- |
| **Subset 1** | p = 0.01 | p = 0.007 | p = 0.01 | p = 0.005 |
| **Subset 2** | p = 0.02 | p = 0.006 | p = 0.01 | p = 0.005 |
| **Subset 3** | p = 0.014 | p = 0.012 | p = 0.011 | p = 0.013 |
| **Subset 4** | p = 0.024 | p = 0.017 | p = 0.012 | p = 0.009 |
| **Subset 5** | p = 0.014 | p = 0.012 | p = 0.011 | p = 0.004 |

In addition, we also compared our PerMANOVA-generated significance-levels with those from the multi-response permutational procedure (MRPP), another permutational method for testing the null-hypothesis of no-difference between community ecological data. (A) PerMANOVA and MRPP results at ≥95% bootstrap confidence level. (B) PerMANOVA and MRPP results at ≥97% bootstrap confidence level. (C) PerMANOVA and MRPP results at ≥99% bootstrap confidence level.

**Table S4.** Indicator species analysis using additional subsets based on both abundance and proportional abundance data matrices generated using OTU definitions of (A) ≥95%, (B) ≥97%, and (C) ≥99% bootstrap confidence levels.

Table S4. (A)

| **95% Conf level** | **Pre-circ indicator species (abundance)** | **Indicator value**  ***p*-value** | **Pre-circ indicator species**  **(prop. abundance)** | **Indicator value**  ***p*-value** | **Post-circ indicator species (abundance)** | **Indicator value**  ***p*-value** | **Post-circ indicator species**  **(prop. abundance)** | **Indicator value**  ***p*-value** |
| --- | --- | --- | --- | --- | --- | --- | --- | --- |
| **Subset 1** | Clostridiales Family XI | 0.83  *p* = 0.006 | Clostridiales Family XI | 0.83  *p* = 0.006 | Corynebacteriaceae | 0.83  *p* = 0.006 | Corynebacteriaceae | 0.81  *p* = 0.005 |
|  | Prevotellaceae | 0.66  *p* = 0.006 | Prevotellaceae | 0.66  *p* = 0.002 | Staphylococcaceae | 0.76  *p* = 0.045 | Staphylococcaceae | 0.76  *p* = 0.038 |
|  |  |  |  |  |  |  |  |  |
| **Subset 2** | Clostridiales Family XI | 0.92  *p* = 0.001 | Clostridiales Family XI | 0.92  *p* = 0.006 | Corynebacteriaceae | 0.81  *p* = 0.003 | Corynebacteriaceae | 0.81  *p* = 0.004 |
|  | Prevotellaceae | 0.74  *p* = 0.003 | Prevotellaceae | 0.74  *p* = 0.002 | Staphylococcaceae | 0.80  *p* = 0.037 | Staphylococcaceae | 0.80  *p* = 0.034 |
|  |  |  |  |  |  |  |  |  |
| **Subset 3** | Clostridiales Family XI | 0.84  *p* = 0.005 | Clostridiales Family XI | 0.84  *p* = 0.003 | Corynebacteriaceae | 0.81  *p* = 0.005 | Corynebacteriaceae | 0.80  *p* = 0.006 |
|  | Prevotellaceae | 0.65  *p* = 0.010 | Prevotellaceae | 0.65  *p* = 0.009 | Staphylococcaceae | 0.77  *p* = 0.007 | Staphylococcaceae | 0.77  *p* = 0.009 |
|  |  |  |  |  |  |  |  |  |
| **Subset 4** | Clostridiales Family XI | 0.74  *p* = 0.030 | Clostridiales Family XI | 0.74  *p* = 0.026 | Corynebacteriaceae | 0.78  *p* = 0.002 | Corynebacteriaceae | 0.81  *p* = 0.003 |
|  | Prevotellaceae | 0.66  p = 0.007 | Prevotellaceae | 0.66  *p* = 0.008 | Staphylococcaceae | 0.78  p = 0.023 | Staphylococcaceae | 0.78  *p* = 0.022 |
|  |  |  |  |  | Xanthomonadaceae* | 0.63  *p* = 0.048 | Xanthomonadaceae* | 0.63  *p* = 0.036 |
|  |  |  |  |  |  |  |  |  |
| **Subset 5** | Clostridiales Family XI | 0.83  *p* = 0.006 | Clostridiales Family XI | 0.83  *p* = 0.004 | Corynebacteriaceae | 0.80  *p* = 0.005 | Corynebacteriaceae | 0.80  *p* = 0.006 |
|  | Prevotellaceae | 0.58  *p* = 0.013 | Prevotellaceae | 0.58  *p* = 0.013 | Staphylococcaceae | 0.76  *p* = 0.034 | Staphylococcaceae | 0.76  *p* = 0.030 |

Table S4. (B)

| **97% Conf level** | **Pre-circ indicator species (abundance)** | **Indicator value**  ***p*-value** | **Pre-circ indicator species**  **(prop. abundance)** | **Indicator value**  ***p*-value** | **Post-circ indicator species (abundance)** | **Indicator value**  ***p*-value** | **Post-circ indicator species**  **(prop. abundance)** | **Indicator value**  ***p*-value** |
| --- | --- | --- | --- | --- | --- | --- | --- | --- |
| **Subset 1** | Clostridiales Family XI | 0.83  *p* = 0.006 | Clostridiales Family XI | 0.83  *p* = 0.006 | Corynebacteriaceae | 0.81  *p* = 0.006 | Corynebacteriaceae | 0.81  *p* = 0.004 |
|  | Prevotellaceae | 0.66  *p* = 0.006 | Prevotellaceae | 0.66  *p* = 0.002 | Staphylococcaceae | 0.75  *p* = 0.044 | Staphylococcaceae | 0.76  *p* = 0.041 |
|  |  |  |  |  |  |  |  |  |
| **Subset 2** | Clostridiales Family XI | 0.92  *p* = 0.001 | Clostridiales Family XI | 0.92  *p* = 0.002 | Corynebacteriaceae | 0.82  *p* = 0.002 | Corynebacteriaceae | 0.82  *p* = 0.002 |
|  | Prevotellaceae | 0.74  *p* = 0.002 | Prevotellaceae | 0.74  *p* = 0.003 | Staphylococcaceae | 0.80  *p* = 0.037 | Staphylococcaceae | 0.81  *p* = 0.031 |
|  |  |  |  |  |  |  | Enterobacteriaceae* | 0.61  *p* = 0.040 |
| **Subset 3** |  |  |  |  |  |  |  |  |
|  | Clostridiales Family XI | 0.84  *p* = 0.005 | Clostridiales Family XI | 0.84  *p* = 0.006 | Corynebacteriaceae | 0.81  *p* = 0.004 | Corynebacteriaceae | 0.81  *p* = 0.004 |
|  | Prevotellaceae | 0.65  *p* = 0.012 | Prevotellaceae | 0.65  *p* = 0.007 | Staphylococcaceae | 0.77  *p* = 0.011 | Staphylococcaceae | 0.77  *p* = 0.006 |
| **Subset 4** |  |  |  |  |  |  |  |  |
|  | Clostridiales Family XI | 0.74  *p* = 0.033 | Clostridiales Family XI | 0.74  *p* = 0.030 | Corynebacteriaceae | 0.83  *p* = 0.002 | Corynebacteriaceae | 0.81  *p* = 0.003 |
|  | Prevotellaceae | 0.66  *p* = 0.008 | Prevotellaceae | 0.66  *p* = 0.007 | Staphylococcaceae | 0.78  *p* = 0.025 | Staphylococcaceae | 0.78  *p* = 0.022 |
|  |  |  |  |  | Xanthomonadaceae* | 0.63  *p* = 0.049 | Xanthomonadaceae* | 0.63  *p* = 0.037 |
| **Subset 5** |  |  |  |  |  |  |  |  |
|  | Clostridiales Family XI | 0.83  *p* = 0.005 | Clostridiales Family XI | 0.83  *p* = 0.008 | Corynebacteriaceae | 0.81  *p* = 0.005 | Corynebacteriaceae | 0.82  *p* = 0.004 |

Table S4. (C)

| **97% Conf level** | **Pre-circ indicator species (abundance)** | **Indicator value**  ***p*-value** | **Pre-circ indicator species**  **(prop. abundance)** | **Indicator value**  ***p*-value** | **Post-circ indicator species (abundance)** | **Indicator value**  ***p*-value** | **Post-circ indicator species**  **(prop. abundance)** | **Indicator value**  ***p*-value** |
| --- | --- | --- | --- | --- | --- | --- | --- | --- |
| **Subset 1** | Clostridiales Family XI | 0.82  *p* = 0.007 | Clostridiales Family XI | 0.83  *p* = 0.007 | Corynebacteriaceae | 0.81  *p* = 0.004 | Corynebacteriaceae | 0.81  *p* = 0.004 |
|  | Prevotellaceae | 0.66  *p* = 0.004 | Prevotellaceae | 0.66  *p* = 0.003 | Staphylococcaceae | 0.75  *p* = 0.045 | Staphylococcaceae | 0.76  *p* = 0.034 |
|  |  |  |  |  |  |  |  |  |
| **Subset 2** | Clostridiales Family XI | 0.92  *p* = 0.002 | Clostridiales Family XI | 0.92  *p* = 0.002 | Corynebacteriaceae | 0.83  *p* = 0.002 | Corynebacteriaceae | 0.83  *p* = 0.003 |
|  | Prevotellaceae | 0.74  *p* = 0.002 | Prevotellaceae | 0.74  *p* = 0.003 | Staphylococcaceae | 0.80  *p* = 0.037 | Staphylococcaceae | 0.81  *p* = 0.029 |
|  |  |  |  |  |  |  |  |  |
| **Subset 3** | Clostridiales Family XI | 0.84  *p* = 0.006 | Clostridiales Family XI | 0.84  *p* = 0.006 | Corynebacteriaceae | 0.81  *p* = 0.004 | Corynebacteriaceae | 0.82  *p* = 0.004 |
|  | Prevotellaceae | 0.65  *p* = 0.011 | Prevotellaceae | 0.65  *p* = 0.010 | Staphylococcaceae | 0.77  *p* = 0.007 | Staphylococcaceae | 0.77  *p* = 0.007 |
|  |  |  |  |  |  |  |  |  |
| **Subset 4** | Clostridiales Family XI | 0.74  *p* = 0.034 | Clostridiales Family XI | 0.74  *p* = 0.033 | Corynebacteriaceae | 0.73  *p* = 0.003 | Corynebacteriaceae | 0.83  *p* = 0.002 |
|  | Prevotellaceae | 0.66  *p* = 0.009 | Prevotellaceae | 0.66  *p* = 0.007 | Staphylococcaceae | 0.78  *p* = 0.024 | Staphylococcaceae | 0.78  *p* = 0.022 |
|  |  |  |  |  | Xanthomonadaceae* | 0.63  *p* = 0.050 | Xanthomonadaceae* | 0.63  *p* = 0.035 |
|  |  |  |  |  |  |  |  |  |
| **Subset 5** | Clostridiales Family XI | 0.83  *p* = 0.007 | Clostridiales Family XI | 0.83  *p* = 0.007 | Corynebacteriaceae | 0.82  *p* = 0.003 | Corynebacteriaceae | 0.82  *p* = 0.004 |
|  | Prevotellaceae | 0.58  *p* = 0.014 | Prevotellaceae | 0.58  *p* = 0.014 | Staphylococcaceae | 0.76  *p* = 0.037 | Staphylococcaceae | 0.76  *p* = 0.028 |
