# Supplementary figures and images for: The Effects of Circumcision on the Penis Microbiome
Source: PLoS One. 2010 Jan 6;5(1):e8422. doi: 10.1371/journal.pone.0008422 (PMC2798966; doi:10.1371/journal.pone.0008422)

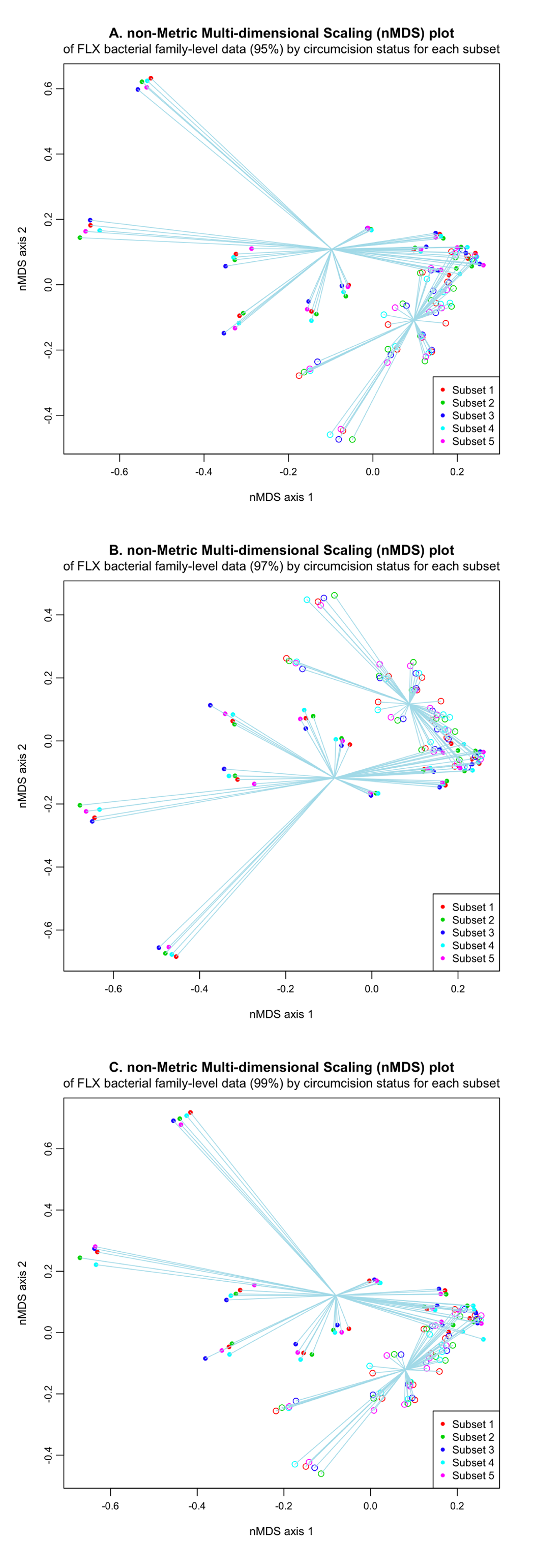

Supplement: Figure S1 — Confirmation of nMDS ordination using multiple subsets. nMDS plots generated using additional subsets at an OTU definition of > = 95%, > = 97%, and > = 99% bootstrap confidence levels, which demonstrated high level of consistency between subsets and among the different levels of OUT definitions. The two-factor PerMANOVA test using the > = 95% bootstrap confidence level dataset (A) comparing the difference between subsets found no difference between subsets (p = 1.00), whereas a significant difference was found between pre-circumcision and post-circumcision coronal sulci microbiota (p = 0.001). Additional PerMANOVA tests using (B) > = 97% and (C) > = 99% bootstrap confidence level datasets showed similar results. (solid dot = pre-circumcision samples; circles = post-circumcision samples). (9.74 MB TIF) [file pone.0008422.s002.tif]

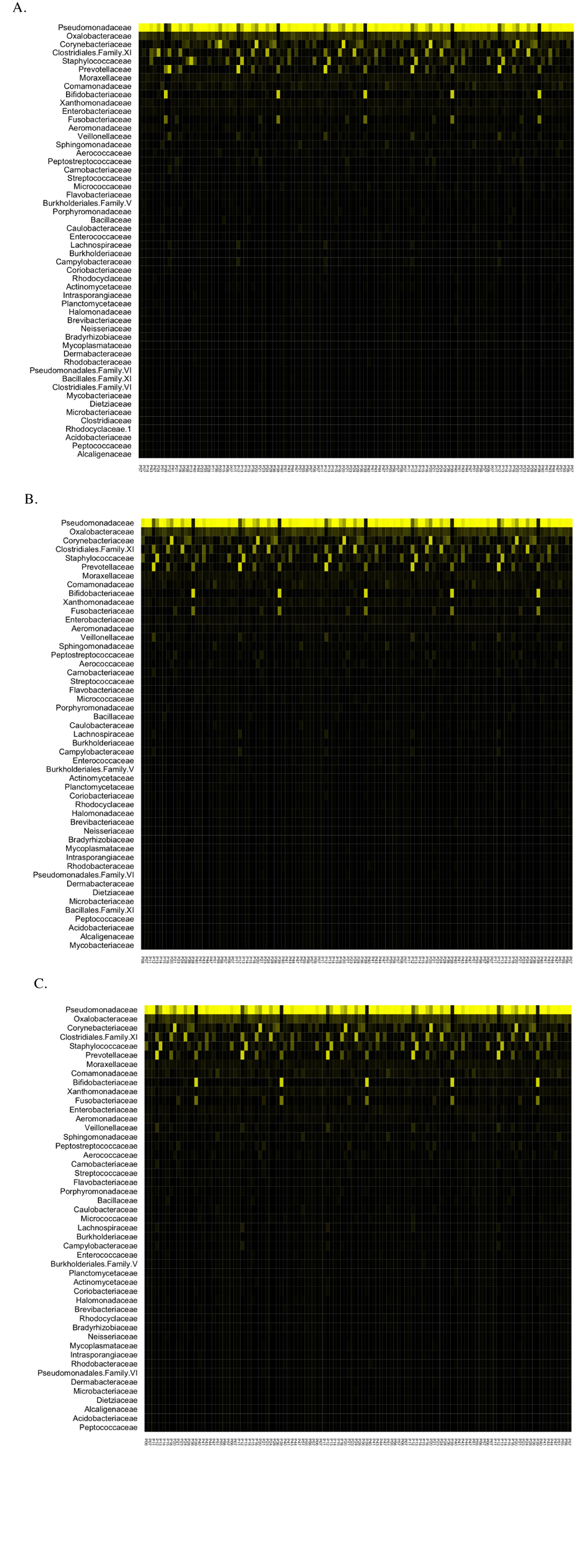

Supplement: Figure S2 — Heatmap plots generated using all five data subsets. Heatmap plots generated using phylotype abundance data from additional subsets at > = 95%, > = 97%, and > = 99% bootstrap confidence levels. The same trend in phylotype abundances is observed among the subsets and three OTU definitions: (A) > = 95%, (B) > = 97%, and (C) > = 99% bootstrap confidence levels. (8.93 MB TIF) [file pone.0008422.s003.tif]

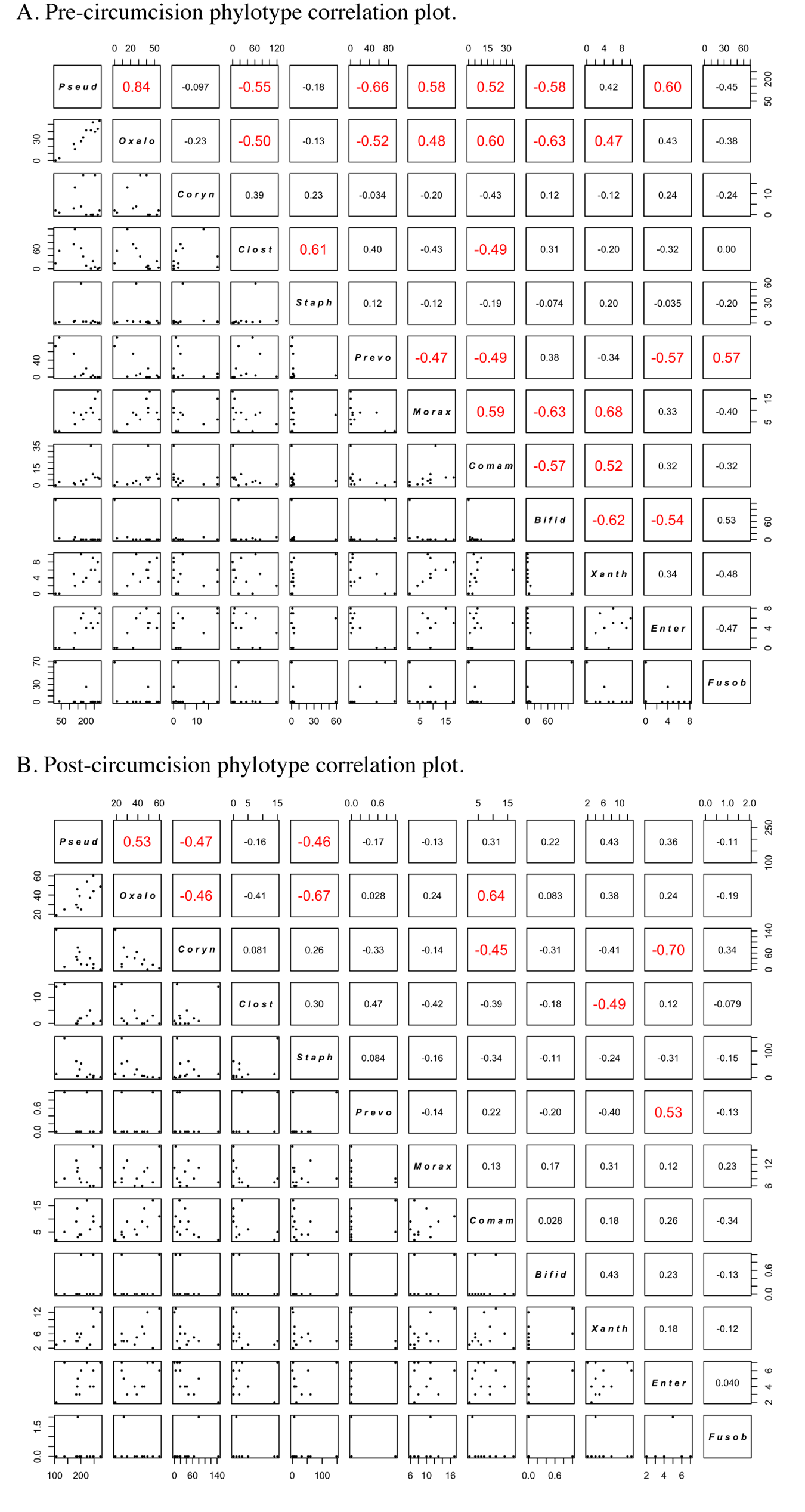

Supplement: Figure S3 — Phylotype correlation plots of the ten most abundant phylotypes found in the pre-circumcision and the post-circumcision coronal sulci microbiota. Phylotype correlation plots of the ten most abundant phylotypes found in the (A) pre-circumcision and the (B) post-circumcision coronal sulci microbiota. In these correlation plots, each phylotype is compared to all nine remaining phylotypes graphically (lower half of the correlation plot) and statistically (upper half of the correlation plot). In the graphical comparison, two phylotypes are plotted against each other in each sub-plot, with one phylotype's abundance on the x-axis and another phylotype's abundance on the y-axis. In the statistical comparison, the Kendall's τ is calculated to evaluate the correlation, with the statistically significant τ values highlighted in red. In contrast to the pre-circumcision correlation plot (A), where 26 potential correlations were observed, only 10 potential correlations were observed among the post-circumcision samples (B). Abbreviations: Pseudo = Pseudomonadaceae, Oxalo = Oxalobacteraceae, Coryn = Corynebacteriaceae, Clost = Clostridiales Family XI, Staph = Staphylococcaceae, Prevo = Prevotellaceae, Morax = Moraxellaceae, Comam = Comamonadaceae, Bifid = Bifidobacteriaceae, Xanth = Xanthomonadaceae, Enter = Enterobacteriaceae, Fusob = Fusobacteriaceae. (10.29 MB TIF) [file pone.0008422.s004.tif]

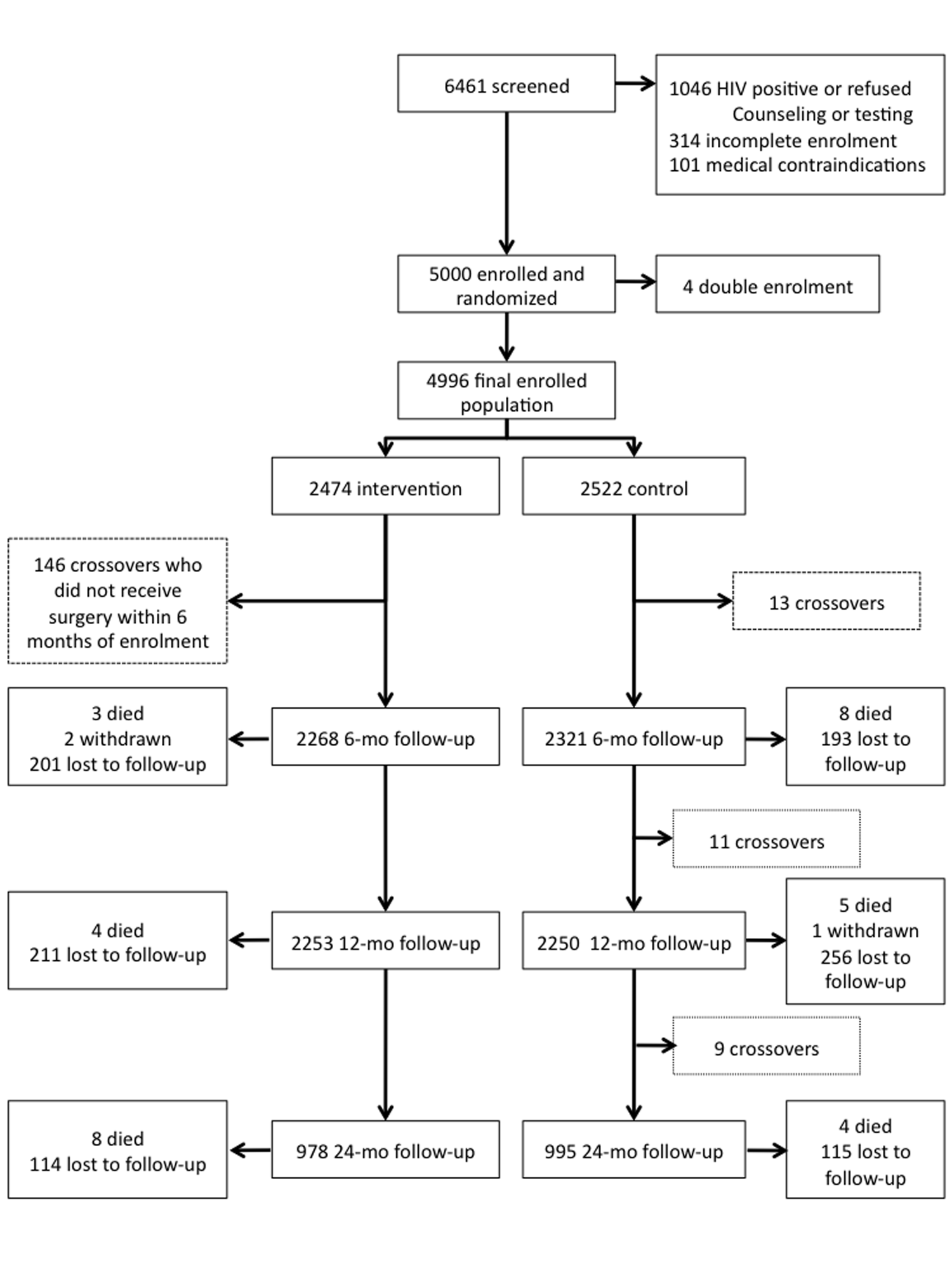

Supplement: Figure S4 — CONSORT diagram from the randomized control trial (12.02 MB TIF) [file pone.0008422.s005.tif]
