# Supplementary material for: The Effects of Circumcision on the Penis Microbiome
Source: PLoS One. 2010 Jan 6;5(1):e8422. doi: 10.1371/journal.pone.0008422 (PMC2798966; doi:10.1371/journal.pone.0008422)
Supplement: Table S1 — Circumcision-associated % change and standard deviations of the six most abundant phylotypes found in coronal sulci microbiota generated from additional subsets at OTU definitions of > = 95% bootstrap confidence level. While the overall inter-subset variations for the mid-abundance phylotpes were small, the inter-subset variations are larger in phylotypes of relatively higher and lower abundance such as Pseudomonaceae and Oxalobacteraceae. (0.03 MB DOC) [file pone.0008422.s006.doc]

| **95% Conf level** | **Pseudomonadaceae**  (mean % change; SD) | **Corynebacteriaceae** (mean % change; SD) | **Prevotellaceae**  (mean % change; SD) | **Staphylococcaceae**  (mean % change; SD) | **Clostridiales Family XI** (mean % change; SD) | **Oxalobacteraceae** (mean % change; SD) |
| --- | --- | --- | --- | --- | --- | --- |
| Subset 1 | 5.94% (27.91%) | 8.94% (10.73%) | -5.51% (8.54%) | 5.77% (11.18%) | -7.75% (10.31%) | 1.68% (6.98%) |
| Subset 2 | 7.86% (28.90%) | 8.61% (10.63%) | -5.32% (8.54%) | 5.53% (11.34%) | -7.92% (9.94%) | 0.06% (7.22%) |
| Subset 3 | 7.92% (29.64%) | 8.51% (10.86%) | -5.17% (8.44%) | 5.53% (10.52%) | -8.48% (10.62%) | 1.81% (7.05%) |
| Subset 4 | 6.98% (28.57%) | 9.13% (10.57%) | -5.25% (8.30%) | 5.12% (11.14%) | -7.26% (9.70%) | 0.45% (6.80%) |
| Subset 5 | 7.43% (28.28%) | 8.68% (10.62%) | -5.08% (8.18%) | 5.94% (11.05%) | -8.05% (10.52%) | 1.10% (7.08%) |
