# Supplementary material for: The Effects of Circumcision on the Penis Microbiome
Source: PLoS One. 2010 Jan 6;5(1):e8422. doi: 10.1371/journal.pone.0008422 (PMC2798966; doi:10.1371/journal.pone.0008422)
Supplement: Table S2 — Richness and diversity values from additional subsets at OTU definitions of > = 95%, > = 97%, and > = 99% bootstrap confidence level. We assessed the level of variation in richness and diversity values between randomly generated subsets among three bootstrap confidence levels. We found little variation between subsets and between (A) > = 95%, (B) > = 97%, and (C) > = 99% bootstrap confidence levels. (0.06 MB DOC) [file pone.0008422.s007.doc]

A.

| **95% Conf level** | **Pre-circ # of unique phylotypes** | **Post-circ # of unique phylotype** | **Total # of unique phylotype** | **Pre-circ Shannon index** | **Post-circ Shannon index** | **Pre-/Post-circ Shannon index ratio** |
| --- | --- | --- | --- | --- | --- | --- |
| **Subset 1** | 10-23; 16.5 (3.55) | 12-21; 16 (3.25) | 42 | 0.96-2.06; 1.51 (0.34) | 1.08-1.73; 1.41 (0.19) | 1.07 |
| **Subset 2** | 12-21; 16.58 (3.15) | 12-21; 15.33 (2.99) | 41 | 0.92-1.97; 1.51 (0.33) | 1.07-1.59; 1.39 (0.17) | 1.09 |
| **Subset 3** | 12-23; 16.58 (3.58) | 11-18; 15.17 (1.90) | 43 | 0.90-2.01; 1.53 (0.33) | 1.05-1.62; 1.39 (0.18) | 1.10 |
| **Subset 4** | 12-21; 16.08 (3.65) | 11-21; 15.83 (3.41) | 44 | 0.96-2.05; 1.50 (0.32) | 1.01-1.60; 1.39 (0.18) | 1.08 |
| **Subset 5** | 12-22; 17.08 (3.26) | 12-21; 16.25 (3.33) | 44 | 0.88-2.08; 1.54 (0.33) | 1.02-1.57; 1.38 (0.17) | 1.12 |

B.

| **97% Conf**  **level** | **Pre-circ # of unique phylotype** | **Post-circ # of unique phylotype** | **Total # of unique phylotype** | **Pre-circ Shannon index** | **Post-circ Shannon index** | **Pre-/Post-circ Shannon index ratio** |
| --- | --- | --- | --- | --- | --- | --- |
| **Subset 1** | 10-23; 16.00 (3.54) | 11-21; 15.58 (3.23) | 41 | 0.89-2.05; 1.49 (0.35) | 1.06-1.67; 1.40 (0.18) | 1.07 |
| **Subset 2** | 12-21; 16.50 (3.03) | 12-21; 14.42 (2.43) | 41 | 0.89-1.95; 1.50 (0.33) | 1.05-1.58; 1.38 (0.17) | 1.09 |
| **Subset 3** | 11-23; 16.17 (3.69) | 11-18; 14.33 (1.87) | 40 | 0.88-1.99; 1.52 (0.34) | 1.03-1.60; 1.37 (0.18) | 1.10 |
| **Subset 4** | 12-20; 15/67 (3.31) | 11-20; 14.67 (2.99) | 42 | 0.92-2.02; 1.49 (0.32) | 1.00-1.61; 1.38 (0.18) | 1.07 |
| **Subset 5** | 11-21; 16.58 (3.18) | 12-20; 15.25 (2.90) | 43 | 0.85-2.06; 1.52 (0.34) | 1.01-1.56; 1.36 (0.17) | 1.12 |

C.

| **99% Conf level** | **Pre-circ # of unique phylotype** | **Post-circ # of unique phylotype** | **Total # of unique phylotype** | **Pre-circ Shannon index** | **Post-circ Shannon index** | **Pre-/Post-circ Shannon index ratio** |
| --- | --- | --- | --- | --- | --- | --- |
| **Subset 1** | 10-22; 15.92 (3.37) | 11-21; 15.25 (3.28) | 41 | 0.89-2.02; 1.49 (0.35) | 1.06-1.66; 1.39 (0.19) | 1.07 |
| **Subset 2** | 10-20; 15.33 (3.03) | 12-20; 14.08 (2.19) | 40 | 0.86-1.95; 1.49 (0.33) | 1.04-1.58; 1.37 (0.17) | 1.09 |
| **Subset 3** | 11-21; 16.00 (3.38) | 11-18; 14.08 (2.15) | 39 | 0.87-1.99; 1.51 (0.34) | 1.05-1.59; 1.37 (0.18) | 1.10 |
| **Subset 4** | 12-20; 15.58 (3.40) | 10-19; 14.33 (2.99) | 42 | 0.92-2.03; 1.48 (0.33) | 1.00-1.67; 1.37 (0.19) | 1.08 |
| **Subset 5** | 11-21; 16.33 (3.03) | 10-20; 14.75 (2.83) | 42 | 0.85-2.05; 1.52 (0.34) | 1.01-1.55; 1.35 (0.16) | 1.12 |
