# Supplementary material for: The Effects of Circumcision on the Penis Microbiome
Source: PLoS One. 2010 Jan 6;5(1):e8422. doi: 10.1371/journal.pone.0008422 (PMC2798966; doi:10.1371/journal.pone.0008422)
Supplement: Table S3 — PerMANOVA analysis using additional subsets based on both abundance and proportional abundance data matrices generated using OTU definitions of > = 95%, > = 97%, and > = 99% bootstrap confidence levels. In addition, we also compared our PerMANOVA-generated significance-levels with those from the multi-response permutational procedure (MRPP), another permutational method for testing the null-hypothesis of no-difference between community ecological data. (A) PerMANOVA and MRPP results at > = 95% bootstrap confidence level. (B) PerMANOVA and MRPP results at > = 97% bootstrap confidence level. (C) PerMANOVA and MRPP results at > = 99% bootstrap confidence level. (0.04 MB DOC) [file pone.0008422.s008.doc]

A.

| **95% Conf level** | **MRPP based on abundance** | **PerMANOVA based on abundance** | **MRPP based on proportional abundance** | **PerMANOVA based on proportional abundance** |
| --- | --- | --- | --- | --- |
| **Subset 1** | p = 0.016 | p = 0.005 | p = 0.010 | p = 0.008 |
| **Subset 2** | p = 0.009 | p = 0.005 | p = 0.011 | p = 0.005 |
| **Subset 3** | p = 0.009 | p = 0.008 | p = 0.017 | p = 0.011 |
| **Subset 4** | p = 0.008 | p = 0.01 | p = 0.012 | p = 0.004 |
| **Subset 5** | p = 0.015 | p = 0.008 | p = 0.01 | p = 0.004 |

B.

| **97% Conf level** | **MRPP based on abundance** | **PerMANOVA based on abundance** | **MRPP based on proportional abundance** | **PerMANOVA based on proportional abundance** |
| --- | --- | --- | --- | --- |
| **Subset 1** | p = 0.017 | p = 0.007 | p = 0.007 | p = 0.007 |
| **Subset 2** | p = 0.015 | p = 0.004 | p = 0.016 | p = 0.006 |
| **Subset 3** | p = 0.019 | p = 0.013 | p = 0.010 | p = 0.009 |
| **Subset 4** | p = 0.011 | p = 0.009 | p = 0.008 | p = 0.014 |
| **Subset 5** | p = 0.012 | p = 0.011 | p = 0.017 | p = 0.007 |

C.

| **99% Conf level** | **MRPP based on abundance** | **PerMANOVA based on abundance** | **MRPP based on proportional abundance** | **PerMANOVA based on proportional abundance** |
| --- | --- | --- | --- | --- |
| **Subset 1** | p = 0.01 | p = 0.007 | p = 0.01 | p = 0.005 |
| **Subset 2** | p = 0.02 | p = 0.006 | p = 0.01 | p = 0.005 |
| **Subset 3** | p = 0.014 | p = 0.012 | p = 0.011 | p = 0.013 |
| **Subset 4** | p = 0.024 | p = 0.017 | p = 0.012 | p = 0.009 |
| **Subset 5** | p = 0.014 | p = 0.012 | p = 0.011 | p = 0.004 |
