# Supplementary material for: The Effects of Circumcision on the Penis Microbiome
Source: PLoS One. 2010 Jan 6;5(1):e8422. doi: 10.1371/journal.pone.0008422 (PMC2798966; doi:10.1371/journal.pone.0008422)
Supplement: Table S4 — Indicator species analysis using additional subsets based on both abundance and proportional abundance data matrices generated using OTU definitions of (A) > = 95%, (B) > = 97%, and (C) > = 99% bootstrap confidence levels. (0.11 MB DOC) [file pone.0008422.s009.doc]

A.

| **95% Conf level** | **Pre-circ indicator species (abundance)** | **Indicator value**  ***p*-value** | **Pre-circ indicator species**  **(prop. abundance)** | **Indicator value**  ***p*-value** | **Post-circ indicator species (abundance)** | **Indicator value**  ***p*-value** | **Post-circ indicator species**  **(prop. abundance)** | **Indicator value**  ***p*-value** |
| --- | --- | --- | --- | --- | --- | --- | --- | --- |
| **Subset 1** | Clostridiales Family XI | 0.83  *p* = 0.006 | Clostridiales Family XI | 0.83  *p* = 0.006 | Corynebacteriaceae | 0.83  *p* = 0.006 | Corynebacteriaceae | 0.81  *p* = 0.005 |
|  | Prevotellaceae | 0.66  *p* = 0.006 | Prevotellaceae | 0.66  *p* = 0.002 | Staphylococcaceae | 0.76  *p* = 0.045 | Staphylococcaceae | 0.76  *p* = 0.038 |
|  |  |  |  |  |  |  |  |  |
| **Subset 2** | Clostridiales Family XI | 0.92  *p* = 0.001 | Clostridiales Family XI | 0.92  *p* = 0.006 | Corynebacteriaceae | 0.81  *p* = 0.003 | Corynebacteriaceae | 0.81  *p* = 0.004 |
|  | Prevotellaceae | 0.74  *p* = 0.003 | Prevotellaceae | 0.74  *p* = 0.002 | Staphylococcaceae | 0.80  *p* = 0.037 | Staphylococcaceae | 0.80  *p* = 0.034 |
|  |  |  |  |  |  |  |  |  |
| **Subset 3** | Clostridiales Family XI | 0.84  *p* = 0.005 | Clostridiales Family XI | 0.84  *p* = 0.003 | Corynebacteriaceae | 0.81  *p* = 0.005 | Corynebacteriaceae | 0.80  *p* = 0.006 |
|  | Prevotellaceae | 0.65  *p* = 0.010 | Prevotellaceae | 0.65  *p* = 0.009 | Staphylococcaceae | 0.77  *p* = 0.007 | Staphylococcaceae | 0.77  *p* = 0.009 |
|  |  |  |  |  |  |  |  |  |
| **Subset 4** | Clostridiales Family XI | 0.74  *p* = 0.030 | Clostridiales Family XI | 0.74  *p* = 0.026 | Corynebacteriaceae | 0.78  *p* = 0.002 | Corynebacteriaceae | 0.81  *p* = 0.003 |
|  | Prevotellaceae | 0.66  p = 0.007 | Prevotellaceae | 0.66  *p* = 0.008 | Staphylococcaceae | 0.78  p = 0.023 | Staphylococcaceae | 0.78  *p* = 0.022 |
|  |  |  |  |  | Xanthomonadaceae* | 0.63  *p* = 0.048 | Xanthomonadaceae* | 0.63  *p* = 0.036 |
|  |  |  |  |  |  |  |  |  |
| **Subset 5** | Clostridiales Family XI | 0.83  *p* = 0.006 | Clostridiales Family XI | 0.83  *p* = 0.004 | Corynebacteriaceae | 0.80  *p* = 0.005 | Corynebacteriaceae | 0.80  *p* = 0.006 |
|  | Prevotellaceae | 0.58  *p* = 0.013 | Prevotellaceae | 0.58  *p* = 0.013 | Staphylococcaceae | 0.76  *p* = 0.034 | Staphylococcaceae | 0.76  *p* = 0.030 |

B.

| **97% Conf level** | **Pre-circ indicator species (abundance)** | **Indicator value**  ***p*-value** | **Pre-circ indicator species**  **(prop. abundance)** | **Indicator value**  ***p*-value** | **Post-circ indicator species (abundance)** | **Indicator value**  ***p*-value** | **Post-circ indicator species**  **(prop. abundance)** | **Indicator value**  ***p*-value** |
| --- | --- | --- | --- | --- | --- | --- | --- | --- |
| **Subset 1** | Clostridiales Family XI | 0.83  *p* = 0.006 | Clostridiales Family XI | 0.83  *p* = 0.006 | Corynebacteriaceae | 0.81  *p* = 0.006 | Corynebacteriaceae | 0.81  *p* = 0.004 |
|  | Prevotellaceae | 0.66  *p* = 0.006 | Prevotellaceae | 0.66  *p* = 0.002 | Staphylococcaceae | 0.75  *p* = 0.044 | Staphylococcaceae | 0.76  *p* = 0.041 |
|  |  |  |  |  |  |  |  |  |
| **Subset 2** | Clostridiales Family XI | 0.92  *p* = 0.001 | Clostridiales Family XI | 0.92  *p* = 0.002 | Corynebacteriaceae | 0.82  *p* = 0.002 | Corynebacteriaceae | 0.82  *p* = 0.002 |
|  | Prevotellaceae | 0.74  *p* = 0.002 | Prevotellaceae | 0.74  *p* = 0.003 | Staphylococcaceae | 0.80  *p* = 0.037 | Staphylococcaceae | 0.81  *p* = 0.031 |
|  |  |  |  |  |  |  | Enterobacteriaceae* | 0.61  *p* = 0.040 |
| **Subset 3** |  |  |  |  |  |  |  |  |
|  | Clostridiales Family XI | 0.84  *p* = 0.005 | Clostridiales Family XI | 0.84  *p* = 0.006 | Corynebacteriaceae | 0.81  *p* = 0.004 | Corynebacteriaceae | 0.81  *p* = 0.004 |
|  | Prevotellaceae | 0.65  *p* = 0.012 | Prevotellaceae | 0.65  *p* = 0.007 | Staphylococcaceae | 0.77  *p* = 0.011 | Staphylococcaceae | 0.77  *p* = 0.006 |
| **Subset 4** |  |  |  |  |  |  |  |  |
|  | Clostridiales Family XI | 0.74  *p* = 0.033 | Clostridiales Family XI | 0.74  *p* = 0.030 | Corynebacteriaceae | 0.83  *p* = 0.002 | Corynebacteriaceae | 0.81  *p* = 0.003 |
|  | Prevotellaceae | 0.66  *p* = 0.008 | Prevotellaceae | 0.66  *p* = 0.007 | Staphylococcaceae | 0.78  *p* = 0.025 | Staphylococcaceae | 0.78  *p* = 0.022 |
|  |  |  |  |  | Xanthomonadaceae* | 0.63  *p* = 0.049 | Xanthomonadaceae* | 0.63  *p* = 0.037 |
| **Subset 5** |  |  |  |  |  |  |  |  |
|  | Clostridiales Family XI | 0.83  *p* = 0.005 | Clostridiales Family XI | 0.83  *p* = 0.008 | Corynebacteriaceae | 0.81  *p* = 0.005 | Corynebacteriaceae | 0.82  *p* = 0.004 |

C.

| **97% Conf level** | **Pre-circ indicator species (abundance)** | **Indicator value**  ***p*-value** | **Pre-circ indicator species**  **(prop. abundance)** | **Indicator value**  ***p*-value** | **Post-circ indicator species (abundance)** | **Indicator value**  ***p*-value** | **Post-circ indicator species**  **(prop. abundance)** | **Indicator value**  ***p*-value** |
| --- | --- | --- | --- | --- | --- | --- | --- | --- |
| **Subset 1** | Clostridiales Family XI | 0.83  *p* = 0.006 | Clostridiales Family XI | 0.83  *p* = 0.006 | Corynebacteriaceae | 0.81  *p* = 0.006 | Corynebacteriaceae | 0.81  *p* = 0.004 |
|  | Prevotellaceae | 0.66  *p* = 0.006 | Prevotellaceae | 0.66  *p* = 0.002 | Staphylococcaceae | 0.75  *p* = 0.044 | Staphylococcaceae | 0.76  *p* = 0.041 |
|  |  |  |  |  |  |  |  |  |
| **Subset 2** | Clostridiales Family XI | 0.92  *p* = 0.001 | Clostridiales Family XI | 0.92  *p* = 0.002 | Corynebacteriaceae | 0.82  *p* = 0.002 | Corynebacteriaceae | 0.82  *p* = 0.002 |
|  | Prevotellaceae | 0.74  *p* = 0.002 | Prevotellaceae | 0.74  *p* = 0.003 | Staphylococcaceae | 0.80  *p* = 0.037 | Staphylococcaceae | 0.81  *p* = 0.031 |
|  |  |  |  |  |  |  | Enterobacteriaceae* | 0.61  *p* = 0.040 |
| **Subset 3** |  |  |  |  |  |  |  |  |
|  | Clostridiales Family XI | 0.84  *p* = 0.005 | Clostridiales Family XI | 0.84  *p* = 0.006 | Corynebacteriaceae | 0.81  *p* = 0.004 | Corynebacteriaceae | 0.81  *p* = 0.004 |
|  | Prevotellaceae | 0.65  *p* = 0.012 | Prevotellaceae | 0.65  *p* = 0.007 | Staphylococcaceae | 0.77  *p* = 0.011 | Staphylococcaceae | 0.77  *p* = 0.006 |
| **Subset 4** |  |  |  |  |  |  |  |  |
|  | Clostridiales Family XI | 0.74  *p* = 0.033 | Clostridiales Family XI | 0.74  *p* = 0.030 | Corynebacteriaceae | 0.83  *p* = 0.002 | Corynebacteriaceae | 0.81  *p* = 0.003 |
|  | Prevotellaceae | 0.66  *p* = 0.008 | Prevotellaceae | 0.66  *p* = 0.007 | Staphylococcaceae | 0.78  *p* = 0.025 | Staphylococcaceae | 0.78  *p* = 0.022 |
|  |  |  |  |  | Xanthomonadaceae* | 0.63  *p* = 0.049 | Xanthomonadaceae* | 0.63  *p* = 0.037 |
| **Subset 5** |  |  |  |  |  |  |  |  |
|  | Clostridiales Family XI | 0.83  *p* = 0.005 | Clostridiales Family XI | 0.83  *p* = 0.008 | Corynebacteriaceae | 0.81  *p* = 0.005 | Corynebacteriaceae | 0.82  *p* = 0.004 |
