# Supplementary material for: The Effects of Circumcision on the Penis Microbiome
Source: PLoS One. 2010 Jan 6;5(1):e8422. doi: 10.1371/journal.pone.0008422 (PMC2798966; doi:10.1371/journal.pone.0008422)
Supplement: Protocol S1 — Rakai Randomized Control Trial Protocol (1.37 MB DOC) [file pone.0008422.s010.doc]

**CIRCUMCISION: HIV/STIS and BEHAVIORS in a RCT and POST-RCT SURVEILLANCE, RAKAI, Uganda**

# U01-AI075115-01A1

**PROTOCOL**

**With Appendices**

**A Follow-up Study to: A Randomized Clinical Trial of Male Circumcision for HIV Prevention - Rakai, Uganda**

**(U01-AI051171-01A2)**

Funded by:

National Institute of Allergy and Infectious Diseases (NIAID)

National Institutes of Health (NIH)

# Protocol Team

Principal Investigator: Ronald H. Gray, Johns Hopkins University

Co-Investigators: Maria J. Wawer, Johns Hopkins University

Larry Moulton, Johns Hopkins University

Mei-Cheng Wang, Johns Hopkins University

David Serwadda, Makerere University

Nelson Sewankambo, Makerere University

Fred Wabwire-Mangen, Makerere University

Godfrey Kigozi, Rakai Health Sciences Program

Tom Lutalo, Uganda Virus Research institute

Stephen Watya, Makerere University

Noah Kiwanuka, Rakai Health Sciences Program

Fred Nalugoda, Rakai Health Sciences Program

Medical Monitor &

Program Officer: Melanie Bacon, NIH, NIAID, Division of AIDS (DAIDS)

Study Site: Rakai District, Uganda

Period of Study: 7/1/07 to 6/30/12

Contact Address:

Ronald H. Gray MD MSc

Robertson Professor of Reproductive Epidemiology

Johns Hopkins University

Bloomberg School of Public Health

615 N. Wolfe Street / E4132A

Baltimore, MD 21205

Tel. 410-955-7818

Fax. 410-614-7386

E-mail [rgray@jhsph.edu](mailto:rgray@jhsph.edu)

**TABLE OF CONTENTS**

**1.0 Background and Rationale....………………………………………………………………………………..........4**

1.1 A Randomized Clinical Trial of Male Circumcision for HIV Prevention, Rakai-Uganda

(U01-AI051171-01A2)………………………………………………………………………….............................4

1.2 Follow-Up Study, Post Trial Surveillance, Rakai (U01-AI075115-01A1)………………………..................4

**2.0 Specific Aims…………………………………………………………………………………………….................4**

2.1 AIM 1 - The efficacy of male circumcision for STI prevention during trial follow up…………….................4

2.2 AIM 2 - Longer-term post-trial follow-up………………………………………………………….................5

2.2.1 Completion of circumcision surgeries for trial participants……………………………..................5

2.2.2 To assess the effectiveness of circumcision for HIV and STI prevention,

and the effects of circumcision on sexual risk behaviors:…………………………………….................5

2.2.3 Long-term qualitative studies of risk behaviors……………………………………………………6

2.2.4 Estimating the number of circumcisions per HIV infection averted and the

cost per infection averted……………………………………………………………………………......6

**3.0 Study Hypotheses………………………………………………………………………………………................7**

3.1 Efficacy of circumcision for STI prevention (based on assays of samples collected

during the trial)……………………………………………………………………………………………….......7

3.2 Long-term post-trial effectiveness of male circumcision for prevention of HIV

and STIs, and the effects of circumcision on sexual risk behaviors………………………………………….......7

**4.0 Study design, Procedures, and Serious Adverse Events………………………………………………………..7**

4.1 AIM 1 - The efficacy of male circumcision for STI prevention during trial follow-up………………….......7

4.1.1 Completion of laboratory STI assays on stored samples and analyses

to estimate the efficacy of circumcision for male STI prevention………………………………………7

4.1.2 Sampling strategy for STI detection………………………………………………………………8

4.1.3 STI symptoms………………………………………………………………………………........8

4.2 AIM 2 - Longer-term post-trial follow-up……………………………………………………………….......9

4.2.1 Long-term incidence of HIV, STI’s, and risk behaviors after completion

of the trial…………………………………………………………………………………….................9

4.3 Study Procedures…………………………………………………………………………………...............10

4.4 Serious Adverse Events…………………………………………………………………………………….12

**5.0 Laboratory methods for HIV/STI detection………………………..................................................................19**

**6.0 Study population and sample size/power calculations…………………………………………......................20**

Figure 1………………………………………………………………………………………………………….21

Aim 1……………………………………………………………………………………….……………...........21

Table 2…………………………………………………………………………………………………………..22

Aim 2…………………………………………………………………………………………………................23

Table 3…………………………………………………………………………………………………………..24

**7.0 Statistical analysis plan…………………………………………………………………………………………25**

**8.0 Timeline …………………………………………………………………………………………………………29**

**9.0 Literature cited ……..…………………………………………………………………………………………..29**

**Appendix 1 - Information sheet for Male Participants at Unblinding of Trial Outcomes……………………..39**

**Appendix 2 – Community Message on Adult Male Circumcision……………………………………………….43**

**Appendix 3 - Consent Form - Post RCT Rakai Surveillance , English Version……………………………….45**

**Appendix 3A – Consent Form - Post RCT Rakai Surveillance - Luganda Translation……………………..50**

**Appendix 4 – Consent Form - Rakai Community Cohort Study (RCCS), Baseline & Follow-up…………….56**

**Appendix 4A - Consent Form - Rakai Community Cohort Study (RCCS), Baseline & Follow-up –**

**Luganda Translation……………………………………………………………………………………………….64**

**Appendix 5 – Consent Form - Surgical Consent for Male Circumcision………………………………………65**

**Appendix 5A - Consent Form - Surgical Consent for Male Circumcision – Luganda Translation…………..72**

**Appendix 6 – Follow-up Questionnaire for Post RCT Rakai Surveillance (also, RCCS Follow-up Questionnaire)………………………………………………………………………………………………………73**

**Appendix 7 – Baseline Questionnaire for Post CRT Rakai Surveillance (also, RCCS Baseline Questionnaire)……………………………………………………………………………………………………..124**

**Appendix 8 - Original Protocol & Baseline Questionnaire: A Randomized Clinical Trial of Male Circumcisionfor HIV Prevention - Rakai, Uganda (U01-AI051171-01-A2………………………..…………..181**

**1.0 BACKGROUND AND RATIONALE**

**1.1 A RANDOMIZED CLINICAL TRIAL OF MALE CIRCUMCISION FOR HIV PREVENTION, RAKAI-UGANDA (U01-AI051171-01A2)**

The NIH-funded, randomized clinical trial (RCT) of male circumcision (MC) for HIV prevention in Rakai, Uganda, was unblinded by DAIDS on December 12, 2006 upon the recommendation of the NIAID Data and Safety Monitoring Board (DSMB) following its review of an unscheduled interim analysis. The analysis demonstrated efficacy of MC in reducing HIV incidence. Data from the Ugandan RCT, as well as other trials completed in South Africa and Kenya, have now shown the efficacy of MC for HIV prevention in varying age groups (18-24 and 15-49 years), and geographic settings (urban, periurban and rural populations).^1.a-c^ Thanks in large part to these data, the World Health Organization and United Nations AIDS (WHO/UNAIDS) recommended that MC be provided as part of a package for prevention of HIV acquisition in men.^1.d^

The original, NIH-funded RCT protocol was amended at the time of unblinding to provide the latest trial results to all trial participants and the Rakai community, and to offer circumcision surgery to the uncircumcised, control participants. That amended protocol is provided as the final Appendix, and will become inactive once a follow-up study protocol, associated informed consent documents and questionnaires receive Institutional Review Board (IRB) approval.

.

**1.2 FOLLOW-UP STUDY, POST TRIAL SURVEILLANCE, RAKAI (U01-AI075115-01A1)**

The Protocol Team submitted a grant application to the NIH to conduct a follow-up, post-RCT surveillance study of the men enrolled in the NIH trial. As a result of a successful peer review of the -01A1 application, the Principal Investigator’s Grantee Institution received notice of grant award for funding the study, which is entitled: “HIV/STIs and Behaviors in a RCT and Post-trial Surveillance, Rakai”**.** This protocol document will detail the recruitment of all eligible participants from the original NIH RCT for continued, long-term follow-up, including 1) the provision for circumcision surgery to uncircumcised, control participants from the original RCT who request the procedure; 2) the conduct of continued analyses to demonstrate the efficacy of circumcision to prevent sexually transmitted infections (STI’s) in HIV-negative trial participants; 3) the conduct of long-term, post-trial surveillance in order to assess the effectiveness of circumcision, to monitor sexual risk behaviors, and to estimate the number of surgeries required to avert one HIV infection over five years.

**2.0 SPECIFIC AIMS**

**2.1 AIM 1 - THE EFFICACY OF MALE CIRCUMCISION FOR STI PREVENTION DURING TRIAL FOLLOW-UP**

To assess the efficacy of circumcision for male STI prevention, we will conduct STI assays on archived specimens (serum, penile swabs and urine) provided by the 5,000 men enrolled in the original NIH RCT. Samples were obtained at enrollment/baseline visit, as well as at 6, 12, and 24 months thereafter. The retention rate was 92% at 24 months.

**The endpoints and assays included:**

1) *Syphilis*. Syphilis serology (RPR/TPPHA), was conducted in real time during the trial. These data are available and unblinded analyses have been conducted.

2) *HSV-2* will be detected by Kalon Biological HSV-2 IgG indirect ELISA. We will identify men who were HSV-2 seronegative at time of enrollment and determine the incidence of HSV-2 over 3 follow up visits by assaying the last available serum sample to detect HSV-2 seroconversions. For HSV-2 seroconverters, we will assay all available samples to determine the timing of infection.

3) *Penile HPV:* We will select a random sample of 50% of trial participants and assess their HPV status at each visit. HPV will be detected using Hybrid Capture 2 (hc2) assays to identify infection with one or more of 13 high risk HPV types (HR-HPV) responsible for cervical and penile cancers. All hc2 positive samples and 10% of HPV-negative samples will be assayed with PGMY09/11 consensus-primer PCR and reverse hybridization (HPV Linear Array, Roche Molecular Systems, Pleasanton, CA) to detect type-specific infections. We will determine point prevalence of HPV at baseline and each follow up, as well as incident HR-HPV infections and clearance of HR-HPV infections, both for the 13 oncogenic types detected by Hc2, and for each specific type detected by PCR. The type-specific HPV assays will conducted in collaboration with NCI.

Statistical analyses will estimate adjusted relative risks or relative hazards of STIs among circumcised intervention arm participants compared with uncircumcised controls, using multivariate log-binomial, Poisson and Cox proportional hazard modeling. We will also determine whether the effects of circumcision on HIV incidence may be mediated by STI cofactors, using stratified models and interaction terms.

**2.2 AIM 2 - LONGER-TERM, POST-TRIAL FOLLOW-UP**

***2.2.1 Completion of circumcision surgeries for trial participants***

Circumcision will continue to be offered to all previous control arm assignment participants, and to intervention arm crossovers (i.e., men randomized to receive immediate circumcision, but who failed to come for surgery within six months of enrollment, and who subsequently request circumcision as a service.)

***2.2.2 To assess the effectiveness of circumcision for HIV and STI prevention, and the effects of circumcision on sexual risk behaviors:***

At current rates of RCT follow-up, we expect to have retained ~ 4,600 men by July 2007, at which time funding support for the NIH RCT will end, and the post-trial surveillance study of the current protocol will begin. Remaining funds from original NIH RCT will be used to continue surveillance until activation of the post-trial surveillance study. The Protocol Team estimates that we will be able to enroll ~85% of these men into long-term follow up, the first visit of which will occur ~12 months after their final RCT visit. We will conduct annual follow up of trial participants for up to 5 years between July, 2007 and ~ Feb, 2012, in order to evaluate long-term HIV incidence, ulcerative STI rates (HSV-2, syphilis and multiplex PCR of current GUD), and risk behaviors (The last 4 months of the grant will be devoted exclusively to data analyses and dissemination.) . The STIs testing under this proposal focuses on HSV-2 and syphilis serology, and multiplex PCR (M-PCR) of genital ulcers, since GUD is a cofactor for HIV acquisition and transmission.^14,38,39^ (*H ducreyi* is uncommon but will be assessed by M-PCR of GUD). We will measure incident HIV, HSV-2, syphilis, GUD and cause-specific GUD etiology. Self-reported risk behaviors will be assessed by interview

We will determine HIV/STI incidence and risk behaviors in circumcised male trial participants compared to uncircumcised male trial participants (i.e., intervention arm crossovers (n=148) and control arm men who decline the procedure at the end of the trial, n ~230), as well as to uncircumcised male non-trial participants enrolled in the Rakai Community Cohort Study (RCCS) who decline the procedure offered as a service during post-trial follow up funded from other sources).

We will compare HIV/STI incidence and prevalence, and sexual risk behaviors among men circumcised as part of the trial (both intervention and control participants), with rates among uncircumcised trial participants who decline surgery, and uncircumcised male non-trial participants enrolled in the cohort. The rate ratio or hazards ratios will be modeled as described in Aim 1. These analyses will assess longer-term circumcision effectiveness with respect to HIV/STI infection, and determine whether circumcision is associated with behavioral disinhibition.

***2.2.3. Long-term qualitative studies of risk behaviors***

We will conduct qualitative research (focus groups and in-depth interviews) among men enrolled in the NIH-funded trial, male community members who did not enroll in the trial and female community members. We will assess attitudes and beliefs regarding circumcision, including perceptions of its potential effects on HIV prevention; perceived barriers to and facilitating factors for circumcision acceptance; and perceptions of surgical risks, HIV and STI risks, personal hygiene, possible impact on sexual satisfaction, and the role of risk reduction behaviors. We will also examine attitudes towards neonatal and child circumcision. Qualitative research will be conducted throughout the study period to monitor possible changes in attitudes which may occur over time, especially if circumcision services become widely available.

***2.2.4. Estimating the number of circumcisions per HIV infection averted and the cost per infection averted.***

The number of HIV infections averted per circumcision will be estimated from the differentials in HIV incidence between circumcised and uncircumcised men during the ~ 5year post-trial follow up. The cost per procedure in the Rakai Program is currently $69.00, including surgery and postoperative monitoring. This price is likely to decline as circumcision is offered as a service, with monitoring based on clinical indications rather than on the fixed follow up schedule stipulated in the trial protocol (i.e., 24-48 hours, 5-9 days and 4-6 weeks post-op until wound healing is certified.) We will estimate the cost per case of HIV infection averted during the post-trial follow up, and using an existing stochastic model based on empirical Rakai data,^40^ we will project the number of HIV infections and costs per infection averted over a longer period of time (e.g., 10-20 years), assuming any protective effects observed during the post-trial follow up will be sustained into the future.

**3.0 HYPOTHESES:**

**3.1 EFFICACY OF CIRCUMCISION FOR STI PREVENTION (BASED ON ASSAYS OF SAMPLES COLLECTED DURING THE TRIAL)**

Intervention arm men circumcised in the NIH-funded trial will have reduced rates of syphilis, HSV-2, penile HPV and trichomonas infections over 24 months, compared to uncircumcised, control-arm men.

**3.2 LONG-TERM POST-TRIAL EFFECTIVENESS OF MALE CIRCUMCISION FOR PREVENTION OF HIV AND STIS, AND THE EFFECTS OF CIRCUMCISION ON SEXUAL RISK BEHAVIORS**

1. Men circumcised in the NIH-funded trial (i.e., both men in the intervention arm, and those control arm men who accept circumcision after completion of 24 month’s follow up), will have lower HIV incidence than uncircumcised trial participants (i.e., intervention arm crossovers and controls who decline surgery after 24 months follow up), and uncircumcised non-trial participants enrolled in the Rakai cohort. This protective effect will be observed over the proposed 4.5 years post-trial follow up.
2. Circumcised trial participants will have lower rates of syphilis, HSV-2 and GUD (which are cofactors for HIV acquisition^14,38,39^), when compared to trial participants who decline the procedure, and uncircumcised non-trial participants in the Rakai cohort.
3. Circumcised trial participants will not increase their sexual risk behaviors (i.e., disinhibition or risk compensation), compared with uncircumcised men.
4. Male circumcision will result in a net reduction in community HIV incidence, both due to direct effects of circumcision on HIV acquisition in men, and indirect effects of reduced incidence in women.

*Please note, these hypotheses for the post-trial follow up do not address the possible biological efficacy of circumcision for HIV and STI prevention per se, since post-trial subjects are not randomized and will be self-selected. Rather, we will test the hypothesis that any biological efficacy of circumcision (established by the trial) will not be offset by behavioral disinhibition, such that there will be a sustained long-term* ***effectiveness*** *of circumcision for HIV/STI prevention during post-trial surveillance*

**4.0 STUDY DESIGN**

**4.1 AIM 1 - THE EFFICACY OF MALE CIRCUMCISION FOR STI PREVENTION DURING TRIAL FOLLOW-UP**

- - 1. ***Completion of laboratory STI assays on stored samples and analyses to estimate the efficacy of circumcision for male STI prevention***

Penile swabs, serum (from whole blood), and urine samples were collected at each visit during the NIH RCT (baseline visit, then 6, 12 and 24 months thereafter), and will continue to be collected in this follow-up study. If a participant experienced STI symptoms between scheduled visits in the RCT, he could receive syndromic management at government or private clinics, but we could not obtain samples for laboratory testing. Nevertheless, we can estimate the point prevalence of symptomatic and asymptomatic STIs at the time of each scheduled visit.

The field collection procedures for the RCT were as follows (and will continue to be followed in this study): At time of specimen collection, samples are logged in and immediately placed on ice in cold boxes. Samples are transported to the central laboratory within 4-6 hours of collection. On accession in the lab, serum and urine specimens are divided into aliquots and archived at -70^o^C. Swabs are frozen at -70^o^C. Samples remain frozen until assay, and the aliquots to be used for STI detection have not been thawed since original accession. Duplicate aliquots are shipped to JHU on wet or dry ice, or liquid nitrogen, and are archived at -70^o^C. Foreskins have been collected and stored in SafeFix preservative or ethanol. During the trial we conducted HIV and syphilis testing at each visit. We thus propose to assay stored samples for HSV-2 and HPV.

Given low gonorrhea and chlamydia prevalence among men in Rakai (1.0% and 2.7%, respectively),^122^ and the fact that observational data suggest little or no protective effects of circumcision with respect to urethral gonorrhea or chlamydia, will not assay for these pathogens or trichomonas.

***4.1.2 Sampling strategy for STI detection***

We will determine STI infections present at the time of scheduled visits. Men who experience episodes of symptomatic STIs between visits seek care for syndromic management from government and private clinics, and no specimens are available for laboratory testing. The sampling strategy for specimens collected during RCT varied with each STI and was designed to minimize costs.

1. ***Syphilis:***

Syphilis serology was conducted at baseline and at each follow up visit, and all seropositive cases were treated with IM Benzathine penicillin or azithromycin. The testing allows estimation of the prevalence of syphilis at all time points, as well as syphilis incidence during each follow up interval in circumcised versus control men. An unblinded analysis did not show any impact of circumcision on the prevalence of serologic syphilis.

***2) HSV-2:***

Since HSV-2 seropositivity is persistent, we will screen all 5000 enrollment samples to determine baseline HSV-2 prevalence in the intervention and control arms. Among men found to be HSV-2 negative at enrollment, we will assay the last available sample to detect seroconversions during follow up. For men whose last sample indicates seroconversion, we will test intervening follow up samples, starting from the next to last available specimen, in order to determine the timing of seroconversion.

***3) HPV*:**

Most HPV infections last < 2 years, and since there are multiple oncogenic or high risk HPV types (HR-HPV),^117a^ as well as types 6 and 11 associated with genital warts, re-infection with the same or different HPV types is possible. Thus, it will be necessary to test enrollment samples and each follow up sample to determine HPV prevalence, incidence and clearance. To reduce costs, a random sample of 50% of men from each arm will be selected for penile HPV assessment.

Incidence rates of any of 13 oncogenic HR-HPV types (detected by hc2) or the type-specific HPV infection (detected by PCR) will be calculated from the number of cases in which any HR-HPV or a given type is detected for the first time in men who tested negative at baseline or at a preceding follow up visit. Person-time will be calculated from the baseline or a prior negative test until the time of detection. HPV clearance will be defined as the presence of a negative test occurring after previously positive HPV test(s). Several definitions of clearance will be considered to decrease misclassification due to test sensitivity.^125^ These will include time to first negative test for a specific HPV type, and time to two and three consecutive negative tests for a specific HPV type.

***4) Genital* ulcer disease:**

At baseline and at each follow up, trial participants were asked about symptomatic GUD within the past year or present at time of interview. At trial enrollment, the period prevalence of GUD was 7.1% and the point prevalence of clinically confirmed current GUD was 0.9%. We collected multiplex PCR swabs from all genital ulcers observed clinically at baseline and each follow-up visit. We will conduct multiplex PCR assays on these swabs to assess the efficacy of male circumcision for prevention of GUD due to HSV-2, syphilis and *H ducreyi*. The prevalence rate ratio of GUD duriing follow up was 0.44 (p < 0.001) in the trial indicating a 56% efficacy of circumcision for prevention of symptomatic GUD.

***4.1.3 STI symptoms***

At each study visit, men are asked whether they had symptoms suggestive of STIs (GUD, urethral discharge and dysuria) at time of interview or during the preceding follow up interval. Anyone reporting symptoms was asked about the number and timing of episodes, medical care received, and whether their partners were informed and treated. At each visit, men underwent clinical examination of the penis and genitals, to minimize the underreporting of current symptomatology.

**4.2 AIM 2 - LONGER-TERM POST-TRIAL FOLLOW-UP**

**4.2.1 LONG-TERM INCIDENCE OF HIV, STIS AND RISK BEHAVIORS AFTER COMPLETION OF THE TRIAL**

***Follow-up study inclusion & Provision of circumcision to uncircumcised, control RCT participants.***

All participants in the NIH-funded RCT and the Rakai community were informed of the trial findings, per previous protocol. These same participants will be invited to participate in this post-trial surveillance study. Circumcision surgery will be offered to trial participants requesting the procedure.

Control participants will be contacted in the chronological order of their trial enrollment, so that controls who enrolled earlier and completed 24 months follow up will be given initial priority, and after completion of surgery for these men, priority will then be accorded to men by date of enrollment. Controls who present at the Kalisizo facility for unscheduled visits and who request surgery will also be provided with circumcision at time of presentation. Incident cases in the control arm who have completed 24 months follow up will be offered surgery. Men initially randomized to the intervention arm who failed to return for surgery within six months of enrollment and were classified as crossovers, will also be offered surgery. All participants and the community at large will be informed of the trial results and of the availability of surgery for trial participants who remain uncircumcised.

**4.3 STUDY PROCEDURES**

Pre-operative history and examination, and sample collection for storage and future testing

- All participants who have not received tetanus toxoid will be offered immunization.
- The genitalia will be inspected

The following additional samples will be collected: urine and swabs from under the foreskin for future testing, such as gonorrhea/chlamydia/HSV-2 and HPV, with separate funding. During surgery a filter paper blood spot will be collected for archival purposes.

- If there is clinical evidence of current infection (e.g., discharge, GUD), that might increase the risk of surgery, circumcision will be deferred and the patient will be treated and given penile hygiene instructions.
- If there are anatomic abnormalities (e.g., hypospadias, severe phimosis, urinary retention), the patient will be referred for evaluation to the consultant urologist, who will visit the clinic approximately at 2-4 week intervals to review these cases.
- For men who live a long distance from the Kalisizo clinic, a pre-operative overnight stay will be made available, if they choose to use this service.

***Surgical Procedure***:

Sleeve circumcision will be performed as follows:

- The penis and both surfaces of the foreskin are preped.
- Local anaesthesia (a mixture of Lignocaine and Marcaine) will be used to block the dorsal penile nerves at the base of the penis and to infiltrate the frenulum.
- With the foreskin in place and the skin lying undistorted on the shaft, a line is drawn following the outlines of the corona and the V of the frenulum. The foreskin is then retracted and a line marked 0.5 - 1 centimeter proximal to the corona, straight across the base of the frenulum.
- With the foreskin retracted the first incision follows the line marked on the inner surface, and bleeding is controlled by bipolar cautery. The incision is deepened to mobilize the skin edge. The prepuce is then extended over the glans and the marked proximal incision is made on the external surface.
- The distal skin edge of the foreskin will be retracted using skin hooks and/or artery or Allis clips, or sutures to permit dissection to free the dartos fascia from the skin.
- The sleeve of prepuce is then removed and will be retained for pathophysiologic studies.
- The foreskin will be laid out flat and measurements taken of the margins for estimates of surface area. The foreskin will then be cut into four sections placed in formalin, absolute alcohol or other appropriate fixative for archiving in Uganda and the US for histopathology and immunohistochemistry.
- All bleeders are controlled,
- Skin edges re-approximated with 4-O or 5-O absorbable sutures. The first stitch is a simple suture at the apex of the frenulum followed by a horizontal mattress suture at the base of the frenulum, and then three vertical mattress sutures of each of the remaining three points of the quadrants, and a minimum of two simple sutures between each principle mattress suture (a total of 13 or more stitches) .
- The wound is cleaned with normal saline
- Medicated, non-adhesive gauze is applied and knotted on the four principle sutures, another free medicated, non-adhesive gauze is wrapped around the first, and a regular gauze dressing applied, followed by an elastic bandage, held in place with strapping
- The procedure usually takes 45-60 minutes.
- The patients will recuperate for approximately 2-4 hours in a post-operative recovery room or until they are ready to go home.
- The surgical procedure and postoperative recovery will be recorded on Form 04.
- Men will be given well fitting underwear for comfort and to prevent wound disruption, if needed.

Other methods of circumcision such as forceps or dorsal slit may be used

***Postoperative care***

- Analgesics will be provided (predominantly acetaminophen)
- Men will not be discharged if they experience severe pain, cannot walk independently or have evidence of bleeding through the dressing
- Men will be instructed not to share their pain medications. The postoperative visit will be used to monitor analgesia use.
- Men will rest for a minimum of 30 minutes or longer until they feel able to return home. All men will be examined by a physician or health worker prior to discharge and a Form 04 completed.
- Men will be provided with instructions on wound care and analgesia, and this will be reviewed with them before discharge (Postoperative patient Information Sheet).
- At time of discharge, men will be offered free transport. Men who decline or do not require transport (residence close to the Rakai Health Sciences Program clinic) will be advised to be accompanied by a family member, friend or Rakai Health Sciences Program staff.
- Free postoperative overnight stay facilities will be made available, if men choose to avail themselves of this service.

***Asepsis***

- All theater staff will be trained in aseptic procedures, including documentation.
- Steam Indicators, Biological Indicators, autoclave tracking labels and instrument lists will be used to insure sterility, tracing of instruments and QC.

***Emergency procedures***:

- A crash cart, IV and oxygen will be available for emergencies.
- The main potential emergency is excessive bleeding, which will be controlled by ligation of vessels and by pressure. If uncontrolled bleeding persists, a pressure dressing will be applied, and a catheter wrapped around the penile shaft as a tourniquet. If these measures do not control bleeding, an IV will be inserted and oxygen provided if needed, and the patient will be transported to hospital (Kalisizo Hospital < 5 minutes away; larger hospitals in Masaka (Masaka Hospital or Kitovu, a mission hospital ~30 minutes) away, if needed.
- Anaphylactic reaction to the local anaesthetic will be managed with epinephrine, oxygen, IV and transport to the Hospital, if needed.

**Concomitant Medications**

***Analgesia***

- Experience has shown that postoperative Tramadol is not needed for pain control due to the long action of the local anaesthesia. Also, Tramadol can delay discharge due to drowsiness Therefore postoperative and subsequent analgesia will use Acetominophen 325 to 650 mg, every 4-6 hours, as needed.

Postoperative analgesia with Tramadol hydrochloride 50-100 mg stat will be provided if Acetominophen does not control postoperative pain.

Men will be evaluated within 48 hours, and if moderate to severe pain is reported, Tramadol hydrochloride 50 - 100mg- will be dispensed.

###### *Treatment of Infections*

1. Treatment of STDs and Balanitis.

- We will use symptom-based management of symptomatic infections at time of survey. In addition, syphilis treatment will be provided on the basis of serology.
- The regimen of treatment will, where possible, use directly observed, single oral therapies developed and evaluated in the STD Control Trial. The treatment algorithms include the following:

1) *GUD*. Therapy will cover *T. pallidum, H. ducreyi* and HSV-2: azithromycin 1 gram, ciprofloxacine 500 mg stat, IM benzathine benzylpenicillin 2.4 million units if there is serologic evidence of syphilis, and acycolvir 400mg t.d.s for five days for active herpes (per PDR 2003) ,other therapy may be provided as medically indicated

2) Urethral discharge/dysuria in men. Therapy will cover *N. gonorrohoeae*, and *C. trachomatis*: azithromycin 1 gram, ciprofloxacine 500 mg.

3) *Balanitis* treatment will consist of metrondizaole 2 grams stat for anaerobes, and topical clotrimazole for *C. albicans*.

2. Pre- or post-operative penile infections (other than balanitis) will be treated with azithromycin and ciprofloxacin. Indications for treatment are: a) clinical evidence of infection among men presenting for circumcision or after circumcision, and b) symptoms suggestive of current STDs (GUD, discharge, dysuria), reported by men during surveys or at post-circumcision visits, in both arms. Uganda MoH standard of care will be used for partner notification (MoH-STD Form 4) – Form 35 and provision of free treatment.

###### Treatment of Anemia

Men with hemoglobin < 8 grams/dL, will receive iron/folate supplements and presumptive treatment for malaria and helminths using standard MoH regimens for treatment of anemia in adults. Enrollment and randomization will be deferred until the hemoglobin is increased to  8 grams/dL. If the patient does not respond to therapy, they will be referred for investigation of persistent anemia. Anemia resulting from hemoglobinopathies such as sickle cell disease will be referred.

**4.4 SERIOUS ADVERSE EVENTS**

**Serious Adverse Event Reporting**

The occurrence of severe adverse events (SAEs) is recorded at every post-randomization visit.

An AE is defined, as “any untoward medical occurrence in a participant and that does not necessarily have a causal relationship with the treatment. An AE can be any unfavorable and unintended sign (including an abnormal laboratory finding), symptom or disease temporarily associated with the beginning of the treatment (i.e., following randomization).**”** Adverse events during screening and prior to randomization that result from study participation were also reported. The adverse events are defined in Table 3.

- The severity of AEs is characterized as:
- *mild* (transient and not affecting activity),
- *moderate* (requiring modification of activity or additional medical/minor surgical/intervention),
- *severe* (resulting in: death, life threatening condition, incapacitating symptoms which require hospitalization, significant medical/surgical interventions or which result in persistent significant disability/incapacity).
- All severe occurrences are considered serious adverse events (SAEs) for reporting purposes, consistent with FDA definitions as follows:
- Life threatening: substantial risk of dying at the time of the AE
- Hospitalization (initial or prolonged): if admission to hospital or prolongation of hospital stay results from the AE.
- Disability: if the AE resulted in a significant, persistent or permanent change, impairment, damage or disruption in the patient’s body function/structure, physical activities or quality of life.
- Intervention required to prevent permanent impairment or damage: if it is suspected that the treatment may result in a condition which required medical or surgical intervention to preclude permanent impairment or damage to the patient.

**Adverse events related to circumcision**

The relationship of AEs to study participation, particularly surgery or medications will in most cases be clear. We use the following classification.

- *definitely related* to study procedures such as surgery or anesthesia/analgesia (occurred during surgery or postoperatively),
- *probably related* (occurred during or after study procedures such as surgery, most likely to be due to the study procedure but could arise from other causes,
- *possibly related* (occurred during participation, but could equally or more plausibly be ascribed to other causes), and
- *unrelated* (an event clearly explained by causes other than the trial participation
- The timing of AEs is divided into screening/enrolment/randomization, operative, postoperative and during longer-term follow up.

The anticipated adverse events are largely those associated with surgery or study medications. The description of anticipated potential AEs is given in Table 1. All medical officers will be trained in the recognition and classification of the adverse events, and in the procedures required for reporting. The senior Medical Officers, will be responsible for AE reports.

Any other serious events not defined in Table 1 will be reported as SAEs.

**Table 1.** Study Definition of Adverse Events

| Adverse Event Type | Description of Adverse Event Type | Severity  and Codes |
| --- | --- | --- |

|  |  |  |
| --- | --- | --- |

| A. During Surgery |  |  |
| --- | --- | --- |

| Pain | Minor, not requiring additional anaesthesia | Mild (APS1) |
| --- | --- | --- |

|  | Moderate/severe controlled with additional anaesthesia* | Moderate (APS2) |
| --- | --- | --- |

|  | Severe, not controlled by additional anaesthesia | Severe (APS3) |
| --- | --- | --- |

| Excessive bleeding | More bleeding than usual, but easily controlled | Mild (ABL1) |
| --- | --- | --- |

|  | Bleeding that requires pressure dressing to control | Moderate (ABL2) |
| --- | --- | --- |

|  | Blood transfusion or transfer to another facility for management required | Severe (ABL3) |
| --- | --- | --- |

| Anesthetic-related event | Palpitations, vaso-vagal reaction or emesis | Mild (AAN1) |
| --- | --- | --- |

|  | Reaction to anesthetic requiring medical treatment in study clinic but not transfer to another facility | Moderate (AAN2) |
| --- | --- | --- |

|  | Anaphylaxis or any reaction requiring transfer to another facility | Severe (AAN3) |
| --- | --- | --- |

| Excessive skin removed | Adds time or material needs to the procedure, but does not result in any discernable adverse condition | Mild (AES1) |
| --- | --- | --- |
|  | Skin is tight, but additional operative work not necessary | Moderate (AES2) |

|  | Requires re-operation or transfer to another facility to correct the problem | Severe (AES3) |
| --- | --- | --- |

| Damage to the penis | Mild bruising or abrasion, not requiring treatment | Mild (ADP1) |
| --- | --- | --- |

|  | Bruise or abrasion to the glans or shaft of the penis requiring pressure dressing or additional surgery to control | Moderate (ADP2) |
| --- | --- | --- |

|  | Portion or all of the glans or shaft of the penis severed or burned by electrocautery | Severe (ADP3) |
| --- | --- | --- |

| B. First Month Post-Surgery |  |  |
| --- | --- | --- |

| Pain | Symptoms of pain requiring bed rest for less than half the day | Mild (BPA1) |
| --- | --- | --- |

|  | Pain requiring bed rest for more than half day | Moderate (BPA2) |
| --- | --- | --- |

|  | Excruciating pain requiring total bed rest | Severe |
| --- | --- | --- |

| Excessive bleeding | Dressing or other materials (for example underwear) spotted but dry | Mild (BBA1) |
| --- | --- | --- |

|  | Dressing or other materials wet with clotted blood requiring change of dressing | Moderate (BBA2) |
| --- | --- | --- |

|  | Dressing or other materials soaked with blood with obvious active bleeding requiring surgical exploration or transfusion | Severe (BBA3) |
| --- | --- | --- |

| Excessive skin removed | Client concerned but no discomfort on erection | Mild (BES1) |
| --- | --- | --- |

|  | Causes slight disconfort on erection but surgical correction not necessary. | Moderate (BES2) |
| --- | --- | --- |

|  | Interferes with life and surgical correction necessary | Severe (BES3) |
| --- | --- | --- |

| Insufficient skin removed | Prepuce partially covers the glans only when extended | Mild (BIS1) |
| --- | --- | --- |

|  | Prepuce still partially covers the glans and re-operation is required to correct | Moderate (BIS2) |
| --- | --- | --- |

|  | Not applicable |  |
| --- | --- | --- |

|  |  |  |
| --- | --- | --- |

| Swelling/Hematoma | More swelling than usual, but no treatment needed | Mild (BSH1) |
| --- | --- | --- |

|  | swelling requiring surgical exploration but no evidence of active bleeding | Moderate (BSH2) |
| --- | --- | --- |

|  | Rapidly expanding Heamatoma suggesting active bleeding requiring surgical exploration or referral | Severe (BSH3) |
| --- | --- | --- |

| Damage to the penis | Mild bruising or abrasion, not requiring treatment | Mild (BDP1) |
| --- | --- | --- |

|  | Bruise or abrasion to the glans or shaft of the penis requiring pressure dressing or additional surgery to control | Moderate (BDP2) |
| --- | --- | --- |

|  | Portion or all of the glans or shaft of the penis severed | Severe (BDP3) |
| --- | --- | --- |

| Infection | Pain and erythema with no obvious swelling | Mild (BIN1) |
| --- | --- | --- |

|  | Painful swelling with erythema or elevated temperature or purulent wound discharge | Moderate (BIN2) |
| --- | --- | --- |

|  | Cellulitis or wound necrosis | Severe (BIN3) |
| --- | --- | --- |

| Delayed wound healing | Healing takes longer than usual, but no extra treatment necessary | Mild (BDW1) |
| --- | --- | --- |

|  | Additional non-operative treatment required | Moderate (BDW2) |
| --- | --- | --- |

|  | Requires re-operation to correct | Severe (BDW3) |
| --- | --- | --- |

| Appearance | Client concerned, but no discernable deformity | Mild (BAP1) |
| --- | --- | --- |

|  | Minimal deformity does not require re-operation | Moderate (BAP2) |
| --- | --- | --- |

|  | Significant deformity requires re-operation to correct | Severe (BAP3) |
| --- | --- | --- |

| Problems with voiding | Transient complaint that resolves without treatment | Mild (BVO1) |
| --- | --- | --- |

|  | Requires a special return to the clinic, but no additional treatment required | Moderate (BVO2) |
| --- | --- | --- |

|  | Requires referral to another facility for management | Severe (BVO3) |
| --- | --- | --- |

| Wound dehiscence | Wound disruption involving no more than one principal suture. | Mild (BWD1) |
| --- | --- | --- |
|  | Wound disruption and involving two or more principal sutures. No surgical intervention required. | Modarete (BWD2) |
|  | Significant wound disruption requiring surgical correction | Severe (BWD3) |
|  |  |  |

| **C. One Month or More Post-Surgery** |  |  |
| --- | --- | --- |

| Infection** | Pain and erythema no obvious swelling | Mild (CIN1) |
| --- | --- | --- |

|  | Painful swelling/purulent wound discharge | Moderate (CIN2) |
| --- | --- | --- |

|  | Cellulitis or wound necrosis | Severe (CIN3) |
| --- | --- | --- |

| Delayed wound healing | Healing takes longer than usual, but no extra treatment necessary | Mild (CDW1) |
| --- | --- | --- |

|  | Additional non-operative treatment required | Moderate (CDW2) |
| --- | --- | --- |

|  | Requires re-operation to correct | Severe(CDW3) |
| --- | --- | --- |

| Appearance | Client concerned, but no discernable deformity | Mild (CAP1) |
| --- | --- | --- |

|  | Significant scarring or other cosmetic problem, but does not require re-operation | Moderate (CAP2) |
| --- | --- | --- |

|  | Requires re-operation to correct | Severe (CAP3) |
| --- | --- | --- |

| Excessive skin removed | Client concerned, but there is no deformity on erection | Mild (CES1) |
| --- | --- | --- |

|  | Causes slight discomfort on erection but surgical correction not necessary | Moderate (CES2) |
| --- | --- | --- |

|  | Interferes with sexual life and surgical correction is necessary | Severe (CES3) |
| --- | --- | --- |

| Insufficient skin removed | Prepuce partially covers the glans only when extended | Mild (CIS1) |
| --- | --- | --- |

|  | Prepuce still partially covers the glans and re-operation is required to correct | Moderate (CIS2) |
| --- | --- | --- |

|  | Not applicable |  |
| --- | --- | --- |

|  |  |  |
| --- | --- | --- |

|  |  |  |
| --- | --- | --- |

| Torsion of penis | Torsion is observable, but does not cause pain or discomfort. | Mild (CTP1) |
| --- | --- | --- |

|  | Causes mild pain or discomfort on erection, but additional operative work not necessary | Moderate (CTP2) |
| --- | --- | --- |

|  | Requires re-operation or transfer to another facility to correct the problem | Severe (CTP3) |
| --- | --- | --- |

|  |  |  |
| --- | --- | --- |

| Erectile dysfunction | Client reports occasional inability to have an erection | Mild (CED1) |
| --- | --- | --- |

|  | Client reports frequent inability to have an erection | Moderate (CED2) |
| --- | --- | --- |

|  | Client reports complete or near complete inability to have erections | Severe (CED3) |
| --- | --- | --- |

| Psycho-behavioral problems | Client reports mild sexual dissatisfaction attributed to circumcision, but no significant psycho-behavioural consequences | Mild (CPB1) |
| --- | --- | --- |

|  | Client reports significant sexual dissatisfaction attributed to circumcision, but no significant psycho-behavioural consequences | Moderate (CPB2) |
| --- | --- | --- |

|  | Significant depression or other psychological problems attributed by the participant to the circumcision | Severe (CPB3) |
| --- | --- | --- |
| Other AES | Other AEs are described below*** |  |
| **Additional Codes following AE review in February, 2006** | |  |
|  | Joint dehiscence (BWD) and infection (BIN) AEs, indicated by BWD/BIN. Degree of severity is per individual event and the first event to occur is reported first. | BWD/BIN |
|  | Stitch sinus: Sinus with non-puss discharge | SS1 |
|  | Stitch sinus with purulent discharge | SS2 |
|  | Stitch granuloma: Small, no need for surgical removal | SG 1 |
|  | Stitch granuloma: Moderate, requiring surgical intervention | SG 2 |
|  | Anatomic site of lesion at frenulum | F |
|  | Pain on intercourse no interference with sex | P1 |
|  | Pain on intercourse which reduces sexual frequency | P 2 |
|  | Pain on intercourse that prevents sex | P 3 |
|  | Pain or dehiscence due to intercourse, conjoint code | I |
|  | Wound dehiscence due to external trauma, conjoint code | EX |
|  | Allergic reaction to iodine skin prep: Mild blisters, resolved | APR 1 |
|  | Allergic reaction to iodine skin prep: Severe blisters requiring treatment | APR 2 |

- Additional anesthesia implies a volume of local anesthetic in excess of initial amount drawn up in the syringe and calculated to be sufficient for patient’s weight before surgery.

** Infection is indicated by swelling, erythema, pain, elevated temperature (locally or systemically, or purulent discharge)

All other SAEs resulting in hospitalization or death are reported to DAIDS and the IRBs of record in real time (i.e., within 10 days of PI notification of the event).

Management of AEs

- Free medical care is provided for AEs directly related to participation including surgical care if needed. If referral for medical or surgical complications is required, participants are referred to the Kalisizo Hospital (2-3 minutes driving time) from the surgical facility the Rakai Hospital or hospitals in the neighboring District of Masaka (approximately 30 minutes driving time) from the surgical facility**.**

Reporting of AEs

- All AEs will be ascertained and documented by Project Medical Officers
- All deaths and grade 3 or higher SAEs are notified to the Ugandan and U.S. PIs within 48 hours of detection, by e-mail or fax.
- The U.S. PI then will notify the relevant IRBs (WIRB and Uganda Virus research Institute) and DAIDS Medical Officer in writing as soon as possible, and not later than 10 days after identification of the SAE. The nature, severity, timing and relationship of the AE to circumcision or study medications is specified by the medical officer. A summary of how the severity and relationship to study participation was determined will be provided by the PI, and clarifications made by phone with the medical officer as needed.

Tabulations of AEs

- The PI will submit aggregate reports of all AEs and tabulations to DAIDS. AEs are described by type, time since surgery or medications, severity and relationship to study procedures. Dates of AEs are also tabulated to assess temporal trends or patterns. AEs are tabulated by total number, and by number of AEs per individual for those participants experiencing more than one AE.

Source documentation

- Operative and postoperative AEs use Rakai Health Sciences Program documentation
- Medical visits to health centers or other facilities outside of the Rakai Health Sciences Program cannot be completely documented due to poor records. We will endeavor to obtain copies of hospital medical records, after obtaining permission from the index person. Rakai Health Sciences Program reports (such as questionnaires), are also used to provide information.
- To maximize documentation, Rakai Health Sciences Program offers free medical care or referral for injury or illnesses as a result of study participation. Phone numbers of medical officers are provided on the Postoperative patient Information Sheet and all consent forms, we provide transport if needed and reimburse participants for transport costs. In addition, we arrange community health workers to provide a contact person in each village who can facilitate contact with the Project. Records are maintained for unscheduled visits (Form 16), and an AE report completed if indicated.

**Aim 2.2 Post-trial surveillance**

We will conduct annual surveillance of consenting trial participants for up to 5 years after the termination of the trial, until Dec 2011. (Additional limited follow-up is likely to extend to approximately March, 2012. in order to “mop-up” men who miss their last scheduled contact.) At each visit we will conduct an interview to determine sociodemographic status (e.g., change in marital status, education etc.), sexual behaviors and networks (number of marital and non-marital partners, partner characteristics, frequency of intercourse, condom use, consumption of alcohol associated with sex, etc.), health status including STD symptoms, attitudes towards circumcision (including perceptions of the level of risk reduction it confers) and the degree of satisfaction with the procedure. We will request a 15 mL venous blood sample for serology (HIV, syphilis, HSV-2) which are ulcerative conditions and cofactors for HIV acquisition. We will also collect and archive a penile swab from the coronal sulcus for HPV detection for future testing under separate funding. All trial participants will be actively followed up irrespective of whether they were circumcised during the trial (intervention arm) or after completion of 24 months’ follow up or study termination in the control arm. This will allow us to determine possible selective compliance with the protocol among intervention arm men who declined circumcision during the trial (5.9%), or control arm men who decline circumcision after completion of their follow up or trial closure.

###### Outcome measures

We will first assess the characteristics and behaviors to determine whether there is evidence of self-selection at time of enrollment into the post-trial effectiveness study (the characteristics of trial participants and non-participants are described below in Statistical Analysis Aim 2). The incidence of HIV and syphilis, HSV-2 and GUD, and rates of sexual risk behaviors will be compared between men circumcised during the trial and uncircumcised men, to determine the effectiveness of male circumcision for HIV/STI prevention and to assess possible behavioral disinhibition associated with circumcision. The comparison will be based on infection rates and sexual risk behaviors assessed at each visit, as well as within-individual changes in behaviors between visits. Men who accept post-trial circumcision will provide additional information on possible self-selection and risk behaviors.

###### HIV and STI sample collection and assays for the post-trial follow up

We will collect serum and penile swabs annually. HIV and syphilis testing will be conducted using the laboratory assays described below. Men who are HSV-2 seronegative at enrolment into the post-trial follow up will have their last available serological sample assayed in Year 5 of the proposed post-trial follow up. If they have seroconverted to HSV-2, intervening samples will be tested to determine the timing of seroconversion. Samples for HPV testing will be archived, but testing of these samples collected during the post-trial surveillance is not budgeted in this proposal

###### Long-term qualitative studies of risk behaviors

We plan to conduct the qualitative research in each year of the study.

##### Focus Group Discussions:

We will conduct ~40 focus groups a year in project Years 1-5. Focus groups for men and women will be stratified by trial participation/non-participation by men and their partners, post-trial acceptance or refusal of circumcision services by non-trial participants and their female partners, age (e.g., 15-19, 20-29, 30-49) and marital status (unmarried/married). Focus group participants will be selected at random from cohort/trial participants. We have a full-time Department of Qualitative Research which has extensive experience with qualitative research under the direction of Ms. Wagman who worked in Rakai for six years. Focus group guides will be prepared, and focus group moderators and note takers will be trained in their use, including role playing and mock discussion sessions. The domains to be specifically explored include perceptions, facilitators and barriers to circumcision, acceptability of the procedure, and the relationship of circumcision to sexuality, religion, and risk for HIV/STDs. We will also assess attitudes towards neonatal circumcision, since in the long term, this is likely to be programmatically the safest and most cost-effective approach to providing circumcision. Changes in attitudes and perceptions over time will also be probed. This information will assist in the development of education messages on the benefits and risks of circumcision, and will facilitate interpretation of quantitative information from the structured interviews. Each focus group will last approximately 1.5 to 2 hours. Light refreshments will be provided. The goal of these group discussions is not to learn about individual behavior, but rather to learn about shared attitudes and expectations regarding circumcision.^127,128^

##### In depth interviews:

We will conduct 40 in-depth interviews per year with knowledgeable key informants. Informants will be purposefully sampled (i.e., criterion-based selection, which represents a commonly used approach in such research) ^127^ to ensure that they are representative of their group (e.g., by age, marital status, trial participation) and to broaden the number of response categories. Criteria for informant selection will also include their enculturation and involvement in their respective communities. Using structured guidelines, open-ended questions will be asked to encourage conversation and enable probing, to elicit information on experiences and on behavioral disinhibition. Interviewers will be trained using role playing and other methods. The key informants will provide insights into the experiences, attitudes, perceptions and opinions of individuals.

Focus groups and in depth interviews, will be conducted in Lugandan, and (contingent on participants’ consent) will be tape recorded for transcription and translation into English. All qualitative research personnel are fluent in English and Lugandan and have extensive experience with transcription and translation. In addition to the tape recordings, focus group leaders and interviewers will write a summary of their notes and observations immediately following each conversation; which will be included in the body of ethnographic data. The qualitative analysis plan is described below.

**5.0 LABORATORY METHODS FOR STI DETECTION**.

**HIV:** We use two separate EIAs (Vironostika HIV-1, Organon Teknika, Charlotte, NC and Cambridge Biotech, Worcester, MA). EIA discordant samples are confirmed by Western blot (HIV-1 Western Blot, Bio-Merieux-Vitek, St. Louis, MO). RT-RNA PCR (Roche Amplicor 1.5) is used for HIV viral load.

**Syphilis:** We use RPR for screening and TPPA/TPHA for confirmation of all RPR positives.

**HSV-2:**  The samples will be tested using HSV-2 IgG ELISA (Kalon Biological Aldershot, UK), with a cut off optical density (OD) ≥ 1.5 OD, provides a sensitivity of 93% and specificity of 94.0%. Dr. Rhoda Ashley Morrow (University of Washington) has shown that serological samples stored at -70^o^C are stable for 10 or more years for HSV-2 assay.

**HPV detection:** Penile swabs from the shaft and the coronal sulcus were stored separately in STM media (Digene) and PCR buffer at -80^o^C in Uganda, and transported on dry ice for archiving at Johns Hopkins. Work by Dr. Giuliano (pc) has shown that the prevalence of HPV detection was 34.9% in circumcised men, compared to 40.9% in uncircumcised men with samples from the coronal sulcus, whereas there were no effects of circumcision on specimens collected from the shaft (prevalence 50.0% and 49.3%, in circumcised and uncircumcised men, respectively). Therefore, we will only assay swabs from the coronal sulcus. We will screen coronal sulcus specimens stored in Digene STM media with Hybrid Capture 2 (hc2, Digene Corporation, Gaithersburg, MD) to detect any of 13 high risk HPV (HR-HPV) types responsible for cervical and penile cancers.^117a^ All hc2-positive specimens and 100-200 hc2-negative specimens will then be tested by PCR/reverse blot hybridization for genotyping discrimination. Only a fraction of the original sample will be denatured and processed to preserve high quality nucleic acid for PCR testing. We will use PCR for amplification of a fragment of the L1 gene as previously described.^126^ Genomic DNA will be extracted using the QIAamp Mini kit (QIAGen,). DNA will be eluted in100μl Nuclease-free water, quantified by fluorescence using the Picogreen assay (Molecular Probes) and stored at –4ºC until further analysis by PCR amplification and HPV genotyping. Specimens will be tested for the presence of HPV by amplifying 50µl of the DNA extracts with the PGMY09/11 L1 consensus primer system^126^ and Ampli*Taq* Gold polymerase (Perkin-Elmer, Foster City, CA.), following the conditions of the manufacturer (Roche Diagnostics, Indianapolis, IN). For every 20 samples a negative and a positive control will be run. In addition, one randomly re-sampled clinical sample will be included for every batch of 20 samples as blinded quality controls. Dr. Gravitt has found that the DNA assays are stable up to 13 years if samples are stored at -70^o^C. HPV genotyping will be conducted using the reverse line blot method (Roche Diagnostics). The HPV genotype strip contains 39 probe lines, detecting 37 individual HPV genotypes and two concentrations of the ß-globin control probe. (types: 6, 11, 16, 18, 26, 31, 33, 35, 39, 40, 42, 45, 51 to 56, 58, 59, 61, 62, 64, 66 to 73, 81, 82, 83, 84, IS39, and CP6108.) The PCR products labeled with biotin will be denatured and added to the probe strip in a hybridization buffer. After strips are washed, a streptavidin-horseradish peroxidase conjugate will be added to facilitate detection. After final wash, buffer will be removed by vacuum aspiration, and strips rinsed in 0.1M sodium citrate. Color development will be activated by incubation in a mixture of hydrogen peroxide in sodium citrate buffer and tetramethylbenzidine in dimethylformamide for 5 minutes on a rotating platform (70 rpm). Developed strips will be interpreted and photographed for future reference. Strip interpretation will be performed with a labeled overlay, with lines indicating the position of each probe relative to the reference mark. QA/QC will be conducted quarterly in Dr. Giuliano’s laboratory. Concordance of less than 85% between laboratories will trigger a re-evaluation..The assays will be performed in Dr. Patti Gravitt’s laboratory at JHU under an existing NCI contract.

**GUD:** Men with a current GUD were asked to provide an ulcer swab which was stored in PCR buffer for multiplex PCR testing to detect HSV-2, syphilis and *H. ducreyi.*^114^

**6.0 STUDY POPULATIONS, SAMPLE SIZES AND POWER CALCULATIONS**

The study populations are summarized in Figure 1. Only those populations of men enrolled in the NIH trial of male circumcision for HIV prevention (indicated in yellow highlight) are covered under this protocol. Comparative populations covered from other protocols are listed in order to explicate estimates of power and analysis plans.

**AIM 1. Study populations, sample size and power calculations for estimating the efficacy of male circumcision for STI prevention on samples collected during the NIH-funded RCT**

We estimated the sample sizes required for each STI endpoint, assuming a power (1-β = 0.80) and two-tailed α = 0.05. Where possible, we used prior Rakai data to estimate the prevalence/incidence of specific STIs, and to determine the likely efficacy of circumcision for reduction of STI rates. Where Rakai data were unavailable, we used published and unpublished findings for prevalence and efficacy estimates (e.g., HPV). The trial has a fixed sample size and person-time available, therefore we have estimated the detectable relative risk in this population for each of the STI outcomes.

###### Estimates of number of participants and person time available during the trial

For the whole trial population of 5000 men, retention rates were ~90% at each scheduled follow up visit (6, 12 and 24 months**.** By December 12, 2006, we had accrued a total of 6,744 person years in both arms combined (3,352 in the intervention arm and 3,392 in the control), which is 73% of the anticipated total information.

***Power calculations for assessment of the efficacy of circumcision for STI prevention (Aim 1).***

Power calculations used the Poisson approximation,^131^ with uniform allocation (λ = 1). For incident events power = 1-β = 1-Φ (A), where A is the proportion of the area of a distribution that is to the left of a point A. A = Z_α_ – [P_c_ – P_t_] / √( P_c_ + P_t_ ) / n_c_. For prevalent events we used the Chi-square approximation where A = Z_α_ √ 2PQ/ n_c_ - P_c_ – P_t_/ √ (P_c_Q_c_ + P_t_Q_t_)/ n_c_

***Syphilis:*** The prevalence of serologic syphilis is 10%, and incidence is ~1.5/100 py. All samples have been tested, and the study has the power to detect a RR ≤0.80 in syphilis prevalence and a RR ≤ 0.50 for incident syphilis in circumcised versus uncircumcised men.

***HSV-2 sample size and power estimates:*** All 5,000 enrollment samples will be screened for HSV-2 baseline seropositivity. From age-specific Rakai HSV-2 prevalence rates, applied to the age structure of the trial population, we expect ~50% of men to be HSV-2 positive at enrollment, leaving 2,500 initially HSV-2 negative men for detection of HSV-2 incidence. We will assay the last available sample for these 2,500 men to detect seroconversions during the RCT. We estimate the annual HSV-2 incidence will be ~ 6/100 py in the trial population. We have accumulated ~1,539 py per arm (based on 1,250 initially HSV-2 negative men per arm, ~90% retention and 72% information), and the study has the power to detect a RR of ≤ 0.67 in intervention relative to control arm men for incident HSV-2. This is within the range of risks reported in several observational studies.^62^

***HPV sample size and power estimates:*** To reduce costs, we propose to conduct assays on a 50% random sample of trial participants at enrollment and to test these men at each follow up visit (i.e., 1,250 subjects per arm for a total of ~1,539 py per arm over two year’s follow up, with 90% retention and 72% total information). HPV point prevalence will be determined at enrollment and each follow up. We do not know the prevalence of penile HPV in Rakai, but in the IARC study, HPV prevalence was 19.6% in uncircumcised men compared to 5.5% in circumcised men.^66^ The Rakai trial has the power to detect ≤ 0.84 reduction of HPV point prevalence at follow up. The incidence of HPV in Rakai men is also unknown. However, female HR-HPV incidence was 7.0/100 py in HIV-negative women,^118^ and if we assume that male incidence is ~0.66 that of female incidence (i.e., ~4.6/100 py), we have the power to detect a rate ratio ≤0.66 in all incident HR-HPV among circumcised versus uncircumcised men. The rate ratios of HPV associated with circumcision reported in observational studies range from 0.36-0.40,^66,67^ thus the study has the power to detect effects of this magnitude. From Rakai data, the type-specific incidence of HPV 16 and 18 which confer the highest neoplastic risk is ~1.2/100 py,^118^ and we have the power to detect a RR ≤ 0.24 for this endpoint. HPV clearance rates in infected men are unknown, but in HIV-negative, HPV infected Rakai women, 37% cleared the infection per year.^118^ Assuming a 19.6% prevalence of HPV in uncircumcised men and a clearance rate of 37% per year, the study has the power to detect a RR ≥1.24 of increased HPV clearance rates associated with circumcision.

**Etiology of Genital ulcer disease*:*** The point prevalence of current GUD is ~0.9%, and we anticipate diagnosis of ~ 128 current GUD episodes at study visits, so the study has the power to detect a RR ≤0.54 for this endpoint. In Rakai, 43% of GUD are due to HSV-2 (using multiplex PCR), and we have the power to detect a RR ≤ 0.35 for a reduction in HSV-2 ulcers in circumcised men. Ulcers due to syphilis or *H ducreyi* are too infrequent to be separately assessed.

**Table 2.** STI outcomes, testing strategies, number of participants and detectable relative risks to assess circumcision efficacy for STI prevention in trial participants (conducted on samples collected in the trial).

| Outcome of interest, expected rates | Testing strategy, ~ # of men both arms combined | Detectable RR |
| --- | --- | --- |
| Syphilis prevalence (10%) | All samples tested during the trial | ≤ 0.80 |
| Syphilis incidence ~1.5/100 py | All samples tested during the trial (~3,200 py/arm) | ≤ 0.50 |
| HSV-2 baseline prevalence (~50%) | Enrollment, baseline comparability (n = 5000) | Na |
| HSV-2 incidence (~6/100 py) in HSV-2-neg men | Test last available follow up samples (~1539/arm), and test preceding samples for seroconverters | ≤ 0.67 |
| HPV prevalence at enrollment ~19.6% | 50% for baseline comparability, (n = 1250/arm) | Na |
| HPV point prevalence during follow up ~ 19.6% | 50% random sample of participants, retested at each visit (n ~ 2557 visits/ arm) | ≤0.84 |
| HPV incidence ~ 4.6/100 py | 50% random sample (~1539 py/ arm) | ≤0.66 |
| Type-specific HR-HPV incidence (~1.2/100 py) | 50% random sample (~1539 py/ arm) | ≤ 0.24 |
| HPV clearance ~37% in HPV positives | Test follow up samples of HPV positive individuals n ~501/arm) | ≥ 1.24 |
| Current GUD, ~ 0.9% | Clinical exam of current GUD | ≤0.54 |
| GUD due to HSV-2 (43% of GUD) | Test GUD by multiplex PCR to detect HSV-2 | ≤ 0.4 |

**AIM 2. Study populations, sample size and power estimates for long-term follow up of trial participants and non-participants**

We first estimate the populations and person years of observation among trial participants and initially uncircumcised non-trial participants. We then estimate the detectable relative risks for key study outcomes with 1-β of 80% and two-tailed α of 0.05. We used the Poisson approximation for incident outcomes and Ch-square approximation for prevalent outcomes, with non-uniform allocation λ≠ 1.^131^

###### Men circumcised in the trial participant population

1,000 trial participants were enrolled between August, 2003 and June, 2004; the remainder (n = 4,000) were enrolled between July 2004 and July 2005. We conducted long term follow up of men enrolled between Aug 2003 and June 2004 using funds which remain after the early termination of the trial.

The trial retention rate is ~ 90% over two years. Thus, at the end of the trial, we expect that ~4,050 out of the initial 5000 men enrolled will be under observation at time of trial closure. Assuming ~85% annual retention rate rates during post-trial follow up, we would expect 3,441 in Year 1, 2,927 py in year 2, 2,486 py in year 3, and 2,113 py in year 4 of post-trial surveillance. There will be an additional 6 months follow up available between year 4 and 4.5, providing ~898 py. Thus, we expect a cumulative total of 11,865 py observation during the post-trial period. However, 148 intervention arm men were crossovers (5.9%), so we discounted their person-time (~439 py). In addition, allowance must be made for an unknown number of control arm men who may decline circumcision after completion of follow up. On current data we would expect ~90% of controls to accept circumcision, so we have discounted person-time further by adjusting for the 10% of controls who may decline circumcision (n~230, ~674 py), yielding a total of ~10,752 py observation in circumcised men over 5 years post-trial follow up.

This protocol only covers men enrolled into the trial of male circumcision for HIV prevention. However, we describe comparative populations of uncircumcised men needed for data analysis. The follow up of the latter men are covered under separate protocols. It is likely that non-trial participants will request surgery after completion of the RCT. However, we do not know how many will do so, therefore, we assumed a range of acceptance from 40-60% (i.e., n~639-959), and thus ~639-959 will decline this post-trial service. These men will provide ~ 1,874 to 2,810 py in an uncircumcised state during post-trial follow up, depending on the proportions accepting surgery and assuming an 85% annual retention rate, as observed in the cohort. In addition, uncircumcised person-time will be accrued from intervention arm crossovers (n=150 with ~430 py) and controls who refuse surgery (n~230 with ~674 py), total ~1104py. The total person-time for uncircumcised men is likely to range from 2,978 to 3,914 py. If we assume circumcision acceptance rates of 40-60% (i.e., n~639-959), and further assume a median time from enrollment to surgery of ~12 months (to allow time for men to decide whether they want the procedure, and time required to complete the circumcisions), then these non-trial participants who become circumcised during the long-term follow up could contribute ~ 1,330-1,996 py (discounting a year delay before surgery and assuming 85% annual retention). We have used these estimates for power calculations for selected endpoints.

***Study power to detect differences in HIV and selected STI incidence and sexual risk behaviors between circumcised trial participants and uncircumcised non-participants.***

We used data from the Rakai cohort to estimate the prevalence or incidence of HSV-2 and syphilis. Rates of HIV, GUD and sexual risk behaviors (e.g., non-marital partners and consistent, inconsistent or no condom use), were derived from the trial results. These estimates were used to determine the detectable relative risks among circumcised trial participants compared with uncircumcised men during the 4.5 year post-trial follow up, assuming ranges of circumcision non-acceptance of 40-60%. Power estimates accounted for the inequality in the number of circumcised trial participants versus uncircumcised men. (Table 2.)

If circumcision reduces the rate of HIV, then during the 5 year surveillance we will be able to detect significant reductions in HIV risk (RR ≤0.53-0.57) with adequate power to detect the risks observed in the three completed trials which range from 0.4-0.52.^18,27^ Please note, HIV incidence in the control arm of the trial was 1.3/100 py, which is lower than the incidence in our previous observational study (1.8/100 py). This probably reflects self-selection of the trial population (see below) which would likely be less in the post-trial surveillance. Thus, our estimates of power for detecting HIV incidence effects (i.e., effectiveness) are very conservative. The reviewers asked whether a lower retention rate might adversely affect study power. If we assume a 75% annual retention and circumcision acceptance of 60%, the study still has the power to detect a significant RR ≤ 0.55 for HIV incidence, which is adequate to detect the RRs in the clinical trial and observational studies.

We have power to detect effects on HSV-2 incidence (RR≤ 0.77-0.84). The power to detect effects on incident syphilis is more constrained (RR ≤0.55-0.60). However, adding the person-time contributed by non-trial participants who opt for circumcision, we will be able to detect a RR ≤ 0.73 for HSV-2 incidence, and ≤ 0.62 for incident syphilis. If incident HSV-2 and syphilis are combined as a single ulcerative STI endpoint, we will have the power to detect RRs compatible with those reported from observational studies.^62^ The study has the power to detect effects on self-reported GUD (RR ≤0.79-0.81), which is well within the range of our trial results (IRR = 0.44) and published data,^24^ We also will have the power to detect even modest increases in sexual risk behaviors (non-marital partners and consistent condom use) among circumcised participants versus uncircumcised men (range of RRs ≥1.09-1.13).

**Table 3.** Detectable relative risks of HIV/STI incidence and sexual risk behavior outcomes during 5 years post-trial follow up in circumcised trial participants relative to uncircumcised men.

| Number and person years, outcomes and expected frequencies in uncircumcised non-trial participants who decline circumcision | Detectable relative risks  (circumcised trial participants, 10,752 py) | | |
| --- | --- | --- | --- |
|  | Uncircumcised men if circ acceptance is 40% in non-trial participants | Uncircumcised men if circ acceptance is 50% in non-trial participants | Uncircumcised men if circ acceptance is 60% in non-trial participant* |
| Numbers and person years | n~1339, py~3923* | n~1179, py~3455* | n~1019, py~2986* |
| HIV incidence (1.3/100 py) | ≤0.57 | ≤0.55 | ≤0.53 |
| HSV-2 incidence (6.0/100 py)** | ≤0.84 | ≤0.79 | ≤0.77 |
| Syphilis incidence (1.5/100 py) | ≤0.60 | ≤0.58 | ≤0.55 |
| GUD in past year (7.1%) | ≤0.81 | ≤0.80 | ≤0.79 |
| Non-marital sex partners (30%) | ≤1.08 | ≤1.09 | ≤1.09 |
| Consistent condom use (18%) | ≤1.10 | ≤1.12 | ≤1.13 |

**Includes intervention arm participants who crossover and controls who refuse circumcision*

**For herpes, it was assumed that 50% are HSV-2 positive at baseline and incidence estimates are based on the initially HSV-2 seronegative populations and person time.

###### Comparability of populations in the post-trial follow up

Self-selection into trial participation or non-participation is an important issue for the statistical analysis. Therefore, we compared the baseline characteristics of trial participants to male cohort members who were eligible for enrollment but did not participate in the circumcision trial and are used as comparative populations for the post-trial surveillance. These non-trial participants are covered by separate protocols.

The age distributions differed between trial participants and non-trial participants (e.g., ages 15-19 27.5% vs 21.2%; 20-29 yrs 45.5% vs 38.7%, 30-39 yrs 20.9% vs 23.3%, and age ≥40 5.7% vs 16.8%, respectively). The two groups differed with respect to marital status (47.0% vs 56.0% were currently married, 47.7% vs 38.5% had never married, and 5.4% vs 5.5% were previously married among trial and non-trial participants, respectively). Education was differed somewhat among trial participants and non-participants (no education 5.7% vs 5.0%, primary 66.1% vs 60.8%, secondary 24.0% vs 23.9%, post-school education 4.3% vs 10.3%, respectively). No sexual activity in the past year was reported by 19.3% of trial participants and 17.9% of non-trial participants. There were modest differences in the number of non-marital partnerships; one non-marital partner was reported by 31.6% of trial participants and 26.6% of non-participants, and two or more non-marital partners were reported by 19.4% of trial subjects vs 35.3% of non-trial participants. However, consistent condom use was in trial participants 13.8% than non-participants 9.5%). Alcohol use associated with sex was reported by 38.0% of participants and 58.1% of non-trial participants. GUD in the past year was reported by 7.1% of trial participants and 8.2% of non-trial participants. The two groups were comparable with respect to symptoms of urethral discharge (3.5% vs 3.8%), and dysuria (7.0% vs 8.4%%, respectively).

In summary, the HIV risk profiles of the two groups (with the exception of age non-marital partners and alcohol use), appear reasonably comparable, and it should be possible to adjust for self-selection bias in the statistical analysis.

**7.0 STATISTICAL ANALYSIS**

**AIM 1. The efficacy of circumcision for prevention of STI infections in intervention and control arm trial participants.**

This will primarily be an intent-to-treat analysis, although per protocol analyses will also be conducted.

STI prevalence will be determined at time of trial enrollment and incidence rates per 100 person-years (py) will be estimated, assuming seroconversion occurred at the mid-point of each follow up interval. In both arms, time from enrollment will be accumulated up to 24 months of follow up. Person time will discount the postoperative period of sexual abstinence for intervention arm subjects. GUD will be assessed as the proportions reporting ulceration during the prior follow up interval, the number of episodes, and as the point prevalence of current GUD at each visit. Exploratory data analysis will examine the population characteristics and STI prevalence in the two arms at enrollment to assess baseline comparability.

Syphilis and HSV-2 incidence will be determined per 100 py in initially seronegative persons. The incidence of any of 13 oncogenic HR-HPV will be determined from the number of newly detected hc2 positive HR-HPV infections in men who were hc2 negative at baseline or at the preceding follow up visit. Since we will only genotype hc2 positive specimens, type specific HPV infections will be restricted to 13 HR-HPV genotypes. Type specific incidence will be based on a new infection with a given HPV type among men who tested negative for that specific HR-HPV type at baseline or at the preceding follow up visit. Cumulative incidence will be computed over 24 months of follow-up. Persistence will be calculated from the number of cases positive for any HR-HPV (detected by hc2) or the same HPV type at more than one consecutive visits among those who are ever positive (i.e., prevalent positive at baseline or incident positive at follow up). STIs will be assessed by strata of covariates such as age, marital status, behaviors (e.g., number of partners, condom use etc.), and other variables to identify potential confounders. We will also assess contamination (i.e., cross-over) among men allocated to circumcision who did not receive surgery (n = 146), and among control subjects who opted to have circumcisions performed from non-Project services (n = 32).

For prevalent infections at enrollment or the point prevalence of infections at follow up, we will use log-binomial regression methods to estimate the adjusted rate ratios of STI prevalence in circumcised versus uncircumcised men. The primary analytical model for incidence analyses will be Poisson regression to compare the relative incidence of syphilis, HSV-2 and HPV between the two study arms. For both log-binomial and Poisson regression models, adjustment will be made for individual-level variables found to differ substantially between groups at enrollment, and for potential confounding variables. Potential confounders identified from prior risk analyses will include age, risk behaviors (i.e., number and nature of partners, condom and alcohol use). Secondary analyses will include any additional potential confounders identified during exploratory data analysis and will include covariates associated with STI incidence (p < 0.15), or covariates with a RR > 2.0 or <0.5. Models will be fit for the whole population and for strata of particular interest (e.g., by age, sexual risk behaviors etc.). Robust standard errors will be estimated using GEE to account for lack of independence in repeated observations within individuals.^132^

###### The associations between STIs, GUD and HIV acquisition

We will also assess the associations between STIs and STD symptoms (primarily GUD), and incident HIV infection to determine whether the effects of circumcision on HIV incidence may be partly mediated by the effects of circumcision on cofactors for HIV acquisition. If, as expected from prior analyses, HSV-2 and GUD are the main cofactors associated with incident HIV,^38,85^ and are reduced by circumcision, as found in our trial, and in observational studies,^24,62-3^,we will assess how these co-infections modify the circumcision effects on HIV incidence using stratified models or via interaction terms.

**Analysis plan for the post-trial follow up to assess circumcision effectiveness and behavioral disinhibition.**

###### Descriptive characteristics of trial participants and non-participants

We will first assess the sociodemographic and behavioral characteristics at initiation of the post-trial follow up for the following groups: a) circumcised trial participants; b) uncircumcised trial participants, c) initially uncircumcised non-trial participants who subsequently accept surgery, or d) decline the procedure. This will evaluate possible self-selection, and any imbalances significant at p<0.15 will be included in multivariate analyses.

**Data Analysis plan**

For circumcision effectiveness we will compare HIV/STI and GUD incidence in circumcised trial participants with rates in uncircumcised men. Person time will be accrued from date of enrollment into the post-trial follow up until the occurrence of an incident event, loss to follow up or censoring by completion of observation. For non-trial participants who ultimately accept circumcision, person time will be accrued from date of surgery. HIV/STI incidence in circumcised and uncircumcised men will be determined at each study visit for assessment of differentials and of time trends. Cumulative incidence over ~ 5 years will also be estimated. For assessment of possible behavioral disinhibition associated with circumcision, we will compare self-reported rates of sexual risk behaviors (e.g., non-marital partners; consistent, inconsistent or no condom use; and alcohol use with sex) in circumcised and uncircumcised men. Behaviors will be evaluated at each study visit to determine differentials between circumcised and uncircumcised men, and to assess time trends In addition, within-individual changes of behaviors will be assessed.

The data and analytical plans will take the following forms:

***Person-time data:***  For HIV, syphilis and HSV-2 incidence we will estimate rates of events over person time. Each event is discrete and multivariate analyses will employ Poisson regression methods with person time as an offset. This is regarded as an exploratory analysis since the underlying point process is assumed to be stationary, but this assumption may not be satisfied.

***Repeated measurement data:*** Outcomes observed at sequential surveys (e.g., risk behaviors) will allow analysis of time trends over 4.5 years. Since individuals will have repeat measurements we will assess intra-individual change in risk behaviors and other time varying characteristics. Multivariate analyses of repeat measurement data will use population-average (marginal) and growth (conditional) models. The population-average model will explore the associations between risk factors (e.g., circumcision status, trial participation, multiple partners, condom, alcohol use, VCT acceptance and sociodemographics), and outcomes (e.g. HIV incidence). The dependence among repeated measurements within individuals is not of interest. Essentially this is a cross-sectional model, but has the additional feature that time-dependent covariates (e.g., time-dependent circumcision status or behaviors) can be incorporated. For continuous and binary outcomes, we will use GEE methods with robust variance.^132^ Statistical methods for categorical ordinal outcomes which generalize those of McCullagh^133^ have been developed by Stram *et a*l.^134^ We will use the generalized linear model ^133^ to explore the relationship between repeated outcome measurements and risk factors without restrictive parametric assumptions. In contrast, the “growth model” provides information about pattern-changes (e.g., changes in behaviors over time). Such models are important for prediction of time trends associated with individual changes. To account for the dependence of repeated measurements within individuals, we will use random-effects growth curve models for continuous response variable.^135-37^. Markovian and random-effects logistic regression models^138-9^ will be used for binary outcomes.

***Time-to-event data:*** HIV and STI incidence can be assessed by time-to-event methods. Univariate survival analysis techniques will be employed to compute (i) discrete Kaplan-Meier curves to estimate the incidence rate of events at discrete time points (e.g., HIV or STI incidence), (ii) log-rank test and weighted log-rank tests to detect overall incidence rate differences between groups, and (iii) the discrete Cox regression model to identify the relative risks for time-independent covariates (e.g., gender) or time-dependent covariates (e.g., changes of risk behaviors, etc.) We will check that the assumption of proportionality is met.

***Recurrent and multiple events*:** Potential repeated events (e.g., sexual risk behaviors), will use the following methods: (i) Pattern/trend analysis of recurrent and multiple event data. Subject to baseline risk factors, nonparametric and semiparametric techniques^140-43^ will be adopted to produce estimates of the rate and cumulative rate functions of recurrent events over time. (ii) Marginal models for recurrent^140^ and multiple events and for multiple event data^144,145^ will be used for exploratory analysis to identify risk factors. Marginal regression models will be designed to understand if risk factors are associated with the rate of recurrent events.^146-49^ In general, marginal models have population-average interpretations and therefore these models are especially appropriate for comparison of risk factors. (iii) Transitional/conditional regression analysis, in contrast with marginal models,^150-51^ can be adopted to analyze recurrent and multiple event data. The model will integrate an individual’s baseline information, time-dependent risk factors, and an individual’s recurrent event history into the covariates for predicting future events. The model interpretation is individual-based and therefore the analysis is viewed as a form of trajectory analysis.

***Dealing with self-selection bias and informative censoring*:**  Self-selection bias arises because the trial participants opted to enroll whereas non-trial participants declined enrollment. Also, among trial participants, there were crossovers (5.9% of intervention and 1% of control men); and control subjects may not accept circumcision after completion of 24 months follow up. In addition, self-selection may occur among non-trial participants who accept or decline circumcision services. For example, we would expect that older men may decline the procedure, but this unknown. The distribution of risk-behaviors and demographic variables are similar among intervention and control participants at enrollment, and as described previously, with the exception of age, there are relatively modest differences in risk characteristics between trial participants and non-participants enrolled in the Rakai cohort. We will use these risk-behavior and demographic variables in regression models to avoid or to minimize possible bias due to self-selection.

A complicating factor in analyzing longitudinal data is the presence of a non-ignorable censoring. When the study is extended to ~ 4.5 years post-trial follow up with an assumed 85% annual retention rate, the potential problem of informative censoring becomes more important because the ultimate loss-to-follow-up by the end-of-study is expected to be around 47.8% (i.e., ~15% annually). Losses to follow up, may be differential between trial participants and non-participants, or differential between non-trial participants accepting or declining post-trial circumcision. Mortality in HIV-negative men is low (~4/1000 py),^93^ and is unlikely to be differential between comparison groups. Also, men who seroconverted during the trial or post-trial follow up will have access to free ARV treatment (under our PEPFAR grant) and we expect few if any deaths during the 5 year surveillance grant. We will first evaluate the level of informative censoring and choose statistical models and techniques to handle informative censoring for both recurrent event data^140,148,142^ and for repeated measurement data.^153^

**Long-term qualitative studies of risk behaviors; analysis of qualitative data.**

Analysis will follow a grounded-theory approach whereby a coding scheme will be developed from interview transcripts based on major themes and categories. Data will be examined to assess patterns and relationships relevant to facilitators or barriers to circumcision, acceptability of the procedure, the perceived relationship of circumcision to sexuality, religion, and risk for HIV/STDs, and attitudes towards neonatal circumcision. As categories emerge, a coding system will be developed so that transcripts can be indexed and systematically analyzed. A consensus coding procedure will be used to foster reliability between coders. Two trained persons will independently code each interview or focus group transcript, compare the coding, discuss discrepancies and arrive at a common set of codes for transcripts.

Coding will be conducted at multiple conceptual levels with general themes and categories being derived from key words, statements, and concepts.^158^ Individual responses will be coded for each questions asked, these will be further coded for more specific, word-level replies. Sets of statement-level responses will be organized within question level categories, examined and semantically similar responses will be collapsed into a single code. Codes within each question category will then be re-examined to determine if they could be aggregated under a more comprehensive category or theme. If so, such a theme will be created as a code or subcategory within each question-level code. Later, cross-cutting themes and categories will be identified and collected into a general set of conceptual codes.

After field data collection, audiotapes from focus group discussions and interviews will be translated and transcribed from Luganda into English text, maintaining vernacular where appropriate. All written transcripts will be entered into MS word, reviewed and edited then reviewed by investigators to be identify main themes related to circumcision. Electronic transcripts will be imported into QSR NVivo for coding. QSR NVivo allows for data to be indexed by codes specific to each of the research questions guiding this study and also allows for work to be done in a combination of languages in the same project, thus Luganda and English data can be simultaneously analyzed. Retrieval and analysis. We will generate matrices of interconnections of the core topic areas to condense and organize the data and to simplify cross-informant/cross-group analysis. Specifically, systematic assessment of the relationships between perceptions of circumcision, potential benefits, and possible effects on sexual risk behaviors will be performed. Structured search commands will be used to identify patterns and relationships among constructs in interview and focus group data. Searches will retrieve all text with a set of two or more specific codes and identify relationships in the data. Search commands will be used to explore research questions in Aim 2. These search functions provide an efficient means of identifying and indexing statements of interest, themes and patterns, and the development of taxonomies that are representative of the ideas or experiences of a large proportion of participants/informants. The key themes and patterns that emerge in interviews and focus groups will be summarized.

###### Estimates of HIV infections averted and cost per infection averted by circumcision

Using the crude and adjusted RRs or hazard ratios from the effectiveness analysis described above, we will estimate the number of HIV infections averted from the difference in incident events between circumcised and uncircumcised men during the 4.5 year follow up. This will provide an estimate of the number of incident events averted by circumcision. The HIV infections averted by circumcision among women will be estimated from HIV incident events in female partners of circumcised and uncircumcised men. We will then estimate the number of circumcisions required to avert one HIV incident event in males, females and in the total population. Using a cost of $69.00 per surgery (or less, as we acquire data on costs associated with surgery provided as a service, rather than in a trial setting), we will then estimate the cost per HIV infection averted. Projections of the number of infections averted over 10-20 years will be based on our stochastic simulation model,^77^ using the effectiveness estimates derived from the 4.5 year follow up data. The costs per surgery will be inflated by 3% py to determine the long-term costs per infection averted. We will assess different levels of circumcision program coverage (0-100%), and will factor in neonatal circumcisions which are likely to increase in number if the trials prove efficacy.

**The population-level effects of circumcision on HIV incidence, and estimation of the population attributable fraction of HIV incidence attributable to circumcision**

HIV incidence in the Rakai cohort can be disaggregated by age, sex, male circumcision status, risk behaviors and coital frequency.^6,38,39,85a^, We will know the number of circumcised and uncircumcised men and their female partners in the cohort, and their rates of HIV acquisition. We will decompose population-level HIV incidence into events among circumcised and uncircumcised men and their partners, and estimate the crude population attributable fraction (PAF) of HIV transmission associated with circumcision versus not being circumcised, during each follow up year and in all years combined.^114,155,156^ In addition, we will have interview information on covariates that affect HIV exposure such as risk behaviors (non-marital partners, condom and alcohol use), acceptance of VCT (by individuals or couples), disclosure of HIV status to partners, concurrent ulcerative STIs, and we can identify HIV+ cohort participants who are or are not on ARVs. This information will be used to estimate adjusted rate ratios of incident HIV in circumcised relative to uncircumcised men, and adjusted PAFs attributable to circumcision in the population.^114,155-58^

8.0 TIME LINE – POST RCT SURVEILLANCE PROTOCOL

|  | Project Year | | | | |
| --- | --- | --- | --- | --- | --- |
| Activity | 1 | 2 | 3 | 4 | 5 |
| **Aim 1. Efficacy of circumcision for STI prevention** | | | | | |
| STI assays on trial participants | XXXX | XXXX |  |  |  |
| Analysis of STI outcomes |  |  | XXXX |  |  |
| **Aim 2. Long-term follow up of trial participants** | | | | | |
| Post-trial follow up | XXXX | XXXX | XXXX | XXXX | XX |
| Qualitative studies | XXXX | XXXX | XXXX | XXXX | XX |
| Final analyses of post-trial follow up and of the qualitative studies |  |  |  |  | XXXX |

**9.0 LITERATURE CITED**

1.a [Gray RH, Kigozi G, Serwadda D, Makumbi F, Watya S, Nalugoda F, Kiwanuka N, Moulton LH, Chaudhary MA, Chen MZ, Sewankambo NK, Wabwire-Mangen F, Bacon MC, Williams CF, Opendi P, Reynolds SJ, Laeyendecker O, Quinn TC, Wawer MJ.](http://www.ncbi.nlm.nih.gov/entrez/query.fcgi?db=pubmed&cmd=Retrieve&dopt=AbstractPlus&list_uids=17321311&query_hl=16&itool=pubmed_DocSum) Male circumcision for HIV prevention in men in Rakai, Uganda: a randomized trial, Lancet. 2007 Feb 24;369(9562):657-66.

1.b [Bailey RC, Moses S, Parker CB, Agot K, Maclean I, Krieger JN, Williams CF, Campbell RT, Ndinya-Achola JO.](http://www.ncbi.nlm.nih.gov/entrez/query.fcgi?db=pubmed&cmd=Retrieve&dopt=AbstractPlus&list_uids=17321310&query_hl=16&itool=pubmed_DocSum) Male circumcision for HIV prevention in young men in Kisumu, Kenya: a randomized controlled trial, Lancet. 2007 Feb 24;369(9562):643-56.

1.c. Auvert B, Taljaard D, Lagarde E, Sobngwi-Tambekou J, Sitta R, Puren A., Randomized, controlled intervention trial of male circumcision for reduction of HIV infection risk: the ANRS 1265 Trial PLoS Med. 2005 Nov;2(11):e298. Epub 2005 Oct 25. Erratum in: PLoS Med. 2006 May;3(5):e298.

1.d. WHO/UNAIDS. New data on male circumcision and HIV prevention: Policy and programme implications. WHO Technical Consultation, Montreuz, 6-8 March, 2007, Conclusions and recommendations. WHO, March 25, 2007

1. Bailey RC, Halperin DT. Male Circumcision and HIV Infection. Lancet 2000;355(9207):927.

2. Bongaarts J, Reining P, Way P, Conant F. The relationship between male circumcision and HIV infection in African populations. AIDS 1989;3(6):373-7.

3. Baeten JM, Richardson BA, Lavreys L, Rakwar JP, Mandaliya K, Bwayo JJ et al. Female-to-male infectivity of HIV-1 among circumcised and uncircumcised Kenyan men. J Infect.Dis. 2005;191(4):546-53.

4. Buve A. HIV/AIDS in Africa: Why So Severe, Why So Heterogeneous? Presented at the 7^th^ Conference on Retroviruses and Opportunistic Infections. Abstract S28. San Francisco, CA, USA: Study Group on Heterogeneity of HIV Epidemics in African Cities; 2000.

5. de Vincenzi I, Mertens T. Male circumcision: a role in HIV prevention? AIDS 1994;8(2):153-60.

6. Gray RH, Kiwanuka N, Quinn TC, Sewankambo NK, Serwadda D, Mangen FW et al. Male circumcision and HIV acquisition and transmission: cohort studies in Rakai, Uganda. Rakai Project Team. AIDS 2000;14(15):2371-81.

7. Gray RH, Wawer M, Serwadda D, Thoma M., Nalugoda F, Li X et al. Male Cirumcision and the Risks of Female HIV and Sexually Transmitted Infections Acquisition in Rakai, Uganda. Denver Colorado, USA: 13 th CROI Conference on Retroviruses and Opportunistic Infection; 2006.

8. Green EC. Male circumcision and HIV infection. Lancet 2000;355(9207):927.

9. Halperin DT, Bailey RC. Male circumcision and HIV infection: 10 years and counting. Lancet 2000;354(9192):1813-5.

10. Harrison DC. Male circumcision and HIV infection. Lancet 2000;355(9207):926.

11. Jessamine PG, Plummer FA, Ndinya Achola JO, Wainberg MA, Wamola I, D’Costa LJ et al. Human immunodeficiency virus, genital ulcers and the male foreskin: synergism in HIV-1 transmission. Scand.J Infect.Dis.Suppl 1990;69:181-6.

12. Lavreys L, Rakwar JP, Thompson ML, Jackson DJ, Mandaliya K, Chohan BH et al. Effect of circumcision on incidence of human immunodeficiency virus type 1 and other sexually transmitted diseases: a prospective cohort study of trucking company employees in Kenya. J Infect.Dis. 1999;180(2):330-6.

13. Moses S, Bradley JE, Nagelkerke NJ, Ronald AR, Ndinya-Achola JO, Plummer FA. Geographical patterns of male circumcision practices in Africa: association with HIV seroprevalence. Int.J Epidemiol. 1990;19(3):693-7.

14. Quinn TC, Wawer MJ, Sewankambo N, Serwadda D, Li C, Wabwire-Mangen F et al. Viral load and heterosexual transmission of human immunodeficiency virus type 1. Rakai Project Study Group. N.Engl.J Med. 2000;342(13):921-9.

15. Reynolds SJ, Shepherd ME, Risbud AR, Gangakhedkar RR, Brookmeyer RS, Divekar AD et al. Male circumcision and risk of HIV-1 and other sexually transmitted infections in India. Lancet 2004;363(9414):1039-40.

16. Sateren W.S., Bautista CT, Shaffer D.N., Foglia G, Wassuna M, Kiplangat S et al. Male circumcision and HIV infection risk among tea plantation residents in Kericho, Kenya: incidence results after 1.5 years of follow-up.. Toronto, Canada: XVI International AIDS Conference; 2006. Abstract no. TUAC0202

17. Seed J, Allen S, Mertens T, Hudes E, Serufilira A, Carael M et al. Male circumcision, sexually transmitted disease, and risk of HIV. J Acquir.Immune Defic.Syndr.Hum.Retrovirol. 1995;8(1):83-90.

18. Weiss HA, Quigley MA, Hayes RJ. Male circumcision and risk of HIV infection in sub-Saharan Africa: a systematic review and meta-analysis. AIDS 2000;14(15):2361-70.

19. Siegfried N, Muller M, Deeks J, Volmink J, Egger M, Low N et al. HIV and male circumcision—a systematic review with assessment of the quality of studies. Lancet Infect.Dis. 2005;5(3):165-73.

20. Urassa M, Todd J, Boerma JT, Hayes R, Isingo R. Male circumcision and susceptibility to HIV infection among men in Tanzania. AIDS 1997;11(3):73-80.

21. Patterson BK, Landay A, Siegel JN, Flener Z, Pessis D, Chaviano A et al. Susceptibility to human immunodeficiency virus-1 infection of human foreskin and cervical tissue grown in explant culture. Am.J Pathol. 2002;161(3):867-73.

22. Potts M. Male circumcision and HIV infection. Lancet 2000;355(9207):926-7.

23. Szabo R, Short RV. How does male circumcision protect against HIV infection? British Medical Journal 2000;320(7249):1592-4.

24. Thoma M., Gray RH. Male circumcision and GUD: A meta-analysis. AIDS, 2006. Submitted

25. McCoombe SG, Short RV. Potential HIV-1 target cells in the human penis. AIDS 2006;20(11):1491-5.

25a. O’Farrell, Morison L, Moodley P, Pilay K, Vanmali T, Quigley M, Hayes R, Sturm AW. Association between HIV and subpreputial penile wetness in uncircumcised men in South Africa. JAIDS 2006;43:69-77.

26. Gray PB. HIV and Islam: is HIV prevalence lower among Muslims? Soc.Sci.Med. 2004;58(9):1751-6.

27. Auvert B, Taljaard D, Lagarde E, Sobngwi-Tambekou J, Sitta R, Puren A. Randomized, controlled intervention trial of male circumcision for reduction of HIV infection risk: the ANRS 1265 Trial. PLoS.Med. 2005;2(11):e298.

28. Williams C. Clinical Trials to Assess the Efficacy of Male Circumcision to Reduce the risk of HIV acquisition. NIH Meeting, May 3, 2005.

29. Bailey RC, Moses S, Agot K., Paker C.B., Maclean I, Ndinya-Achola J. A randomized controlled trial of male circumcision to reduce HIV incidence in Kisumu, Kenya: progress to date.. Toronto, Canada: XVI International AIDS Conference.; 2006. Abstract no. TUAC0201

30. Quigley MA, Weiss HA, Hayes RJ. Male circumcision as a measure to control HIV infection and other sexually transmitted diseases. Curr.Opin.Infect.Dis. 2001;14(1):71-5.

30.a. SchaeferC, Hannemann D, Meister R. Post-marketing surveillance system for drugs in pregnancy- 15 years experience of ENTS. Reprod Toxicol 2005;20:331-43

30.b Davis AJ. A comparative analysis of drug safety withdrawals in the UK and US (1971-92): implications for current regulatory policy. Soc Sci Med, 2005;61:881-92

30.c. Zaheer K. <http://news.yahoo.com/s/nm/20061219/india>

31. Meeting on operational research for male circumcision UNAIDS and WHO, January 18, 2006.

32. Male circumcision: Current status and next steps. Gates Foundation. Washington DC, May 6, 2006.

33. Van Dam, Anastasi MC. Male Circumcision and HIV prevention. Pop Council .2000. 2006.

33a. Lie RK, Emanuel EJ, Grady C. Circumcision and HIV prevention research: an ethical analysis. Lancet 2006;368:522-5.

34. Rottingen JA, Cameron DW, Garnett GP. A systematic review of the edpidemiologic interactions between classic sexually transmitted diseases and HIV. STDs 2001;28:579-97

35. Crepaz N, Hart TA, Marks G. Highly active antiretroviral therapy and sexual risk behavior: a meta-analytic review. JAMA 2004;292(2):224-36.

35a. Richens J, Imrie J, Copas A. Condoms and seat belts: the parallels and the lessons. Lancet 2000;355(9201):400-3.

36. Gray RH, Li X, Wawer MJ, Gange SJ, Serwadda D, Sewankambo NK et al. Stochastic simulation of the impact of antiretroviral therapy and HIV vaccines on HIV transmission; Rakai, Uganda. AIDS 2003;17(13):1941-51.

37. Kahn J.G.. Cost-effectiveness of male circumcision in sub-Saharan Africa. Abstract no. TUAC0204. Toronto, Canada: XVI International AIDS Conference.; 2006.

38. Gray RH, Wawer MJ, Brookmeyer R, Sewankambo NK, Serwadda D, Wabwire-Mangen F et al. Probability of HIV-1 transmission per coital act in monogamous, heterosexual, HIV-1-discordant couples in Rakai, Uganda. Lancet 2001;357(9263):1149-53.

39. Wawer MJ, Gray RH, Sewankambo NK, Serwadda D, Li X, Laeyendecker O et al. Rates of HIV-1 transmission per coital act, by stage of HIV-1 infection, in Rakai, Uganda. J Infect.Dis. 2005;191(9):1403-9.

40. Gray RH, Li X, Kigozi G, Serwadda D, Nalugoda F, Watya S, Reynolds SJ, Wawer MJ. The impact of male circumcision on HIV incidence, and cost-per infection prevented: A stochastic simulation model from Rakai, Uganda *AIDS* 2007. In press

41. Mishra V. Patterns of HIV seroprevalence and associated risk factors. Durban, South Africa.: PEPFAR Annual Meeting, June; 2006.

42. Zoossmann-Diskin A. No protective effect of circumcision on human immunodeficiency virus incidence. J Infect.Dis. 2000;181(5):1865-8.

45. Kelly R, Kiwanuka N, Wawer MJ, Serwadda D, Sewankambo NK, Wabwire-Mangen F et al. Age of male circumcision and risk of prevalent HIV infection in rural Uganda. AIDS 1999;13(3):399-405.

47. Bailey RC, Neema S, Othieno R. Sexual behaviors and other HIV risk factors in circumcised and uncircumcised men in Uganda. J Acquir.Immune Defic.Syndr. 1999;22(3):294-301.

48. Mayatula V, Mavundla TR. A review on male circumcision procedures among South African blacks. Curationis. 1997;20(3):16-20.

49. Mogotlane SM, Ntlangulela JT, Ogunbanjo BG. Mortality and morbidity among traditionally circumcised Xhosa boys in the Eastern Cape Province, South Africa. Curationis. 2004;27(2):57-62.

50. Diseker RA, Lin LS, Kamb ML, Peterman TA, Kent C, Zenilman J et al. Fleeting foreskins: The misclassification of male circumcision status. Sexually Transmitted Diseases 2001;28(6):330-5.

51. Brown JE, Micheni KD, Grant EMJ, Mwenda JM, Muthiri FM, Grant AR. Varieties of male circumcision - A study from Kenya. Sexually Transmitted Diseases 2001;28(10):608-12.

52. Moses S, Bailey RC, Ronald AR. Male circumcision: assessment of health benefits and risks. Sex Transm.Infect. 1998;74(5):368-73.

54. Wawer MJ, Reynolds SJ, Serwadda D, Kigozi G, Kiwanuka N, Gray RH. Might male circumcision be more protective against HIV in the highly exposed? An immunological hypothesis. AIDS 2005;19(18):2181-2.

55. Fowke KR, Kaul R, Rosenthal KL, Oyugi J, Kimani J, Rutherford WJ et al. HIV-1-specific cellular immune responses among HIV-1-resistant sex workers. Immunology and Cell Biology 2000;78(6):586-95.

56. Kaul R, Rowland-Jones SL, Kimani J, Dong T, Yang HB, Kiama P et al. Late seroconversion in HIV-resistant Nairobi prostitutes despite pre-existing HIV-specific CD8(+) responses. Journal of Clinical Investigation 2001;107(3):341-9.

57. Devito C, Hinkula J, Kaul R, Lopalco L, Bwayo JJ, Plummer F et al. Mucosal and plasma IgA from HIV-exposed seronegative individuals neutralize a primary HIV-1 isolate. AIDS 2000;14(13):1917-20.

58. Trabattoni D, Lo Caputo S, Maffeis G, Vichi F, Biasin M, Pierotti P et al. Human alpha defensin in HIV-exposed but uninfected individuals. JAIDS 2004;35(5):455-63.

59. Montori VM, Devereaux PJ, Adhikari NKJ, Burns KEA, Eggert CH, Briel M et al. Randomized trials stopped early for benefit: A systematic review. JAMA 2005; 294 (17):2203-9.

60. Pocock SJ. When (not) to stop a clinical trial for benefit. JAMA 2005;294(17):2228-30.

61. Manual for Male Circumcision Under Local Anaesthesia. Draft. WHO, UNAIDS: June; 2006.

62. Weiss HA, Thomas SL, Munabi SK, Hayes RJ. Male circumcision and risk of syphilis, chancroid, and genital herpes: a systematic review and meta-analysis. Sex Transm.Infect. 2006;82(2):101-9.

63. Gray R, Azire J, Serwadda D, Kiwanuka N, Kigozi G, Kiddugavu M et al. Male circumcision and the risk of sexually transmitted infections and HIV in Rakai, Uganda. AIDS 2004;18(18):2428-30.

64. Diseker RA, III, Peterman TA, Kamb ML, Kent C, Zenilman JM, Douglas JM, Jr. et al. Circumcision and STD in the United States: cross sectional and cohort analyses. Sex Transm.Infect. 2000;76(6):474-9.

65. To T, Agha M, Dick PT, Feldman W. Cohort study on circumcision of newborn boys and subsequent risk of urinary-tract infection. Lancet 2000;352(9143):1813-6.

66. Castellsague X, Bosch FX, Munoz N, Meijer CJ, Shah KV, de Sanjose S et al. Male circumcision, penile human papillomavirus infection, and cervical cancer in female partners. N.Engl.J Med. 2002;346(15):1105-12.

66a. Gray RH. M Wawer, M Thoma, D Serwadda, F Nalugoda, G Kigozi, N Kiwanuka, N Sewankambo, O Laeyendecker, T Quinn. Male Circumcision and the Risks of Female HIV and STI Acquisition in Rakai, Uganda. Conference on Retroviruses and Opportunistic Infections, Boston, 2006.

67. Baldwin SB, Wallace DR, Papenfuss MR, Abrahamsen M, Vaught LC, Giuliano AR. Condom use and other factors affecting penile human papillomavirus detection in men attending a sexually transmitted disease clinic. Sex Transm.Dis. 2000;31(10):601-7.

68. Aynaud O, Ionesco M, Barrasso R. Penile intraepithelial neoplasia. Specific clinical features correlate with histologic and virologic findings. Cancer 1994;74(6):1762-7.

69. Dillner J, von Krogh G, Horenblas S, Meijer CJ. Etiology of squamous cell carcinoma of the penis. Scand.J Urol.Nephrol.Suppl 2000(205):189-93.

70. Maden C, Sherman KJ, Beckmann AM, Hislop TG, Teh CZ, Ashley RL et al. History of circumcision, medical conditions, and sexual activity and risk of penile cancer. J Natl Cancer Inst 1993;85(1):19-24.

71. Buonaguro FM, Tornesello ML, Salatiello I, Okong P, Buonaguro L, Beth-Giraldo E et al. The Uganda study on HPV variants and genital cancers. J Clin.Virol. 2000;19(1-2):31-41.

72. Hunter DJ, Maggwa BN, Mati JK, Tukei PM, Mbugua S. Sexual behavior, sexually transmitted diseases, male circumcision and risk of HIV infection among women in Nairobi, Kenya. AIDS 2000;8(1):93-9.

73. Fonck K, Kidula N, Kirui P, Ndinya-Achola J, Bwayo J, Claeys P et al. Pattern of sexually transmitted diseases and risk factors among women attending an STD referral clinic in Nairobi, Kenya. Sex Transm.Dis. 2000;27(7):417-23.

73.a. Turner AN et al. Men’s circumcision status and women’s risk of HIV acquisition in Zimbabwe and Uganda. Unpublished manuscript, UNC, 2006

74. Williams BG, Lloyd-Smith JO, Gouws E, Hankins C, Getz WM, Hargrove J et al. The Potential Impact of Male Circumcision on HIV in Sub-Saharan Africa. PLoS.Med. 2006;3(7):e262.

75. Mesesan K. The potential benefits of expanded male circumcision programs in Africa: predicting the population-level impact on heterosexual HIV transmission in Soweto. Abstract no. TUAC0203. Toronto, Canada: XVI International AIDS Conference, 2006.

76. Gray R. What will it take to control the epidemic? Reducing HIV transmission: Lessons from Rakai and other African Studies. Rio De Janero, Brazil: IAS; 2005.

77. Gray R. The potential impact of male circumcision on HIV incidence, HIV infections averted and cost-per infection prevented: A stochastic simulation model from Rakai, Uganda: RH. Gray, X Li, G Kigozi, D Serwadda, F Nalugoda, S Watya, SJ. Reynolds, M Wawer. Submitted to AIDS 2006.

78. Quinn TC. Circumcision and HIV Transmission: The Cutting Edge. Paper # 120. Denver, CO.: 13^th^ Conference on Retroviruses and Opportunistic Infections; 2006.

79. Nnko S, Washija R, Urassa M, Boerma JT. Dynamics of male circumcision practices in northwest Tanzania. Sexually Transmitted Diseases 2001;28(4):214-8.

80. Halperin DT, Fritz K, Mcfarland W, Woelk G. Acceptability of adult male circumcision for sexually transmitted disease and HIV prevention in Zimbabwe. Sexually Transmitted Diseases 2005;32(4):238-9.

81. Bailey RC, Muga R, Poulussen R, Abicht H. The acceptability of male circumcision to reduce HIV infections in Nyanza Province, Kenya. Aids Care-Psychological and Socio-Medical Aspects of AIDS/HIV 2002;14(1):27-40.

82. Pinkerton SD. Sexual risk compensation and HIV/STD transmission: empirical evidence and theoretical considerations. Risk Anal. 2001;21(4):727-36.

83. Cassell MM, Halperin DT, Shelton JD, Stanton D. Risk compensation: the Achilles’ heel of innovations in HIV prevention? BMJ 2006;332(7541):605-7.

84. Agot K., Kiarie J. NHOJOTWN. Male circumcision in Siaya and Bondo districts, Kenya: a prospective cohort study to assess behavioural disinhibition following circumcision.. Toronto, Canada: XVI International AIDS Conference; 2006. Abstract no. TUAC0205

85a. Wawer MJ, Sewankambo NK, Serwadda D, Quinn TC, Paxton LA, Kiwanuka N, Wabwire-Mangen F, Li C, Lutalo T, Nalugoda F, Gaydos CA, Moulton LH, Meehan M, Ahmed S, Rakai Study Group, and Gray RH. Control of sexually transmitted diseases for AIDS prevention in Uganda: a randomized community trail. Lancet 353:525-535,1999.

85.b. Gray RH, Wabwire-Mangen F, Kigozi G, Sewankambo NK, Serwadda D, Moulton L, Quinn TC, O=Brien KL, Meehan M, Abramowsky C, Robb M, and Wawer MJ. Randomized trial of presumptive sexually transmitted disease therapy during pregnancy in Rakai, Uganda. Am J Obstet Gynecol 185:1209-17, 2001.

85. Serwadda D, Gray RH, Sewankambo NK, Wabwire-Mangen F, Chen MZ, Quinn TC et al. Human immunodeficiency virus acquisition associated with genital ulcer disease and herpes simplex virus type 2 infection: a nested case-control study in Rakai, Uganda. J Infect.Dis. 2003;188(10):1492-7.

86. Gray RH, Li X, Wawer MJ, Serwadda D, Sewankambo NK, Wabwire-Mangen F et al. Determinants of HIV-1 load in subjects with early and later HIV infections, in a general-population cohort of Rakai, Uganda. J Infect.Dis. 2004;189(7):1209-15.

87. Laeyendecker O, Li X, Arroyo M, McCutchan F, Gray R, Wawer M et al. The Effect of HIV Subtype on Rapid Disease Progression in Rakai, Uganda. Boston Massachusetts, USA: 13^th^ CROI Conference on Retrovirsues and Opportunistic Infection; 2006.

87a. Arroyo MA, Sateren WB, Serwadda D,Gray RH, Wawer MJ, Sewankambo NK, Kiwanuka N, Kigozi G, Wabwire-Mangen F, Eller M, Eller LA, Birx DL, Robb ML, and McCutchan FE. Higher HIV-1 incidence and genetic complexity along main roads
in Rakai District, Uganda. Submitted to JAIDS, 2006.

88. Gray RH, Wawer MJ, Serwadda D, Sewankambo N, Li C, Wabwire-Mangen F et al. Population-based study of fertility in women with HIV-1 infection in Uganda. Lancet 1998;351(9096):98-103.

89. Nguyen RHN, Gange SJ, Quinn TC, Serwadda D, Wabwire-Mangen F, Wawer M, Gray RH.  Association between HIV viral load and reduced fertility among women in Rakai District, Uganda.  *Int J STD AIDS* in press (2006).

90. Gray RH, Li X, Kigozi G, Serwadda D, Brahmbhatt H, Wabwire-Mangen F et al. Increased risk of incident HIV during pregnancy in Rakai, Uganda: a prospective study. Lancet 2005;366(9492):1182-8.

91. Paxton LA, Kiwanuka N, Nalugoda F, Gray R, Wawer MJ. Community based study of treatment seeking among subjects with symptoms of sexually transmitted disease in rural Uganda. BMJ 1998;317(7173):1630-1.

92. Kiddugavu MG, Kiwanuka N, Wawer MJ, Serwadda D, Sewankambo NK, Wabwire-Mangen F et al. Effectiveness of syphilis treatment using azithromycin and/or benzathine penicillin in Rakai, Uganda. Sex Transm.Dis. 2005;32(1):1-6.

93. Sewankambo NK, Gray RH, Ahmad S, Serwadda D, Wabwire-Mangen F, Nalugoda F et al. Mortality associated with HIV infection in rural Rakai District, Uganda. AIDS 2000;14(15):2391-400.

94. Makumbi FE, Gray RH, Serwadda D, Nalugoda F, Kiddugavu M, Sewankambo NK et al. The incidence and prevalence of orphanhood associated with parental HIV infection: a population-based study in Rakai, Uganda. AIDS 2005;19(15):1669-76.

95. Brahmbhatt H, Kigozi G, Wabwire-Mangen F, Serwadda D, Lutalo T, Nalugoda F et al. Mortality in HIV-infected and uninfected children of HIV-infected and uninfected mothers in rural Uganda. JAIDS. 2006;41(4):504-8.

96. Porter L, Hao L, Bishai D, Serwadda D, Wawer MJ, Lutalo T et al. HIV status and union dissolution in sub-Saharan Africa: the case of Rakai, Uganda. Demography 2004;41(3):465-82.

97. Nalugoda F, Gray RH, Serwadda D, Sewankambo NK, Wabwire-Mangen F, Kiwanuka N et al. Burden of infection among heads and non-head of rural households in Rakai, Uganda. AIDS Care 2004;16(1):107-15.

98. Koenig MA, Lutalo T, Zhao F, Nalugoda F, Wabwire-Mangen F, Kiwanuka N et al. Domestic violence in rural Uganda: evidence from a community-based study. Bull.World Health Organ 2003;81(1):53-60.

99. Koenig MA, Lutalo T, Zhao F, Nalugoda F, Kiwanuka N, Wabwire-Mangen F et al. Coercive sex in rural Uganda: prevalence and associated risk factors. Soc.Sci.Med. 2004;58(4):787-98.

100. Zablotska IB, Gray RH, Serwadda D, Nalugoda F, Kigozi G, Sewankambo N et al. Alcohol use before sex and HIV acquisition: a longitudinal study in Rakai, Uganda. AIDS 2006;20(8):1191-6.

101. Nyblade LC, Menken J, Wawer MJ, Sewankambo NK, Serwadda D, Makumbi F et al. Population-based HIV testing and counseling in rural Uganda: participation and risk characteristics. J Acquir.Immune Defic.Syndr. 2001;28(5):463-70.

102. Matovu JK, Kigozi G, Nalugoda F, Wabwire-Mangen F, Gray RH. The Rakai Project counselling programme experience. Trop.Med.Int.Health 2002;7(12):1064-7.

103. Matovu JK, Gray RH, Makumbi F, Wawer MJ, Serwadda D, Kigozi G et al. Voluntary HIV counseling and testing acceptance, sexual risk behavior and HIV incidence in Rakai, Uganda. AIDS 2005;19(5):503-11.

104. Kagaayi J, Dreyfuss ML, Kigozi G, Chen MZ, Wabwire-Mangen F, Serwadda D et al. Maternal self-medication and provision of nevirapine to newborns by women in Rakai, Uganda. J Acquir.Immune Defic.Syndr. 2005;39(1):121-4.

105. Lutalo T, Kidugavu M, Wawer MJ, Serwadda D, Zabin LS, Gray RH. Trends and determinants of contraceptive use in Rakai District, Uganda, 1995-98. Stud.Fam.Plann. 2000;31(3):217-27.

106. Ahmed S, Lutalo T, Wawer M, Serwadda D, Sewankambo NK, Nalugoda F et al. HIV incidence and sexually transmitted disease prevalence associated with condom use: a population study in Rakai, Uganda. AIDS 2001;15(16):2171-9.

107. Kiwanuka N, Gray RH, Serwadda D, Li X, Sewankambo NK, Kigozi G et al. The incidence of HIV-1 associated with injections and transfusions in a prospective cohort, Rakai, Uganda. AIDS 2004;18(2):342-4.

108. Thoma M, Gray RH, Kiwanuka N, Serwadda D, Wawer M. Unsafe injections and transmission of HIV-1 in sub-Saharan Africa. Lancet 2004;363(9421):1650-1.

110. Wawer MJ, Gray RH, Serwadda D, Namukwaya Z, Makumbi F, Sewankambo N et al. Declines in HIV Prevalence in Uganda: Not as Simple as ABC. Boston, MA: 12^th^ Conference on Retroviruses and Opportunistic Infections; 2005.

111. Wawer MJ, Serwadda D, Gray RH, Sewankambo NK, Li C, Nalugoda F et al. Trends in HIV-1 prevalence may not reflect trends in incidence in mature epidemics: data from the Rakai population-based cohort, Uganda. AIDS 1997;11(8):1023-30.

112. Gray RH, Sewankambo NK, Wawer MJ, Serwadda D, Kiwanuka N, Lutalo T. Disclosure of HIV status on informed consent forms presents an ethical dilemma for protection of human subjects. J Acquir.Immune Defic.Syndr. 2006;41(2):246-8.

113. Wawer MJ, Eng SM, Serwadda D, Sewankambo NK, Kiwanuka N, Li C et al. Prevalence of Kaposi sarcoma-associated herpesvirus compared with selected sexually transmitted diseases in adolescents and young adults in rural Rakai District, Uganda. Sex Transm.Dis. 2001;28(2):77-81.

114. Gray RH, Wawer MJ, Sewankambo NK, Serwadda D, Li C, Moulton LH et al. Relative risks and population attributable fraction of incident HIV associated with symptoms of sexually transmitted diseases and treatable symptomatic sexually transmitted diseases in Rakai District, Uganda. Rakai Project Team. AIDS 1999;13(15):2113-23.

115. Laeyendecker O, Henson C, Gray RH, Nguyen RH, Horne BJ, Wawer MJ et al. Performance of a commercial, type-specific enzyme-linked immunosorbent assay for detection of herpes simplex virus type 2-specific antibodies in Ugandans. J Clin.Microbiol. 2004;42(4):1794-6.

116. Gamiel JL, Laeyendecker OB, Reynolds SJ, Ashley-Morrow R, Hogrefe W, Serwadda D et al. Development of an Algorithm for HSV-2 Testing in Africa. National STD Prevention Conference, Poster # 301. Jacksonville, Florida: 2006.

117. Serwadda D, Wawer MJ, Shah KV, Sewankambo NK, Daniel R, Li C et al. Use of a hybrid capture assay of self-collected vaginal swabs in rural Uganda for detection of human papillomavirus. J Infect.Dis. 1999;180(4):1316-9.

117a. Cogliano V, Baan R, Straif K, Grosse Y, Secretan B, El Ghissassi F, WHO International Agency for Cancer. Carcinogenicity of human papillomaviruses. Lancet Oncology 2005;6:204.

118. Safaeian M, Kiddugavu, M., Gravitt, P. E., Ssekasanvu J, Murokora D, Sklar M, Serwadda D, Wawer M, Shah, K. V., and Gray R. Comparability of self-collected vaginal swabs and physician-collected cervical swabs for detection of human papillomavirus infections in Rakai, Uganda. Sexually Transmitted Diseases . 2006. In press.

118a. Utility of self-collected vaginal swabs for studying the epidemiology of HPV infection. PhD Dissertation. Johns Hopkins University; 2006.

119. Giuliano AR, Harris R, Sedjo RL, Baldwin S, Roe D, Papenfuss MR et al. Incidence, prevalence, and clearance of type-specific human papillomavirus infections: The Young Women’s Health Study. J Infect.Dis. 2002;186(4):462-9.

120. Giuliano AR, Papenfuss M, Schneider A, Nour M, Hatch K. Risk factors for high-risk type human papillomavirus infection among Mexican-American women. Cancer Epidemiol.Biomarkers Prev. 1999;8(7):615-20.

121. Baldwin SB, Wallace DR, Papenfuss MR, Abrahamsen M, Vaught LC, Kornegay JR et al. Human papillomavirus infection in men attending a sexually transmitted disease clinic. J Infect.Dis. 2003;187(7):1064-70.

122. Baldwin SB, Djambazov B, Papenfuss M, Abrahamsen M, Denman C, Guernsey dZ et al. Chlamydial infection in women along the US-Mexico border. Int.J STD AIDS 2004;15(12):815-21.

123. Flores R, Papenfuss M, Klimecki WT, Giuliano AR. Cross-sectional analysis of oncogenic HPV viral load and cervical intraepithelial neoplasia. Int.J Cancer 2006;118(5):1187-93.

125. Koutsky LA, Ault KA, Wheeler CM, Brown DR, Barr E, Alvarez FB et al. A controlled trial of a human papillomavirus type 16 vaccine. N.Engl.J.Med. 2002;347(21):1645-51.

126. Gravitt PE, Peyton CL, Alessi TQ, Wheeler CM, Coutlee F, Hildesheim A et al. Improved amplification of genital human papillomaviruses. J Clin.Microbiol. 2000;38(1):357-61.

126.a Gravitt PE, Peyton CL, Apple RJ, Wheeler CM. Genotyping of 27 human papillomavirus types by using L1 consensus PCR products by a single-hybridization, reverse line blot detection method. J Clin Microbiol 1998;36:3020-7.

127.Maxwell JA. Qualitative research; an interactive approach. 2^nd^ ed. Sage Publications, Inc. 2004

128. Morgan DL> Focus groups as qualitative research. 2^nd^ ed. Sage Publications Inc. 1997

131. Meinert CL and Tonascia S. Clinical Trials: Design, Conduct and Analysis. Monographs in Epidemiology and Biostatistics Vol 8. Oxford university Press, 1986.

132. Liang KY, Zeger SL. Longitudinal Data-Analysis Using Generalized Linear-Models. Biometrika 1986;73(1):13-22.

133. McCullagh P. Regression-Models for Ordinal Data. Journal of the Royal Statistical Society Series B-Methodological 1980;42(2):109-42.

134. Stram DO, Wei LJ, Ware JH. Analysis of Repeated Ordered Categorical Outcomes with Possibly Missing Observations and Time-Dependent Covariates. Journal of the American Statistical Association 1988;83(403):631-7.

135. Rao CR. Theory of Least Squares When Parameters Are Stochastic and Its Application to Analysis of Growth Curves. Biometrika 1965;52:447.

136. Rao CR. Simultaneous Estimation of Parameters in Different Linear-Models and Applications to Biometric Problems. Biometrics 1975;31(2):545-54.

137. Laird NM, Ware JH. Random-Effects Models for Longitudinal Data. Biometrics 1982;38(4):963-74.

138. Stiratelli R, Laird N, Ware JH. Random-Effects Models for Serial Observations with Binary Response. Biometrics 1984;40(4):961-71.

139. Anderson DA, Aitkin M. Variance Component Models with Binary Response - Interviewer Variability. Journal of the Royal Statistical Society Series B-Methodological 1985;47(2):203-10.

140. Wang MC, Chang SH. Nonparametric estimation of a recurrent survival function. Journal of the American Statistical Association 1999;94(445):146-53.

141. Wang MC, Chen YQ. Nonparametric and semiparametric trend analysis for stratified recurrence times. Biometrics 2000;56(3):789-94.

142. Wang MC, Qin J, Chiang CT. Analyzing recurrent event data with informative censoring. Journal of the American Statistical Association 2001;96(455):1057-65.

143. Wang MC, Chiang CT. Non-parametric methods for recurrent event data with informative and non-informative censorings. Statistics in Medicine 2002;21(3):445-56.

144. Huang Y, Wang MC. Frequency of recurrent events at failure time: Modeling and inference. Journal of the American Statistical Association 2003;98(463):663-70.

145. Lin DY, Sun W, Ying ZL. Nonparametric estimation of the gap time distributions for serial events with censored data. Biometrika 1999;86(1):59-70.

147. Lin DY, Wei LJ, Yang I, Ying Z. Semiparametric regression for the mean and rate functions of recurrent events. Journal of the Royal Statistical Society Series B-Statistical Methodology 2000;62:711-30.

149. Chen YQ, Wang MC, Huang Y. Semiparametric regression analysis on longitudinal pattern of recurrent gap times. Biostatistics 2004;5(2):277-90.

150. Prentice RL, Williams BJ, Peterson AV. On the Regression-Analysis of Multivariate Failure Time Data. Biometrika 1981;68(2):373-9.

151. Wawer MJ, Sewankambo NK, Serwadda D, Quinn TC, Paxton LA, Kiwanuka N et al. Control of sexually transmitted diseases for AIDS prevention in Uganda: a randomised community trial. Rakai Project Study Group. Lancet 1999;353(9152):525-35.

152. Wang MC, Chiang CT. Non-parametric methods for recurrent event data with informative and non-informative censorings. Stat.Med. 2002;21(3):445-56.

153. Kreiss JK, Hopkins SG. The association between circumcision status and human immunodeficiency virus infection among homosexual men. J.Infect.Dis. 1993;168(6):1404-8.

154. Wawer MJ, Sewankambo NK, Serwadda D, Quinn TC, Paxton LA, Kiwanuka N et al. Control of sexually transmitted diseases for AIDS prevention in Uganda: a randomised community trial. Rakai Project Study Group. Lancet 1999;353(9152):525-35.

155. Hanley JA. A heuristic approach to the formulas for population attributable fraction. J Epidemiol.Community Health 2001;55(7):508-14.

156. Rothman KJ, Greenland S. Modern Epidemiology. 2nded. Philadelphia: Lippincott Raven; 1998.

157. Greenland S, Drescher K. Maximum likelihood estimation of the attributable fraction from logistic models. Biometrics 1993;49(3):865-72.

158. Strauss AL, Corbin J. Basics of Qualitative Research: Techniques and Procedures for Developing Grounded Theory. p.312. 2nded. Thousand Oaks, CA.: Sage Publications; 1998.

**APPENDIX 1.**

# MALE CIRCUMCISION FOR HIV PREVENTION STUDY

# INFORMATION SHEET FOR MALE PARTICIPANTS AT UNBLINDING OF TRIAL OUTCOMES

Hello, my name is ____________________. I work for the Rakai Health Sciences Program.

As you were previously informed, the circumcision study you were enrolled in planned to follow up participants for 2 years. However, on December 12, 2006, we were informed by the Data and Safety Monitoring Board (DSMB) of the NIH-funded circumcision trial that circumcision reduces the risk of acquiring HIV by about half in HIV-negative men. Two other studies in Kenya and South Africa had similar results. For this reason, the NIH-funded study has been stopped early. Therefore, we are now offering circumcision surgery to HIV- negative uncircumcised men.

Men are urged provide a blood sample and to receive their HIV results, to see if they are eligible to receive circumcision as a service at this time.

In addition, on December 19, 2006, the DSMB for the Gates-funded trial of male circumcision, which included both HIV+ and HIV-negative men, also stopped the enrolment of new participants. Males enrolled in that trial, and their partners, continues to be followed. On March 1^st^, 2007 the Data Safety and Monitoring Board that examined data from the Gates funded trial revealed the following results:

- That there is a trend towards increased risk of HIV transmission from HIV infected circumcised men to their uninfected sexual partners in individuals who resume sex before wound healing.
- That HIV infected men who were circumcised had a 50 percent lower rate of genital ulceration.

Further analysis is still ongoing, and you will be informed of new results as soon as they are available. The Gates trial Data Safety and monitoring Board reviewed and approved a new protocol under which HIV+ uncircumcised men in the Gates or NIH trial will be offered surgery. The DSMB has also recommended that we carry out studies to further understand wound healing in both HIV positive and HIV negative men. Some participants will be asked to participate in this study where more frequent weekly follow up will be conducted until complete wound healing is certified. As each extra weekly postoperative visit 5 mL (one teaspoonful) of venous blood will be collected for various tests, including the determination of the amount of virus in the body (HIV viral load) to assess whether surgery affects the amount of virus in blood. Men are strongly urged not to resume sex before certified/complete wound healing since this may expose them or their partners to a higher risk of HIV infection.

The DSMB also recommended that an extra visit be done for HIV+ men and some HIV negative men and their female partners at ~18 months post enrollment in order to study HIV and STDs, and sexual behaviors at a time earlier than 2 years. Men and women who make this visit will be compensated for their time and transport.

Since not all HIV-negative men will decide to be circumcised and since, many HIV-positive men have been circumcised and more are being circumcised now, being circumcised does not mean that a man is uninfected or infected with HIV. At this time, we do know that complications following circumcision are similar in both HIV-negative and HIV+ men.

However, it is very important for you to know that adult male circumcision alone does not completely protect men from HIV. Because circumcised men can still get HIV, they must continue to practice safe sex, which means sexual abstinence, or being faithful to a partner who is HIV-negative, or using condoms with all other sexual partners.

If you have not yet been circumcised, you are now free to decide whether you would like to receive the procedure from the Rakai Program.

*****************************************

Have you been circumcised?

**Note to person getting the informed consent:** If participant answers “no”, please read him the following section in italics. If he answers he has been circumcised, skip the section in italics.

*If you decide to accept circumcision now, please let me know, and I will make arrangements for you to be scheduled for surgery. If, however, you wish to wait to make your decision, you will need to contact the Rakai program office (phone number 0772 621 515) or resident counselor or your community health mobilizer (mention name of the resident counselor or community health mobilizer) when you are ready, for the operation. Please note that the Rakai Program will be able to offer free surgery up to the end of October, 2007. Men who wish to receive circumcision will be given a date for surgery which will be performed at the Rakai Program clinic in Kalisizo. Men who have circumcision will receive free surgery and any needed postoperative treatment, and will be given instructions on wound care and when it is safe to resume sex after the wound has healed (around 28 days after surgery). We will try to see all men on the first day and ask them to return for a check up between 5-9 days and at 4-6 weeks after surgery. Men who develop problems will be asked to come to the hubs or to the Rakai Program clinic at Kalisizo for care. Men will be offered circumcision as a service, and will receive 6550 Shillings on the day of surgery, on the first postoperative day, on day 5-9, and at the 4-6 weeks post surgery visits for a total of $15.00 (Uganda shillings 26,200) as compensation for the time lost from work.*

*Men who will be asked to come more frequently will be given ~3$ for each visit.*

*Would you like to accept circumcision? 1*

*Would you like to make your decision later? 2*

*Would you prefer not to get circumcised? 3*

*If the participant is willing to accept circumcision now, give him information on the date and place he should come in order to be provided with information on when to come to the Kalisizo clinic for surgery****.***

*If the participant decides to defer his decision to get surgery, refer him to the appropriate contact person in his community if he decides he would like to schedule surgery in the future.*

***********************************************************

At this visit, we would like to administer a questionnaire like the ones we previously administered to you during this trial. We would also like to collect some specimens. These include a blood sample (about 10mls or two teaspoons), urine, and swabs from the shaft of your penis and from under the foreskin. These samples will be tested for infections such as HIV, syphilis, other STDs, and potentially for ways that the body protects itself from infections. Treatment will be provided for syphilis and for any STDs that might be diagnosed at this visit. There are no health risks associated with taking the swabs and urine. The risks of taking a blood sample are the same as during the trial of male circumcision and can include bruising or bleeding. As in the trial, the blood sample will be collected by a trained Rakai Program health professional, in order to minimize risk. If you decline to answer the survey or to provide any or all of the samples, that will not affect your right to receive services from the Rakai Program. I will ask you to sign a section at the end of this form to indicate whether or not you agree to provide samples.

You will receive compensation for your time today.

You will continue to have free access to the medical and other services offered by the Rakai Program in its study communities. However, you will **not** receive any compensation for future visits made for medical treatment or to receive services.

If you develop any medical condition that you think is related to your participation in the circumcision study, please don’t hesitate to inform us through your community health mobilizer, the project resident counselor based in your community, or call 0772 **621 515** for assistance. Alternatively, you may choose to report to any government health centre or clinic nearest to you. **However, if you choose to come to Kalisizo, you will not receive any transport refund or compensation for time lost.**

A team of health educators is visiting communities and conducting meetings to inform community members of the findings of the study.

Although your participation in the trial of male circumcision study has ended, we have applied for funding to conduct a new study in which men who participated in the Rakai Health Sciences Program circumcision trials will be followed for up to 5 more years, to assess the effects of being circumcised over time. Thus, today **or in the near future,** we may ask whether you consent to the longer term follow up. Whether or not you consent to the longer term follow up will not affect your right to receive free circumcision from the program, or to receive other services offered by the Rakai Program in its study communities.

In addition, please note that you can enroll in other Rakai studies in the future. The Rakai Health Sciences Program may recontact some or all past circumcision study participants to invite them to participate in other studies involving components such as interviews, focus groups, sample collection or clinical studies. If you are re-contacted, the additional research will be described to you and you will be asked to provide your consent to participate in the additional research. If you prefer not to participate in any additional study or studies, you will be free to decline. As is the Rakai Program policy, your circumcision data will remain confidential. At the end of this information sheet, you will be asked to sign whether you agree to or decline to be contacted for additional studies because of the information you provided in the circumcision trial.

If you have participated in other Rakai Program studies or services, the data you provide as part of the circumcision study may be linked to the data you provide or have provided in those other studies or services. .

On behalf of the Rakai Program, let me thank you once again for your participation in the male circumcision trial.

**Signature/thumbprint indicating that he has been informed about the availability of circumcision**

Name of participant:_______________________________ Date:_____________________

# Signature of participant:_____________________

# Signature/thumbprint indicating consent to be contacted for additional studies for which you are selected through circumcision study interview data or lab results:

Agree to be contacted for future studies: ________________

Declines to be contacted for future studies: ________________

**Signature/thumbprint indicating that he has agreed or disagreed to provide the biological samples described in this form**

Agree to provide _______________________________

Disagree to provide samples ______________________________________________

You may agree to one of the following options with regard to future use of stored samples you provide, but permission will be sought from the Science and Ethics Committee to test the samples for conditions other than those outlined in this form and in the protocol.

Please sign (thumb print or X if non-literate) on one of the lines below, to indicate the option you select.

1. That the samples you provide may be stored and identified by your study number ___________

2. That the samples you provide may be stored but not identified by your study number. ________

3. That your samples should not be stored after testing is completed. ______________

Name of field worker:__________________________________ Date:___________________

Signature of field worker:_______________________________

# APPENDIX 2.

**RAKAI HEALTH SCIENCES PROGRAM**

**COMMUNITY MESSAGES ON ADULT MALE CIRCUMCISION**

Since 2003, Rakai Health Sciences Program has been conducting two studies on Male circumcision in Rakai District. One study, funded by NIH looked at whether male circumcision might reduce the chances that a man gets HIV. The other funded by the Gates Foundation looked at whether circumcision is safe in HIV+ men and whether it might reduce the chance that a positive man transmits the virus to a woman partner.

On Dec 12, 2006, an independent group, called the Data Safety and Monitoring Board (DSMB) which had the responsibility for monitoring the results from the Rakai NIH sponsored trial came to the conclusion that male circumcision reduced the risk for HIV-negative men acquiring HIV from a female partner by about half. The results from two other studies of male circumcision, one in South Africa and the second in Kisumu, Kenya, also showed a similar reduction in the risk of men getting HIV.

It is very important for you to know that adult male circumcision can only partly reduce the man’s risk of getting HIV. Because circumcised men can still get HIV and, since HIV+ men can transmit HIV to their partners whether or not they are circumcised, we strongly urge all people to practice safe sex. That means abstaining, or being faithful to one partner who has been HIV tested and is HIV-negative, or using condoms every time in any other circumstances. If either partner is HIV-positive, the couple must practice safe sex, that is, either abstain or use condoms every time they have sex, whether or not the man is circumcised.

Men who have not yet got their HIV results should do so, and should encourage their partners to get their results, and receive couples’ counseling to share results with partners. People should also avoid getting any injections with unclean needles and any transfusions if the blood had not been tested for HIV.

Given these results, Rakai Health Sciences Program will offer circumcision to all HIV-negative uncircumcised men enrolled in both studies.

In addition, on March 1^st^, 2007 the Data Safety and Monitoring Board that examined data from the Gates funded trial revealed the following results:

- That there is a trend towards increased risk of HIV transmission from HIV infected circumcised men to their uninfected sexual partners in individuals who resume sex before wound healing.
- That HIV infected men who were circumcised had a 50 percent lower rate of genital ulceration.

Males enrolled in that Gates funded trial, and their partners, continue to be followed. Further analysis is still going on, and you will be informed of any new results in future. The Gates trial Data Safety and Monitoring Board reviewed an approved a new protocol under which HIV+ uncircumcised men in the Gates or NIH trial will be offered surgery. The DSMB has also recommended that we carry out studies to further understand wound healing in both HIV-positive and HIV-negative men. Some participants will be asked to participate in this study where more frequent weekly follow up visits will be made until complete wound healing is certified. Men are strongly urged not to resume sex before certified complete wound healing since this may expose them or their partners to a higher risk of HIV infection.

Being circumcised or not does not mean that a man does or does not have HIV since not all men will decide to be circumcised and since, many HIV-positive men have already been circumcised. At this time, we do know that complications of circumcision are similar in both HIV-negative and HIV+ men.

In order to understand the long term impact of adult male circumcision on risk of HIV, risk-taking behavior and attitudes, Rakai Health Sciences Program plans to continue to follow up participants from both the NIH and the Gates-funded studies over the next 4-5 years.

Before medical adult male circumcision becomes widely available to all HIV-negative men as one of the HIV prevention methods, we must wait for the Uganda Ministry of Health policy guidelines. When these guidelines become available, information will be provided to the general public.

**Key message Points**

1. Circumcision of HIV-negative adult men, provided by skilled medical personnel, is beneficial in reducing a man’s risk of HIV acquisition
2. Adult male circumcision alone does not completely protect men from acquiring HIV.
3. RSHP plans to continue to follow-up study participants in order to establish the long term impact of male circumcision.
4. Circumcising HIV-positive men has shown a trend towards increased risk of HIV transmission from HIV infected circumcised men to their uninfected sexual partners among men who resume sex before wound healing.

**For further information on Adult Male Circumcision trial results, please contact the following:**

1. Dr. Kigozi Godfrey, RHSP Kalisizo 0772 593 267

2. Dr. Kiwanuka Noah, RHSP Kalisizo 0782 788 036

3. Prof. Serwadda David, Makerere University, Kampala 0772 768 089

3. Mr. Nalugoda Fred, RHSP Kalisizo 0772 510 402

# APPENDIX 3

**CONSENT FOR PARTICIPATION IN THE POST-CIRCUMCISION TRIAL SURVEILLANCE**

**English Version**

# Principal Investigators: Ronald H. Gray, M.D., Johns Hopkins University and

Maria J. Wawer, M.D., Johns Hopkins University

**Ugandan Principal Investigator:** Dr. David Serwadda and Dr. Nelson Sewankambo

**Hello, my name is __________________________, and I work for the Rakai Health sciences Program.**

Because you completed 24 months of the NIH-funded, randomized trial of male circumcision, Rakai, Uganda, you are now being asked to take part in a post-trial, follow-up research study. This study is being carried out by The Rakai Health Sciences Program, the Johns Hopkins University, and Makerere University, and is sponsored by the National Institute of Allergy and Infectious Diseases (NIAID).

This document is a consent form, which will provide you information about this study. The staff here will discuss all information with you in person, and you are free to ask questions at any time. Before you can decide whether or not to take part in the study, you need to know the purpose of the study, the possible risks and benefits, and what is expected of you. This process is called informed consent. Once you understand the study, and if you decide to take part, you will be asked to sign this consent form. You will be given a copy to keep.

**PURPOSE OF THE STUDY**

Studies done in Uganda, Kenya, and South Africa have shown that male circumcision surgery reduces the rates of HIV infection in men by more than half in the first 1-2 years after surgery. However, male circumcision does not provide 100% protection, and certain behaviors continue to put people at higher risk for getting HIV and other sexually transmitted infections (STI’s). The purpose of this study is to look at the long-term effects of male circumcision on preventing HIV and other STI’s in Rakai, Uganda.

**ELIGIBILITY**

All participants of the NIH-funded randomized trial of male circumcision who completed 24 months are eligible to participate in this follow-up study. This includes men who received circumcision surgery and men who did not receive circumcision surgery in the trial. If you did not already have the surgery, you will be offered circumcision surgery in this follow-up study. It is up to you to decide if you want to be circumcised or not. You are not required to receive the circumcision surgery in order to participate in this study. This study will include up to 4996 men in the Rakai District.

This study will be conducted as a subgroup of the Rakai Community Cohort Study (RCCS). Therefore, should you decide to join this follow up study, you will also be required to enroll in the RCCS. You will be asked to review and sign a separate consent form which will describe the procedures, risks and benefits of the RCCS.

**YOUR PARTICIPATION IS VOLUNTARY**

Deciding to join this study is completely up to you. It is important that you know the following:

• Your participation is entirely voluntary.

• You may decide not to take part or to withdraw from the study at any time. You can still take part in other research studies here, if available.

**LENGTH OF THE STUDY & STUDY PROCEDURES**

The study is expected to end by 07/31/2012. You are being asked to participate for the next 5 years, and to complete one study visit per year during that time.

At each of the follow-up/RCCS study visits you will be interviewed about your social and demographic characteristics, sexual behaviors and health. You will also be asked to provide a sample of blood and urine to test for HIV and other sexually transmitted infections, and to provide a penile swab to test for a virus called HPV. Some of blood and swabs not used right away for testing will be stored for future research. At each study visit you will also receive counseling about ways to prevent HIV and other STI’s.

**STOPPING THE STUDY**

If you agree to take part in this study, it is important for you to keep all your appointments. However, you may decide to stop or leave the study at any time. You will not lose any benefits that you would have if you had not joined the study.

Also, the study may be stopped without your consent for the following reasons:

•  If your private doctor or the study staff feels that staying in the study is harmful to you.

•  If you don't keep appointments or follow study procedures.

•  If the study sponsor, the Institutional Review Board (IRB) or Ethics Committee in the US and Uganda, decides to stop or cancel the study (IRB’s and Ethics Committees protect the safety and rights of research participants).

**POSSIBLE RISKS AND BENEFITS**

The risks of taking blood samples include pain, bleeding, swelling, bruising or infection where the needle enters the skin, and in rare cases lightheadedness or faint feeling. The risks of penile swabs include discomfort or slight pain while the swab is pressed against the penis, and possible pain during urination after the swab is taken.

There is also the risk that answering personal questions about sexual activity and your personal habits can make you feel uncomfortable, embarrassed or stressed. You may also feel distressed if you test positive for HIV and other STI’s.

Should you wish to have the circumcision surgery in this study, you will be asked to review and sign a separate consent form which will describe the procedures, risks and benefits of the surgery, pain medication, compensation for lost time, and transportation home after surgery.

It is important that you know that neither circumcision surgery, nor participation in this study, ensures that you will be protected from becoming HIV infected. *It is very important that you avoid any behavior that would put you at risk for getting infected with HIV.*

It is possible that you will receive no benefit from taking part in this follow-up study, or from studies done on your stored samples. However, information learned may help other people with HIV and other STI’s.

**COSTS TO YOU**

There is no cost to you for any of the clinic visits or laboratory tests for this follow-up study.

**CONFIDENTIALITY**

Your study records will be confidential to the extent allowed by law. All of your study information will be identified by a code, not your name. Only you and the people working on the study here will know your code number. No personal information from your records will be released without your written permission.

Data will be entered onto a computer, where it will be identified by study code only, and the computers are password protected. Your responses will not be sent on the Internet or by e-mail.

Medical records obtained to confirm STD diagnosis and treatment will become part of your study data file and will be treated in the same confidential manner as all other information in this

You will not be personally identified in any publication or presentation about this study.

Your records may be reviewed, under guidelines of the U.S. Federal Privacy Act, by the National Institute of Allergy and Infectious Diseases, NIH and its study monitor contractors, study staff, the Johns Hopkins University, and the IRB and Ethics Committee of the US and Uganda***.***

**MONITORING OF THE STUDY**

The study will be monitored by the NIH National Institute of Allergy and Infectious Diseases and their contractors. They will pay close attention for any problems which could arise with participants in the study

**NEW FINDINGS**

You will be told about any new information learned during the course of the study that might cause you to change your mind about staying in the study. At the end of the study, you will be told when study results may be available and how to learn about them.

**POLICY ABOUT RESEARCH-RELATED INJURIES (SITE-SPECIFIC STATEMENT)**

If you are injured as a result of participating in this study, treatment will be made available through the RCCS. No payment for study-related injuries is available through the National Institute of Allergy and Infectious Diseases (NIAID), NIH.

**QUESTIONS/POINTS OF CONTACT**

If you have any questions, please ask, and we will do our best to answer them. If you have additional questions or if you need to discuss any other aspect of the study, you can contact:

Dr. Joseph Kagaayi, Dr. Godfrey Kigozi, and Dr. Gertrude Nakigozi, Medical Officers, and Mr. Fred Nalugoda Director of Field Activities Rakai Program office in Kalisizo (Tel 0772- 405861)

Dr. Nelson Sewankambo or Dr. David Serwadda, Ugandan Principal Investigators, at the Department of Medicine at Makerere University in Kampala (Tel 041-530020) and Institute of Public Health/MUK (tel: 041-545001) respectively.

If you have any questions concerning your rights as a participant in this research, please contact the Chairman of Science and Ethics committee of UVRI (tel 041-320385/6).

**STATEMENT OF PARTICIPANT CONSENT**

**CONSENT OF ADULT PARTICIPANT/ ASSENT OF UNEMANCIPATED MINOR**

I ___________________________, age, ______have been asked to participate in a five year follow up study of men who participated in the NIH funded trial of Male Circumcision for HIV Prevention. The principal investigator Dr. Sewankambo/ Dr. Serwadda or the medical officers Dr. Kigozi, or Dr. Kagaayi or Dr. Gertrude Nakigozi or their representative, has explained the study to me, how long it will last, the testing I will undergo, and risks that I might take. The information above has been read to me. I have been given an opportunity to ask questions about this research project. All questions were answered in a way that I understand. If I have other questions about this research, I can ask the study representative, the field medical officers named above or Dr. Sewankambo, or Dr. Serwadda.

I understand that I will be required to participate in the Rakai Community Cohort Study, and will have to read and sign the RCCS consent form.

I understand that my participation is voluntary, and that I can decline to be in the study or leave the study at any time. If I decline to participate or if I leave the study, I will not lose any benefits or access to Rakai Program services. I am signing my name below to indicate my consent participate in this project. I will be given a copy of the signed consent form.

_____________________________, _______________

SIGNATURE OF PARTICIPANT DATE

(thumbprint if non-literate)

_____________________________ _____________________________

PRINTED NAME OF PARTICIPANT SIGNATURE/THUMBPRINT

INDICATING CONSENT

ADDRESS OF PARTICIPANT

Signature/thumbprint indicating use of stored samples to test for genetic markers.

I accept

_____________________________, _______________

SIGNATURE OF PARTICIPANT DATE

(thumbprint if non-literate)

I decline

_____________________________, _______________

SIGNATURE OF PARTICIPANT DATE

(thumbprint if non-literate)

_____________________________ ___________________________ ____________

SIGNATURE OF WITNESS PRINTED NAME OF WITNESS DATE (same as participant’s)

_____________________________ ________

SIGNATURE OF INVESTIGATOR ELICITING CONSENT DATE

**CONSENT OF PARENT OR GUARDIAN** ***if participant is an unemancipated minor under age 18.***

I have been asked whether my son/ward may participate in the five year follow up study of men who participated in the NIH funded trial of Male Circumcision for HIV Prevention. The principal investigator Dr. Sewankambo/ Dr. Serwadda or the field medical officers Dr. Kigozi, or Dr. Kagaayi or Dr. Nakigozi or their representative has explained the study to me, its duration, the testing that my child/ward will be asked to undergo, and possible risks. I understand that my child/ward will be required to participate in the Rakai Community Cohort Study, and I will have to read and sign the RCCS consent form. The information above has been read to me. I have been given an opportunity to ask questions about this research project. All questions were answered in a way that I understand. If I have other questions about this research, I can ask the study medical officers named above or Dr. Sewankambo or Dr. Serwadda.

I understand that my agreement to let my child/ward participate is voluntary, and that I can decline to let my child participate or ask that my child leave the study at any time, without losing access to Rakai Program services for myself or my child. I also understand that s/he has the right to voluntarily refuse to participate in all or part of the study. I am signing my name below to indicate my assent for my child/ward to participate in this project.

__________________________________, _______________

SIGNATURE OF PARENT OR GUARDIAN DATE

__________________________________, ____________________________

PRINTED NAME OF PARENT/GUARDIAN ADDRESS OF PARENT/GUARDIAN

_____________________________ ___________________________ ____________

SIGNATURE OF WITNESS PRINTED NAME OF WITNESS DATE (same as participant’s)

_____________________________ ________

SIGNATURE OF INVESTIGATOR ELICITING CONSENT DATE

ELICITING CONSENT

# APPENDIX 3A.

Luganda Version of CONSENT FOR PARTICIPATION IN THE POST-CIRCUMCISION TRIAL SURVEILLANCE

**EKIWANDIIKO EKIRAGA OKUKKIRIZA OKWONGERA OKWETABA MU KUNOONYEREZA NG’OKUNOONYEREZA OKUKWATA KU KUKOMOLA ABASAJJA KUWEDDE.**

**Abakulira okunoonyereza mu America:**

Ronald H. Gray M.D, Johns Hopkins University ne

Maria J Wawer M.D, Johns Hopkins University

**Abakulira okunoonyereza mu Uganda:** Dr. David Sserwadda ne Dr.Nelson Sewankambo

Nkulamusizza, erinnya lyange nze _______________________, era nga nkolera mu kitongole kya Rakai Health Sciences Program.

Olw’okuba wamaliriza emyezi gyo 24 egy’okunoonyereza okukwata ku kukomola abasajja okwawagirwa NIH mu Rakai, Uganda, osabibwa okwetaba mu kunoonyereza okw’okwongera okugoberera abo abetaba mu kunoonyereza okwo. Okunoonyereza kuno kukolebwa ekitongole kya Rakai Health Sciences program, Johns Hopkins University wamu ne Makerere University era nga kusasulirwa aba National Institute of Allergy ne infectious diseases (NAID)

Ekiwandiiko kino ekiraga okukkiriza okwetaba mu kunoonyereza, kijja kukuwa amawulire agakwata ku kunoonyereza kuno. Abakozi ba Rakai Program abakola ku kunoonyereza kuno bajja kukubaganya naawe ebirowoozo era oli wa ddembe okubuuza ebibuuzo obudde bwonna. Nga tonnasalawo oba oneetaba mu kunoonyereza oba nedda, wetaaga okumanya ekigendererwa ky’okunoonyereza, obuzibu obuyinza okubaawo, emigaso gy’okwetaba mukunoonyereza, wamu ne kye tukusuubiramu.. Emitendera gino egiyitibwamu giyamba omuntu okusalawo nga yeesimidde. Bw’omala okutegeera ebikwata ku kunoonyereza, n’osalawo okukwetabamu ojja kusabibwa okussa omukono ku kiwandiiko kino. Ojja kuweebwako kkopi ey’okutereka.

**EKIGENDERERWA KY’OKUNOONYEREZA**

Okunoonyereza okukoleddwa mu Uganda, Kenya ne South Africa kulaze nti okukomola abasajja nga kukoleddwa abasawo abatendeke obulungi kukendeeza obuzibu/akatyabaga k’okukwatibwa akawuka ka siriimu mu basajja ebitundu ebisukka ku kimu kyakubiri wakati w’ebbanga ery’omyaka ogumu n’ebiri nga bamaze okubakomola. Wabula okukomola abasajja tekumalirawo ddala katyabaga ka kukwatibwa kawuka ka siriimu, era n’enneyisa ezimu zongera okuteeka abantu mu katyabaga k’okukwatibwa akawuka ka siriimu n’endwadde endala ez’obukaba. Ekigendererwa ky’okunoonyereza kuno, kwe kwongera okutunuulira okulaba oba okukomola abasajja kuyamba mu kuziyiza akawuka ka siriimu n’endwadde endala ez’obukaba nga wayiseewo ekiseera ekiwanvu okuva omuntu lwe yakomolebwa mu Rakai, Uganda.

**EBISANYIZO BW’OKWENYIGIRA MU KUNOONYEREZA.**

Abantu bonna abeetaba mu kunoonyereza kw’okukomola abasajja abaali mu kibinja ekya sasulirwa NIH nga baamaliriza emyezi 24 be bagwanidde okwetaba mu kunoonyereza kw’okwongera okugobererwa. Mu bano otwaliramu abasajja abaakomolebwa n’abo abataakomolebwa. Bw’oba tokomolebwanga ate nga oyagala okukomolebwa, ojja kuwebwa omukisa gw’okukomolebwa mu kunoonyereza kuno okwokugobererwa. Kiri gy’oli okusalawo oba oyagala okomolebwa oba nedda. Okwetaba mu kunoonyereza kuno tekitegeza nti olina kukomolebwa. Okunoonyereza kuno kujja kwetabwamu abasajja nga 4996 okuva mu Rakai disitulikiti n’ebitundu ebiriranyeewo.

Okunoonyereza kuno kujja kugattibwa ku kunoonyereza kwa Rakai program mw’ekyalira abantu buli mwaka (RCCS), kukolebwe ng’ekimu ku bitundu by’okunoonyereza kuno. N’olwekyo, singa osalawo okwetaba mu kunoonyereza kw’okugobererwa kuno ojja kuba oyingizibwa mu kunoonyereza kwa RCCS mwe bakyalira abantu buli mwaka. Ojja kusabibwa okwejjukanya n’okussa omukono ku kiwandiiko eky’enjawulo ekinaakubuulira engeri RCCS gy’ekolamu emirimo gyayo, by’onaganyulwa n’obuzibu obuyinza okubaawo nga wetabye mu kunoonyereza kuno okwa RCCS.

**OKWETABA MU KUNOONYEREZA KUNO KWA KYEYAGALIRE.**

Okusalawo okwetaba mu kunoonyereza kuno kiri eri ggwe. Kya mugaso okumanya bino wammanga.

- Okwetaba mu kunoonyereza kuno kwa kyeyagalire.
- Oli waddembe okusalawo obuteetaba mu kunoonyereza kuno oba okukuvaamu ekiseera kyonna. Osobola okusigala nga wetaba mu kunoonyereza okulala okwa Rakai program bwe kuba wekuli.

**EBBANGA OKUNOONYEREZA LYE KUNATWALA N’ENKOLA NGA BW’ENEEBA.**

Okunoonyereza kusuubirwa okuggwa nga 31.07,2012

Osabibwa okwetaba mu kunoonyereza kuno okumala ebbanga lya myaka etaano ng’okyalirwa omulundi gumu mu mwaka.

Buli mulundi lwe banaakukyaliranga ojja kubuuzibwanga ebibuuzo ebikwata ku by’obulamu, enneeyisa yo ng’omuntu, n’ebikwata ku by’okwegatta.

Tujja kukusaba otuweeyo akasaayi n’omusulo okukeberamu akawuka ka siriimu n’endwadde endala ez’obukaba, ne kappamba okuva ku kasolo ko okukeberamu akawuka akaleeta cancer ku kasolo (HPV). Akamu ku kasaayi ne bu ppamba obunaaba butakebereddwawo bujja kuterekebwa busobole okukozesebwa mu konoonyereza mu biseera eby’omu maaso.

Buli mulundi gwe tunaakukyalira ojja kubudaabudibwa ku ngeri gy’oyinza okwewalamu akawuka ka siriimu n’endwadde endala ez’obukaba.

**OKUYIMIRIZA OKUNOONYEREZA NGA TEKUNNAGGWA**

Bw’okkiriza okwetaba mu kunoonyereza kuno kya mugaso okubeerangawo buli lwe bakugambye nti bakwetaaga. Wabula oyinza okusalawo okubivaamu obudde bwonna. Tojja kufiirwa kintu kyonna kye wandifunye singa teweetaba mu kunoonyereza.

Wabula era okunoonyereza kuno kusobola okukomezebwa nga teweyagalidde olw’ensonga zino wammanga.

- singa omusawo wo (private doctor) oba abakola ku kunoonyereza balowooza nti okusigalakwo mu kunoonyereza kuyinza okuba okw’omutawaana eri obulamu bwo.
- Bw’oba totuukiriza nkyalo zo oba bw’oba togoberera nkola ya kunoonyereza.
- Singa abasasulira okunoonyereza oba abeekenneenya amateeka agafuga eby’okunoonyereza (IRBs) mu Uganda ne mu America basalawo okuyimiriza oba okusazaamu okunoonyereza. (IRBs bwe bukiiko obukola ku bulamu n’eddembe ly’abeetaba mu kunoonyereza).

**EBIZIBU EBIYINZA OKUKUTUUKAKO N’EBYONOGANYULWAMU.**

- Ebizibu ebiyinza okubaawo nga bakuggyeeko omusaayi, mwe muli; okulumizibwa, okuvaamu otusaayi, okuzimba, okunuubuka oba okutana we baba bajje akasaayi, oluusi oyinza okufuna okulumizibwamu omutwe oba okuwulira ng’afunye kammunguluze.
- Ekizibu ekiyinza okubaawo ng’ojjibwako ka ppamba ku kasolo, kwe kuwulira obubi oba obulumi nga bajjako ka ppamba ku kasolo oba okuwulira obulumi ng’ofuuyisa nga bamazze okukujjako ka ppamba.

Waliwo era obuzibu nti okuddamu ebibuuzo ebikukwatako mu by’okwegatta n’enneyisa yo, kiiyinza okukuleetera okuwulira obubi, obuswavu oba obutawulira mirembe.

Oyinza obutawulira mirembe nga okebereddwa n’osangibwa ng’olina akawuka ka siriimu n’endwadde z’obukaba endala.

Singa oneetaaga okukomolebwa mu kunoonyereza kuno, ojja kusabibwa okw’ekenneenya era osse omukono ku kiwandiiko ekirala ekinnyonnyola enkola, obuzibu n’emigaso egiri mu kukomolebwa, eddagala erikkakanya obulumi, okusasulira ebiseera byo, n’entambula ekuzzaayo awaka ng’omaze okukomolebwa.

Kikulu okumanya nti okukomolebwa abasawo abatendeke oba okwetaba mu kunoonyereza kuno tebisobola kukakasa nti tosobola kukwatibwa kawuka ka siriimu. ***Kikulu nnyo ddala era kyetaagisa nnyo okwewala enneeyisa yonna eyinza okukuleetera/okukwasa akawuka ka siriimu.***

Kisoboka nti oyinza obutabaako na ky’ofuna mu kwetaba mu kunoonyereza kuno okw’okwongera okugobererwa, oba mu binaava mu kunoonyereza ebikwata ku byakujjibwako n’ebiterekebwa (omusaayi, omusulo, bu ppamba). Wabula amawulire aganaafunibwa gayinza okuyamba abantu abalala abalina akawula ka siriimu n’endwadde endala ez’obukaba.

**OKUSASULWA.**

Tewajja kubaawo kusasulira nkyalo zo oba okukebera ebinaakujjibwako mu kunoonyereza kuno.

**OKUKAKASA NTI EBIKUKWATAKO BIJJA KUKUUMIBWA NGA BYA KYAMA**

Ebikukwatako mu kunoonyereza bijja kukuumibwa nga bya kyama, okusinziira ng’amateeka bwe galagira. Ebiwandiiko ebikukwatako mu kunoonyereza kuno bijja kubaako nnamba naye ssi mannya go. Ggwe wamu n’abantu abakola ku kunoonyereza kuno bokka be bajja okumanya nnamba yo. Tewali bikukwatako ggwe ng’omuntu bijja kufulumizibwa nga tetufunye lukusa mu buwandiike okuva gy’oli.

By’otuzzemu bijja kuyingizibwa mu kyuma kikalimagezi (computer) mwe bijja okulambibwa nga tukozesa nnamba yokka era n’ekyuma kino kijja kubaako nnamba gy’oteekamu akakwakkulizo akamanyiddwa abantu abatono ddala mw’oyita okusobola okubituukako (password). By’onotuddamu tebijja kuweerezebwa ku mpewo (Internet) oba mu bbaluwa eziyitibwa e-mail.

Ebiwandiiko ebikakasa nti olina endwadde ez’obukaba wamu n’ebyo ebiraga obujjanjabi bw’edwadde ezo bijja kutwalibwa nga’ekitundu ky’okunoonyereza kuno era bijja kuyisibwa mu ngeri y’emu nga y’akyama ng’ebikujjiddwako ebirala.

Amannya go tegajja kufulumira ku biwandiiko oba ebintu byonna ebifulumizibwa mu kunoonyereza kuno.

Ggwe ng’omuntu tojja kumanyibwa mu binaafulumizibwa mu biwandiiko oba mu nkuaana z’ebikwata mu kunoonyereza kuno.

Ebiwandiiko ebikukwatako biyinza okwekenneenyezebwa ekitongole kya National institute of Allergy and infectious disease, NIH, abalondoola okukakasa enkola ennungi ey’okunoonyereza (monitors), abakola okunoonyereza, Johns Hopkins University, n’ebitongole ebikola ku ddembe ly’abantu abeetaba mu kunoonyereza IRB ne Ethics committee okuva mu America (US) ne Uganda.

**Okulondoola enkola y’okunoonyereza**

Okunoonyereza kujja kuba kulondoolwa aba NIH National Institute of Allergy and infectious diseases oba abo abanaakola ku lw’ekitongole ekyo (Contractors). Bajja kutunuulira nnyo obuzibu obuyinza okubaawo ku bantu abeetabye mu kunoonyereza.

**Ebipya ebinaaba bizuuliddwa**

Ojja kumanyisibwa ebipya ebinaaba bizuuliddwa ng’okunoonyereza kugenda mu maaso ebiyinza okukuleetera okukyusa endowooza yo, oba osigala mu kunoonyereza kuno oba nedda. Okunoonyereza nga kuwedde ojja kutegezebwa ddi ebivudde mu kunoonyereza lwe binaabawo n’engeri gy’oyinza okubifuna/okubimanya.

**Enkola nga bw’eri ku bubenje obukwata ku kunoonyereza (mu kitongole kya Rakai Health sciences program)**

Singa oba okoseddwa olw’okwetabaakwo mu kunoonyereza kuno, ojja kufuna obujjanjabi okuva mu RCCS. Tewali kusasulwa kwonna olw’okukosebwa mu kunoonyereza kuno kw’ofuna okuva mu kitongole kya National Institute of Allergy and Infectious diseases (NIAID), NIH.

**Ebibuuzo n’abantu b’oyinza Okutuukirira:**

Bw’oba olina ebibuuzo byonna bambi nkusaba obuuze, era naffe tuli beetegefu okukola kyonna ekisoboka okubyanukula. Bw’oba ng’olina ebibuuzo ebirala byonna, oba nga weetaaga okukubaganya ebirowoozo ebikwata ku kunoonyereza kuno osobola okutuukirira Dr.Joseph Kagaayi, Dr Godfrey Kigozi, Dr Gertrude Nakigozi abasawo abakulu eb’ebyobulamu, ne Mr Fred Nalugoda alabibirira entambula y’emirimu mu Rakai Program, ku offiisi e Kalisizo(essimu 0772-405861).

Dr Nelson Sewankambo (essimu 041-530020) oba Dr David Serwadda ( essimu 041-545001) abakulira okunoonyereza mu Uganda abasangibwa mu ttendekero ly’abasawo e Makerere University mu Kampala.

Bwoba olina ebibuuzo byonna ebikwata ku ddembe lyo ng’omuntu eyetabye mu kunoonyereza kuno nkusaba otuukirire ssentebe akulira akakiiko akalabirira ebya ssayansi ne ddembe ly’abantu abeetaba mu kunoonyereeza aka UVRI (essimu 041-320385/6).

#### EKIWANDIIKO EKIRAGA OKUKKIRIZA

**Okukiriza kw’omuntu omukulu/Omwana eyerabirira nga tannaweza myaka kkumi na munaana.**

Nze ___________________________________, ow'emyaka ____, nsabiddwa okwetaba mu kunoonyereza okunatwala ebbanga ery’emyaka etaano okukwata ku kugoberera abasajja ebeetaba mu kunoonyereza okukwata ku kukomola abasajja, okwawagirwa NIH okw’okuziyiza akawuka akaleeta siriimu. Abakulu b'okunoonyereza kuno, Dr. Sewankambo/Dr Serwadda oba abasawo ab’ebyobulamu Dr.Kigozi, oba Dr Kagaayi oba Dr Gertrude Nakigozi oba omubaka waabwe annyinnyonnyodde omulamwa gw'okunoonyereza, ebbanga lye kunatwala, okukeberebwa okunaankolebwako, n’obuzibu oba okukosebwamu okuyinza okuntuukako. Nsomeddwa ekiwandiiko kino. Mpeereddwa omukisa okubuuza ebibuuzo ebikwata ku kunoonyereza kuno. Ebibuuzo byonna binziriddwamu mu ngeri gye ntegeera obulungi, era bwe mba nnina ebibuuzo ebirala byonna ebikwata ku kunoonyereza kuno, nsobola okubuuza omubaka akiikiridde pulogulamu, abasawo ab’ebyobulamu abogedwako waggulu, oba Dr. Sewankambo oba Dr. Serwadda.

Ntegeera nti kijja kunneetaagisa okwetaba mu kunoonyereza kwa Rakai Community cohort study, era nina okusoma/okusomerwa era ne nteekako omukono oba ekinkumu kyange kukiwandiiko kya RCCS ekiraga okukkiriza kwange.

Ntegeera nti okwetaba kwange mu kunoonyereza kwa kyeyagalire era nsobola obuteetaba mu kunoonyereza oba okuvaamu obudde bwonna. Ssinga nsalawo obutetaba mu kunoonyereza oba okukuvaamu,tewali kyenja kufiirwa kw’ebyo Rakai Program by’ekola oba by’egaba.

ôôenda kussaako omukono/ekinkumu okulaga nti nzikirizza okwetaba mu kunoonyereza kuno. Nja kuweebwa kkopi y’ekiwandiiko kino ekiraga okukkiriza kwange era nga ntaddeko n'omukono/ekinkumu kyange.

____________________________ ____/_____/______

Omukono/ekinkumu ky’akkirizza Ennaku z’omwezi

____________________________

Erinnya ly’akkirizza

________________________________________________

Endagiriro y’akkirizza

Omukono/ekinkumu okulaga okukkiriza ebijjidwako/ebyajjibwako n’ebiterekebwa okukeberwamu obutundu bw’omubiri obusangibwa mu mubiri gw’omuntu nga yabufuna okuva ku bazadde be (genetic markers)

Nzikirizza;

__________________________ _____/____/_____

Omukono/ekinkumu Ennaku z’omwezi

Sikkirizza;

__________________________ _____/____/_____

Omukono/ekinkumu Ennaku z’omwezi

__________________ ________________ ___/___/_____

Omukono/ekinkumu Erinnya ly’abaddewo Ennaku z’omwezi

ky’abaddewo

__________________ ___/___/_____

Omukono gw’omusawo Ennaku z’omwezi

#### EKIWANDIIKO EKIRAGA OKUKKIRIZA KW’OMUZADDE/AVUNAANYIZIBWA KU MWANA ATANNAWEZA MYAKA 18

Nsabiddwa okukkiriza omwana wange/oba omwana gwe ndabirira okwetaba mu kunoonyereza okunatwala ebbanga ery’emyaka etaano okukwata ku kugoberera abasajja ebeetaba mu kunoonyereza okwawagirwa NIH okukwata ku kukomola okw’okuziyiza akawuka akaleeta siriimu. Abakulu b'okunoonyereza kuno, Dr. Sewankambo/Dr Serwadda oba abasawo ab’ebyobulamu Dr.Kigozi, oba Dr Kagaayi oba Dr Gertrude Nakigozi oba omubaka waabwe annyinnyonnyodde omulamwa gw'okunoonyereza, ebbanga lye kunatwala, okukeberebwa okunaakolebwa ku mwana wange/oba omwana gwe ndabirira, n’obuzibu oba okukosebwamu okuyinza okumutuukako. Ntegeera nti omwana wange/oba omwana gwe ndabirira ajja kwetagibwa okwetaba mu kunoonyereza kwa Rakai Community cohort study, era nina okusoma/okusomerwa era ne ntekako omukono oba ekinkumu kyange ku kiwandiiko kya RCCS ekiraga okukkiriza kwange. Nsomeddwa ekiwandiiko kino. Mpeereddwa omukisa okubuuza ebibuuzo ebikwata ku kunoonyereza kuno. Ebibuuzo byonna binziriddwamu mu ngeri gye ntegeera obulungi, era bwe mba nnina ebibuuzo ebirala byonna ebikwata ku kunoonyereza kuno, nsobola okubuuza abasawo ab’ebyobulamu aboogedwako waggulu, oba Dr. Sewankambo oba Dr. Serwadda.

Ntegeera nti okukkiriza omwana wange/omwana gwe ndabirira okwetaba mu kunoonyereza kwa kyeyagalire era nsobola obutamukkiriza kwetaba mu kunoonyereza oba okuvaamu obudde bwonna era tewali nze oba omwana wange/omwana gwe ndabirira kyetujja kufiirwa kw’ebyo Rakai Program by’ekola oba by’egaba. Era Ntegeera nti omwana waddembe okugaana okwetaba mu byonna oba ebimu ebikwata ku kunoonyereza kuno. ôôenda kussaako omukono/ekinkumu okulaga nti nzikirizza omwana wange/omwana gwe ndabirira okwetaba mu kunoonyereza kuno.

__________________________ _____/____/_____

Omukono/ekinkumu Ennaku z’omwezi

ky’omuzadde

__________________________ ______________________

Erinnya ly’omuzadde Endagiriro y’omuzadde

__________________ ________________ ___/___/_____

Omukono/ekinkumu Erinnya ly’abaddewo Ennaku z’omwezi

ky’abaddewo

__________________ ___/___/_____

Omukono gw’omusawo Ennaku z’omwezi

# APPENDIX 4.

RAKAI COMMUNITY COHORT STUDY (RCCS)

CONSENT FOR ARV & POST-RCT SURVEILLANCE – BASELINE & FOLLOW-UP

***Includes information on studies of circumcision and of ARVs, which are nested in the RCCS.***

**Project Title:** **Rakai Community Cohort Study**

**Principal Investigators:** Drs. Maria J. Wawer and Ronald H. Gray

**Ugandan Principal Investigators:** Drs. Nelson K. Sewankambo and David Serwadda

Hello, my name is ______________. I work for the Rakai Health Sciences Program, a collaboration between the Uganda Ministry of Health through the Uganda Virus Research Institute, Entebbe; and researchers from Makerere University, Kampala; and Columbia University and Johns Hopkins University in the United States. The Rakai Program has worked in Rakai District for over 19 years.

I would like to invite you to participate in a study of the health of people in Rakai and neighboring districts, including a study of the virus that causes AIDS, which is conducted by the Rakai Health Sciences Program. The study is called the Rakai Community Cohort Study (RCCS).

**BACKGROUND**

The acquired immunodeficiency syndrome (AIDS) is a sickness in which the body is unable to protect itself from germs because the Human Immunodeficiency Virus (HIV) has destroyed the body’s protective system, known as the “immune system”. We know that HIV can be spread by having sex with people who are infected with HIV, by sharing needles, by getting an injection with a needle which has not been properly cleaned, or by a mother with HIV passing the virus to her baby during pregnancy or breastfeeding. HIV is a serious health problem in Rakai and neighboring districts. Other infections and conditions, such as TB, malaria and sexually transmitted diseases (STDs) are also common and cause many health problems in Rakai and neighboring districts, and can be particularly serious in persons with HIV. By studying such infections and conditions, including how they are transmitted, and how the body tries to protect itself, we hope to provide information to improve prevention and care of health problems, in the general population and in groups such as pregnant women, infants and children.

**OBJECTIVES AND PLAN OF THE STUDY**

The purpose of the RCCS is to study HIV; other infections such as STDs, malaria and TB; and reproductive health including pregnancy, which affect health in Rakai and neighboring districts. We will also assess behaviors and other factors which may affect reproductive health and the risk of acquiring or transmitting HIV and other infections. The RCCS has been ongoing for over 11 years. We plan to continue the RCCS for at least 4 more years, and to continue enrolling and following all consenting residents aged 15-49 years as well as infants and young children, living in up to 49 communities in Rakai and neighboring districts.

Within the RCCS, there are a number of other, related studies.

1. The Rakai Program conducted a randomized trial of male circumcision for HIV prevention in men. This trial showed that circumcised men had half the rate of new HIV infections compared to uncircumcised men, and the Uganda Ministry of Health and the World Health Organization now recommend circumcision for male HIV prevention, and the Rakai Program is providing circumcision as a service to all men who wish to have the operation. The National Institutes of Health which funded this circumcision trial has agreed to support the long-term evaluation of circumcision on HIV infections and sexual risk behaviors in Rakai. Therefore, we are inviting all men who participated in this trial to enroll into the Rakai Community Cohort Study for this long-term evaluation. With separate support from the Gates Foundation, we are also inviting the wives of these trial participants to also enroll into the Rakai Community Cohort Study.
2. The Rakai Program started a new study to assess the effects of providing HIV antiretroviral (ARV) drugs, the medications which help a person with HIV fight the infection. The study, called “ARV effects on HIV epidemiology and behaviors” will be conducted mainly through the annual surveys of the RCCS. The assessment will include HIV-infected (HIV+) persons whether or not they are on ARVs, as well as HIV-negative people. Study goals include finding out what HIV+ and HIV-negative people know and think about ARVs; whether the availability of ARVs influences behaviors including acceptance of HIV voluntary testing and counseling; whether eligible persons accept ARVs and use them correctly; and what the effects of ARVs are on the spread of HIV, on health in HIV+ persons, and on families. As part of the ARV study, we will also identify pregnant women (both HIV+ and HIV-negative): the pregnant women will be invited to participate in an additional study of mothers’ and infants’ health, called the “ARV-Related Maternal Infant study”. Pregnant women will be provided with additional information and a separate consent for the mother/infant ARMIS study.
3. Rakai Program is continuing to follow men and women who were enrolled into a study to determine whether male circumcision affects the risks of HIV and STDs in men and women. Men living in about 50-60 villages were invited to participate in this circumcision study, which had separate procedures for male consent and enrolment.

However, during the Rakai Community Cohort Study visits, we will ask all men, whether or not they were in the separate circumcision study, if they have been circumcised and when they underwent the procedure, and we will also ask women whether their partners have been circumcised. We will ask everyone about attitudes towards circumcision~~.~~

The data and samples you provide during RCCS will be used to examine whether the rates of HIV, STDs, other infections and conditions, and of behaviors in Rakai and neighboring districts change over time, and factors which may increase or decrease the risks of infections and health problems. We will also see whether there are changes in the rates of HIV, other infections and behaviors as ARVs and male circumcision become available through the Rakai Program or other sources. Since HIV+ persons on ARVs may still be able to transmit HIV, we strongly urge all participants to either abstain or be faithful to one partner who has been HIV tested and is HIV-negative, or to use condoms every time in any other circumstances.

From time to time, the Rakai Health Sciences Program may re-contact RCCS participants who have certain characteristics (for example, age or health status) to invite them to participate in other studies involving procedures such as interviews, focus groups, sample collection or clinical studies. If you are re-contacted, the additional research, will be described to you and you will be asked to provide your consent to participate in the additional research. If you prefer not to participate in the additional study(ies), you will be free to decline. At the end of this consent, you will be asked to sign whether you agree to or decline to be contacted for additional studies.

If you have participated in other Rakai Program studies or services, the data you provide as part of the RCCS study may be linked to the data you provide or have provided in those studies or services.

**PROCEDURES**

The Rakai Community Cohort Study will last at least 4 more years. If you agree to participate in the study, we will visit you in the home approximately once every 12 months. During the visits, we will interview you and ask you questions about yourself such as age and marital status; your health, particularly illnesses you may have had between our visits; your understanding of ways to avoid HIV; and about personal behaviors such as sexual matters. As I explained above, the information will also be used to assess the effects of circumcision and ARVs on behaviors, HIV and STD rates, health, and on families and the community.

We will also ask you to provide a blood sample taken from a vein in your arm. This sample (approximately 15 ml or 1 and ½ tablespoon of blood) will be used to determine whether you have the HIV virus and possibly other infections such as syphilis, other STDs, malaria, and other conditions, to study how they might affect health, and to assess the body’s ability to fight infections and other health conditions. We will also ask you to provide a urine sample in a cup which will be tested for infections, including sexually transmitted diseases. Women will be asked to provide vaginal swabs (which they can collect themselves) and these swabs will be tested for similar infections and conditions. In some survey rounds, you may be requested to provide additional samples for research or so that we can provide you with health services. When additional samples are requested, you will be informed of the type of sample(s) and the reasons for collecting them, and where appropriate, how to access the results of tests. You will be free to agree or decline to provide the additional samples at that time.

We will ask all RCCS participants (men and women) to provide information to help us identify their married or official partners. Such information will help us to assess questions such as factors which might affect the transmission of HIV, other STDs or other infections between people, determine whether a man’s circumcision status affects the health of his female partner overtime (including the risk of acquiring HIV or other STDs), and whether knowledge or use of ARVS affects HIV and STD risks for partners. If we are unsure whether we have identified the correct person, we may ask you to check a photograph of the individual who we think is your partner. The information you provide in your questionnaire, your HIV results, and any STD results will be kept strictly confidential (secret), unless you and your partner both agree to share this information with one another. We strongly encourage all participants who have a sexual partner .to disclose their HIV results to their partner and to receive couples’ counseling, but the Rakai Program will not disclose the survey information you provide or HIV test results to a partner, family member, employers or the community, without direct permission from a participant in writing.

Each interview and sample collection will take approximately 1 hour. You can agree to participate in some or all of the study. Even if you decline to participate in any part of the study, you will not lose your access to Rakai Program clinics or other services.

You will be given a picture identity card (if you have never got one before). The staff will retain a copy of the card used solely for identifying you at each visit.

**RISKS FROM BEING IN THE STUDY**

Potential risks include:

- Some bruising or bleeding at the site of the blood collection. There is also a very low risk of infections. The Rakai Program minimizes these risks through the training it provides to the personnel who collect blood from participants.

- Social consequences if the answers to your questions or your HIV, STDs or other infections or conditions become known. We will minimize this risk by interviewing you in private, and keeping all information and results safe and confidential. All laboratory results will only be revealed to you in private at your request.

**BENEFITS**

The following Rakai Program services are available to all residents in RCCS communities, whether or not they agree to participate in the RCCS:

- HIV prevention and other health education.
- Access to free care, including STD symptom-based care, in Rakai mobile clinics
- Access to free treatment at the Rakai STD clinic in Kalisizo
- Access to free HIV testing in Rakai Suubi clinics, screening for ARV eligibility also offered through the Suubi clinics, and if needed, free ARV treatment and medicines to prevent HIV-related infections (also known as Opportunistic infections or OIs).

The following additional services are available only to RCCS participants:

- The chance for some participants to get home-based HIV results and counseling while others get similar services from our community-based counseling sites.
- Free condoms.
- For HIV+ persons, home-based collection of blood to see if they need to start ARV treatment.
- For all persons who are not pregnant, we will offer home-based oral treatment for worms.

(Women should inform the investigator if they are pregnant.)

- For pregnant women, pregnancy may be confirmed by a urine test and pregnant women will be offered a single dose oral treatment with Azithromycin. These medicines treat infections including gonorrhea, chlamydia and early syphilis, all of which can cause health problems in mothers and babies. Rakai studies have shown these medications improve the health of babies and of mothers.
- Access to free STD treatment in the Rakai Program STD clinic in Kalisizo.

Even if you decide not to participate or to leave the study at a later date, such a decision will not affect your use of the Rakai Program services, described earlier, which are available to the whole community.

**ASSURANCE OF CONFIDENTIALITY**

Research records of your participation in this study will be maintained by the Rakai Program in locked files in either Kalisizo or Entebbe. Only authorized project personnel (approved by the Ugandan Principal Investigators) will have access to these files. All data and medical information collected from you will be kept confidential, to the full extent allowed by the law.

**PARTICIPATION IS VOLUNTARY**

Your participation in this study is completely voluntary. You are free to withdraw at any time or decline to participate in any or all components of the study (interview, sample collection). Such a decision will not affect your medical care by the Rakai Program, now or in the future.

**COMPENSATION**
Men and women in this study will receive 3,000/= UG (approximately $1.50 US) per annual visit as compensation for the time lost as a result of participation in RCCS activities.

STORED SAMPLES

The biological sample you provide in the RCCS study may be used for the tests I described earlier, as well as in other studies such as ARV assessment study. Also, they may also be stored and used for additional research purposes, to learn more about other diseases and conditions, or may be used in new products, tests or discoveries. You will not receive any payment or financial benefit from any products, tests or discoveries. At this time, we do not foresee a specific time when samples from this study will be destroyed. Participation in this extra research is voluntary and your decision will not affect your participation in the current study or your care from the Rakai Program.

Today, or at any time in the future, you may agree to one of the following:

- That your samples, with your identification number attached, can be used for additional future studies.

- That your samples, with your identification number removed, can be used for additional future studies. Please note that if your identification number is removed, it will not be possible for you to have access to any potential future research findings from those samples.

- That your samples cannot be used for any additional research.

If you agree, some of your stored blood provided today or previously may be used to test for body chemicals called genetic markers. These are substances passed on to you from your mother and father. One such substance is called Human Lymphocyte Antigen (HLA) and can influence your ability to fight certain diseases.  In the future, information on genetic markers might help us develop better ways of preventing or treating many conditions, including HIV.

If you decide to participate in this research on genetic markers, you should consider how you might feel if you learned that you and your children might be at increased risk of some disease, especially if there were no treatment. This could make you upset, nervous or sad.

Any genetic information collected or discovered about you and your family will be confidential and will be maintained in a secure manner. If information is shared with other researchers, it will not include your name or your family name, so your confidentiality will be maintained. If in the future we have findings which may have benefits for you or your family, we will discuss the advantages and disadvantages of knowing the information with you, and you can decide whether you want to learn the information and whether you want other family members to also know the information

You can agree or not agree to have your samples tested for genetic markers called HLA. If you agree to these tests there may be no direct benefit to you. However, if you decide not to allow these tests, we will not be able to inform you of possible benefits, should these arise.

**MEDICAL CARE FOR INJURY OR ILLNESS**

The procedures used in the RCCS are safe; we will not be testing any new drugs or medical procedures in the RCCS. Therefore, the risk of illness or harm due to your participation is minimal. However, if you are injured from participating in the study, no money will be available to pay you, but treatment will be available. If you experience any symptoms which you feel may be related to the study, be sure to report them immediately to the persons listed immediately below.

**QUESTIONS/POINTS OF CONTACT**

If you have any questions, please ask, and we will do our best to answer then. If you have additional questions or if you need to discuss any other aspect of the study, you can contact:

Dr. Joseph Kagaayi, Dr. Godfrey Kigozi, and Dr. Gertrude Nakigozi, Medical Officers, and Mr. Fred Nalugoda Director of Field Activities Rakai Program office in Kalisizo (Tel 0772- 405861)

Dr. Nelson Sewankambo or Dr. David Serwadda, Ugandan Principal Investigators, at the Department of Medicine at Makerere University in Kampala (Tel 041-530020) and Institute of Public Health/MUK (tel: 041-545001) respectively.

If there is any portion of this consent explanation sheet that you do not understand, ask me before signing. You will receive a copy of this consent form.

If you have any questions concerning your rights as a participant in this research, please contact the Chairman of Science and Ethics committee of UVRI (tel 041-320385/6).

**STATEMENT OF PARTICIPANT CONSENT**

**CONSENT OF ADULT PARTICIPANT/ ASSENT OF UNEMANCIPATED MINOR**

I ___________________________, age, ______have been asked to participate in a research study named Rakai Cohort Surveillance Study. The principal investigator Dr. Sewankambo/ Dr. Serwadda or the field medical officers Dr. Kigozi, or Dr. Kagaayi or Dr. Gertrude Nakigozi or their representative, ___________________ has explained the study to me, how long it will last, the testing I will undergo, and risks that I might take. The information above has been read to me. I have been given an opportunity to ask questions about this research project. All questions were answered in a way that I understand. If I have other questions about this research, I can ask the study representative, ________________, the field medical officers named above or Dr. Sewankambo, or Dr. Serwadda.

I understand that my participation is voluntary, and that I can decline to be in the study or leave the study at any time. If I decline to participate or if I leave the study, I will not lose any benefits or access to Rakai Program services. I am signing my name below to indicate my consent participate in this project. I will be given a copy of the signed consent form.

_____________________________, _______________

SIGNATURE OF PARTICIPANT DATE

(thumbprint if non-literate)

_____________________________ _____________________________________

PRINTED NAME OF PARTICIPANT SIGNATURE/THUMBPRINT INDICATING CONSENT

FOR USE OF STORED SAMPLES for

additional research with ID #:______

OR: anonymous (without ID#):____________

________________________________ OR: DO NOT CONSENT TO USE OF

ADDRESS OF PARTICIPANT STORED SAMPLES ___________________

Signature/thumbprint indicating consent to be contacted for additional studies through RCCS interview data or lab results:

Agree to be contacted: ________________

Declines to be contacted: ________________

Signature/thumbprint indicating use of stored samples to test for genetic markers.

I agree to have my blood samples tested for genetic markers:

Signature_______________________________________ Date ____________________

I do not agree to have my blood samples tested for genetic markers:

Signature________________________________________ Date_____________________________

________________________ __________________________ ________________________

SIGNATURE OF WITNESS PRINTED NAME OF WITNESS DATE (same as participant’s)

________________________________________ ____________________

SIGNATURE OF INVESTIGATOR ELICITING CONSENT DATE

**CONSENT OF PARENT OR GUARDIAN** ***if participant is an unemancipated minor under age 18.***

I have been asked whether my son/daughter/ward may participate in the Rakai Community Cohort Study. The principal investigator Dr. Sewankambo/ Dr. Serwadda or the field medical officers Dr. Kigozi, or Dr. Kagaayi or Dr. Nakigozi or their representative, ___________________ has explained the study to me, its duration, the testing that my child/ward will be asked to undergo, and possible risks. The information above has been read to me. I have been given an opportunity to ask questions about this research project. All questions were answered in a way that I understand. If I have other questions about this research, I can ask the study representative, ________________ one of the field medical officers named above or Dr. Sewankambo or Dr. Serwadda.

I understand that my agreement to let my child/ward participate is voluntary, and that I can decline to let my child participate or ask that my child leave the study at any time, without losing access to Rakai Program services for myself or my child. I also understand that s/he has the right to voluntarily refuse to participate in all or part of the study. I am signing my name below to indicate my assent for my child/ward to participate in this project.

__________________________________, _______________

SIGNATURE OF PARENT OR GUARDIAN DATE

__________________________________, ____________________________

PRINTED NAME OF PARENT/GUARDIAN ADDRESS OF PARENT/GUARDIAN

__________________________________, _______________

SIGNATURE OF INVESTIGATOR DATE

ELICITING CONSENT

*********************************************************************

**APPENDIX 4A**

**Luganda Translation of RAKAI COMMUNITY COHORT STUDY (RCCS)**

**CONSENT FOR ARV & POST-RCT SURVEILLANCE – BASELINE & FOLLOW-UP**

**APPENDIX 5.**

**CONSENT FOR CIRCUMCISION SURGERY**

# Principal

**Investigators:** Ronald H. Gray, M.D., Johns Hopkins University and

Maria J. Wawer, M.D., Johns Hopkins University

**Ugandan Principal**

**Investigator:** Dr. David Serwadda

You previously consented to be enrolled in the Rakai Program Male Circumcision Trial. As you were previously informed, you were selected by chance to be offered circumcision either immediately or about two years after enrolment.

You previously indicated that you would like to accept circumcision, and today I will explain the procedures, risks and benefits of being circumcised.

# PROCEDURES

The procedures are as follows:

- You will be given the chance to ask any additional questions you may have.
- If you would like to receive a circumcision, you will be asked to sign this consent form allowing the study doctor / clinical officer to examine you and to ask some questions about your health, and if there are no problems, to provide you with a circumcision operation.
- Prior to the circumcision, we would like to collect some specimens. These include samples such as swabs from the outside of your penis and from under the foreskin prior to surgery, blood and urine. These samples will be tested for infections such as HIV and other STDs, and potentially for ways that the body protects itself from infections. We would also like to keep the foreskin that is removed during surgery for future studies, which may include studies of how different parts of the foreskin affect the risk of HIV and other infections. There are no health risks associated with taking the swabs and of preserving the foreskin. If you decline to answer the survey or to provide any or all of the samples, that will not affect your right to receive circumcision from the Rakai Program. I will ask you to sign a section at the end of this form to indicate whether or not you agree to provide samples
- If the health questionnaire or the examination show you may have an STD, you will receive treatment. If you have a serious infection or some other problem with the penis or foreskin, you may also be sent for examination and free treatment by a specialist. You will be asked to wait in order to cure the problem before you are circumcised.
- We will assess if you have enough hemoglobin, the substance that caries oxygen in your blood, for surgery and if you do not have any serious health problem you will be offered circumcision today. If your hemoglobin is low, we will give you iron and vitamin pills to strengthen your blood before you have surgery.
- For the circumcision, your penis and the areas around it will be cleaned.
- The surgeon will inject a drug at the base of the penis to eliminate pain (local anesthetic).
- Then the foreskin will be removed, the wound site sewn up and bandaged. The operation usually takes about 45 minutes
- You will be given pain medicine, and told how to use it.
- You will be required to rest for 30 minutes or more and when you feel OK, you will then be provided with transport back to your home, if needed. If you live far away, you can rest overnight in a Rakai Health Sciences Program dormitory
- You will be seen by a male health worker 1-2 days after surgery, to check on the

wound, change the dressing, if needed, and to check on your use of the pain medicine.

You will be provided with more pain medicine if needed.

- If problems arise after you have gone home, you can contact the Rakai program mobilizer or community counselor, you can also contact us by phone (0772-621515) or come to the clinic. Transport can be arranged if needed in case of circumcision related problems that occur before wound healing is certified.
- You should return to see the health worker 5-9 days after surgery to make sure that the wound is healing properly and 4-6 weeks after surgery to make sure the wound is properly healed**.** A few participants will be asked to return weekly to inspect their wound until it is completely healed using a magnifying glass or other methods that may become available. This will help us to study how long complete wound healing takes and enable us to correctly advise people who accept circumcision as to when to safely resume sex post surgery. At each weekly postoperative visit 5 mL (one teaspoon full) of venous blood will be collected for various tests including determination of the amount of virus in the blood of HIV-infected men (HIV viral load) to assess whether surgery affects the amount of virus in blood.

- Following circumcision, you should refrain from sex until the health worker has certified that the wound is completely healed. This usually takes about 4-6 weeks. This is very important. Starting to have sex before complete wound healing may increase the risk of contracting STD or HIV infection, may result in delayed wound healing, and may cause pain and other problems. The health worker will tell you when the wound looks healed and it is safe to resume sex. You are advised to use condoms when you resume sex.

Please note: We now have evidence that male circumcision reduces the risk of HIV acquisition in HIV negative men. However, this protection is only partial (about 50%), so a circumcised man can still get HIV. Thus, we strongly advise you to always practice safe sex. As you know, safe sex means abstaining from sex, or only having sex with a regular spouse or partner who you know is HIV-uninfected, or using condoms whenever you have sex with a casual partner or someone whose HIV status you do not know.

**RISKS FROM BEING CIRCUMCISED**

Potential risks include:

- risks of surgery for the men who receive circumcision
- risks associated with collection of interview/clinical data and of specimens collected.

*Risks of surgery* include complication such as pain, bleeding, swelling, infections and potentially some numbness.

Discomfort requiring pain medicine may last as long as one week.

Complications are uncommon if circumcision is done properly (in Rakai, around 3-4% of men had problems following surgery that required treatment). We will minimize these risks by having trained doctors, ensuring that all things used for surgery and wound dressing are sterile, and by giving pain medicine. We will follow up participants to ensure proper wound healing, and provide free care or referral to hospitals if needed.

In the past, there have been rare cases of damage to the penis due to an instrument called electrocautery. This is exceptionally uncommon and is avoided by use of a special electrocautery instrument in our theaters.

Rarely, men may get allergic symptoms such as rash, swelling or difficulty in breathing due to the pain medication, the substance used to clean the skin before surgery, or the immunization. We have medicines to treat this type of allergy. The medicines to control pain may make some people drowsy, dizzy and unsteady.

You should not drink alcohol when taking this pain medicine.

Rakai will also provide compensation for time off work, to allow men to stay home for 3 to 4 days after surgery, in order to improve healing.

If you have a serious infection or abnormality of the penis, you will be evaluated and treated by a doctor before surgery. If your condition cannot be treated, you will not be offered circumcision.

*Risks associated with collection of interview/clinical data and samples for testing*:

In the screening consent at the time you enrolled in the study, we described the risks associated with collection of interview data and samples that may occur at any time in this study. Do you have any questions about these risks?

If at any time you feel you have experienced any negative effect from being circumcised, you can contact Dr. D. Serwadda or his representatives (named below).

**COMPENSATION FOR TIME LOST AFTER SURGERY**

Men who are circumcised will receive 6550/= on the day of surgery, 6550/= on the visits 1-2 days after surgery, 6550/= 5-9 days after surgery and 6550/= when they return to check on wound healing 4-6 weeks after surgery for a total of 26,200/= (~$15.00). Men who will be asked to come more frequently will be given 5,300/= (~$3) for each extra visit.

**Alternatives**

You are free to refuse circumcision if you don’t feel like being circumcised.

**COMPENSATION FOR RESEARCH RELATED INJURY**

If you suffer any injury or bad effects due to the surgery, the Rakai Health Sciences Program will provide free treatment, and if necessary we will transport you to a hospital and pay for any hospital costs. No payment for injuries related to the surgery is available through the National Institute of Allergy and Infectious Diseases (NIAID), NIH. The Rakai Health Sciences Program and its collaborating institutions have no program to provide compensation for injury or bad effects which are not due to your participation in the study and which are not the fault of the investigators.

**ASSURANCE OF CONFIDENTIALITY**

All data and medical information will be obtained in private and will be kept confidential, to the full extent allowed by law. Your research/clinical records will receive a unique identification code (number) that is known only to the investigators and will be kept in securely locked files. Only authorized project personnel (approved by the Ugandan Principal Investigators) will have access to these files.

Information relating to you may be shared with other scientists and program developers through presentation or publication, but you will not be identified by name.

**PARTICIPATION IS VOLUNTARY**

Your participation in this service is completely voluntary. You are free to withdraw at any time or decline to participate in any or all components of the service (interview, sample collection) without any penalty or denial of services offered by the Rakai Program.

**QUESTIONS/POINTS OF CONTACT**

If you have any questions, please ask, and we will do our best to answer them. If you have additional questions or if you need to discuss any other aspect of the service, you can contact:

Dr. Noah Kiwanuka, Dr. Godfrey Kigozi, Dr. Kiggundu Valerian or Dr. Joseph Kagayi, Medical Officers, Rakai Program, Kalisizo (tel 0772-621515).

Dr. David Serwadda, Ugandan Principal Investigator, Institute of Public Health, Makerere University, in Kampala (tel 0414-545001) and Uganda Virus Research Institute, Entebbe (0414-321700)

If there is any portion of this consent explanation sheet that you do not understand, ask the clinician before signing. You will receive a copy of this consent form.

If you have any questions concerning your rights as a participant in this service, please contact
the Chairman of Science and Ethics committee of UVRI (tel 0414-320385/6).

**STATEMENT OF VOLUNTEER CONSENT for Surgery, Rakai Male Circumcision Study**

I, , age , have been offered circumcision as a service

I understand that my participation is voluntary and that I can withdraw from the service at any time. I have read or have been read the above and agree to have the circumcision.

The principal investigator, Dr. Serwadda, or the field medical officers, Dr. Kiwanuka,
Dr. Kigozi, Dr. Kiggundu Valerian or Dr. Kagayi, or their representative,
 has explained the service, the testing that I will undergo, the methods to be used, and the risks that I may take.

I have been given an opportunity to ask questions about this service. All questions were answered in a way that I understand. If I have other questions about this service, I can ask the study representative, , one of the field medical offices named above, or Dr. Serwadda.

I have read and understand the information in this statement. I am signing my name below to indicate my consent to circumcision as a service. I understand that I can decide not to be circumcised and I will not lose any benefits which I could otherwise receive from the Rakai Program nor will I receive any penalty. I have been given a copy of the signed consent form.

***CONSENT OF PARENT OR GUARDIAN if participant is an unemancipated minor aged 15-17:***

The service above has been explained to me and the informed consent has been read. I agree to allow the surgery of my son or ward. I also understand that he also has the right to voluntarily refuse to participate in all or part of the service.

SIGNATURE OF PARENT OR GUARDIAN DATE

***************************************************************

____________________________________________________________

SIGNATURE OF PARTICIPANT (thumb print or X if non-literate) DATE

You may agree or disagree to provide the biological samples described in this consent form (blood, swabs and/or foreskin)

Agree to provide sample(s)___________

Disagree to provide sample(s) _________

You may agree to one of the following options with regard to future use of stored samples you provide, but permission will be sought from the Science and Ethics Committee to test the samples for conditions other than those outlined in this form and in the protocol.

Please sign (or fingerprint or X if non-literate) the line, which indicates which option you select.

1. That the samples you provide may be stored and identified by your study number.

2. That the samples you provide may be stored but not identified by your study number.

3. That your samples should not be stored after testing is completed.

ADDRESS OF PARTICIPANT

SIGNATURE OF WITNESS PRINTED NAME OF WITNESS DATE

If participant is non-literate (same as participant's)

SIGNATURE OF CLINICIAN ELICITING CONSENT DATE

(same as participant's)

**APPENDIX 5A**

**Luganda Translation of CONSENT FOR CIRCUMCISION SURGERY**

**APPENDIX 6.**

**RAKAI COMMUNICTY COHORT STUDY (RCCS)**

**ROUND 12 FOLLOW-UP MALE QUESTIONNAIRE Last Edited Jan. 16 2007**

**IDENTIFICATION SHEET FOR MALE FOLLOW-UP QUESTIONNAIRE**

INTERVIEWER # |__|__|__| {FIELDWRKR}

{INT_DATE ddmmyy}

INTERVIEW DATE ____/____/200__

dd mm yy yy

| PLACE  COMPUTER ID HERE |
| --- |

VISIT # **|_R_|_1_|_2**_|{VISITNO}Current ID

Super Cluster # |__|__|__| {REGION}

Community # |__|__|__| {COMM_NUM}

HH # |__|__|__|__| {HH_NUM}

Member # |__|__|__| {MEMBER_NUM}

CIN # |__|__/__|__|__/___|___|___|__/__|__|

**M**

SEX

Names: Usually called__________________________________________ {NAMES memo}

#### Religious names ________________________________________

Other names ___________________________________________

Q.1.How old are you? (**Age in completed years)**  |__|__|{AGEYRS}

**(Valid 15-49)**

RECORD CONFIRMED AGE |__|__| {CONFYRS}

Q.2. Are you enrolled in the Rakai Male Circumcision Trial? |__| {MCENROLLE}

Yes 1

No 2

Registered but not enrolled yet 3

#### CHECK LABEL FOR THE FOLLOWING DETAILS

1. CIRC ID#
2. DATE OF LAST CIRC VISIT
3. DATE OF LAST RCCS VISIT

INTERVIEWER # |__|__|__| {FIELDWRKR)

INTERVIEW DATE {INT_DATE ddmmyyyy)

____/____/200__

dd mm yy yy

| **PLACE**  **COMPUTER ID HERE** |
| --- |

TIME STARTED |__|__| |__|__| am/pm {TSTART}

VISIT # |_R_|_1_|_2_|{VISITNO}

Super Cluster # |__|__|__|{REGION}

Community # |__|__|__|{COMM_NUM}

HH # |__|__|__|__|{HH_NUM}

Member # |__|__|__|{MEMBER_NUM}

#### M

SEX

Q.1. How old are you? (**Age in completed years)**   **|**__|__| {AGEYRS}

**(Valid 15-49)**

RECORD CONFIRMED AGE------------------🡪 |__|__| {CONFYRS}

Q.2 What kinds of work do you do, or what kinds of activities keep you

busy during an average day, whether you get money for them or not?

**[Record answer(s) as given]:** __________________(OCCUP1)

**__________________** (OCCUP2)

**[Code up to two responses, code first mentioned** **occupation first]**

Agriculture for home use/barter 01 |__|__|{OCCUP1}

Agriculture for selling 02 |__|__|{OCCUP2}

Housework in your own home 03

Housekeeper (for relative or employer) 04

Home brewing 05

Government/clerical/teaching 06

Fishing 07

Student 08

Military/police 09

Shopkeeper 10

Trading/vending 11

Bar worker or owner 12

Trucker 13

Unemployed (**PROBE _ NO AGRIC OR HOUSE WORK?**) 14

Other (specify)_____________________ 15

No additional occupation use in 2^nd^ field if one occupation is cited 88

**FOR RESPONDENTS 15-21 YRS ASK Q.2a, ELSE SKIP TO Q.3a**

Q.2a Do you go to school? Yes 1 |__| {SCHOOL}

No 2 ------------> Q.3a

Q.2b What class / level of training are you? P1 - P4 1 |__| {SCHLEV}

P5 - P7 2

S1 - S4 3

S5 - S6 4

Technical/University 5

Primary professional 6

‘O’ level professional 7

Q.3a How many children would you wish to have in your lifetime?

(code actual number, 97 for DK) |__|__|{FERTINTENT}

Q.7a Are you or any of your partners currently using the following family

Planning methods? **[PROMPT]**

Q.7b **IF USING ASK**:For how long have you and/or your partner used

this method consistently without a break?

**[Code completed days, weeks, months or years 8 or 98 for other**

**space in duration coded. Leave duration of other methods** **not**

**currently used blank].**

**(If the person is using a calendar method, code calendar and its duration.**

**Then circle the backup method and code 0 under the duration in days.**

**Then code 8/98 under Week, Month and Year)**

**a) Using b) Duration**

**Yes No DK D W MM YY**

Pills 1 2 7 {FPUSING1} |__| |__| |__|__| |__|__| {FPDUR1}

Condom 1 2 7 {FPUSING2} |__| |__| |__|__| |__|__| {FPDUR2}

Spermicide 1 2 7 {FPUSING3} |__| |__| |__|__| |__|__| {FPDUR3}

Injection 1 2 7 {FPUSING4} |__| |__| |__|__| |__|__| {FPDUR4}

Abstinence 1 2 7 {FPUSING5} |__| |__| |__|__| |__|__| {FPDUR5}

Calendar/rhythm 1 2 7 {FPUSING6} |__| |__| |__|__| |__|__| {FPDUR6}

IUD 1 2 7 {FPUSING7} |__| |__| |__|__| |__|__| {FPDUR7}

Breastfeeding 1 2 7 {FPUSING9} |__| |__| |__|__| |__|__| {FPDUR9}

Herbs/Traditional medicines 1 2 7 {FPUSNG10} |__| |__| |__|__| |__|__| {FPDUR10}

Tubal ligation/vasectomy 1 2 7 {FPUSNG11} |__| |__| |__|__| |__|__| {FPDUR11}

Norplant 1 2 7 {FPUSNG12} |__| |__| |__|__| |__|__| {FPDUR12}

Other Method 1 2 7 {FPUSING8} |__| |__| |__|__| |__|__| {FPDUR8}

Specify __________________________ {OTHFP}

Q.16 Have you or any of your partner(s) ever used a condom for:

**Prompted** **Yes No DK NA (never had sex)**

i) STD/HIV prevention? 1 2 7 8 |__| {EVCONSTD}

ii) Family planning? 1 2 7 8 |__| {EVCONFP}

**IF ‘YES TO 16(i) ASK Q17a, IF NO ASK 17b ELSE SKIP TO Q18a**

Q.17a. Are you or any of your partner(s) currently using condoms

for STD/HIV prevention? |__| {CONDMNOW}

Yes 1-----------🡪18a

No 2

DK/DR 7----------🡪18a

(Has ever had sex but currently no sex partner) NA 8-----------🡪18a

Q.17b. What are the reasons you did not use or are not using condoms for

STD/HIV prevention?

**(Code up to 3 responses)**

Do not know how to put on a condom 01 |__|__| {CURNOCO1}

Want to have a child/children 02 |__|__| {CURNOCO2}

We have both tested negative for HIV 03 |__|__| {CURNOCO3}

Condoms reduce sexual satisfaction 04

Condom use is against our religious beliefs 05

Condom use is against our traditional beliefs 06

I trust my partner (partner is faithful to me) 07

My partner trusts me 08

Do not trust condoms 09

Do not know where to get condoms 10

Cannot afford condoms 11

Don’t feel comfortable getting condoms in our community 12

Condom use not compatible with our sexual style 13

The condoms do not fit properly 14

Partner refused 15

Other (specify)_______________________ 16 {CURNOTHER}

Partner does not have HIV/STD 17

I do not have HIV/STD 18

Q.18a. Have you ever been married or entered a consensual union?

**[If present union is the** **ONLY marriage ever, code ‘Yes’]**

Yes 1 |__|{EVERMARR}

No 2 --------> Q.21a

Q.18b. In your lifetime how many marital/consensual unions have you

had including those that have dissolved? |__|__| {MARORDER}

Q.19. Are you currently married (whether traditional, civil, or religious or in

consensual union)?

###### Yes 1 |__|{CURRMARR}

No 2--------------> Q.21a

Q.20. How many wives do you have? |__|__|{POLYMAR~~}~~

**[Record actual # wherever possible, otherwise code 92= a few (01-02),**

**93=a lot/many (03+), no response= 99]**

Q.21a. Do you currently have a sexual relationship with someone to whom you

are not officially married or in a consensual union?

Yes 1 |__|{CURRLTN}

No 2

DK 7

Q.21b.Do you currently have a mutual non sexual relationship with some one

you are not officially married to/in a consensual union |__|__| {ABSTAIN}

Yes 1

No 2

Q.21c. How long ago did this mutual non sexual relationship start ?

|__|__|{DAYSABST}

|__|__|{WKSABST}

|__|__|{MTHABST}

|__|__|{MTHABST}

|__|__|{YRSABST}

~~Q.22a. Over the past 12 months, have you had a change in your relationships~~

~~with any of your sexual partners? Yes 1 |__|{CHANGEPY}~~

No 2 ----------->Q.23

DK 7----------->Q.23

**Q.22b. If yes**, how has it changed? **[PROBE]** Yes No

Engaged 1 2 {STATPY1}

Married 1 2 {STATPY2}

Ended a non-marital 1 2 {STATPY3}

### Acquired non- marital 1 2 {STATPY4}

Divorced/ separated marital 1 2 {STATPY5}

Widowed 1 2 {STATPY6}

Death of a non- marital partner 1 2 {STATPY7}

Had separated with wife but came back 1 2 {STATPY9}

Had separated with girl friend but came back 1 2 {STATPY10}

Other (specify) 1 2 {OSTATPY}

Specify ________________________ {OTHSTATPY}

Q.23. Have you had sexual intercourse with any person in the last 12 months?

Yes 1 |__| {SEXYEAR}

No 2----------->Q.36 NR 9

Q.31. How many different sexual partners have you had in the last 12 months ,

including married or consensual partners and any other partners? |__|__|{SEXP1YR}

[**Record actual # wherever possible, otherwise**

**code 92= a few (01-02), 93=a lot/many(03+), no response= 99]**

Q.32. How many partners in the last 12 months were from outside your

community?

**[valid codes: Record actual # wherever possible, otherwise**  |__|__|{SEXP1OUT}

**Code 92= a few (01-02), 93=a lot/many (03+), no response= 99]**

Q.33. How long since you last had sexual intercourse?

Valid codes **If < 1 day code 00; < 1 Week code days Days** |__|__|{SEXYRDAY}

**If <1 month code weeks** **Weeks** |__|__|{SEXYRWKS}

**(If DK code 97 in days and 98 in the rest)** **Months**  |__|__|{SEXYRMOS}

**If < 1 year code months**

Q.34. During the past 12 months have you or your partner(s) used condoms?

Yes 1 |__|{CONYR}

No 2----->Q.36

DK 7----->Q.36

NR 9------> Q.36

Q.35. When you have sex, how often did you use condoms, sometimes or always?

Sometimes 1 |__|{CONSISYR}

Always 2

DK 7

Q36. How many different sexual partners have you had in the last five years,

including married or consensual partners and any one already mentioned?

**[valid codes: Record actual # wherever possible, otherwise** |__|__|{SEXP5YR}

**Code 92= a few (01-02), 93=a lot/many (03+), no response= 99]**

Q37. How many different sexual partners have you had in your lifetime, including

married or consensual partners and any one already mentioned?

**[Valid codes: Record actual # wherever possible, otherwise** |__|__|{SEXPEVER}

**Code 92= a few (01-02), 93=a lot/many (03+), no response= 99]**

Q.38a. Do you take alcohol?

Yes 1 |___| {DRINKALC}

No 2------------ >Q.40

Q.38b. When you take alcohol do you usually have the following effects

**(PROMPTED)**

Yes No NR

Unsteady gait 1 2 9 ~~{~~SALCGAIT}

Fall over 1 2 9 {SALCFALL}

Get angry/violent 1 2 9 ~~{~~SALCVIOL}

Difficulty speaking 1 2 9 ~~{~~SALSPEA}

Other (specify) 1 2 9 {SALCOTHE)

Specify _____________________ ~~{~~OTHESALCmemo}

Q.39. In the last 30 days, have you drunk alcohol?

Yes 1 |__| {ALCLSTMOY1}

No 2

NR 9

#### I would like to ask you questions about your penile hygiene

Q.40. Do you wash your penis? **If Yes ask**, How often do you usually wash

your penis? [PROMPT]

Once a month or less 1 |__| [GENBATHY1]

Several times a month 2

Weekly 3

Several times a week 4

Daily 5

More than once a day 6

Don’t wash penis 8-------------------------- > Q.42

Q.41.Why do you wash your penis? **[UNPROMPTED]**

Yes No

Cleanliness 1 2 [WSHREAS1Y1]

Treatment 1 2 [WSHREAS2Y1]

Religion 1 2 [WSHREAS3Y1]

Removing bad odour 1 2 [WSHREAS4Y1]

Other (specify) _____1 2 [OTWSHREAY1]

Q.42**.Do you use any of the following hygiene practices? (Prompted)**

**Yes No Never sex**

Washing genitals before sex 1 2 8 (WSHGBSX)

Washing genitals after sex 1 2 8 (WSHGASX)

Washing genitals before prayer 1 2 8 (WSHGBPRY)

Using a piece of cloth before sex 1 2 8 (WIPEBSX)

Using a piece of cloth after sex 1 2 8 (WIPEASX)

IF NEVER MARRIED OR IN A RELATIONSHIP AND NO SEXUAL

PARTNER IN THE PAST 12 MONTHS (i.e Q.18-37 ARE “NO”/00), THEN

ASK Q.43a, ELSE SKIP TO Q.44a

Q.43. Have you ever had a sexual relationship? Yes 1 |__|__|{EVERSEX}

No 2----------------Q.46C

**EARLY SEXUAL EXPERIENCE: MEN**

Q.44a. Have you ever forced your partner to have sex when she did not want to?

YES 1 |__| {FORCSXTN}

NO 2 ------------ > Skip to Q. 45

## Q.44b. How old were you when this first happened? |__|__| {FORCSXAGE}

**IN RELATIONSHIPS BETWEEN MEN AND WOMEN,**

**DISAGREEMENTS ON SOME ISSUES OCCUR, WHICH**

**SOMETIMES RESULT INTO VIOLENCE.**

**I WOULD LIKE TO ASK YOU SOME QUESTIONS ON VIOLENCE.**

Q.45. In the past 12 months did you do any of the following to your wife /

partner;**PROMPT**

**Yes No NA**

Verbally abuse or shout at her? 1 2 8 {YABUSEYR}

Push her, pull her, slap her or hold her down? 1 2 8 {YPUSHYR}

Punch her with fist or with something that could hurt her? 1 2 8 {YFISTYR}

Kick her or drag her? 1 2 8 {YKICKYR}

Try to strangle her or burn her? 1 2 8 {YBURNYR}

Threaten her with a knife, gun or other weapon? 1 2 8 {YWEAPYR}

Attack her with a knife, gun or other type of weapon? 1 2 8 {YATKNYR}

Other (specify)________________________ 1 2 8 {YOTHRVYR}

Q.46a. In the past 12 months, did you do any of the following

**SEXUAL COERCION**

to your wife/primary partner; **(PROMPT)** **Yes No NA**

Used verbal threats to force her to have sex when she did

not want to? 1 2 8 {YVTHRTFSX}

Physically forced her to have sex when she did not want to? 1 2 8 {YPFOCSEX}

Forced her to perform other sexual acts she did not want to? 1 2 8 {YOTHSXFOC}

**If any yes in question 46a ask 46 b ELSE skip to 46 c**

Q46b When you used force on your partner(s), how often had

you consumed alcohol? |__| {ALCFORCSEX}

Never 1

Occasionally 2

Sometimes 3

Frequently 4

Q.46c Is a man justified in beating his wife/partner if?

Yes No

She neglects household responsibilities 1 2 {BJNEGHH}

She disobeys the instructions of her husband/elders 1 2 {BJDOBEY}

She uses contraception without permission 1 2 {BJFPPRM}

she refuses her husband sex 1 2 {BJREFSEX}

He learns about wife’s/partner’s positive HIV sero status 1 2 {BJPHIVST}

He learns about his positive HIV sero status 1 2 {BJSHIVST}

Argues over money 1 2 {BJARGMON}

Is unfaithful 1 2 {BJUNFTH}

Other reasons (**PROBE**) 1 2 {BOTREAS}

Specify _______________________________ {OTHBEATmemo}

IF NEVER HAD SEX SKIP TO QUESTION 191

**BEGIN REPETITIVE SEXUAL PARTNER BLOCKS**

**The following blocks of questions should be asked for** **each current and**

**past relationship in the last 12 months up to 4 partners. Begin by**

**asking about the most** **recent partner. If the most recent relationship was**

**more** **than a year ago, still ask about this most recent** **partner.**

**"Now I would like to ask you about your most recent sex partner. Please remember that**

**FIRST BLOCK:**

**all of your answers are confidential. Your answers are very important to our research to**

**help us understand health problems in Rakai".**

Q.47.Remembering the most recent time you had sex, what was your relationship

to that partner at that time?

Current wife (at the time) 01 |__|__| {RLTN1}

Current consensual partner( at the time) 02

Former wife/consensual 03

Girlfriend 04

Occasional or casual friend 05

Visitor (incl. wedding/funeral) 06

Stranger 07

Workmate 08

Boss/work supervisor 09

Employee 10

Fellow student 11

Sugar mummy 12

Relative other than spouse (specify)________________ 13 {OTHRLTNR1}

Other non-relative **(specify)________________** 14 {OTHRLNR1}

Don't Know 97

Q.48 What are/were your partner's main occupations?

**(Record answer(s) as given):** **__________________ (OCCUPATION1)**

**__________________ (OCCUPATION2)**

**(Code up to two responses, code first mentioned** **occupation first.**

**If DK or unemployed code 98 in the 2^nd^ box)**

Agricultural for home use/barter 01 |__|__|{OCCUP11}

Agricultural for selling 02 |__|__|{OCCUP21}

Housework in own home 03

Housekeeper (for relative or employer) 04

Home brewing 05

Government/clerical/teaching 06

Fishing 07

Student 08

Military/police 09

Shopkeeper 10

Trading/vending 11

Bar worker or owner 12

Trucker 13

Unemployed (**PROBE _ NO AGRIC OR HOUSE WORK?**) 14

Other ____________________________ 15

No additional response (***use in 2nd field if one occupation is cited***) 88

Do Not Know 97

Q.49. How long did you know this person before you had sex with her for the

first time? [i.e from the time she /you proposed a relationship to first have sex ]

DAYS |__|__| {COTDYSP1}

WEEKS |__|__| {COTWKSP1}

MONTHS |__|__| {COTMOSP1}

YEARS |__|__| {COTYRSP1}

**Less than 1 day code 00 days,Less 1 week code days, Less 1 month code**

**weeks, Less 1 year code months, If 1 year or more code completed years.**

**(If DK Code 97 in days and 98 in other boxes; 99 for NR)**

Q.50. How long ago did you first have sex with this person?

**Less than 1 day code 00, Less than 1 week code days**  |__|__|{DAYS1}

**Less than one month code weeks** |__|__|{WEEKS1}

**Less than one year code months** |__|__|{MONTHS1}

**If 1 year or more code completed years** |__|__|{YEARS1

**(If DK Code 97 in days and 98 in other boxes; 99 for NR**)

Q.51.How long ago, did you last have sex with this person?

**Less than 1 day code 00 days, less than 1 week code |_**_|__|{RLDYSLT1}

**Less than 1 month code weeks** |__|__|{RLWKSLT1}

**Less than 1 year code months** |__|__|{RLMOSLT1}

If 1 year or more code completed years |__|__|{RLYRSLT1}

**(If DK Code 97 in days and 98 in other boxes; 99 for NR)**

Q.52. Are you still in a sexual relationship with her?

Yes 1 |__| {RLTONGO1}

### No 2

### Don't know 7

**During the last time you were having sexual relationship with this partner,**

**please tell me how often you usually had intercourse.**

**FOR QS.53-56 IF DON'T KNOW WRITE DK AND NO RESPONSE**

**WRITE NR BESIDES THE BOX**

Q53 When you had/have sex, how many times per day did/do you usually have

sexual intercourse?**(code # of times per day)** |__| {TIMDAY1}

Q.54. How many days per week did/do you usually have sexual intercourse?

**(code # of days per week)** |__|{DAYSWEK1}

Q.55. How many weeks per month did/do you usually have sexual intercourse?

**(code # of weeks per months)** |__|{WKSMON1}

~~Q56b. How many months in the past 12 months did/have you have/had sexual~~

~~intercourse?~~ **~~(code # of months in the past 12 months)~~**

~~|__|__| {MTSMONY1}~~

**IF THE RELATIONSHIP ENDED MORE THAN 30 DAYS AGO**

**SKIP TO Q.59**.

Q57. In the past seven days how many times did have you had sex with this

person? |__|__| {SFREQWK1}

**[Record actual #, code 97 for DK, 99 for NR]**

Q58. In the past 30 days how many times did you have sex with this person? |__|__| {SFREQMN1}

**[Record actual #, code 97 for DK, 99 for NR]**

Q.59. Is/was she older, younger, or about the same age?

Older 1 |__| {RLTNAGE1}

Younger 2

Same age 3--------------------->Q. 61

DK 7--------------------->Q. 61

Q.60. About how many years [older/younger]? |__|__|{RLTNYRS1}

**(Record actual # or 97=don't know)**

Q.61. Does(was) she live(living) in the same household with you?

|__|{RLTNHH1}

Yes 1---------------------->Q. 64

No 2

Q62. Does (was)she regularly live(living) in your community? |__| {RLTNCM1}

Yes 1----------------------->Q. 64

No 2

### DK 7

Q.63. How far away does/was she normally live/living?

Within one hour’s walk or 3 miles 1 |__| {RLTNRES1}

More than one hour's walk or >3 miles 2

Don't Know 7

Q.64 Have you ever discussed condom use with this partner?

Yes 1 |__| {CNDISCP1}

DR/DK 7 No 2

DK/DR 7

Q.65. Have you and this partner ever used a condom? |__|{CNDEVER1}

Yes 1

No 2------------------------>**Q.**68

NR 9

Q66 During the most recent 12 months you were having sexual relationship

with this partner, how often did you use condoms; never,

sometimes or always? |__|{RNYRCON1}

Never 1**---------------------->Q.68**

Sometimes/inconsistent 2

Always 3

DK 7

Q67a Why did you use condoms with this partner?(UNPROMPTED)

Yes No

To prevent HIV transmission/acquisition/reinfetion 1 2 {CONDHIV1}

To prevent STD acquisition/transmission 1 2 {CONDSTD1}

For family planning 1 2 {CONDFP1}

Just trying it out 1 2 {CONDTRY1}

Other(specify)________________________ 1 2 {CONDOTH1}

Q.67b. Did you use a condom the last time you had sex with that partner?

Yes 1 |__|{RLTNLST1}

No 2

Don't know 7

Q.67c. During the last 30 days you were having a sexual relationship

with this partner, how often did you use condoms?(PROMPTED)

Never 1

Sometimes/Inconsistent 2

Always 3-------------🡪68

DK 4

Q.67d. In the most recent 30 days what are the reasons you did not use/

inconsistently used condoms with this partner?

Do not know how to put on a condom 01 |__|__| {PCURNOCO11}

Want to have a child/children 02 |__|__| {PCURNOCO21}

We have both tested negative for HIV 03 |__|__| {PCURNOCO31}

Condoms reduce sexual satisfaction 04

Condom use is against our religious beliefs 05

Condom use is against our traditional beliefs 06

I trust my partner(partner is faithful) 07

My partner trusts me 08

Do not trust condoms 09

Do not know where to get condoms 10

Cannot afford condoms 11

Don’t feel comfortable getting condoms in our community 12

Condom use not compatible with our sexual style 13

The condoms do not fit properly 14

Partner refused 15

Other _________________________ 16

Partner does not have HIV/STD 17

I do not have HIV/STD 18

Q.68. In the previous 12 months, did this partner have sexual

Relations /intercourse with persons other than yourself?

Yes 1 |__| {RLNSXOT1}

No 2------------------------------>Q. 74a

DK 7------------------------------>Q. 74a

Q.69. **If yes in Q.68;** How many other sexual partners other than

yourself, do you think your partner had sex with in the last 12 months? |__|__|{RLTPNO1}

Q.74a. Do/did you drink alcohol before sex with this partner? |__| {ALCSF1}

Yes 1

No 2 -----------------------------------> Q.76a

Q.74b **IF YES**, is/was it sometimes or always? |__|{ALCSXSF1}

Sometimes/Inconsistent 2

Always/Often 3

DK 7

Q.75. During the times you take/took alcohol and have/had sex with this partner do/

did you usually have the following effects? **(PROMPT)**

Yes No NR

Unsteady gait 1 2 9 {ALUNGSF1}

Fall over 1 2 9 {ALFOSF1}

Get angry/violent 1 2 9 {ALANVSF1}

Difficulty speaking 1 2 9 {ALDSPSF1}

Other 1 2 9 {ALOTHSF1}

Specify ______________ {OTHALCSF1}

Q.76a. Does/did this partner drink alcohol before sex? |__| {ALCBSXPT1

Yes 1

No 2 -------- > Q.79

Q.76b **IF YES**, is/was it sometimes or always? |__|{ALCSXPT1}

Sometimes/Inconsistent 2

Always/Often 3

DK 7

Q.77.When your partner drinks/drank alcohol, and have/had sex with you,

does/did she usually have the following effects **(Prompted )**

Yes No NR

Unsteady gait 1 2 9 {ALCGAPT1}

Fall over 1 2 9 {ALCFAPT1}

Get angry/violent 1 2 9 {ALCVIPT1}

Difficulty speaking 1 2 9 {ALCASPPT1}

Other 1 2 9 {ALOTHPT1}

Specify _____________________ {OTHALCPT1}

Q.79.How likely do you think it is that this partner has been exposed to

HIV (virus which causes AIDS)? (**Prompted**)

Very likely 1 |__| PAIDSRSK1

Somewhat likely 2

Unlikely 3

Not at all 4

Don't Know 7

Q.80. In the last 12 months has this partner informed you of her HIV

sero-status? |__| {INFPHIV1}

Yes 1

No 2

Never tested/got HIV results 3

Received couple counseling 4

DR 7

NA 8

Q.81. In the last 12 months have you informed this partner of your HIV

sero-status? |__| {INFYPHIV1}

Yes 1

No 2

Never tested/got HIV results 3

Received couple counseling 4

DR 7

NA 8

Q.70 In the last 12 months have you informed this

**SECOND BLOCK**

"Can you tell me about the sexual partner just prior to the one we have

just discussed?" **( in the last 12 months).**

Q.82. Have you had any other sexual partners in the last 12 months other

than the one we just discussed? |__| {MORE2}

Yes 1

No 2-----------Q.189

Q.83. Remembering the most recent time you had sex, what was your

relationship to that partner at that time? |__|__| {RLTN2}

Current wife (at the time) 01

Current consensual partner( at the time) 02

Former wife/consensual 03

Girlfriend 04

Occasional or casual friend 05

Visitor (incl. wedding/funeral) 06

Stranger 07

Workmate 08

Boss/work supervisor 09

Employee 10

Fellow student 11

Sugar mummy 12

Relative other than spouse (specify) 13 {OTHRLTR2}

Other non-relative **(specify)________________** 14 {OTHRLNR2}

Don't Know 97

Q.84. What are/were your partner's main occupations?

**(Record answer(s) as given):** **__________________** (OCCUPATION 12)

__________________ (OCCUPATION 22)

**(Code up to two responses, code first mentioned** **occupation first.)**

Agricultural for home use/barter 01 |__|__|{OCCUP12}

Agricultural for selling 02 |__|__|{OCCUP22}

Housework in own home 03

Housekeeper (for relative or employer) 04

Home brewing 05

Government/clerical/teaching 06

Fishing 07

Student 08

Military/police 09

Shopkeeper 10

Trading/vending 11

Bar worker or owner 12

Trucker 13

Unemployed (PROBE _ NO AGRIC OR HOUSE WORK**?**) 14

Other __________________ 15

No additional response (***use in 2nd field if one occupation is cited***) 88

Do Not Know 97

Q.85. How long did you know this person before you had sex with her for the

first time? [i.e from the time she /you proposed a relationship to first have sex ]

days |__|__| {COTDYSP2}

weeks |__|__| {COTWKSP2}

months |__|__| {COTMOSP2}

years |__|__| {COTYRSP2}

**Less than 1 day code 00 days, Less 1 week code days, Less 1 month code**

**weeks, Less 1 year code months, If 1 year or more code completed years.**

**(If DK Code 97 in days and 98 in other boxes; 99 for NR)**

Q.86. How long ago did you first have sex with this person?

**Less than 1 day code 00 days, Less than 1 week code Days** |__|__|{DAYS2}

**Less than one month code weeks** **Weeks** |__|__|{WEEKS2}

**Less than one year code months Months** |__|__|{MONTHS2}

**If one year or more code completed years** **Years** |__|__| {YEARS2}

**(If DK Code 97 in days and 98 in other boxes;99 for NR)**

Q.87. How long ago, did you last have sex with this person?

**Less than 1 day code 00 days, Less than 1 week code Days** |__|__|{RLDYSLT2}

**Less than one month code weeks** **Weeks** |__|__|{RLWKSLT2}

**Less than one year code months Months** |__|__|{RLMOSLT2}

**(If DK Code 97 in days and 98 in other boxes;99 for NR)**

Q.88. Are you still in a sexual relationship with her?

Yes 1 |__| {RLTONGO2}

### No 2

### Don't know 7

**During the last time you were having sexual relationship with this partner,**

**please tell me how often you usually had intercourse.**

**FOR Qns.89-92 IF DON'T KNOW WRITE DK AND NO RESPONSE**

**WRITE NR BESIDES THE BOX**

Q.89. When you had/have sex, how many times per day did/do you usually have

sexual intercourse?**(code # of times per day)** |__| {TIMDAY2}

Q.90. How many days per week did/do you usually have sexual intercourse?

**(code # of days per week)** |__|{DAYSWEK2}

Q.91. How many weeks per month did/do you usually have sexual intercourse?

**(code # of weeks per months)** |__|{WKSMON2}

~~Q.92. How many months in the past 12 months did/have you have/had sexual~~

~~intercourse?~~ **~~(code # of months in the past 12 months)~~**  ~~|__|__| {MTSMON2Y1}~~

**IF THE RELATIONSHIP ENDED MORE THAN 30 DAYS AGO**

**SKIP TO Q.95**.

Q.93. In the past seven days how many times did you have sex with this person? |__|__| {SFREQWK2}

**[Record actual #, code 97 for DK, 99 for NR]**

Q.94. In the past 30 days how many times did you have sex with this person? |__|__| {SFREQMN2}

**[Record actual #, code 97 for DK, 99 for NR]**

Q95. Is/was she older, younger, or about the same age?

Older 1 |__|RLTNAGE2}

Younger 2

Same age 3-------------------------->**Q. 97**

DK 7-------------------------->Q. 97

Q.96. About how many years [older/younger] ? |__|__|{RLTNYRS2} **(Record actual # or 97=don't know)**

Q.97. Does(was) she live(living) in the same household with you?

|__|{RLTNHH2}

Yes 1------>**Q.100**

No 2

Q.98. Does(was)she regularly live(living) in your community? |__| {RLTNCM2}

Yes 1------- >**Q. 100**

No 2

### DK 7

Q.99. How far away does/was she normally live/living?

Within one hour’s walk or 3 miles 1 |__| {RLTNRES2}

More than one hour's walk or >3 miles 2

Don't Know 7

Q.100. Have you ever discussed condom use with this partner?

Yes 1 |__| {CNDISCP2}

DR/DK 7 No 2

DK/DR 7

Q.101. Have you and this partner ever used a condom? |__|{CNDEVER2Y1}

Yes 1

No 2------------------------->**Q.**104

NR 9

Q102. During the most recent 12 months you were having sexual relationship

With this partner, how often did you use condoms; never,

sometimes or always?

|__|{RNYRCON2}

Never 1**-------------------------->Q.104**

Sometimes/inconsistent 2

Always 3

DK 7

Q103.a.Why did you use condoms with this partner?(UNPROMPTED)

Yes No

To prevent HIV transmission/acquisition/reinfetion 1 2 {CONDHIV2}

To prevent STD acquisition/transmission 1 2 {CONDSTD2}

For family planning 1 2 {CONDFP2}

Just trying it out 1 2 {CONDTRY2}

Other ___________________________ 1 2 {CONDOTH2}

Q.103b. Did you use a condom the last time you had sex with that partner?

Yes 1 |__|{RLTNLST2}

No 2

Don't know 7

Q.103c. During the last 30 days you were having a sexual relationship

with this partner, how often did you use condoms?(PROMPTED)

Never 1

Sometimes/Inconsistent 2

Always 3-------------🡪104

DK 4

Q.103d. In the most recent 30 days what are the reasons you did not use/

inconsistently used condoms with this partner?

Do not know how to put on a condom 01 |__|__| {PCURNOCO12}

Want to have a child/children 02 |__|__| {PCURNOCO22}

We have both tested negative for HIV 03 |__|__| {PCURNOCO32}

Condoms reduce sexual satisfaction 04

Condom use is against our religious beliefs 05

Condom use is against our traditional beliefs 06

I trust my partner(partner faithful) 07

My partner trusts me 08

Do not trust condoms 09

Do not know where to get condoms 10

Cannot afford condoms 11

Don’t feel comfortable getting condoms in our community 12

Condom use not compatible with our sexual style 13

The condoms do not fit properly 14

Partner refused 15

Other _________________________ 16

Partner does not have HIV/STD 17

I do not have HIV/STD 18

Q.104. In the previous 12 months, did this partner have sexual

Relations /intercourse with persons other than yourself?

Yes 1 |__| {RLNSXOT2}

No 2------------------------------>Q. 110a

DK 7------------------------------>Q. 110a

Q.105. **If yes in Q.104.** How many other sexual partners other than

yourself, do you think your partner had sex with in the last12 months? |__|__|{RLTPNO2}

Q.110a. Do/did you drink alcohol before sex with this partner? |__| (ALCSF2)

Yes 1

No 2 --------------- > Q.112a

Q.110b **IF YES**, is/was it sometimes or always? |__|{ALCSXSF2}

Sometimes/Inconsistent 2

Always/Often 3

DK 7

Q.111. During the times you take/took alcohol and have/had sex with this partner do/did

you usually have the following effects? (PROMPT)

Yes No NR

Unsteady gait 1 2 9 {ALUNGSF2}

Fall over 1 2 9 {ALFOSF2}

Get angry/violent 1 2 9 {ALANVSF2}

Difficulty speaking 1 2 9 {ALDSPSF2}

Other 1 2 9 {ALOTHSF2}

(Specify ) ______________ {OTHALCSF2}

Q.112a. Does/did this partner drink alcohol before sex? |__|{ALCBSXPT2}

Yes 1

No 2 ---------------------- > Q.115

Q.112b **IF YES**, is/was it sometimes or always? |__|{ALCSXPT2}

Sometimes/Inconsistent 2

Always/Often 3

DK 7

Q.113. When your partner drinks/drank alcohol, and have/had sex with you ,does/did

she usually have the following effects **(Prompted )**

Yes No NR

Unsteady gait 1 2 9 {ALCGAPT2}

Fall over 1 2 9 {ALCFAPT2}

Get angry/violent 1 2 9 {ALCVIPT2}

Difficulty speaking 1 2 9 {ALCASPPT2}

Other 1 2 9 {ALOTHPT2}

Specify _____________________ {OTHALCPT2}

Q.115. How likely do you think it is that this partner has been exposed to HIV (virus

which causes AIDS)? (**Prompted**)

Very likely 1 |__|{PAIDSRSK2}

Somewhat likely 2

Unlikely 3

Not at all 4

Don't Know 7

Q.116. In the last 12 months has this partner informed you of her HIV sero-status?

Yes 1 |__|{INFPHIV2}

No 2

Never tested/got HIV results 3

Received couple counseling 4

DR 7

Q.117. In the last 12 months have you informed this partner of your HIV

sero-status? Yes 1 |__|{ INFYPHIV2}

No 2

Never tested/got HIV results 3

Received couple counseling 4

DR 7

**THIRD BLOCK**

"**Can you tell me about the sexual partner just prior to the one we have just**

**discussed?"( in the last 12 months).**

Q.118. Have you had any other sexual partners in the last 12 months other than

the one we just discussed? **|__|** {MORE3}

Yes 1

No 2-----------Q.189

Q.119 Remembering the most recent time you had sex, what was your

relationship to that partner at that time?

Current wife (at the time) 01 |__|__| {RLTN3}

Current consensual partner( at the time) 02

Former wife/consensual 03

Girlfriend 04

Occasional or casual friend 05

Visitor (incl. wedding/funeral) 06

Stranger 07

Workmate 08

Boss/work supervisor 09

Employee 10

Fellow student 11

Sugar mummy 12

Relative other than spouse (specify)________________ 13 {OTHRLTR3}

Other non-relative **(specify)________________** 14 {OTHRLNR3}

Don't Know 97

Q.120. What are/were your partner's main occupations?

**(Record answer(s) as given):** **__________________ (OCCUPATION13)**

**__________________ (OCCUPATION23)**

**(Code up to two responses, code first mentioned** **occupation first.**

Agricultural for home use/barter 01 |__|__|{OCCUP13}

Agricultural for selling 02 |__|__|{OCCUP23}

Housework in own home 03

Housekeeper (for relative or employer) 04

Home brewing 05

Government/clerical/teaching 06

Fishing 07

Student 08

Military/police 09

Shopkeeper 10

Trading/vending 11

Bar worker or owner 12

Trucker 13

Unemployed (**PROBE _ NO AGRIC OR HOUSE WORK?**) 14

Other ____________________________ 15

No additional response (***use in 2nd field if one occupation is cited***) 88

Do Not Know 97

Q.121. How long did you know this person before you had sex with her for

the first time? [i.e from the time she /you proposed a relationship to

first have sex ]

**Less than 1 day code 00 days,Less 1 week code days** Days |__|__|{COTDYSP3}

**Less 1 month code weeks** Weeks |__|__|{COTWKSP3}

**Less 1 year code months** Months |__|__|{COTMOSP3}

**If 1 year or more code completed years** Years |__|__|{COTYRSP3}

**(If DK Code 97 in days and 98 in other boxes; 99 for NR)**

Q.122. How long ago did you first have sex with this person?

**Less than 1 day code 00 days,Less 1 week code days** Days |__|__|{DAYS3}

**Less 1 month code weeks** Weeks |__|__|{WEEKS3}

**Less 1 year code months** Months |__|__|{MONTHS3}

**If 1 year or more code completed years** Years |__|__|{YEARS3}

**(If DK Code 97 in days and 98 in other boxes; 99 for NR)**

Q.123. How long ago, did you last have sex with this person?

**Less than 1 day code 00 days,Less 1 week code days** Days |__|__|{ RLDYSLT3}

**Less 1 month code weeks** Weeks |__|__|{ RLWKSLT3}

**Less 1 year code months** Months |__|__|{ RLMOSLT3}

Q.124. Are you still in a sexual relationship with her?

Yes 1 |__| {RLTONGO3}

### No 2

### Don't know 7

**During the last time you were having sexual relationship with this partner,**

**please tell me how often you usually had intercourse.**

**FOR QS 125-128 IF DON'T KNOW WRITE DK AND NO RESPONSE**

**WRITE NR BESIDES THE BOX**

Q.125. When you had/have sex, how many times per day did/do you usually have

sexual intercourse?**(code # of times per day)** |__| {TIMDAY3}

Q.126 How many days per week did/do you usually have sexual intercourse?

**(code # of days per week)** |__|{DAYSWEK3}

Q.127 How many weeks per month did/do you usually have sexual intercourse?

**(code # of weeks per months)** |__|{WKSMON3}

~~Q.128b How many months in the past 12 months did/have you have/had sexual~~

~~intercourse? (~~**~~code # of months in the past 12 months~~**~~)~~

~~|__|__| {MTSMON3Y1}~~

**IF THE RELATIONSHIP ENDED MORE THAN 30 DAYS AGO**

**SKIP TO Q.131**

Q.129. In the past seven days how many times did have you had sex with this

person?**[Record actual #, code 97 for DK, 99 for NR]** |__|__| ~~{~~SFREQWK3}

Q.130. In the past 30 days how many times did you have sex with this person?

**[Record actual #, code 97 for DK, 99 for NR]** |__|__| {SFREQMN3}

Q.131. Is/was she older, younger, or about the same age ?

Older 1 |__| {RLTNAGE3}

Younger 2

Same age 3------------------------- >Q. 133

DK 7------------------------- >Q. 133

Q.132. About how many years [older/younger] ? |__|__|{RLTNYRS3}

**(Record actual # or 97=don't know)**

Q.133. Does(was) she live(living) in the same household with you? |__|{RLTNHH3}

Yes 1----------------------->Q. 136

No 2

Q.134. Does(was)she regularly live(living) in your community? |__| {RLTNCM3}

Yes 1--------------------->Q. 136

No 2

### DK 7

Q.135. How far away does/was she normally live/living?

Within one hour’s walk or 3 miles 1 |__| {RLTNRES3Y1}

More than one hour's walk or >3 miles 2

Don't Know 7

Q.136. Have you ever discussed condom use with this partner?

Yes 1 |__| {CNDISCP3}

DR/DK 7 No 2

DK/DR 7

Q.137 Have you and this partner ever used a condom? |__|{CNDEVER3Y1}

Yes 1

No 2----------------------->**Q.**140

NR 9

Q.138. During the most recent 12 months you were having sexual relationship

with this partner, how often did you use condoms; never,

sometimes or always? |__|{RNYRCON3}

Never 1**--------------------->Q.140**

Sometimes/inconsistent 2

Always 3

DK 7

Q139.a.Why did you use condoms with this partner?(UNPROMPTED)

Yes No

To prevent HIV transmission/acquisition/reinfetion 1 2 {CONDHIV3}

To prevent STD acquisition/transmission 1 2 {CONDSTD3}

For family planning 1 2 {CONDFP3}

Just trying it out 1 2 {CONDTRY3}

Other ___________________________ 1 2 {CONDOTH3}

Q.139b. Did you use a condom the last time you had sex with that partner?

Yes 1 |__|{RLTNLST3}

No 2

Don't know 7

Q.139c. During the last 30 days you were having a sexual relationship

with this partner, how often did you use condoms?(PROMPTED)

Never 1

Sometimes/Inconsistent 2

Always 3-------------🡪140

DK 4

Q.139d. In the most recent 30 days what are the reasons you did not use/

inconsistently used condoms with this partner?

Do not know how to put on a condom 01 |__|__| {PCURNOCO13}

Want to have a child/children 02 |__|__| {PCURNOCO23}

We have both tested negative for HIV 03 |__|__| {PCURNOCO33}

Condoms reduce sexual satisfaction 04

Condom use is against our religious beliefs 05

Condom use is against our traditional beliefs 06

I trust my partner(partner faithful) 07

My partner trusts me 08

Do not trust condoms 09

Do not know where to get condoms 10

Cannot afford condoms 11

Don’t feel comfortable getting condoms in our community 12

Condom use not compatible with our sexual style 13

The condoms do not fit properly 14

Partner refused 15

Other _________________________ 16

Partner does not have HIV/STD 17

I do not have HIV/STD 18

Q.140. In the previous 12 months, did this partner have sexual

Relations /intercourse with persons other than yourself?

Yes 1 |__| {RLNSXOT3}

No 2------------------------------->Q. 146a

DK 7------------------------------->Q. 146a

Q.141. **If yes in Q.140;** How many other sexual partners other than

yourself, do you think your partner had sex with in the last 12 months?

|__|__|{RLTPNO3}

Q.146a. Do/did you drink alcohol before sex with this partner? |__| {ALCSF3}

Yes 1

No 2 --------------------- > Q.147a

Q.146b **IF YES**, is/was it sometimes or always? |__|{ALCSXSF3}

Sometimes/Inconsistent 2

Always/Often 3

DK 7

Q.146c . During the times you take/took alcohol and have/had sex with this partner,

do/did you usually have the following effects? (PROMPT)

Yes No NR

Unsteady gait 1 2 9 {ALUNGSF3}

Fall over 1 2 9 {ALFOSF3}

Get angry/violent 1 2 9 {ALANVSF3}

Difficulty speaking 1 2 9 {ALDSPSF3}

Other 1 2 9 {ALOTHSF3}

(Specify ) ______________ {OTHALCSF3}

Q.147a. Does/did this partner drink alcohol before sex? |__| {ALCBSXPT3}

Yes 1

No 2 --------------- > Q.150

Q.147b **IF YES**, is/was it sometimes or always? |__|{ALCSXPT3}

Sometimes/Inconsistent 2

Always/Often 3

DK 7

Q148.When your partner drinks/drank alcohol, and have/had sex with you

does/did she usually have the following effects **(Prompted )**

Yes No NR

Unsteady gait 1 2 9 {ALCGAPT3}

Fall over 1 2 9 {ALCFAPT3}

Get angry/violent 1 2 9 {ALCVIPT3}

Difficulty speaking 1 2 9 {ALCASPPT3}

Other 1 2 9 {ALOTHPT3}

Specify _____________________ {OTHALCPT3}

Q.150. How likely do you think it is that this partner has been exposed to HIV

(virus which causes AIDS)? (**prompted**)

Very likely 1 |__|}{PAIDSRSK3}

Somewhat likely 2

Unlikely 3

Not at all 4

Don't Know 7

Q.151. In the last 12 months has this partner informed you of her HIV sero-

status?

Yes 1 |__| {INFPHIV3}

No 2

Never tested/got HIV results 3

Received couple counseling 4

DR 7

Q.152. In the last 12 months have you informed this partner of your HIV sero-

status?

Yes 1 |__| {INFYPHIV3}

No 2

Never tested/got HIV results 3

Received couple counseling 4

DR 7

**FOURTH BLOCK**

"Can you tell me about the sexual partner just prior to the one we have just

discussed?"**( in the last 12 months).**

Q.153. Have you had any other sexual partners in the last 12 months other

than the one we just discussed? |__|{MORE4}

Yes 1

No 2-----------Q.189

Q.154. Remembering the most recent time you had sex, what was your

relationship to that partner at that time?

Current wife (at the time) 01 |__|__| {RLTN4}

Current consensual partner( at the time) 02

Former wife/consensual 03

Girlfriend 04

Occasional or casual friend 05

Visitor (incl. wedding/funeral) 06

Stranger 07

Workmate 08

Boss/work supervisor 09

Employee 10

Fellow student 11

Sugar mummy 12

Relative other than spouse (specify) 13 OTHRLTR4}

Other non-relative **(specify)________________** 14 {OTHRLNR4

Don't Know 97

Q.155. What are/were your partner's main occupations?

**(Record answer(s) as given):** **__________________ (OCCUPATION 14)**

**__________________ (OCCUPATION 24)**

**(Code up to two responses, code first mentioned** **occupation first.**

**If DK or unemployed code 98 in the 2^nd^ box)**

Agricultural for home use/barter 01 |__|__|{OCCUP14~~}~~

Agricultural for selling 02 |__|__|{OCCUP24}

Housework in own home 03

Housekeeper (for relative or employer) 04

Home brewing 05

Government/clerical/teaching 06

Fishing 07

Student 08

Military/police 09

Shopkeeper 10

Trading/vending 11

Bar worker or owner 12

Trucker 13

Unemployed (**PROBE _ NO AGRIC OR HOUSE WORK?**) 14

Other ____________________________ 15

No additional response (***use in 2nd field if one occupation is cited***) 88

Do Not Know 97

Q.156. How long did you know this person before you had sex with her for

the first time?[i.e from the time she /you proposed a relationship to first

have sex ]

**Less than 1 day code 00 days,Less 1 week code days** Days |__|__|{ COTDYSP4}

**Less 1 month code weeks** Weeks |__|__|{ COTWKSP4}

**Less 1 year code months** Months |__|__|{COTMOSP4}

**If 1 year or more code completed years** Years |__|__|{COTYRSP4}

**(If DK Code 97 in days and 98 in other boxes; 99 for NR)**

Q.157. How long ago did you first have sex with this person?

**Less than 1 day code 00 days,Less 1 week code days** Days |__|__|{DAYS4}

**Less 1 month code weeks** Weeks |__|__|{WEEKS4}

**Less 1 year code months** Months |__|__|{MONTHS4}

**If 1 year or more code completed years** Years |__|__|{YEARS4}

**(If DK Code 97 in days and 98 in other boxes; 99 for NR)**

Q.158. How long ago, did you last have sex with this person?

**Less than 1 day code 00 days,Less 1 week code days** Days |__|__|{RLDYSLT4}

**Less 1 month code weeks** Weeks |__|__|{RLWKSLT4}

**Less 1 year code months** Months |__|__|{RLMOSLT4}

Q.159. Are you still in a sexual relationship with her?

Yes 1 |__| {RLTONGO4}

### No 2

### Don't know 7

**During the last time you were having sexual relationship with this partner,**

**please tell me how often you usually had intercourse.**

**FOR QS.160-163 IF DON'T KNOW WRITE DK AND NO RESPONSE**

**WRITE NR BESIDES THE BOX**

Q.160. When you had/have sex, how many times per day did/do you usually

have sexual intercourse?**(code # of times per day)** |__| {TIMDAY4}

Q.161 How many days per week did/do you usually have sexual intercourse?

**(code # of days per week)** |__|{DAYSWEK4}

Q.162. How many weeks per month did/do you usually have sexual intercourse?

**(code # of weeks per months)** |__|{WKSMON4}

~~Q163b. How many months in the past 12 months did/have you have/had sexual~~

~~intercourse?~~**~~(code # of months in the past 12 months)~~**  ~~|__|__| {MTSMON4Y1}~~

**IF THE RELATIONSHIP ENDED MORE THAN 30 DAYS AGO**

**SKIP TO Q.166**.

Q164 In the past seven days how many times did you have sex with

this person?

**[Record actual #, code 97 for DK, 99 for NR]** |__|__| {SFREQWK4}

Q.165 In the past 30 days how many times did you have sex with this

person?

**[Record actual #, code 97 for DK, 99 for NR]** |__|__|{SFREQMN4}

Q.166 Is/was she older, younger, or about the same age ?

Older 1 |__| {RLTNAGE4}

Younger 2

Same age 3---------------- >**Q. 168**

DK 7---------------- >Q. 168

Q.167. About how many years [older/younger] ? |__|__|{RLTNYRS4}

**(Record actual # or 97=don't know)**

Q.168. Does(was) she live(living) in the same household with you?

|__|{RLTNHH4}

Yes 1---------------->Q.171

No 2

Q.169. Does(was)she regularly live(living) in your community? |__| {RLTNCM4}

Yes 1--------------- >Q. 171

No 2

### DK 7

Q.170. How far away does/was she normally live/living?

Within one hour’s walk or 3 miles 1 |__| {RLTNRES4}

More than one hour's walk or >3 miles 2

Don't Know 7

Q.171. Have you ever discussed condom use with this partner?

Yes 1 |__| {CNDISCP4}

DR/DK 7 No 2

DK/DR 7

Q.172. Have you and this partner ever used a condom? |__|{CNDEVER4}

Yes 1

No 2------------------->**Q.**175

NR 9

Q.173. During most recent 12 months you were having sexual relationship

with this partner, how often did you use condoms; never,

sometimes or always? |__|{RNYRCON4}

Never 1**------------------------>Q.175**

Sometimes/inconsistent 2

Always 3

DK 7

Q174a.Why did you use condoms with this partner?(UNPROMPTED)

Yes No

To prevent HIV transmission/acquisition/reinfetion 1 2 {CONDHIV4}

To prevent STD acquisition/transmission 1 2 {CONDSTD4}

For family planning 1 2 {CONDFP4}

Just trying it out 1 2 {CONDTRY4}

Other ___________________________ 1 2 {CONDOTH4}

Q.174b. Did you use a condom the last time you had sex with that partner?

Yes 1 |__|{RLTNLST4}

No 2

Don't know 7

Q.174c. During the last 30 days you were having a sexual relationship

with this partner, how often did you use condoms?(PROMPTED)

Never 1

Sometimes/Inconsistent 2

Always 3-------------🡪175

DK 4

Q.174d. In the most recent 30 days what are the reasons you did not use/

inconsistently used condoms with this partner?

Do not know how to put on a condom 01 |__|__| {PCURNOCO14}

Want to have a child/children 02 |__|__| {PCURNOCO24}

We have both tested negative for HIV 03 |__|__| {PCURNOCO34}

Condoms reduce sexual satisfaction 04

Condom use is against our religious beliefs 05

Condom use is against our traditional beliefs 06

I trust my partner(partner faithful) 07

My partner trusts me 08

Do not trust condoms 09

Do not know where to get condoms 10

Cannot afford condoms 11

Don’t feel comfortable getting condoms in our community 12

Condom use not compatible with our sexual style 13

The condoms do not fit properly 14

Partner refused 15

Other _________________________ 16

Partner does not have HIV/STD 17

I do not have HIV/STD 18

Q.175. In the previous 12 months, did this partner have sexual

relations /intercourse with persons other than yourself? |__| {RLNSXOT4}

Yes 1

No 2------->Q. 181a

DK 7------->Q. 181b

Q.176.**If yes in Q.175;** How many other sexual partners other than

yourself, do you think your partner had sex with in the last 12 months?

|__|__|{RLTPNO4}

Q.181a. Do/did you drink alcohol before sex with this partner? |__| {ALCSF4}

Yes 1

No 2 ------------------------------------- > Q.182a

Q.181b **IF YES**, is/was it sometimes or always? |__|{ALCSXSF4}

Sometimes/Inconsistent 2

Always/Often 3

DK 7

Q.181c. During the time you took alcohol and have/had sex with this partner

do/did you usually have the following effects? (PROMPT)

Yes No NR

Unsteady gait 1 2 9 {ALUNGSF4}

Fall over 1 2 9 {ALFOSF4}

Get angry/violent 1 2 9 {ALANVSF4}

Difficulty speaking 1 2 9 {ALDSPSF4}

Other 1 2 9 {ALOTHSF4}

(Specify ) ______________ {OTHALCSF4}

Q.182a. Does/did this partner drink alcohol before sex? |__| {ALCBSXPT4}

Yes 1

No 2 -------------------------------------------- > Q.185

Q.182b **IF YES**, is/was it sometimes or always? |__|{ALCSXPT4}

Sometimes/Inconsistent 2

Always/Often 3

DK 7

Q.183. When your partner drinks/drank alcohol, and has/had sex with

you does/did she usually have the following effects **(Prompted )**

Yes No NR

Unsteady gait 1 2 9 {ALCGAPT4}

Fall over 1 2 9 {ALCFAPT4}

Get angry/violent 1 2 9 {ALCVIPT4}

Difficulty speaking 1 2 9 {ALCASPPT4}

Other 1 2 9 {ALOTHPT4}

Specify _____________________ {OTHALCPT4}

Q.185. How likely do you think it is that this partner has been exposed to

HIV (virus which causes AIDS)? (**Prompted**)

Very likely 1 |__| PAIDSRSK4

Somewhat likely 2

Unlikely 3

Not at all 4

Don't Know 7

Q.186. In the last 12 months has this partner informed you of her HIV

sero-status?

Yes 1 |__|{INFPHIV4}

No 2

Never tested/got HIV results 3

Received couple counseling 4

DR 7

Q.187. In the last 12 months have you informed this partner of your HIV

sero-status?

Yes 1 |__| {INFYPHIV4}

No 2

Never tested/got HIV results 3

Received couple counseling 4

DR 7

**END OF REPETITIVE SEXUAL BLOCKS**

**SKIP TO Q.191 IF HAD NO SEX IN THE LAST 12 MONTHS**

Q.189. In the last 12 months, have you ever had sex in a situation in which

money or gifts were exchanged?

Yes 1 |__|{SEXGIFT~~}~~

No 2

Q.191. In the last 12 months have you had any of the following health problems?

**PROMPTED:**  (Code in since **last 12 months column** )

Do you have these symptoms now or have you had them over the past 7

days (Code in current Column) **For GUD as**k: How many episodes in the past

12 month

**Last 12 months**  Current# of episodes

**Yes No DK Yes No DK**

Genital ulcer 1 2 7 {SXM1Y1} 1 2 7 {CUR1Y1} |__|__| {GUDPY}

Genital discharge 1 2 7 {SXM2Y1} 1 2 7 {CUR2Y1}

Frequent urination 1 2 7 {SXY1Y1} 1 2 7 {CUR6Y1}

Painful urination 1 2 7 {SXM7Y1} 1 2 7 {CUR7Y1}

pain during intercourse 1 2 7 {SXM8Y1} 1 2 7 {CUR8Y1}

bleeding during intercourse 1 2 7 {SXM9Y1} 1 2 7{CUR9Y1}

lower abdominal pain 1 2 7 {SXM10Y1} 1 2 7{CUR10Y1}

Genital warts 1 2 7 {SXM11Y1} 1 2 7{CUR11Y1}

**IF NO TO ALL IN Q.191 SKIP TO Q.199a**

**IF RESPONDENT REPORTED STD SYMPTOM EITHER CURRENTLY**

**(last 7 days)** **OR IN THE LAST 12 MONTHS, ASK DURATION FOR** **THE**

**MOST RECENTLY HEALED EPISODE:**  **IF RESPONDENT HAD ONLY**

**ONE OF THE FOLLOWING AILMENTS**

**CODE 98 FOR THE REST. Also code 98 in all if symptoms reported are**

**neither GUD, discharge nor painful urination**

Q.192 **For respondents who had GUD ask:**

How long did the most recent healed Ulcer last?

If < 1 week code days Days |__|__|{GUDAYS}

If < 1 month code Weeks Weeks |__|__|{GUWEEKS}

If < 1 year code Months Months |__|__|{GUMONTHS}

code completed months)

**If DK for all, code 97 in days and 98 in the rest)**

Q.193 **For respondents who had painful urination ask**:

How long did the painful urination last until it cured?

If < 1 week code days Days |__|__|{PANDAYS}

If < 1 month code Weeks Weeks |__|__|{PANWEEKS}

If < 1 year code Months Months |__|__|{PANMNTHS}

**If DK for all,code 97 in days and 98 in the rest)**

Q.194 **For respondents who had urethral** **discharge ask:**

How long did you have this discharge before it went away?

If < 1 week code days Days |__|__|{DISDAYS}

If < 1 month code Weeks Weeks |__|__|{DISWEEKS}

If < 1 year code Months Months |__|__|{DISMNTHS}

**If DK for all,code 97 in days and 98 in the rest)**

Q.195. Did you/ have you had sexual activity while suffering from these

symptoms

Yes 1 |__|{SYMPSEX}

No 2

DK 7

Q.196. Did you tell your spouse(s) or any partner(s) about these symptoms?

Yes 1 |__|{SPS}

No 2

( No sex partner during that period when

he had the symptoms)NA 8

NR 9

Q197. Did you do anything to help cure these symptoms or to prevent passing

on infection to your spouse/partner(s)?

Yes 1 |__|{TMT}

No 2 **----------------------------------->**Q. 199a

NR 9**------------------------------------>**Q. 199a

Q198 **If Yes to Q.197** ask: What actions did you take?

**PROBE and RECORD all RESPONSES**

Yes No NR

Used condoms 1 2 9 {CON}

Abstinence 1 2 9 {ABS}

Sought treatment for self 1 2 9 {SLF}

Sought treatment for partner 1 2 9 {TMTSPS}

Other action 1 2 9 {OTHERACT}

Specify)_____________________ {OTHACT}

**I DO NOT KNOW YOUR HIV STATUS AND I'M NOT GOING TO ASK**

**YOU ABOUT IT BUT WE ARE INTERESTED IN HOW MANY**

**PEOPLE REQUEST AND RECEIVE THEIR HIV RESULTS**

**FROM ANY ORGANIZATION**

Q.199a. Have you ever received your HIV results? |**__**| {RHIVEVER}

YES 1

NO 2------------------------------------------------------------Q.200f

NA 8-------------------------------------------------------------Q.200f

Q.199b In the last 12 months, have you received HIV results from anywhere? |__| {RHIVRLST}

YES 1

NO 2-----------------------------------------------------------Q.200f

Q.200a From whom did you receive your HIV results?

**[Code up to 2 responses]**

|__|__| {OGHIVTS1}

Rakai Project 01 |__|__| {OGHIVTS2}

Other NGO (Specify ______________ ) 03 {OTHOGHIV1memo}

Gov't Doctor/Nurse 04

Private Doctor/Nurse/Health worker 05

Other (specify _____________) 06 {OTHOGHIV2memo}

No additional response 88

Q.200b In the most recent 12 months did you discuss your HIV status with your husband or long term

consensual partner? |__|{HIVDISP}

Yes 1

No 2----------------->200f

N/A 8----------------->200f

NR 9----------------->200f

Q.200c What were his reactions?(**UNPROMPTED**)

Acceptance 01 |__|__|{ACTP1}

Discuss going for couple counselling 02 |__|__|{ACTP2}

Discuss going for HIV test/results 03 |__|__|{ACTP3}

Abstinence 04

Condom use 05

Other(SPECIFY)_________________ 06 {OTHACTP}

Faithfulness 07

Happy 08

Unhappy 09

Become violent 10

Abandoned me 11

**Code 88 for no additional response**

Q.200d Did you and your spouse/partner ever receive couples’counseling?

Yes 1 |__|{CPLECONS}

No 2-----------🡪200f

Q.200e When did you last receive couples’ counseling?

|__|__|{DYSCPLE}

|__|__|{WKSCPLE}

|__|__|{MTHCPLE}

|__|__|{YRSCPLE}

Q. 200f. Are you currently using sexual abstinence as a way of

preventing HIV infection? |__| {CURRABST}

Yes 1

No 2----------------------------------------------200i

NR 9----------------------------------------------200i

Q.200g. How long ago did you decide to be faithful to your spouse/partner

(Code days if less than a week, weeks if less than a month, months if less than a year

and years if more than 12 months)

|__|__|{DYSBSTN}

|__|__|{WKSABSTN}

|__|__|{MTHABSTN}

|__|__|{YRSABSTN}

Q.200h. Since taking the decision have you had sex?

Yes 1 |__|{SEXABST}

No 2

Q.200i. Are you currently being faithful to your wife/ partner in

order to prevent HIV infection? |__| {CURFAITH}

Yes 1

No 2-----------------------------------------200.1a

NR 9-----------------------------------------200.1a

Q.200j.Did you take the decision at the start of the relationship/marriage

or later on during the relationship/marriage?

At start of relationship 1 |__|{FAITHSTART}

Later on during relationship 2

Q.200k. How long ago did you decide to be faithful as way of preventing HIV

Infection? Days |__|__|{ DYSFAITH}

Weeks |__|__|{WKSFAITH}

Months |__|__|{MTHFAITH}

Years |__|__|{YRSFAITH}

Q.200l.What factors led you to being faithful as a way of preventing

HIV infection?

Got married 01 |__|__|{FAITHWHY1}

Learned my HIV status 02 |__|__|{FAITHWHY2}

Learned my patner’s HIV status 03 |__|__|{FAITHWHY3}

Got couple’s counseling 04

Got a steady partner 05

Lost a relative/friend to HIV 06

Partner did not accept condoms 07

I did not want to use condoms 08

Religious/moral ethics 09

It’s the right thing to do 10

Love/respect for my partner 11

My family wants me to be faithful 12

Other(specify)_________________ 13

Q.200m.Since taking that decision have you been unfaithful to

your partner?

Yes 1 |__|__|{UNFAITH}

No 2

**ARV MODULE**

**I WOULD LIKE TO ASK YOU SOME QUESTIONS ABAOUT ANTIRETROVIRAL DRUGS(ARVS).ARVS ARE MEDICINES WHICH HIV POSITIVE PERSONS CAN TAKE TO CONTROL THE VIRUS WHICH CAUSES AIDS.ARVS STARTED TO COME INTO UGANDA A FEW YEARS AGO.**

Q.200. 1a Have you heard about drugs called antiretroviral (ARV) for HIV

infected persons? |__|{HEARDARV}

Yes 1

No 2

Q.200.1. b **If Yes:**

Where did you hear about these medications? |__|__| {TOLDARV1}

Rakai Program 01

TASO 02

Uganda Cares 03 |__|__| {TOLDARV2}

Hospital 04

Govt health worker 05 |__|__| {TOLDARV3}

Private health worker 06

Radio 07

Newspaper 08

Church 09

Family member 10

Friend 11

Other ____________ 12 {OTOLDARV}

DK 97

Q.200.2a. Are ARVs available in Rakai District? |__| {AVLARVRD}

Yes 1

No 2------------------>Q 200.3

DK 7------------------->Q 200.3

Q.200.2b. **If yes**

Who provides ARVs in Rakai (**code all of respondent’s answers**)

Rakai Program 1 2 {RPARV}

TASO 1 2 {TASOARV}

Uganda Cares 1 2 {UGCARARV}

Hospital 1 2 {HOSPARV}

Govt doctor/health worker 1 2 {GOVTARV}

Private doctor/health worker 1 2 {PRIVTARV}

Traditional healer(s) 1 2 {TRADARV}

Other (specify) 1 2 {OTHARV}

Specify __________________ {OTHARVmemo}

DK 1 2 {DKARV}

Q.200.3. Do you personally know anyone who takes ARVs in your

community? |__|{USEARV}

Yes 1

No 2

Q.200.4. Are you aware of any traditional healer services offering

HIV/AIDS care in your community? |__|{TRADTRT}

Yes 1

No 2

Q.200.5. In your opinion, can traditional healers provide medications that can

cure HIV/AIDS? |__|{TRADHIV

Yes 1

No 2

DK 7

Q.200.6. Have you heard of drugs which can prevent transmission of HIV

from an infected mother to her baby? |__|{HRPMTCT}

Yes 1

No 2

Q.200.7a.From what you have heard, do ARV drugs have benefits for

an HIV-infected person? |__|{HRDBEN}

Yes

No----------------------------------------🡪Q.200.8

DK---------------------------------------🡪Q.200.8

Q.200.7b. If yes, what benefits? |__|__| {HRDBEN1}

**(UNPROMPTED)** **Code up to three** |__|__| HRDBEN2}

Cure HIV 01 |__|__| {HRDBEN3}

Make an HIV-infected person feel better/stronger 02

Make an HIV-infected person live longer 03

Help an HIV-infected partner have sex again 04

Help an HIV-infected person be able to work again/

care for family again 05

Reduce the chance of passing HIV to a partner 06

Eliminate the chance of passing HIV to a partner 07

Other _______________ 08 {OTHHRDBENmemo}

DK 97

Q.200.8. Can people taking ARVs transmit HIV if they have sex with their

partners without a condom?

Yes 1 |__| {ARVTRANS}

No 2

DK 7

Q.200. 9. From what you know, are there any difficulties in taking ARVs? |__| {ARVDIFF}

Yes 1

No 2 -------------🡪 Q 200.10

DK/NR

Q.200.9b. What are the difficulties in taking the medicine that you know of? |__|__| ARVDIFF1}

**UNPROMPTED Code up to three** |__|__|{ARVDIFF2}

Cause side effects 01 |__|__| ARVDIFF3}

Can make people feel sick 02

Hard to take 03

Many pills to swallow 04

Hard to remember to take pills 05

Expensive 06

Not always available/hard to get 07

Taste bad 08

Other: ______________________ 09 {OTARVDIFFmemo}

Need to eat a lot yet food not enough 10

Burden of lifetime treatment 11

DK 97

**NOW I WOULD LIKE TO ASK YOU ABOUT THE EFFECTS OF**

**THE AVAILABILITY OF FREE ARVS IN RAKAI**

Q.200.10. With the availability of free ARVs is there still a need to take

precautions, such as abstinence,monogamy or condom use, to prevent

getting or transmitting HIV? |__|{CAUTHIV2}

Yes 1

No 2

DK 7

Q.200.11. With the availability of free ARVS is it easier for people to

make the decision to learn their HIV results? |__|{VCTAKE2}

Yes 1

No 2

DK 7

**NOW I WOULD LIKE TO ASK FOR YOUR OPINION ABOUT**

**WHETHER THE AVAILABILITY OF FREE ARVS HAS**

**REDUCED, INCREASED OR HAD NO EFFECT ON THE**

**FOLLOWING CHARACTERISTICS AMONG THE PEOPLE IN**

**YOUR COMMUNITY**

Q.200.12 Do you think the availability of free ARVs has reduced, increased,

or had no effect on stigma against HIV+ persons in the community?: |__|{ARVSGM2}

Reduce stima 1

Increase stigma 2

No effect 3

Other(specify)______ _4 {ORVSTGMmemo}

DK____________________ 7

Q.200.13 Do you think the availability of free ARVs has reduced, increased, or had

no effect on HIV+ people’s willingness to disclose their HIV status to their

spouse/partner? |__|{DISSPOUSE2}

Increase disclosure 1

Decrease disclosure 2

No effect 3

Other (specify) ____________________ 4 {ODISSPOUS2memo}

DK/NR 7

Q200.14 Do you think the availability of free ARVs has increased, decreased or had

no effect on HIV+ people’s willingness to disclose their HIV status to their

family? |__| {DISFAMLY2}

Increase disclosure 1

Decrease disclosure 2

No effect 3

Other (specify) ___________________ 4 {ODISFAMLY2memo}

DK/NR 7

Q200.15 Do you think the availability of free ARVs has increased, decreased or had

no effect on HIV+ people’s willingness to disclose their HIV status in the

community, and if so, what effect? |__| {DISCOMMTY2}

Increase disclosure 1

Decrease disclosure 2

No effect 3

Other (specify) ____________________ 4 {SCOMMT2memo}

DK/NR 7

Q.200.16. Do you think the availability of free ARVs has affected sexual behaviors

in the community? |__| {ARVSEXB2}

Yes 1

No 2

DK 7

Q.200.17a. Do you think the availability of free ARVs has increased, decreased, or

had no effect on the number of partners that people have? |__| {ARCSEXPRD2}

More partners 1

Fewer partners 2

No effect 3

DK 4

Q.200.17b. Do you think the availability of free ARVs has made it more or less

likely that people would have more casual sex partners? |__| {ARVNBRPH2}

More casual partners 1

Less casual partners 2

No effect 3

DK 7

Q.200.17c. Do you think the availability of ARVs has increased, decreased or

had no effect on the practice of sexual abstinence for

HIV prevention in the community? |__| {ARVABS2}

More abstinence 1

Less abstinence 2

No effect 3

DK 7

Q.200.18. Do you think the availability of ARVs has increased, decreased or

had no effect on condom use in the community? |__|{ARVCOND2}

More use 1

Less use 2

No effect 3

DK 7

Q.200.20a. Do ARVs affect the risk of transmitting HIV infection from an

HIV+ person to their partner? |__| {ARVTRANS}

Yes 1

No 2

DK 7

Q.200.20b. If yes, what effect? |__| ARVTRANSW}

Decreased risk 1

Increased risk 2

DK 7

**FOR PERSONS WHO EVER HAD VCT(Q.199a is yes)**

**WE WANT TO ASK YOU ABOUT USE OF LONG-TERM MEDICATION.**

**THIS INFORMATION WILL BE HELD STRICTLY CONFIDENTIAL.**

**YOU DO NOT HAVE TO ANSWER THIS QUESTION.**

Q.201a. Are you or were you on any long-term medications? |__|{LTEMMED}

Yes 1

No 2________________Q.202

DK 7________________Q.202

NR 9________________Q.202

Q.201b. **IF YES** , are/were these ARV medications? |__|{ARVMED}

Yes 1

No 2

DK 7

NR 9

Q.202. During the last 12 months, have you had any of the following health

problems? **PROMPTED:**

Yes No NR

Substantial weight loss 1 2 9 {SYMP02}

Diarrhoea <=1 month 1 2 9 {SYMP03}

Diarrhoea > 1 month 1 2 9 {SYMP04}

Bloody Diarrhoea > 1 month 1 2 9 {SYMP05}

Fever <=1 month 1 2 9 {SYMP06}

Fever >1 month 1 2 9 {SYMP07}

Generalised severe itching 1 2 9 {SYMP08}

Body Rash **(not scabies)** 1 2 9 {SYMP09}

Cough >1 month 1 2 9 {SYMP10}

Kaposi sarcoma 1 2 9 {SYMP11}

Herpes zoster 1 2 9 {SYMP12}

Swollen glands in the neck or arm pits 1 2 9 {SYMP13}

Malaria(B/S) 1 2 9 {SYMP14}

Candida (Oral Thrush) 1 2 9 {SYMP15}

Tuberculosis 1 2 9 {SYMP16}

Scabies 1 2 9 {SYMP17}

Onyongnyong 1 2 9 {SYMP18}

Pneumonia **(Probe for chestpain,fever,cough,**

difficulty in breathing 1 2 9 {SYMP19}

Meningitis 1 2 9 {SYMP20}

Other Cancers 1 2 9 {SYMP21}

Specify __________________ {OTHCANC}

Other problems 1 2 9 {SYMP22}

Specify __________________ {OTHSYMP}

Q.203. Have you been bedridden in the past 30 days?

Yes 1 |__|{BEDRID}

No 2----------> Q. 204

**If Yes** How long were you in bed?

Less than half a day 1 |__|{BEDTIME}

Half a day to a day 2

More than a day (give # of days) 3

**(Record actual number of days)---------------------------------->**|__|__|{DYSBDRID}

.204. Have you had a blood transfusion in the last 12 months ?

Yes 1 |__| {BLOODTRA~~Y1~~

No 2

Q.205. In last 12 months , have you received any injection(s) from any

source other than Rakai program? |__|{INJOUTRP}

Yes 1

No 2

DK 7

Q.207 How likely do you think that you have been exposed to HIV (virus

which causes AIDS)? (**PROMPTED**)

Very likely 1 |__|{SAIDSRSK}

Somewhat likely 2

Unlikely 3

Not at all 4

Don't Know 7

Q.210a. Are you circumcised? Yes 1 |__| {CIRCY1}

No 2 -------------> SKIP TO Q.207

Q.210b. Where were you circumcised from?

Circumcised by Rakai program 1 |__| {MCIRCPLA}

Government Health Unit 2

Private practitioner 3

Other (Specify source)______________ 4 {OMCIRCPL}

DK 7

Q.210c. How long ago were you circumcised?

Less than 1 day code 00 days, Less than 1 week code Days |__|__| PDCIRCY1}

Less than one month code Weeks |__|__| {PWCIRCY1}

Less than one year code Months |__|__| {PMCIRCY1}

If one year or more code completed Year |__|__| {PYCIRCY1}

(If DK Code 97 in days and 98 in otherboxes; 99 for NR)

IF CIRCUMCISED >3 YEARS SKIP TO 207 ELSE ASK 211a

Q.211a Have you had sex with any one since you were circumcised? |__|{SEXOPY1}

Yes 1

No 2-----🡪.END

NR 9

Q.211b How soon after circumcision did you resume sex?

HOURS |__|__|{SEXSONHRY1}

DAYS |__|__|{SEXSONDYY1}

MONTHS |__|__|{SEXSONMOY1}

YEAR |__|__|{SEXSONYRY1}

If DK/DR code 97 in HOURS and 98 in other boxes

TIME ENDED |__|__| |__|__| am/pm {TEND}

Editor # |__|__|__|{EDITOR}QC # |__|__|__|{Q CONTROL}TEAM LEADER#|__|__|__| {TEAMLEAD}

Data Entry Clerk # |__|__|__| {DATA CLERK} DE Datedd mm yyyy__/__/200__

# PARTNER IDENTIFICATION

| **PUT COMPUTER ID HERE** |
| --- |

**__**_**_/**_**__/200__**

Int_Date dd mm yyyy

VISIT # **|__|__|__|__|** {VISITNO}

Current ID

Super Cluster # |__|__|__|{REGION}

Community # |__|__|__|{COMM_NUM

HH # |__|__|__|__|{HH_NUM}

Member # |__|__|__|{MEMBER_NUM

**INTERVIEWER:**

**FOR ALL RESPONDENTS IN MARRIAGE OR CONSENSUAL UNION TRY TO OBTAIN INFORMATION THAT WILL ALLOW US TO LINK THE INDIVIDUAL TO HIS MARRIAGE OR CONSENSUAL PARTNERS WITHIN THE SAME HOUSEHOLD OR WITHIN THE SAME COMMUNITY. PAY ATTENTION TO CASES OF POLYGAMY WHERE WIVES MAY BE LIVING WITHIN ONE HOUSE OR IN DIFFERENT HOUSEHOLDS. USE ADDITIONAL PAGES IF >4 PARTNERS.**

**FOR SAME HOUSEHOLD PARTNERS, TRY TO FIND THE MEMBER # FROM THE HOUSEHOLD CENSUS LISTING. FOR PARTNERS IN DIFFERENT HOUSEHOLDS, THEIR INFORMATION WILL HAVE TO BE COMPLETED LATER.**

"You stated previously that you are currently married or in a consensual union"

(If Yes),

**Partner 1**

I would like to clearly know, do you have a wife that you are staying with in this household?

**(If Yes,)** Tell me all the possible names by which she is called.

__**If she has current ID record it here otherwise write actual location below.**_____________________________________________________________

Current ID # for partner 1: **|__|__|/|__|__|__|/|__|__|__|__|/|__|__|**

(supercluster/community/household/member)

**Location: (Similar to that of husband) _______________________________________________________**

**__________________________________________________________**

**(If No),** Does she normally stay in your community?

Yes .....1 No.....2 Outside spouse ...3

What are her names?________________________________________________________

**SUPERVISOR: TRY TO LOCATE ID FROM HOUSEHOLD CENSUSES IN COMMUNITY**.

Current ID # for partner 1: **|__|__|/|__|__|__|/|__|__|__|__|/|__|__|**

(supercluster/community/household/member)

If Yes, write the physical location:__________________________________________________

**(If not)** Can you tell me where she stays?

_________________________________________________________________

_________________________________________________________________

**Partner 2**

Do you have any other wife you are staying with in this household?

**(If Yes,)** Tell me all the possible names by which she is called.

_______________________________________________________________

**If she has current ID record it here otherwise write actual location below.**

Current ID # for partner 2: **|__|__|/|__|__|__|/|__|__|__|__|/|__|__|**

(supercluster/community/household/member)

**Location: (Similar to that of husband)_______________________________________________________**

**______________________________________________________________________________**

**(If No),** Does she normally stay in your community?

Yes .....1 No.....2 Outside spouse.....3

What are her names? ___________________________________________________________

**SUPERVISOR: TRY TO LOCATE ID FROM HOUSEHOLD CENSUSES IN COMMUNITY**.

Current ID # for partner 2: **|__|__|/|__|__|__|/|__|__|__|__|/|__|__|**

(supercluster/community/household/member)

If Yes, write the physical location:_______________________________________________________

**(If not)** Can you tell me where she stays?

________________________________________________________________

**Partner 3**

Do you have any other wife you are staying with in this household?

**(If Yes,)** Tell me all the possible names by which she is called.

_______________________________________________________________

**If she has current ID record it here otherwise write actual location below.**

Current ID # for partner 3: **|__|__|/|__|__|__|/|__|__|__|__|/|__|__|**

(supercluster/community/household/member)

**Location: (Similar to that of husband) _________________________________________________**

**_________________________________________________________________**

**(If No),** Does she normally stay in your community?

Yes .....1 No.....2 Outside Spouse .....3

What are her names?_________________________________________________________

**SUPERVISOR: TRY TO LOCATE ID FROM HOUSEHOLD CENSUSES IN COMMUNITY**.

Current ID # for partner 3: **|__|__|/|__|__|__|/|__|__|__|__|/|__|__|**

(supercluster/community/household/member)

If Yes, write the physical location:_______________________________________________________

**(If not)** Can you tell me where she stays?

_________________________________________________________________

**Partner 4**

Do you have any other wife you are staying with in this household?

**(If Yes,)** Tell me all the possible names by which she is called.

_______________________________________________________________

**If she has current ID record it here otherwise write actual location below.**

Current ID # for partner 4: **|__|__|/|__|__|__|/|__|__|__|__|/|__|__|**

(supercluster/community/household/member)

**Location: (Similar to that of husband)_______________________________________________________**

**______________________________________________________________________________**

**(If No),** Does she normally stay in your community?

Yes .....1 No.....2 Outside Spouse .....3

What are her names?

_________________________________________________________________

**SUPERVISOR: TRY TO LOCATE ID FROM HOUSEHOLD CENSUSES IN COMMUNITY**.

Current ID # for partner 4: **|__|__|/|__|__|__|/|__|__|__|__|/|__|__|**

(supercluster/community/household/member)

If Yes, write the physical location:_______________________________________________________

**(If not)** Can you tell me where she stays?

_________________________________________________________________

**APPENDIX 7.**

**RAKAI COMMUNICTY COHORT STUDY (RCCS)**

**ROUND 12 BASELINE MALE QUESTIONNAIRE Last Edited Nov. 06 2006**

INTERVIEWER # |__|__|__|{FIELDWRKR}

**PLACE**

**COMPUTER ID HERE**

INTERVIEW DATE {INT DATE ddmmyy}

___/___/200_

dd mm yyyy

VISIT #  **|R|12|**{VISITNO}

SuperCluster # | |__|__|__|{REGION}

Community # | |__|__|__|{COMM_NUM}

HH # |__|__|__|__|{HH_NUM}

Member # |__|__|__|{MEMBER_NUM}

SEX **M**

Q.1 How old are you? **Age in completed years**

**(Valid range 15-49, Code 97 for Don't know)** |__|__|{AGEYRS}

Q.2 What is your birth date? Day |__|__|{BIRTHDY}

**(check birth date vs age** **reconcile** **the two**) Month |__|__|{BIRTHMO}

**if needed** **code 99 for unknown dd, mm, yy)** Year |__|__|__|__|{BIRTHYR}

Q.3 To which tribe do you belong? |__|{TRIBE}

Muganda 1

Munyankole 2

Munyarwanda 3

Murundi 4

Mukiga 5

Mutanzania 6

Other **(specify)**______________________ 7 {OTHTRIBE}

Q.4 What is your religion?

None 1 |__|{RELIGION}

Catholic 2

Protestant (inc. Church of Uganda) 3

Saved/Pentecostal 4

Muslim 5

Other **(specify)**__ _____________________6 {OTHRELIG}

Q.5 Have you ever gone to school? Yes 1 |__|{EDUCATE}

No 2------------>**Q.7**

Q.6 To what level? P1-P4 1 |__|{EDUCYRS)

(Code highest level) P5-P7 2

S1-S4 3

S5-S6 4

Technical/University 5

Primary professional 6

O’level professional 7

Q.7 What kind of work do you do, or what kind of activities keep you busy

during an average day, whether you get money from them or not?

**(Record answer(s) as given):**___________________**__________________ (OCCUP 1)**

____________**___________________________ (OCCUP 2)**

**(Code up to two responses, code first mentioned** **occupation first)**

Agriculture for home use/barter 01 |__|__|{OCCUP1}

Agriculture for selling 02 |__|__|{OCCUP2}

Housework in your own home 03

Housekeeper (for relative or employer) 04

Home brewing 05

Government/clerical/teaching 06

Fishing 07

Student 08

Military/police 09

Shopkeeper 10

Trading/vending 11

Bar worker or owner 12

Trucker 13

Unemployed (**PROBE _ NO AGRIC OR HOUSE WORK?**) 14

Other(specify)______________________ 15

No additional occupation (use in 2nd field if one

Occupation is cited) 88

Q.8 How long have you lived in this community?

**if < 1 week code days**  DAYS |__|__| |{RESIDDYS}

**if < 1 month code weeks**  WEEKS |__|__|{RESIDWKS}

**if < 1 year code months else completed years** MONTHS |__|__|{RESIDMOS}

YEARS |__|__|{RESIDYRS}

**(If DK for all Code 97 in days and 98 in other boxes; 99 for NR**

**code 91<2 years,92:2-4 years,93:5-9 years,94:10 and above)**

Q.9 How long do you intend to stay in this community?

**if < 1 week code days**  DAYS |__|__|{LONGDYS}

**if < 1 month code weeks**  WEEKS |__|__|{LONGWKS}

**if < 1 year code months** MONTHS |__|__|{LONGMOS}

**If >=1 year code years**  YEARS |__|__|{LONGYRS}

**(If ALL LIFE Code 77 in days and 98 in other boxes)**

**(If DK for all Code 97 in days and 98 in other boxes; 99 for NR**

**code 91<2 years,92:2-4 years,93:5-9 years,94:10 and above)**

Q.10 How long do you intend to stay in this district?

**if < 1 week code days** DAYS |__|__|{INTEDDYS}

**if < 1 month code weeks** WEEKS |__|__|{INTEDWKS}

**if < 1 year code months** MONTHS |__|__|{INTEDMOS}

**If >=1 year code years** YEARS |__|__|{INTEDYRS}

**(If ALL LIFE Code 77 in days and 98 in other boxes)**

**(If DK for all Code 97 in days and 98 in other boxes; 99 for NR**

**code 91<2 years,92:2-4 years,93:5-9 years,94:10 and above)**

Q.11 In the last 12 months, have you travelled to any

of the following places? NA for residence

**[PROMPTED]** Yes No NA

Kalisizo town 1 2 8 {TRAVYR01}

Kyotera town 1 2 8 {TRAVYR02}

Lyantonde town 1 2 8 {TRAVYR03}

Other place >3miles/>1hr walk in Rakai district 1 2 {TRAVYR05}

Masaka district 1 2 {TRAVYR11}

Kampala District 1 2 {TRAVYR12}

Other Place in Uganda 1 2 {TRAVYR09}

Other country (Specify) 1 2 {TRAVYR10}

____________________________ {OTTRAVYR Memo}

Q.21 How many children would you wish to have in your lifetime?

(code actual number, 97 for DK)

|__|__|{FERTINTENT}

**I WOULD LIKE TO ASK YOU ABOUT WAYS IN WHICH PEOPLE CAN**

**PLAN** **THEIR FAMILIES**

Q.22 Are you or any of your partners currently using the

following Family planning methods? **PROMPTED**

**If using calendar, circle calendar and the back up.**

Yes No DK

Pills 1 2 7 {FPUSING1}

Condom 1 2 7 {FPUSING2}

Spermicide 1 2 7 {FPUSING3}

Injection 1 2 7 {FPUSING4}

Abstinence 1 2 7 {FPUSING5}

Calendar/rythm 1 2 7 {FPUSING6}

IUD 1 2 7 {FPUSING7}

Other method 1 2 7 {FPUSING8}

Specify ________________ {OTHFP}

Breastfeeding 1 2 7 {FPUSING9}

Herbs/Traditional Medicine 1 2 7 {FPUSNG10}

**IF HE DOES NOT CURRENTLY USE ANY FP METHOD SKIP TO Q.24**

Q.22b Where do you usually get family planning methods/services, from?

Government clinics 01 |__|__| {FPLACE1} Rakai Project clinics 02 |__|__| {FPLACE2}

Private clinics 03

Social Marketing Shops 04

Other ____________________ 05 {OTFPLACE}

Drug shop/pharmacy 06

Herbalist/traditional healer 07

COBRA 08

No additional response 88

Do not know 97

No t Applicable 98

Q.24 Have you and any of your partner(s) ever had a discussion on using any |__|{FPEVERDSC}

family planning method in the course of your relationship?

Yes 1

No 2

NA 8

Q.25 Have you or any of your partner(s) ever used a condom for STD/HIV prevention? |__|{CONDEVER}

Yes 1

No 2--------->**Q.26b**

NA 8--------->**Q.31a**

Q.26a Are you or any of your partner(s) currently using condoms for STD/HIV

prevention? |__|{CONDMNOW}

Yes 1---------->**Q.27**

No 2

NA 8---------->**Q.31a**

Q.26b What are the reasons you did not use/ are not using condoms for STD/HIV

prevention?

Do not know how to put on a condom 01 |__|__|{CURNOCO1}

Want to have a child/children 02 |__|__|{CURNOCO2}

We have both tested negative for HIV 03 |__|__|{CURNOCO3}

Condoms reduce sexual satisfaction 04

Condom use is against our religious beliefs 05

Condom use is against our traditional beliefs 06

I trust my partner(partner is faithful) 07

My partner trusts me 08

Do not trust condoms 09

Do not know where to get condoms 10

Cannot afford condoms 11

Don’t feel comfortable getting condoms in our community 12

Condom use is not compatible with our sexual style 13

The condoms do not fit properly 14

Partner refused 15

Other(specify)_____________ 16 {CURNOTHER}

Partner does not have HIV/STD 17

I don’t have HIV/STD 18

**(If answered Qn 26b, skip to Qn 31a)**

Q.27 Where do you usually get condoms

for HIV/STD prevention from?

Government clinics 01 |__|__| {CPLACE1}

Rakai Project clinics 02 |__|__| {CPLACE2}

Private clinics 03 Social Marketing Shops 04

Other ____________________ 05 {OTCPLACE}

Drug shop/pharmacy 06

Herbalist/traditional healer 07

COBRA 08

No additional response 88

Do not know 97

Q.31a Have you ever been married or entered a consensual

union? **(If present union is the** **ONLY marriage ever,**

**code Yes)** Yes 1 |__| {EVERMARR}

No 2---------->**Q.37**a

DK 7---------->**Q.37**a

Q.31b In your life time how many marital/consensual unions have you had

Including those that have dissolved |__|__|{MARORDER}

Q.32 How old were you when you first got married or entered

a consensual union? |__|__|AG1STMAR}

**(Record age in completed years, 97 for DK)**

Q.33 Are you currently married (whether traditional, civil,

or religious, or in a consensual union)?

Yes 1 |__|{CURRMARR}

No 2-------------------->**Q.36**

Q.34 How many wives do you have now? |__|__|{POLYMAR}

[**Record actual # wherever possible, otherwise**

**code 92= a few (01-02), 93=a lot/many(03+), no response= 99]**

Q.35 **[FOR CURRENTLY MARRIED ASK]**

Is your marriage or union **(Prompted)** Traditional 1 |__|{MARRSP1}

Religious 2 |__|{MARRSP2}

Consensual 3 |__|{MARRSP3}

Civil 4 |__|{MARRSP4}

Don't Know 7

Q.36 What is the state of past marital relationships?**(Prompted)**

**(Most recently ended marriage first)**

Never married 1 |__|{PSTAT1}

Separated 2 |__|{PSTAT2}

Divorced 3 |__|{PSTAT3}

Widowed/Partner died 4 |__|{PSTAT4}

Q.37a Do you currently have a relationship with someone to whom

you are not officially married or in a consensual union?

Yes 1 |__| {CURRLTN}

No 2

DK 7

Q37b Do you currently have a mutual non sexual relationship with someone

you are not officially married to/in a consensual union with?

Yes 1 |__|__|{ABSTAIN}

No 2---------->**Qn.38**

Q.37c How long ago did this mutual non-sexual relationship start? |__|__|{DAYSABST}|__|__|{WEEKSABST}

|__|__|{MTHABST}

|__|__|{YRSABST}

Q.38 Have you had sexual intercourse with any person in the

last 12 months? Yes 1 |__|{SEXYEAR}

No 2>**Q.46**

NR 9

Q.41 How many different sexual partners have you had in the last 12 months,

including married or consensual partners and anyone already mentioned? |__|__|{SEXP1YR}

**[Record actual # wherever possible, otherwise**

**code 92= a few (01-02), 93=a lot/many(03+), no response= 99]**

Q.42 How many partners in the last twelve months were from outside this community? |__|__|{SEXP1OUT}

**[Record actual # wherever possible, otherwise**

**code 92= a few (01-02), 93=a lot/many(03+), no response= 99]**

Q.43 How long since you last had sexual intercourse?

**[if< 1 day, code 00 if < 1 Week, code days, if < 1 Month, code weeks if < 1 year, code months]** Days |__|__| {SEXYRDAYS}

**If DK code 97 in days and 98 in the rest** Weeks |__|__| {SEXYRWKS}

Months |__|__| {SEXYRMOS}

Q.44 During the past 12 months have you or your partner(s) used condoms?

Yes 1 |__|{CONYR}

No 2-------------------->**Q.46**

DK 7-------------------->**Q.46**

Q.45 **If Yes, to used condoms ask:** When you have sex, how often

did you use condoms, sometimes or always? |__|{CONSISYR}

Sometimes 1

Always 2

DK 7

Q.46 How many different sexual partners have you had in the last 5 years,

including married or consensual partners and anyone already mentioned? |__|__| {SEXP5YR}

Valid codes: Record actual # wherever possible, otherwise

**code 92= a few (01-02), 93=a lot/many(03+), no response= 99]**

Q.47 How many different sexual partners have you had in your lifetime including

married or consensual partners. |__|__|{SEXPEVER}

**[valid codes: Record actual # wherever possible, otherwise**

**code 92= a few (01-02), 93=a lot/many(03+), no response= 99]**

**IF NEVER MARRIED OR IN A RELATIONSHIP AND NO SEXUAL**

**PARTNER IN THE 12 months (i.e. Q.31-47 ARE NO/00)**

**THEN ASK Q.48**  **ELSE SKIP TO Q.49b**

Q.48 Have you ever had a sexual relationship? Yes 1 |__|{EVERSEX}

No 2---------------->**Q.50c**

**FIRST SEXUAL EXPERIENCE: MEN**

Q.49b How old were you the first time you had sexual intercourse? |__|__| {AGE1STSEX}

**[Record completed years, DK=97, NR=99]**

Q.49c Thinking back to that time when you first had sex, how willing

were you at that time to have sex? |__| {WILFSTSX}

Very willing 1

Somewhat willing 2

Not willing/Unwilling 3

DK 7

NR 9

Q.49d What was your relationship with the person you had sex with the

first time? |__|__| {WHOFSTSX}

Current wife (at the time) 01

Current consensual partner ( at the time) 02

Girlfriend 04

Occasional or casual friend 05

Visitor (incl. wedding/funeral) 06

Stranger 07

Workmate 08

Boss/work supervisor 09

Employee 10

Fellow student 11

Sugar mammy 12

Relative other than spouse **(specify)________________** 13 {OTHRLTNR1memo}

Other non relative **(specify)________________** 14 {OTHRLNR1}

Rapist (by a stranger) 15

Religious leader 16

Teacher 17

Don't Know 97

Q. 49e Did your first sexual partner give you presents or money at that time? |__| {FSPBUY }

Yes 1

No 2

DK/DR 7

Q.49f Did your partner use a condom at the time of first sex? |__| {FSPCONDO}

Yes 1

No 2

DK/DR 7

Q.49g Was the person you first had sex with older than you, same

age as you or younger than you? |__| {PYOFSTSX}

Older than you 1

Younger than you 2

Same age as you 3 ---------------> **SKIP TO Q.49l**

Don’t know 7 ---------------> **SKIP TO Q.49l**

No response 9 ---------------> **SKIP TO Q.49l**

CURRENT ID #

____/____/____/____

COMPUTER ID

____________________

Q.49h **IF NOT SAME AGE:**

How many years older or younger was he? |__|__| {PAGFSTSX}

**[Record actual # or 97=Don’t know]**

Q.49l How did you feel after your first sexual experience? |__| {FELFSTSX}

Regrets 1

No regrets 2

Happy 3

Other (Specify) ________________ 4

DK/DR 7

No response 9

Q.49m Were you still in school, before joining school, never ever went

to school or had you left school at the time you first had sex? |___| {FSEXEDUC}

Before joining school 1

In school 2

Had left school 3

Never ever went to school 4

DK/DR 7

NR 9

Q.49n Thinking back to the first time you had sex, were either you or your

female partner under the influence of alcohol at that time? **PROMPT** |___| {FSEXALC}

Neither under the influence 1

Respondent only under the influence 2

Female partner only under the influence 3

Both under the influence 4

### DK/DR 7

NR 9

**IN RELATIONSHIPS BETWEEN MEN AND WOMEN, DISAGREEMENTS**

**ON SOME ISSUES OCCUR, WHICH SOMETIMES RESULT INTO**

**VIOLENCE. I WOULD LIKE TO ASK YOU SOME QUESTIONS ON**

**VIOLENCE.**

Q.50 In the past 12 months did you do the following to your wife/partner: **PROMPTED**

**Yes No NA**

Verbally abuse or shout at her? 1 2 8 {PABUSEYR}

Push her, slap her or held her down? 1 2 8 {PPUSHYR}

Punch her with fist or with something that could hurt her? 1 2 8 {PFISTYR}

Kick her or dragged her? 1 2 8 {PKICKYR}

Tried to strangle her or burn her? 1 2 8 {PBURNYR}

Threatened her with a knife, gun, or other weapon? 1 2 8 {PWEAPYR}

Attacked her with a knife, gun, or other type of weapon? 1 2 8 {PATKNYR}

Other (specify)________________________ 1 2 8 {POTHVYR}

Q.50b In the past 12 months, have you done of the following to any of your

sexual partners: **[PROMPT]**

Yes No NA

Used verbal threats to force her to have sex

when she did not want to? 1 2 8 {VTHRSXPY}

Physically forced her to have sex when she did not want to? 1 2 8 {PFOCSXPY}

Forced her to perform other sexual acts she did not want to do? 1 2 8 {OTHFSXPY}

Q. 50c Is a man justified in beating his wife/partner if?

Yes No

She neglects household responsibilities 1 2 {BJNEGHH}

She disobeys the instructions of her husband/elders 1 2 {BJDOBEY}

She uses contraception without permission 1 2 {BJFPPRM}

she refuses her husband sex 1 2 {BJREFSEX}

He learns about wife’s/partner’s positive HIV sero status 1 2 {BJPHIVST}

He learns about his positive HIV sero status 1 2 {BJHIVST}

Argues over money 1 2 {BJARGMON}

Is unfaithful 1 2 {BJUNFTH}

Other reasons (**PROBE**) 1 2 {BOTREAS}

Specify _______________________________ {OTHBEATmemo}

**IF NEVER HAD SEX SKIP TO Q.169a**

**BEGIN REPETITIVE SEXUAL PARTNER BLOCKS**

**(The following blocks of questions should be asked for** **each current and past**

**relationship in the last 12 months up to 4 partners. Begin by asking about the**

**most** **recent partner. If the most recent relationship was more** **than a year ago,**

**still ask about this most recent partner).**

**FIRST BLOCK:**

**"Now I would like to ask you about your most recent sex partners.**

**Please remember that all of your answers are confidential. Your answers are**

**very important to our research to help us understand health problems**

**in Rakai".**

Q.51 Remembering the most recent time you had sex, what

was your relationship to that partner at that time?

Current wife(at the time) 01 |__|__| {RLTN1}

Current consensual partner( at the time) 02

Former wife/consensual 03

Girlfriend 04

Occasional or casual friend 05

Visitor (incl. wedding/funeral) 06

Stranger 07

Workmate 08

Boss/work supervisor 09

Employee 10

Fellow student 11

Sugar mummy 12

Relative other than spouse (specify) 13 {OTHRLTN1}

Other non relative **(specify)________________** 14 {OTHRLNR1}

Don't Know 97

Q.52 What are/were your partner's main occupations?

**(Record answer(s) as given):**__________________**(OCCUPATION 1)**

__________________**(OCCUPATION 2)**

**(Code up to two responses, code first mentioned** **occupation first.)**

Agriculture for home use/barter 01 |__|__|{OCCUP11}

Agriculture for selling 02 |__|__|{OCCUP21}

Housework in own home 03

Housekeeper (for relative or employer) 04

Home brewing 05

Government/clerical/teaching 06

Fishing 07

Student 08

Military/police 09

Shopkeeper 10

Trading/vending 11

Bar worker or owner 12

Trucker 13

Unemployed (**PROBE _ NO AGRIC OR HOUSE WORK?**) 14

Other ____________________________ 15

No additional response(use in 2nd field if one occupation is cited 88

Do Not Know 97

Q.53 How long did you know this person before you had sex with

him for the first time?[i.e from the time he/you proposed

a relationship to first sex]

**Less than 1 day code 00, Less 1 week** code days |__|__|{COTDYSP1}

**Less 1 month code weeks** weeks |__|__|{COTWKSP1}

**Less 1 year code months** months |__|__|{COTMOSP1}

**If 1 year or more code completed years** years |__|__|{COTYRSP1}

**(If DK Code 97 in days and 98 in other boxes; 99 for NR)**

Q.54 How long ago did you first have sex with this person?

**Less than 1 day code 00, Less than 1 week code days**  |__|__|{DAYS1}

**Less than one month code weeks weeks** |__|__|{WEEKS1}

**Less than one year code months months** |__|__|{MONTHS1}

If **1 year or more code completed Years Years** |__|__|{YEARS1}

**(If DK Code 97 in days and 98 in other boxes; 99 for NR)**

Q.55 Are you still in a sexual relationship with her?

Yes 1 |__| {RLTONGO1}

No 2

Don't know 7

**During the last time you were having sexual relations with this**

**partner, please tell me how often you usually had intercourse.**

**For Qs. 56-58a If Don't know write DK and**

**no response** **write NR besides the box**

Q.56 When you had/have sex, how many times per day did/do you usually

have sexual intercourse? **(code # of times per day)**  |__| {TIMDAY1}

Q.57 How many days per week did/do you usually have sexual intercourse?

**(code # of days per week)**  |__|{DAYSWEK1}

Q.58 How many weeks per month did/do you usually have sexual

intercourse? **(code # of weeks per months)** |__|{WKSMON1}

Q. 58a How many months in the past 12 months did/have you had sexual

intercourse? **(Code # of months in the past 12 months)**  |__|__| {MTSPYR1}

**IF RELATIONSHIP ENDED >30 DAYS AGO SKIP TO Q.59**

Q.58 b In the past 7 days how many times did you have sexual intercourse

with this person?

**[Record actual #, code 97 for DK, 98 for NA, 99 for NR]** |___|___|{SFREQWK1}

Q.58 c In the past 30 days how many times did you have sexual intercourse

with this person?

**[Record actual #, code 97 for DK, 98 for NA, 99 for NR]** |___|___|{SFREQMN1}

Q.59 Is/was she older, younger, or about the same age ?

Older 1 |__| {RLTNAGE1}

Younger 2

Same age 3---->**Q.61**

DK 7----->**Q.61**

Q.60 About how many years [older/younger] ? |__|__|{RLTNYRS1}

**(Record actual # or 97=don't know)**

Q.61 Does(was) she live(living) in this household ? |__|{RLTNHH1}

Yes 1-------->**Q.64**

No 2

Q.62 Does(was)she regularly live(living) in this community? |__| {RLTNCM1}

Yes 1---->**Q.64**

No 2

DK 7

Q.63 How far away does/was she normally live/living?

Within one hour’s walk or 3 miles 1 |__| {RLTNRES1}

More than one hour's walk or >3 miles 2

Don't Know 7

Q.64 Have you and this partner ever used a condom? |__|{CNDEVER1}

Yes 1

No 2---------------->**Q.67**

NR 9

Q.65 During the most recent 12 months you were having sexual relationship

with this partner, how often did you use condoms? |__|{RNYRCON1}

Never 1**---------------->Q.67**

Sometimes/inconsistent 2

Always 3

DK 7

Q.66a Why did you use condoms with this partner?**(UNPROMPTED)**

Yes No

To prevent HIV transmission/acquisition/re-infection 1 2 {CONDHIV1}

To prevent STD transmission/ acquisition 1 2 {CONDSTD1}

For family planning 1 2 {CONDFP1}

Just trying it out 1 2 {CONDTRY1}

Other________________________ 1 2 {CONDOTH1}

Q.66b Did you use a condom the last time you had sex with that partner?

Yes 1 |__|{RLTNLST1}

No 2

DK/DR 7

Q66c During the the last 30 days you were having a sexual relationship |__|{RNMTCON1}

with this partner, how often did you use condoms?(PROMPTED)

Never 1

Sometimes/inconsistent 2

Always 3------------>Q.67

Dk 4------------>Q.67

Q.66d In the most recent 30 days what are the reasons you did not use

/inconsistently used condoms with this partner?

Do not know how to put on a condom 01 |__|__|{PCURNOCO11}

Want to have a child/children 02 |__|__|{PCURNOCO21}

We have both tested negative for HIV 03 |__|__|{PCURNOCO31}

Condoms reduce sexual satisfaction 04

Condom use is against our religious beliefs 05

Condom use is against our traditional beliefs 06

I trust my partner(partner is faithful to me) 07

My partner trusts me 08

Do not trust condoms 09

Do not know where to get condoms 10

Cannot afford condoms 11

Don’t feel comfortable getting condoms in our community 12

Condom use is not compatible with our sexual style 13

The condoms do not fit properly 14

Partner refused 15

Other_________________________________ 16

Partner does not have HIV/STDS 17

I don’t have HIV/STD S 18

Q.67 Have you ever discussed condom use with this partner?

Yes 1 |__|{CNDISCP1}

No 2

DK/DR 7

Q.68 In the last 12 months while you were having a relationship with this person,

did this partner have sexual relations/ intercourse with persons other

than yourself?

Yes 1 |__| {RLNSXOT1}

No 2>**Q.70**

DK 7>**Q.70**

Q.69 **If yes in Q.68;** How many other sexual partners other than yourself,

do you think your partner had sex with in the last 12 months, while you

were in a sexual relationship? |__|__|{RLTPNO1}

**COMPUTER ID**

**--------------------------------------------------**

**CURRENT ID**

**-------/-------/-------/-------**

Q.70 How long ago, did you last have sex with this person?

**Less than 1 day code 00 days, less than 1 week code** days |__|__|{RLDYSLT1

**Less than 1 month code weeks** weeks|__|__|{RLWKSLT1

**Less than 1 year code months** months|__|__|{RLMOSLT1

**If one year or more code completed years** years |__|__|{RLYRSLT1

**(If DK Code 97 in days and 98 in other boxes; 99 for NR)**

Q.70a When you have/had sex with this partner do/did you wash your genitals

before sexual intercourse?

Yes 1 |__| [WSHBFSX1]

No 2 --------------> **Q.71a**

Q.70b. **IF ‘YES’,** How frequently? (Unprompted)

Sometimes 1 |__| [FQWSHBF1Y1]

Usually 2

Always 3

Q.70c Generally, how soon before sexual intercourse?

**If less than 1 minute, code 00 minutes, If less than 1 hour (60 min),**

**code completed minutes If less than 1 day, code completed hours,**

**If one day or more, code days.**

**Minutes** |__|__| [MINBFSX1Y1]

**Hours** |__|__| [HRBFSX1Y1]

**Days** |__|__|[DAYBFSX1Y1]

Q.71a. When you have sex with this partner do you wash your genitals after sex?

Yes 1 |__| [WSHAFTS1Y1]

No 2 --------------> **Q.72**

Q.71b **IF ‘YES’,** How frequently? (Unprompted)

Sometimes 1 |__|[FQWSHAFTS1Y1]

Usually 2

Always 3

Q71c. **IF ‘YES’;** How soon after?

**If less than 1 minute, code 00 minutes, If less than 1 hour (60 min),**

**code completed minutes, If less than 1 day, code completed hour.**

**MINUTES** |__|__| [MINAFTS1]

**HOURS**  |__|__|[HRAFTS1]

Q.72. When you have/had sex with this partner what do you use to clean your

genitals after sex? Piece of cloth 1 |__|{CLINGEN11}

Washing 2 |__|{CLINGEN21}

Piece of cloth and washing 3

Other specify_______________ 4

None 5

No additional response 8

Q.73a. Do/did you drink alcohol before sex with this partner? |__| {ALCRBSEX1}

Yes 1

No 2 -------- > **Q.74a**

Q.73b **IF YES**, to Q.73a ask: Is/was it sometimes or always? |__|{ALCSXSF1}

Sometimes/Inconsistent 2

Always/Often 3

DK 7

Q.73c. During the time you took alcohol and have/had sex with this partner

do/did you usually have the following effects? **(PROMPT)**

Yes No NR

Unsteady gait 1 2 9 {ALUNGSF1}

Fall over 1 2 9 {ALFOSF1}

Get angry/violent 1 2 9 {ALANVSF1}

Difficulty speaking 1 2 9 {ALDSPSF1}

Other 1 2 9 {ALOTHSF1}

Specify _____________________ {OTHALSF1}

Q.74a. Does/did this partner drink alcohol before sex? |__| {ALCBSXPT1}

Yes 1

No 2 -------- > **Q.75**

Q.74b **IF YES** to 74a: Is/was it sometimes or always? |__|{ALCSXPT1}

Sometimes/Inconsistent 2

Always/Often 3

DK 7

Q.74c.When your partner drinks/drank alcohol, and have/had sex with you,does/did she usually have the following effects **(Prompted )**

Yes No NR

Unsteady gait 1 2 9 {ALCGAPT1}

Fall over 1 2 9 {ALCFAPT1}

Get angry/violent 1 2 9 {ALCVIPT1}

Difficulty speaking 1 2 9 {ALCASPPT1}

Other 1 2 9 {ALOTHPT1}

Specify) ________________________ { OTHALCPT1}

Q.75 How likely do you think it is that this partner has been exposed to HIV

(virus which causes AIDS)? (**prompted**)

Very likely 1 |__|{PAIDSRSK1}

Somewhat likely 2

Unlikely 3

Not at all 4

Don't Know 7

Q76. In the last 12 months has this partner informed you of her HIV sero-status? |___| {INFPHIV1}

Yes 1

No 2

Never tested/got HIV results 3

Received Couple counseling 4

DR 7

NA 8

Q.77. In the last 12 months have you informed this partner of your HIV

sero-status? |___| {INFYPHIV1}

Yes 1

No 2

Never tested/got HIV results 3

Received couple counseling 4

DR 7

NA 8

**SECOND BLOCK**

"Can you tell me about the sexual partner just prior to the one we just discussed?"

**(In the last twelve months)**

Q.78 Have you had any other sexual partners in the past 12 months other

than the one we just discussed? Yes 1 |__|{MORE2}

No 2---->**Q.168**

Q.79 Remembering the most recent time you had sex, what

was your relationship to that partner at that time?

Current wife(at the time) 01 |__|__| {RLTN2}

Current consensual partner( at the time) 02

Former wife/consensual 03

Girlfriend 04

Occasional or casual friend 05

Visitor (incl. wedding/funeral) 06

Stranger 07

Workmate 08

Boss/work supervisor 09

Employee 10

Fellow student 11

Sugar mummy 12

Relative other than spouse (specify) 13 {OTHRLTN2}

Other non relative **(specify)________________** 14 {OTHRLNR2}

Don't Know 97

Q.80 What are/were your partner's main occupations?

**(Record answer(s) as given):**__________________**(OCCUPATION 1)**

__________________**(OCCUPATION 2)**

**(Code up to two responses, code first mentioned** **occupation first.)**

Agriculture for home use/barter 01 |__|__|{OCCUP12}

Agriculture for selling 02 |__|__|{OCCUP22}

Housework in own home 03

Housekeeper (for relative or employer) 04

Home brewing 05

Government/clerical/teaching 06

Fishing 07

Student 08

Military/police 09

Shopkeeper 10

Trading/vending 11

Bar worker or owner 12

Trucker 13

Unemployed (**PROBE _ NO AGRIC OR HOUSE WORK?**) 14

Other ____________________________ 15

No additional response(use in 2nd field if

one occupation is cited 88

Do Not Know 97

Q.81 How long did you know this person before you had sex with

him for the first time?[i.e from the time he/you proposed

a relationship to first sex]

**Less than 1 day code 00, Less 1 week** **code** days |__|__|{COTDYSP2}

**Less 1 month code weeks** weeks |__|__|{COTWKSP2}

**Less 1 year code months** months |__|__|{COTMOSP2}

**If 1 year or more code completed years**  years |__|__|{COTYRSP2}

**(If DK Code 97 in days and 98 in other boxes; 99 for NR)**

Q.82 How long ago did you first have sex with this person?

**Less than 1 day code 00, Less than 1 week code** days |__|__|{DAYS2}

**Less than one month code weeks** weeks |__|__|{WEEKS2}

**Less than one year code months** months |__|__|{MONTHS2}

**If 1 year or more code completed Years** Years |__|__|{YEARS2}

**(If DK Code 97 in days and 98 in other boxes; 99 for NR)**

Q.83 Are you still in a sexual relationship with her?

Yes 1 |__| {RLTONGO2}

No 2

Don't know 7

**During the last time you were having sexual relations with this**

**partner, please tell me how often you usually had intercourse.**

**For Qs. 84-87a If Don't know write DK and**

**no response** **write NR besides the box**

Q.84 When you had/have sex, how many times per day did/do you usually

have sexual intercourse? **(code # of times per day)**  |__| {TIMDAY2}

Q.85 How many days per week did/do you usually have sexual intercourse?

**(code # of days per week)** |__|{DAYSWEK2}

Q.86 How many weeks per month did/do you usually have sexual

intercourse? **(code # of weeks per months)** |__|{WKSMON2}

Q. 87a How many months in the past 12 months did/have you had sexual

intercourse? **(Code # of months in the past 12 months)** |__|__| {MTSPYR2}

( **IF RELATIONSHIP ENDED MORE THAN 30 DAYS AGO**

**SKIP TO Q.88**)

Q.87 b In the past 7 days how many times did you have sexual intercourse

with this person? |___|___|{SFREQWK2}

**[Record actual #, code 97 for DK, 98 for NA, 99 for NR]**

Q.87 c In the past 30 days how many times did you have sexual intercourse

with this person? |___|___|{SFREQMN2}

**[Record actual #, code 97 for DK, 98 for NA, 99 for NR]**

Q.88 Is/was she older, younger, or about the same age ?

Older 1 |__| {RLTNAGE2}

Younger 2

Same age 3--->**Q.90**

DK 7----->**Q.90**

Q.89 About how many years [older/younger] ? |__|__|{RLTNYRS2}

**(Record actual # or 97=don't know)**

Q.90 Does(was) she live(living) in this household ? |__|{RLTNHH2}

Yes 1 >**Q.93**

No 2

Q.91 Does(was)she regularly live(living) in this community? |__| {RLTNCM2}

Yes 1>**Q.93**

No 2

DK 7

Q.92 How far away does/was she normally live/living?

Within one hour’s walk or 3 miles 1 |__| {RLTNRES2}

More than one hour's walk or >3 miles 2

Don't Know 7

Q.93 Have you and this partner ever used a condom? |__|{CNDEVER2}

Yes 1

No 2---------------->**Q.96**

NR 9

Q.94 During the most recent 12 months you were having sexual relationship

with this partner, how often did you use condoms? |__|{RNYRCON2}

Never 1**---------------->Q.96**

Sometimes/inconsistent 2

Always 3

DK 7----------------> **Q.96**

Q.95a Why did you use condoms with this partner?**(UNPROMPTED)**

Yes No

Prevent HIV transmission/acquisition/reinfection 1 2 {CONDHIV2}

Prevent STD acquisition/transmission 1 2 {CONDSTD2}

For family planning 1 2 {CONDFP2}

Just trying it out 1 2 {CONDTRY2}

Other________________________ 1 2 {CONDOTH2}

Q.95b Did you use a condom the last time you had sex with that partner?

Yes 1 |__|{RLTNLST2}

No 2

DK/DR 7

Q95c During the the last 30 days you were having a sexual relationship |__|{RNMTCON2}

with this partner, how often did you use condoms?(PROMPTED)

Never 1

Sometime/inconsistent 2

Always 3------------>96

Dk 4------------>96

Q.95d In the most recent 30 days what are the reasons you did not use

/inconsistently used condoms with this partner?

Do not know how to put on a condom 01 |__|__|{PCURNOCO12}

Want to have a child/children 02 |__|__|{PCURNOCO22}

We have both tested negative for HIV 03 |__|__|{PCURNOCO32}

Condoms reduce sexual satisfaction 04

Condom use is against our religious beliefs 05

Condom use is against our traditional beliefs 06

I trust my partner(partner is faithful) 07

My partner trusts me 08

Do not trust condoms 09

Do not know where to get condoms 10

Cannot afford condoms 11

Don’t feel comfortable getting condoms in our community 12

Condom use is not compatible with our sexual style 13

The condoms do not fit properly 14

Partner refused 15

Other____________________ 16

Partner does not have HIV/STD 17

I don’t have HIV/STD 18

Q.96 Have you ever discussed condom use with this partner?

Yes 1 |__|{CNDISCP2}

No 2

DR/DK 7

Q.97 In the last 12 months while you were having a relationship with this person,

did this partner have sexual relations/ intercourse with persons other

than yourself?

Yes 1 |__| {RLNSXOT2}

No 2>**Q.99**

DK 7>**Q.99**

Q.98 **If yes in Q.97;** How many other sexual partners other than yourself, do

you think your partner had sex with in the last 12 months, while you

were in a sexual relationship? |__|__|{RLTPNO2}

Q.99 How long ago, did you last have sex with this person?

**CURRENT ID**

**-------/-------/-------/-------**

**COMPUTER ID**

**-------------------------------------**

**Less than 1 day code 00 days, less than 1 week code** days |__|__|{RLDYSLT2

**Less than 1 month code weeks**  weeks |__|__|{RLWKSLT2

**Less than 1 year code months** months|__|__|{RLMOSLT2

**(If DK Code 97 in days and 98 in other boxes; 99 for NR)**

Q.100a When you have/had sex with this partner do/did you wash your

genitals before sexual intercourse?

Yes 1 |__| [WSHBFSX2]

No 2 -----------> **Q.101a**

Q.100b. **IF ‘YES’,** How frequently? (Unprompted)

Sometimes 1 |__| [FQWSHBF2]

Usually 2

Always 3

Q.100c. Generally, how soon before sexual intercourse?

**If less than 1 minute, code 00 minutes, If less than 1 hour (60 min),**

**code completed minutes If less than 1 day, code completed hours,**

**If one day or more, code days.**

**Minutes** |__|__| [MINBFSX2]

**Hours** |__|__| [HRBFSX2]

**Days** |__|__|[DAYBFSX2]

Q.101a. When you have sex with this partner do you wash your genitals

after sex? Yes 1 |__| [WSHAFTS2]

No 2 ------------->**Q.102**

Q.101b **IF ‘YES’,** How frequently? (Unprompted)

Sometimes 1 |__| [FQWSHAF2]

Usually 2

Always 3

Q101c. **IF ‘YES’;** How soon after?

**If less than 1 minute, code 00 minutes, If less than 1 hour (60 min),**

**code completed minutes, If less than 1 day, code completed hour.**

**MINUTES** |__|__| [MINAFTS2]

**HOURS**  |__|__|[HRAFTS2]

Q.102. When you have/had sex with this partner what do you use to clean your

genitals after sex?

Piece of cloth 1 |__|{CLINGEN12}

Washing 2 |__|{CLINGEN22}

Piece of cloth and washing 3

Other specify_______________ 4

None 5

No additional response 8

Q.103a. Do/did you drink alcohol before sex with this partner? |__| {ALCSF2}

Yes 1

No 2 ------------- >**Q.104a**

Q.103b **IF YES** to Q.103a ask: Is/was it sometimes or always? |__|{ALCSXSF2}

Sometimes/Inconsistent 2

Always/Often 3

DK 7

Q.103c. During the time you took alcohol and have/had sex with this partner

do/did you usually have the following effects? (**PROMPT**)

Yes No NR

Unsteady gait 1 2 9 {ALUNGSF2}

Fall over 1 2 9 {ALFOSF2}

Get angry/violent 1 2 9 {ALANVSF2}

Difficulty speaking 1 2 9 {ALDSPSF2}

Other 1 2 9 {ALOTHSF2}

Specify _____________________ {OTHALSF2}

Q.104a. Does/did this partner drink alcohol before sex? |__| {ALCBSXPT2}

Yes 1

No 2 ------------>**Q.105**

Q.104b **IF YES** to Q.104a ask: Is/was it sometimes or always? |__|{ALCSXPT2}

Sometimes/Inconsistent 2

Always/Often 3

DK 7

Q.104c. When your partner drinks/drank alcohol, and have/had sex with you,

does/did she usually have the following effects **(Prompted )**

Yes No NR

Unsteady gait 1 2 9 {ALCGAPT2}

Fall over 1 2 9 {ALCFAPT2}

Get angry/violent 1 2 9 {ALCVIPT2}

Difficulty speaking 1 2 9 {ALCASPPT2}

Other 1 2 9 {ALOTHPT2}

Specify) _____________________ { OTHALCPT2}

Q.105 How likely do you think it is that this partner has been exposed to HIV

(virus which causes AIDS)? (**prompted**)

Very likely 1 |__|{PAIDSRSK2}

Somewhat likely 2

Unlikely 3

Not at all 4

Don't Know 7

Q.106 In the last 12 months has this partner informed you of her HIV sero-status? |___|{INFPHIV2}

Yes 1

No 2

Never tested/got HIV results 3

Received Couple counseling 4

DK/DR 7

Q. 107 In the last 12 months have you informed this partner of your HIV

sero-status? |___|{INFYPHIV2}

Yes 1

No 2

Never tested/got HIV results 3

Received couple counseling 4

DK/DR 7

**THIRD BLOCK**

"Can you tell me about the sexual partner just prior to the one we just discussed?"

**(In the last twelve month)**

Q.108 Have you had any other sexual partners in the past year other than the

one we just discussed?

Yes 1 |__|{MORE3}

No 2----->**Q.168**

Q.109 Remembering the most recent time you had sex, what

was your relationship to that partner at that time?

Current wife(at the time) 01 |__|__| {RLTN3}

Current consensual partner( at the time) 02

Former wife/consensual 03

Girlfriend 04

Occasional or casual friend 05

Visitor (incl. wedding/funeral) 06

Stranger 07

Workmate 08

Boss/work supervisor 09

Employee 10

Fellow student 11

Sugar mummy 12

Relative other than spouse (specify) 13 {OTHRLTN3}

Other non relative **(specify)________________** 14 {OTHRLNR3}

Don't Know 97

Q.110 What are/were your partner's main occupations?

**(Record answer(s) as given):**__________________**(OCCUPATION 1)**

__________________**(OCCUPATION 2)**

**(Code up to two responses, code first mentioned** **occupation first.)**

Agriculture for home use/barter 01 |__|__|{OCCUP13}

Agriculture for selling 02 |__|__|{OCCUP23}

Housework in own home 03

Housekeeper (for relative or employer) 04

Home brewing 05

Government/clerical/teaching 06

Fishing 07

Student 08

Military/police 09

Shopkeeper 10

Trading/vending 11

Bar worker or owner 12

Trucker 13

Unemployed (**PROBE _ NO AGRIC OR HOUSE WORK?**) 14

Other ____________________________ 15

No additional response(use in 2nd field if one occupation is cited 88

Do Not Know 97

Q.111 How long did you know this person before you had sex with him for the

first time? [i.e from the time he/you proposed a relationship to first sex]

**Less than 1 day code 00, Less 1 week code** days |__|__|{COTDYSP3}

**Less 1 month code weeks** weeks |__|__|{COTWKSP3}

**Less 1 year code months** months |__|__|{COTMOSP3}

**If 1 year or more code completed years** years |__|__|{COTYRSP3}

**(If DK Code 97 in days and 98 in other boxes; 99 for NR)**

Q.112 How long ago did you first have sex with this person?

**Less than 1 day code 00, Less than 1 week code**  days |__|__|{DAYS3}

**Less than one month code weeks**  weeks |__|__|{WEEKS3}

**Less than one year code months**  months |__|__|{MONTHS3}

**If 1 year or more code completed Years**  years |__|__|{YEARS3}

**(If DK Code 97 in days and 98 in other boxes; 99 for NR)**

Q.113 Are you still in a sexual relationship with her?

Yes 1 |__| {RLTONGO3}

No 2

DK 7

**During the last time you were having sexual relations with this partner,**

**please tell me how often you usually had intercourse.**

**For Qs. 114-117a If Don't know write DK and no response**

**write NR besides the box**

Q.114 When you had/have sex, how many times per day did/do you usually

have sexual intercourse? **(code # of times per day)**  |__| {TIMDAY3}

Q.115 How many days per week did/do you usually have sexual intercourse?

**(code # of days per week)** |__|{DAYSWEK3}

Q.116 How many weeks per month did/do you usually have sexual

intercourse? **(code # of weeks per months)** |__|{WKSMON3}

Q. 117a How many months in the past 12 months did/have you had sexual

intercourse? **(Code # of months in the past 12 months)** |__|__|{MTSPYR3}

**IF RELATIONSHIP ENDED >30 DAYS AGO SKIP TO Q.118**

Q.117 b In the past 7 days how many times did you have sexual intercourse

with this person?

**[Record actual #, code 97 for DK, 98 for NA, 99 for NR]** |___|___|{SFREQWK3}

Q.117 c In the past 30 days how many times did you have sexual intercourse

with this person?

**[Record actual #, code 97 for DK, 98 for NA, 99 for NR]** |___|___|{SFREQMN3}

Q.118 Is/was she older, younger, or about the same age ?

Older 1 |__|{RLTNAGE3}

Younger 2

Same age 3----->**Q.120**

DK 7------->**Q.120**

Q.119 About how many years [older/younger] ? |__|__|{RLTNYRS3}

**(Record actual # or 97=don't know)**

Q.120 Does(was) she live(living) in this household ? |__|{RLTNHH3}

Yes 1------->**Q.123**

No 2

Q.121 Does(was)she regularly live(living) in this community? |__| {RLTNCM3}

Yes 1---->**Q.123**

No 2

DK 7

Q.122 How far away does/was she normally live/living?

Within one hour’s walk or 3 miles 1 |__| {RLTNRES3}

More than one hour's walk or >3 miles 2

Don't Know 7

Q.123 Have you and this partner ever used a condom? |__|{CNDEVER3}

Yes 1

No 2------------->**Q.126**

NR 9

Q.124 During the most recent 12 months you were having sexual relationship

with this partner, how often did you use condoms? |__|{RNYRCON3}

Never 1**------------>Q.126**

Sometimes/inconsistent 2

Always 3

DK 7----------->**Q.126**

Q.125a Why did you use condoms with this partner?(UNPROMPTED)

Yes No

Prevent HIV transmission/acquisition/reinfection 1 2 {CONDHIV3}

Prevent STD acquisition/transmission 1 2 {CONDSTD3}

For family planning 1 2 {CONDFP3}

Just trying it out 1 2 {CONDTRY3}

Other________________________ 1 2 {CONDOTH3}

Q.125b Did you use a condom the last time you had sex with that partner?

Yes 1 |__|{RLTNLST3}

No 2

DK/DR 7

Q125c During the the last 30 days you were having a sexual relationship | |__|{MTCON3}

with this partner, how often did you use condoms?(PROMPTED)

Never 1

Sometime/inconsistent 2

Always 3------------>**Q.126**

Dk 4------------>**Q.126**

Q.125d In the most recent 30 days what are the reasons you did not use

/inconsistently used condoms with this partner?

Do not know how to put on a condom 01 |__|__|{PCURNOCO13}

Want to have a child/children 02 |__|__|{PCURNOCO23}

We have both tested negative for HIV 03 |__|__|{PCURNOCO33}

Condoms reduce sexual satisfaction 04

Condom use is against our religious beliefs 05

Condom use is against our traditional beliefs 06

I trust my partner(partner is faithful) 07

My partner trusts me 08

Do not trust condoms 09

Do not know where to get condoms 10

Cannot afford condoms 11

Don’t feel comfortable getting condoms in our community 12

Condom use is not compatible with our sexual style 13

The condoms do not fit properly 14

Partner refused 15

Other____________________ 16

Partner does not have HIV/STD 17

I don’t have HIV/STD 18

Q.126 Have you ever discussed condom use with this partner?

Yes 1 |__|{CNDISCP3}

No 2

DR/DK 7

Q.127 In the last 12 months while you were having a relationship with this

person, did this partner have sexual relations/ intercourse with persons

other than yourself?

Yes 1 |__| {RLNSXOT3}

No 2>**Q.129**

DK 7>**Q.129**

Q.128 **If yes in Q.127;** How many other sexual partners other than yourself,

do you think your partner had sex with in the last 12 months, while you

were in a sexual relationship? |__|__|{RLTPNO3}

Q.129 How long ago, did you last have sex with this person?

**COMPUTER ID**

**-----------------------------------**

**CURRENT ID**

**-------/-------/-------/-------**

**Less than 1 day code 00 days, less than 1 week code** days |__|__|{RLDYSLT3

**Less than 1 month code weeks** weeks |__|__|{RLWKSLT3

**Less than 1 year code months**  months|__|__|{RLMOSLT3

**(If DK Code 97 in days and 98 in other boxes; 99 for NR)**

Q.130a When you have/had sex with this partner do/did you wash your

genitals before sexual intercourse?

Yes 1 |__| [WSHBFSX3]

No 2 ----------->**Q.131a**

Q.130b. **IF ‘YES’,** How frequently? (Unprompted)

Sometimes 1 |__| [FQWSHBF3]

Usually 2

Always 3

Q.130c. Generally, how soon before sexual intercourse?

**If less than 1 minute, code 00 minutes, If less than 1 hour (60 min),**

**code completed minutes If less than 1 day, code completed hours,**

**If one day or more, code days.**

**Minutes** |__|__| [MINBFSX3]

**Hours** |__|__| [HRBFSX3]

**Days** |__|__|[DAYBFSX3]

Q.131a. When you have sex with this partner do you wash your genitals after sex?

Yes 1 |__| [WSHAFTS3]

No 2 ------------->**Q.132**

Q.131b **IF ‘YES’,** How frequently? (**UNPROMPTED**)

Sometimes 1 |__| [FQWSHAF3]

Usually 2

Always 3

Q131c. **IF ‘YES’;**How soon after?

**If less than 1 minute, code 00 minutes, If less than 1 hour (60 min),**

**code completed minutes, If less than 1 day, code completed hour.**

**MINUTES** |__|__| [MINAFTS3]

**HOURS** |__|__|[HRAFTS3]

Q.132. When you have/had sex with this partner what do you use to clean your

genitals after sex?

Piece of cloth 1 |__|{CLNGEN13} Washing 2 |__|{CLNGEN23}

Piece of cloth and washing 3

None 5

Other specify_______________4

No additional response 8

Q.133a. Do/did you drink alcohol before sex with this partner? |__|{ALCSF3}

Yes 1

No 2 ------------ >**Q.134a**

Q.133b **IF YES**, is/was it sometimes or always? |__|{ALCSXSF3}

Sometimes/Inconsistent 2

Always/Often 3

DK 7

Q.133c. During the time you took alcohol and have/had sex with this partner

do/did you usually have the following effects? **(PROMPT)**

Yes No NR

Unsteady gait 1 2 9 {ALUNGSF3}

Fall over 1 2 9 {ALFOSF3}

Get angry/violent 1 2 9 {ALANVSF3}

Difficulty speaking 1 2 9 {ALDSPSF3}

Other 1 2 9 {ALOTHSF3}

Specify _____________________ {OTHALSF3}

Q.134a. Does/did this partner drink alcohol before sex? |__| {ALCBSXPT3}

Yes 1

No 2 ------------ >**Q.135**

Q.134b **IF YES**, is/was it sometimes or always? |__|{ALCSXPT3}

Sometimes/Inconsistent 2

Always/Often 3

DK 7

Q.134c. When your partner drinks/drank alcohol, and have/had sex with you,

does/did he usually have the following effects **(PROMPTED )**

Yes No NR

Unsteady gait 1 2 9 {ALCGAPT3}

Fall over 1 2 9 {ALCFAPT3}

Get angry/violent 1 2 9 {ALCVIPT3}

Difficulty speaking 1 2 9 {ALCASPPT3}

Other 1 2 9 {ALOTHPT3}

Specify) _____________________ { OTHALCPT3}

Q.135 How likely do you think it is that this partner has been exposed to HIV

(virus which causes AIDS)? (**prompted**)

Very likely 1 |__|{PAIDSRSK3}

Somewhat likely 2

Unlikely 3

Not at all 4

Don't Know 7

Q.136 In the last 12 months has this partner informed you of her HIV sero-status? |___| {INFPHIV3}

Yes 1

No 2

Never tested/got HIV results 3

Received Couple counseling 4

DR 7

NA 8

Q. 137 In the last 12 months have you informed this partner of your HIV

sero-status? |___| {INFYPHIV3}

Yes 1

No 2

Never tested/got HIV results 3

Received couple counseling 4

DR 7

NA 8

**FOURTH BLOCK**

"Can you tell me about the sexual partner just prior to the one we just discussed?"

**(In the last twelve month)**

Q.138 Have you had any other sexual partners in the past year |__|{MORE4}

other than the one we just discussed? Yes 1

No 2>**Q.168**

Q.139 Remembering the most recent time you had sex, what

was your relationship to that partner at that time?

Current wife(at the time) 01 |__|__| {RLTN4}

Current consensual partner( at the time) 02

Former wife/consensual 03

Girlfriend 04

Occasional or casual friend 05

Visitor (incl. wedding/funeral) 06

Stranger 07

Workmate 08

Boss/work supervisor 09

Employee 10

Fellow student 11

Sugar mummy 12

Relative other than spouse (specify) 13 {OTHRLTN4}

Other non relative **(specify)________________** 14 {OTHRLNR4}

Don't Know 97

Q.140 What are/were your partner's main occupations?

**(Record answer(s) as given):**__________________**(OCCUPATION 1)**

__________________**(OCCUPATION 2)**

**(Code up to two responses, code first mentioned** **occupation first.)**

Agriculture for home use/barter 01 |__|__|{OCCUP14}

Agriculture for selling 02 |__|__|{OCCUP24}

Housework in own home 03

Housekeeper (for relative or employer) 04

Home brewing 05

Government/clerical/teaching 06

Fishing 07

Student 08

Military/police 09

Shopkeeper 10

Trading/vending 11

Bar worker or owner 12

Trucker 13

Unemployed (**PROBE _ NO AGRIC OR HOUSE WORK?**) 14

Other ____________________________ 15

No additional response(use in 2nd field if

one occupation is cited 88

Do Not Know 97

Q.141 How long did you know this person before you had sex with him for the

first time? [i.e from the time he/you proposed a relationship to first sex]

**Less than 1 day code 00, Less 1 week** **code**  days |__|__|{COTDYSP4}

**Less 1 month code weeks** weeks |__|__|{COTWKSP4}

**Less 1 year code months** months |__|__|{COTMOSP4}

**If 1 year or more code completed years** years |__|__|{COTYRSP4}

**(If DK Code 97 in days and 98 in other boxes; 99 for NR)**

Q.142 How long ago did you first have sex with this person?

**Less than 1 day code 00, Less than 1 week code** days |__|__|{DAYS4}

**Less than one month code weeks** weeks |__|__|{WEEKS4}

**Less than one year code months** months |__|__|{MONTHS4}

**If 1 year or more code completed Years** Years |__|__|{YEARS4}

**(If DK Code 97 in days and 98 in other boxes; 99 for NR)**

Q.143 Are you still in a sexual relationship with her?

Yes 1 |__| {RLTONGO4}

No 2

DK 7

**During the last time you were having sexual relations with this**

**partner, please tell me how often you usually had intercourse.**

**For Qs. 144,145,& 146 If Don't know write DK and**

**no response** **write NR besides the box**

Q.144 When you had/have sex, how many times per day did/do you usually

have sexual intercourse? **(code # of times per day)**  |__| {TIMDAY4}

Q.145 How many days per week did/do you usually have sexual intercourse?

**(code # of days per week)** |__|{DAYSWEK4}

Q.146 How many weeks per month did/do you usually have sexual

intercourse? **(code # of weeks per months)** |__|{WKSMON4}

Q. 147a How many months in the past 12 months did/have you had sexual

intercourse? **(Code # of months in the past 12 months)**  |__|__| {MTSPYR4}

**IF RELATIONSHIP ENDED>30 DAYS AGO SKIP TO Q.148**

Q.147 b In the past 7 days how many times did you have sexual intercourse

with this person?

**[Record actual #, code 97 for DK, 98 for NA, 99 for NR]** |___|___|{SFREQWK4}

Q.147 c In the past 30 days how many times did you have sexual intercourse

with this person? |___|___|{SFREQMN4}

**[Record actual #, code 97 for DK, 98 for NA, 99 for NR]**

Q.148 Is/was she older, younger, or about the same age ?

Older 1 |__| {RLTNAGE4}

Younger 2

Same age 3------>**Q.150**

DK 7------>**Q.150**

Q.149 About how many years [older/younger] ? |__|__|{RLTNYRS4}

**(Record actual # or 97=don't know)**

Q.150 Does(was) she live(living) in this household ? |__|{RLTNHH4}

Yes 1 ------>**Q.153**

No 2

Q.151 Does(was)she regularly live(living) in this community? |__| {RLTNCM4}

Yes 1--->**Q.153**

No 2

DK 7

Q.152 How far away does/was she normally live/living?

Within one hour’s walk or 3 miles 1 |__| {RLTNRES4}

More than one hour's walk or >3 miles 2

Don't Know 7

Q.153 Have you and this partner ever used a condom? |__|{CNDEVER4}

Yes 1

No 2--------------->**Q.156**

NR 9

Q.154 During the most recent 12 months you were having sexual relationship

with this partner, how often did you use condoms? |__|{RNYRCON4}

Never 1**--------------->Q.156**

Sometimes/inconsistent 2

Always 3

DK 7

Q.155a Why did you use condoms with this partner?(UNPROMPTED)

Yes No

To prevent HIV transmission/acquisition/reinfection 1 2 {CONDHIV4}

TO prevent STD acquisition/transmission 1 2 {CONDSTD4}

For family planning 1 2 {CONDFP4}

Just trying it out 1 2 {CONDTRY4}

Other________________________ 1 2 {CONDOTH4}

Q.155b Did you use a condom the last time you had sex with that partner?

Yes 1 |__|{RLTNLST4}

No 2

DK/DR 7

Q155c During the the last 30 days you were having a sexual relationship |__|{MTCON4}

with this partner, how often did you use condoms?(PROMPTED)

Never 1

Sometime/inconsistent 2

Always 3------------>**Q.156**

Dk 4------------>**Q.156**

Q.155d In the most recent 30 days what are the reasons you did not use

/inconsistently used condoms with this partner?

Do not know how to put on a condom 01 |__|__|{PCURNOCO14}

Want to have a child/children 02 |__|__|{PCURNOCO24}

We have both tested negative for HIV 03 |__|__|{PCURNOCO34}

Condoms reduce sexual satisfaction 04

Condom use is against our religious beliefs 05

Condom use is against our traditional beliefs 06

I trust my partner(partner is faithful) 07

My partner trusts me 08

Do not trust condoms 09

Do not know where to get condoms 10

Cannot afford condoms 11

Don’t feel comfortable getting condoms in our community 12

Condom use is not compatible with our sexual style 13

The condoms do not fit properly 14

Partner refused 15

Other____________________ 16

Partner does not have HIV/STD 17

I don’t have HIV/STD 18

Q.156 Have you ever discussed condom use with this partner?

Yes 1 |__|{CNDISCP4}

No 2

DK 7

Q.157 In the last 12 months while you were having a relationship with this

person, did this partner have sexual relations/ intercourse with persons

other than yourself?

Yes 1 |__| {RLNSXOT2}

No 2--->Q.159

DK 7----->Q.159

Q.158 **If yes in Q.157;** How many other sexual partners other than yourself,

do you think your partner had sex with in the last 12 months, while

you were in a sexual relationship? |__|__|{RLTPNO2}

Q.159 How long ago, did you last have sex with this person?

**CURRENT ID**

**-------/-------/-------/-------**

**COMPUTER ID**

**-------------------------------------**

**Less than 1 day code 00 days, less than 1 week code**  days |__|__|{RLDYSLT4

**Less than 1 month code weeks** weeks |__|__|{RLWKSLT4

**Less than 1 year code months** months|__|__|{RLMOSLT4

**If one year or more code completed years**  year |__|__|{RLYRSLT4

**(If DK Code 97 in days and 98 in other boxes; 99 for NR)**

Q.160a When you have/had sex with this partner do/did you wash your

genitals before sexual intercourse?

Yes 1 |__| [WSHBFSX4]

No 2 -----------> **Q.161a**

Q.160b. **IF ‘YES’,** How frequently? **(Unprompted)**

Sometimes 1 |__| [FQWSHBF4]

Usually 2

Always 3

Q.160c. Generally, how soon before sexual intercourse?

**If less than 1 minute, code 00 minutes, If less than 1 hour (60 min),**

**code completed minutes If less than 1 day, code completed hours,**

**If one day or more, code days.**

**Minutes** |__|__| [MINBFSX4]

**Hours** |__|__| [HRBFSX4]

**Days**  |__|__|[DAYBFSX4]

Q.161a. When you have sex with this partner do you wash /wipe your genitals

after sex?

Yes 1 |__| [WSHAFTS4]

No 2 ----------->**Q.162**

Q.101b **IF ‘YES’,** How frequently? (Unprompted)

Sometimes 1 |__| [FQWSHBF4]

Usually 2

Always 3

Q101c. **IF ‘YES’;** How soon after?

**If less than 1 minute, code 00 minutes, If less than 1 hour (60 min),**

**code completed minutes, If less than 1 day, code completed hour.**

**MINUTES** |__|__| [MINAFTS4]

**HOURS**  |__|__|[HRAFTS4]

Q.162. When you have/had sex with this partner what do you use to clean your

genitals after sex?

Piece of cloth 1 |__|{CLNGEN14}

Washing 2 |__|{CLNGEN24}

Piece of cloth and washing 3

Other specify_______________4

No additional response 8

None 5

Q.163a. Do/did you drink alcohol before sex with this partner? |__|{ALCSF4}

Yes 1

No 2 ----------- >**Q.164a**

Q.163b **IF YES**, is/was it sometimes or always? |__|{ALCSXSF4}

Sometimes/Inconsistent 2

Always/Often 3

DK 7

Q.163c. During the time you took alcohol and have/had sex with this partner

do/did you usually have the following effects? **(PROMPT)**

Yes No NR

Unsteady gait 1 2 9 {ALUNGSF4}

Fall over 1 2 9 {ALFOSF4}

Get angry/violent 1 2 9 {ALANVSF4}

Difficulty speaking 1 2 9 {ALDSPSF4}

Other 1 2 9 {ALOTHSF4}

Specify _____________________ {OTHALSF4}

Q.164a. Does/did this partner drink alcohol before sex? |__|{ALCBSXPT4}

Yes 1

No 2 ------------ >**Q.105**

Q.164b **IF YES**, is/was it sometimes or always? |__|{ALCSXPT4}

Sometimes/Inconsistent 2

Always/Often 3

DK 7

Q.164c. When your partner drinks/drank alcohol, and have/had sex with you,

does/did he usually have the following effects **(Prompted )**

Yes No NR

Unsteady gait 1 2 9 {ALCGAPT4}

Fall over 1 2 9 {ALCFAPT4}

Get angry/violent 1 2 9 {ALCVIPT4}

Difficulty speaking 1 2 9 {ALCASPPT4}

Other 1 2 9 {ALOTHPT4}

Specify) _____________________ { OTHALCPT4}

Q.165 How likely do you think it is that this partner has been exposed to HIV

(virus which causes AIDS)? (**prompted**)

Very likely 1 |__|{PAIDSRSK4}

Somewhat likely 2

Unlikely 3

Not at all 4

Don't Know 7

Q.166 In the last 12 months has this partner informed you of her HIV sero-status? |___| {INFPHIV4}

Yes 1

No 2

Never tested/got HIV results 3

Received Couple counseling 4

DR 7

NA 8

Q. 167 In the last 12 months have you informed this partner of your HIV

sero-status? |___| {INFYPHIV4}

Yes 1

No 2

Never tested/got HIV results 3

Received couple counseling 4

DR 7

NA 8

**END OF REPETITIVE SEXUAL BLOCKS**

**SKIP TO Q.169a IF HAD NEVER HAD SEX**

Q.168 In the last 12 months, have you ever had sex in a situation in which

money or gifts were exchanged? Yes 1 |__|{SEXGIFT}

No 2

Q.168b Do you prefer to have sexual intercourse when your partner’s vagina is:

**PROMPT** |__|{VAGSTAT}

Dry during intercourse 1

Wet during intercourse 2

Other preference(specify)____________________________ 3 {OTHRVGSTmemo}

No preference 4

Q.169 (a) Do you drink alcohol? Yes 1 |__| {DRINKALC}

No 2-------------->**Q. 170**

Q. 169 (b) When you drink alcohol, do you usually have/get the following?

**PROMPT**

Yes No N/R

Unsteady gait 1 2 9 {SALCGAIT}

Fall over 1 2 9 {SALCFALL}

Get angry/violent 1 2 9 {SALCVIOL}

Dfficulty in speaking 1 2 9 {SALCSPEA}

Other 1 2 9 {SALCOTHE}

Specify______________________ {OTHESALC}

Q.169 (c) In the last 30 days, have you drunk alcohol? Yes 1 |__| {ALCLSTMO}

No 2

NR 9

Q.170 In the last 12 months have you had any of the following health

problems? **PROMPTED:**  (Code in since last 12months column)

**If he had STD symptoms ask:**

Do you have these symptoms now or have you had them over the

past 7 days (Code in current Column)

**If he had GUD in past 12 months ask:** How many episodes in the past 12 months

**Last 12 months Current**

**Yes No DK Yes No DK**

Genital ulcer 1 2 7{SXM1} 1 2 7{CUR1} |__|__|{GUDPY}

Genital discharge 1 2 7{SXM2} 1 2 7{CUR2}

Frequent urination 1 2 7{SXM6} 1 2 7{CUR6}

Painful urination 1 2 7{SXM7} 1 2 7{CUR7}

Pain during intercourse 1 2 7{SXM8} 1 2 7{CUR8}

Bleeding during intercourse 1 2 7{SXM9} 1 2 7{CUR9}

Lower abdominal pain 1 2 7{SXM10} 1 27{CUR10}

Genital warts 1 2 7{SXM11} 1 2 7{CUR11}

**IF NO TO ALL THE ABOVE SKIP TO Q.179**

**IF RESPONDENT REPORTED STD SYMPTOM EITHER CURRENTLY**

**(LAST 7 DAYS)** **OR PAST 12 MONTHS, ASK DURATION FOR** **THE MOST**

**RECENTLY HEALED EPISODE:** **IF RESPONDENT HAD ONLY ONE OF THE**

**FOLLOWING AILMENTS CODE 98 FOR THE REST**

**For respondents who had GUD ask:**

Q.171 **For respondents who had GUD ask:**

How long did the most recent healed Ulcer last?

**If < 1 week code days** Days |__|__|{GUDAYS}

**If < 1 month code Weeks** Weeks |__|__|{GUWEEKS}

**If < 1 year code Months** Months |__|__|{GUMONTHS}

**code completed months)**

**If DK for all, code 97 in days and 98 in the rest)**

Q.172 **For respondents who had painful urination ask**:

How long did the painful urination last until it cured?

**If < 1 week code days** Days |__|__|{PANDAYS}

**If < 1 month code weeks** Weeks |__|__|{PANWEEKS}

**If < 1 year code months**  Months |__|__|{PANMNTHS}

**If DK for all, code 97 in days and 98 in the rest)**

**For respondents who had urethral** **discharge ask:**

Q.173 **For respondents who had urethral** **discharge ask:**

How long did you have this discharge before it went away?

**If < 1 week code days** Days |__|__|{DISDAYS}

**If < 1 month code Weeks** Weeks |__|__|{DISWEEKS}

**If < 1 year code Months** Months |__|__|{DISMNTHS}

**If DK for all, code 97 in days and 98 in the rest)**

Q.174 Did you/ have you had sexual activity while suffering from these

symptoms |__|{SYMPSEX}

Yes 1

No 2

DK 7

Q.175 Did you tell your spouse(s) or any partner(s) about these symptoms?

Yes 1 |__|{SPS}

No 2

NA 8

NR 9

Q.176 Did you do anything to help cure these symptoms or to prevent passing

on infection to your spouse/partner(s)? Yes 1 |__|{TMT}

No 2 ---------->**Q.179**

NR 9----------->**Q.179**

Q.177 **If Yes to Q176** ask: What actions did you take?

**PROBE and RECORD all RESPONSES**

Yes No NR

Used condoms 1 2 9 {CON}

Abstinence 1 2 9 {ABS}

Sought treatment for self 1 2 9 {SLF}

Sought treatment for partner 1 2 9 {TMTSP}

Other action 1 2 9 {OTHRACT

Specify)_____________________ {OTHACT spec}

**IF RESPONDENT DID NOT SEEK TREATMENT FOR SELF,**

**SKIP TO Q.179**

Q.178 Where did you go for treatment?

**(PROBE and RECORD up to 3 RESPONSES)**

Pharmacy/Drug store 01 |__|__| {TRTSTD1}

Market/shop 02

Rakai Project Clinic 03 |__|__| {TRTSTD2}

Govt. doctor/nurse/clinic 04

Prvt. doctor/nurse/clinic 05 |__|__| {TRTSTD3}

Traditional healer 06

Other (specify _____________________) 07 {OTHTRTSTD memo}

Don't know 97

No additional responses 88

**I DO NOT KNOW YOUR HIV STATUS AND I'M NOT GOING** **TO ASK**

**YOU ABOUT IT BUT WE ARE INTERESTED IN HOW MANY PEOPLE**

**REQUEST AND RECEIVE THEIR HIV RESULTS FROM ANY**

**ORGANIZATION**

Q.179 Have you ever requested your HIV results from any where? |__|{HIVQUEST}

Yes 1

No 2**------->Q.185a**

N/A 8-------**>Q.185a**

NR 9-------**>Q.185a**

Q.180 Did you actually receive your HIV results? **|__|**{RHIVRLST}

Yes 1

No 2**>Q.185a**

NR 9**>Q.185a**

Q.181 From whom did you receive your HIV results? **(code up to 2 responses)**

Rakai Project 01 **|__|__|**{OGHIVTS1}

RAIN 02 **|__|__|**{OGHIVTS2}

Other NGO (Specify __________) 03

Gov't Doctor/Nurse 04

Private Doctor/Nurse/Health worker 05

Other (specify __________) 06 {OTHOGHIV}

No additional response 88

Q.182 How long ago did you last receive your HIV results?

**Less than 1 day code 00 days; <1 week code** Days |__|__|{HIVDAYS}

**Less than 1 month code**  Weeks |__|__|{HIVWKS}

**Less than 1 year code** Months |__|__|{HIVMOS}

**If 1 year or more code completed** Years|__|__|{HIVYRS}

**[If DK in all, Code 97 in days and 98 in other boxes; 99 for NR]**

Q.183 Did you discuss your HIV status with your wife or long term

consensual partner? |__|{HIVDISP}

Yes 1

No 2---------->**Q.185a**

N/A 8---------->**Q.185a**

NR 9

Q.184a What were her reactions?

Acceptance 01 |__|__|{ACTP1}

Discuss going for couple counselling 02 |__|__|{ACTP2}

Discuss going for HIV test/results 03 |__|__|{ACTP3}

Abstinence 04

Condom Use 05

Other(SPECIFY)________________ 06 {OTHACTP}

Faithfulness 07

Happy 08

Unhappy 09

Became violent 10

Abandoned me 11

**[CODE 88 FOR NO ADDITIONAL RESPONSE]**

Q.184b. Did you and your spouse/partner ever receive couples, counseling?

Yes 1 |__|{CPLECONS}

No 2------------------------>**185a**

Q.184c. When did you last receive couples’ counseling?

|__|__|{DYSCPLE}

|__|__|{WKSCPLE}

|__|__|{MTHCPLE}

|__|__|{YRSCPLE}

Q. 185a. Are you currently using sexual abstinence as a way of

preventing HIV infection? |__| {CURRABST}

(Probe abstinence as away of preventing HIV infection)

Yes 1

No 2------------->**Q.186a**

NR 9------------->**Q.186a**

Q. 185b How long ago did you take the decision to start using |__|__|{DYSABSTN}

abstinence as a way of preventing HIV? |__|__|{WKSABSTN}

|__|__|{MTHABSTN}

|__|__|{YRSABSTN}

Q. 185c. Since taking the decision have you had sex?

Yes 1 |__|{SEXABST}

No 2

Q.186a. Are you currently being faithful to your wife/ partner in

order to prevent HIV infection? |__| {CURFAITH}

Yes 1

No 2------------->**Q.187.1**

NR 9------------->**Q.187.1**

**(No sex partner currently)** NA 8-------------->**Q.187.1**

Q. 186b. Did you take the decision at the start of the relationship/marriage

or later on during the relationship/marriage?

At start of relationship 1 |__|{FAITHSTART}

Later-on during the relationship 2

Q. 186c. How long ago did you decide to be faithful as a way

of preventing HIV infection? |__|__|{DYSFAITH}

|__|__|{WKSFAITH}

|__|__|{MTHFAITH}

|__|__|{YRSFAITH}

Q.186d. What factors led you to being faithful as a way of preventing

HIV infection? |__|__|{FAITHWHY1}

|__|__|{FAITHWHY2}

|__|__|{FAITHWHY3}

Got married 01

Learned my HIV status 02

Learned of my partners HIV status 03

Got couples’ counseling 04

Got a steady partner 05

Lost a relative/friend to HIV 06

Partner did not accept condoms 07

I did not want to use condoms 08

My religious/moral ethics 09

It’s the right thing to do 10

Love/ respect to my partner 11

My family wants me to be faithful 12

Other specify____________________________ 13

Q.186e. Since taking that decision have you been unfaithful to your partner?

Yes 1 |__|{UNFAITH}

No 2

**ARV MODULE**

I WOULD LIKE TO ASK YOU SOME QUESTIONS ABOUT

ANTIRETROVIRAL DRUGS(ARVS).ARVS ARE MEDICINES WHICH HIV

POSITVE PERSONS CAN TAKE TO CONTROL THE VIRUS WHICH

CAUSES AIDS.ARVS STARTED TO COME INTO UGABDA A FEW YEARS

AGO.

Q.187. 1 Have you heard about drugs called antiretroviral (ARV) for HIV

infected persons? |__|{HEARDARV}

Yes 1

No 2------------>**Q.187.5**

Q.187.2 **If Yes:** Where did you hear about these medications? |__|__|{TOLDARV1}

Rakai Program 01

TASO 02 |__|__|{TOLDARV2}

Uganda Cares 03

Hospital 04 |__|__|{TOLDARV3}

Govt health worker 05

Private health worker 06

Radio 07

Newspaper 08

Church 09

Family member 10

Friend 11

Other _____________12 {OTOLDARV}

DR 97 {DRARV}

Q.187.3a. Are ARVs available in Rakai District? |__| {AVLARVRD}

Yes 1

No 2---------------------->**Q.187.4**

DK 7---------------------->**Q.187.4**

Q.187.3b. **If yes:**

Who provides ARVs in Rakai (**code all of respondent’s answers**)

Yes No Rakai Program 1 2 {RPARV}

TASO 1 2 {TASOARV}

Uganda Cares 1 2 {UGCARARV}

Hospital 1 2 {HOSPARV}

Govt doctor/health worker 1 2 {GOVTARV}

Private doctor/health worker 1 2 {PRIVTARV}

Drug shops 1 2 {DSHOPARV}

Traditional healer(s) 1 2 {TRADARV}

Other ___________________1 2 {OTHARV}

{OTHARVmemo}

DK 1 2 {DKARV}

Q.187.4. Do you personally know anyone who takes ARVs in your community? |__|{USEARV}

Yes 1

No 2

Q.187.5. Are you aware of any traditional healer services offering HIV/AIDS

care in your community? |__|{TRADTRT}

Yes 1

No 2

Q.187.6. In your opinion, can traditional healers provide medications that

can cure HIV/AIDS? |__|{TRADHIV

Yes 1

No 2

DK 7

Q.187.7. Have you heard of drugs which can prevent transmission of HIV

from an infected mother to her baby? |__|{HRPMTCT}

Yes 1

No 2

Q.187.8a. From what you have heard, do ARV drugs have benefits for an

HIV-infected person? |__|{HRDBEN}

Yes 1

No 2

DK 3

Q.187.8b. If yes, what benefits? |__|__| {HRDBEN1}

**(UNPROMPTED)** **Code up to three** |__|__| {HRDBEN2}

Cure HIV 01 |__|__| {HRDBEN3}

Make an HIV-infected person feel better/stronger 02

Make an HIV-infected person live longer 03

Help an HIV-infected partner have sex again 04

Help an HIV-infected person be able to work again/

care for family again 05

Reduce the chance of passing HIV to a partner 06

Eliminate the chance of passing HIV to a partner 07

Other _______________ 08 {OTHHRDBENmemo}

DK 97

Q.187.9 Can people taking ARVs transmit HIV if they have sex without using a condom?

Yes 1 |__| {ARVTRANS}

No 2

DK 7

Q.187. 10a From what you know, are there any difficulties in taking ARVs? |__| {ARVDIFF}

Yes 1 -------------> Q 187.11.

No 2 -------------> Q. 187.11

DK/NR 7

Q.187.10b. What are the difficulties in taking the ARV medicine that you

know of? **UNPROMPTED Code up to three**

Cause side effects 01 |__|__| {ARVDIFF1}

Can make people feel sick 02 |__|__|{ARVDIFF2}

Hard to take 03 |__|__| {ARVDIFF3}

Many pills to swallow 04

Hard to remember to take pills 05

Expensive 06

Not always available/hard to get 07

Taste bad 08

Other: ______________________ 10 {OTARVDIFFmemo}

DK 97

**NOW I WOULD LIKE TO ASK YOU ABOUT THE EFFECTS OF THE AVAILABILITY OF FREE ARVS IN RAKAI**

Q.187.11. With the availability of free ARVS is there still

a need to take precautions, such as

abstinence, monogamy or condom use, to prevent getting or

transmitting HIV? |__|{CAUTHIV2}

Yes 1

No 2

DK 7

Q.187.12 With the availability of free ARVS

is it easier for people to make

the decision to learn their HIV results? |__|{VCTAKE2}

Yes 1

No 2

DK 7

**NOW I WOULD LIKE TO ASK FOR YOUR OPINION ABOUT**

**WHETHER THE AVAILABILITY OF FREE ARVS HAS**

**REDUCED, INCREASED OR HAD NO EFFECT ON THE**

**FOLLOWING CHARACTERISTICS AMONG THE PEOPLE IN**

**YOUR COMMUNITY**

Q.187.13Do you think the availability of free ARVs has reduced, increased,

or had no effect on:

Reduce stigma 1 |__| {ARVSTGM2}

Increase stigma 2

No effect 3

Other (specify) ___________________ 4 {OARVSTGM2}

DK 7

Q.187.14 Do you think ARVs have increased, decreased or had no effect on

HIV+ people’s willingness to disclose their HIV status to their

spouse/partner? |__| {DISSPOUSE2}

Increase disclosure 1

Decrease disclosure 2

No effect 3

Other (specify) _________________ 4 {ODISSPOUS2}

DK 7

Q.187.15 Do you think ARVs have increased, decreased or had no effect on HIV+

people’s willingness to disclose their HIV status to their family? |__| {DISFAMLY2}

Increase disclosure 1

Decrease disclosure 2

No effect 3

Other (specify) _________________ 4 {ODISFAMLY2}

DK 7

Q.187.16. Do you think ARVs have increased , decreased or had no effect on HIV+

people’s willingness to disclose their HIV status in the community,

and if so, what effect? |__| {DISCOMMTY2}

Increase disclosure 1

Decrease disclosure 2

No effect 3

Other (specify) _________________ 4 {ODISCOMMY2}

DK 7

Q.187.17 Do you think the availability of ARVs has affected sexual behaviors

in the community? |__| {ARVSEXB2}

Yes 1

No 2

DK 7

Q.187.18a Do you think that the availability of ARVs has increased, decreased,

or had no affect on the number of sexual partners that people have? |__| {ARCSEXPRD2}

More partners 1

Less partners 2

No effect 3

DK 7

Q. 187.18b Do you think the availability of ARVs has made it it more or less |__| {ARVNBRPH2}

likely that people will have casual sex partners? More casual partners 1

Less casual partners 2

No effect 3

DK 7

Q.187.19a Do you think that the availability of ARVs has increased, decreased

or had no effect on the practice of sexual abstinence for HIV

prevention in the community? |__| {ARVABS2}

More abstinence 1

Less abstinence 2

No effect 3

DK 7

Q.187.19b. Do you think that the availability of ARVs has increased, decreased

or had no effect on condom use in the community? |__| {ARVCOND2}

More use 1

Less use 2

No effect 3

DK 7

Q.187.20. Do you think ARVs increase, decrease, or have no effect on the

risk of transmitting HIV infection from an HIV+ person to

their partner? |__| {TRANSARV}

Decreased risk 1

Increased risk 2

No effect 3

DK 7

WE WANT TO ASK YOU ABOUT USE OF LONG-TERM MEDICATION.

THIS INFORMATION WILL BE STRICTLY CONFIDENTIAL. YOU DO

NOT HAVE TO ANSWER THIS QUESTION

Q. 188 Are you or were you on any long-term medications? |__| {LTEMMED}

Yes 1

No 2--------------->**Q. 190**

DK 7--------------->**Q. 190**

NR 9--------------->**Q. 190**

Q.189 **IF YES**, Are/were these ARV medications? |__| {ARVMED}

Yes 1

No 2

DK 7

NR 9

Q.190 During the last 12 months, have you had any of the following

health problems? **PROMPTED:**

Yes No NR

Substantial weight loss 1 2 9 {SYMP02}

Diarrhoea <=1 month 1 2 9 {SYMP03}

Diarrhoea > 1 month 1 2 9 {SYMP04}

Bloody Diarrhoea > 1 month 1 2 9 {SYMP05}

Fever <=1 month 1 2 9 {SYMP06}

Fever >1 month 1 2 9 {SYMP07}

Generalised severe itching 1 2 9 {SYMP08}

Body Rash **(not scabies)** 1 2 9 {SYMP09}

Cough >1 month 1 2 9 {SYMP10}

Kaposi sarcoma 1 2 9 {SYMP11}

Herpes zoster 1 2 9 {SYMP12}

Swollen glands in the neck or arm pits 1 2 9 {SYMP13}

Malaria(B/S) 1 2 9 {SYMP14}

Candida (Oral Thrush) 1 2 9 {SYMP15}

Tuberculosis 1 2 9 {SYMP16}

Scabies 1 2 9 {SYMP17}

Onyongnyong 1 2 9 {SYMP18}

Pnemonia 1 2 9 {SYMP19}

Meningitis 1 2 9 {SYMP20}

Other Cancers 1 2 9 {SYMP21}

Specify __________________ {OTHCANC}

Other problems 1 2 9 {SYMP22}

Specify __________________ {OTHSYMP}

Q.191 Have you been bedridden in the past 30 days?

Yes 1 |__|{BEDRID}

No 2--------->**Q.195**

**If Yes** How long were you in bed?

Less than half a day 1 |__|{BEDTIME}

Half a day to a day 2

More than a day (give # of days) 3

**(Record actual number of days)--------------->**|__|__|{DYSBDRID

**I would like to ask you questions regarding your sexual experience**

**with in the past 12 months. These questions are important for our**

**research, all answers you give me will be kept confidential.**

Q.195. Sexual desire refers that may include wanting to have sexual

intercourse or thinking about having sex. In the past TWELVE

months, how has your level of sexual desire generally been?

**(PROMPTED)**

None at all 1 |__| {SEXDSR}

Low 2

Medium 3

High 4

Dk 7

NR 9

Q196. How likely do you think that you have been exposed to HIV

(virus which causes AIDS)? **(UNPROMPTED)**

Very likely 1 |__|{SAIDSRSK}

Somewhat likely 2

Unlikely 3

Not at all 4

Don't Know 7

**MALE CIRCUMCISION**

**I WOULD LIKE TO ASK YOU A FEW QUESTIONS CONCERNING**

**CIRCUMCISION.**

Q197.Are you circumicised? |__|{CIRCUM}

Yes 1

No 2----------------->**Q.202**

Q198.At what age were you circumicised? |__|{CIRCAGE}

Infant/Child (<12) 1------------>**Q.200**

Youth adolescent (13-20) 2

Adult (21+) 3

DK/NR 7------------>**Q.200**

Q.199 (a) IF CIRCUMCISED AS AN ADOLESCENT OR ADULT ASK:

Were you circumcised after July 2003? |__| {MCIRCAUG}

Yes 1

No 2

Q. 199b Where were you circumcised from? |___| {MCIRCPLA}

Rakai Program 1

Gov’t Health Unit 2

Private practitioner 3

Other (specify)____ 4 {OMCIRCOLmemo}

DK 7

Q200. Why were you circumcised? |__|{WHYCIRC}

Traditional/Religious 1

Health 2

Other(specify)_____________ 3 {OTHWHYCmemo}

DK/NR 7

Q201. Do you have/have you had pain and redness/pain and

swelling on the head of your penis? |__|{PAINSKIN}

Yes 1----------------> PARTNER ID FORM

No 2----------------> PARTNER ID FROM

**FOR CIRCUMCISED MEN GO TO PARTNER IDENTIFICATION FORM**

**FOR UNCIRCUMISED MALES ASK Q.202 THEN FOLLOW SKIP**

**INSTRUCTIONS:**

Q202. Do you have/have you had pain and redness/pain and swelling of the

head of your penis with a white or yellow substance under your penis? |__|{PAINSKIN}

Yes 1

No 2

Q203.Do you retract the foreskin of your penis when bathing? |__|{RETRBATH}

Yes 1

No 2

COMPUTER ID CURRENT ID

-----/-------/-------/-------

Team Leader# |__|__|__|{TEAMLEAD} Editor |__|__|__|{EDITOR} QC|__|__|__|{QC}

Data entry clerk#|__|__|__|{DATA CLERK} {DE DATE dd mm yy}___/____/____

**PARTNER IDENTIFICATION**

**PLACE**

**COMPUTER ID HERE**

**__/__/_200__**

dd mm yy

VISIT # (R12,etc) **|R_|12_|**{VISITNO}

Current ID

SuperCluster # |__|__|__|{REGION}

Community # |__|__|__|{COMM_NUM

HH # |__|__|__|__|{HH_NUM}

Member # |__|__|__|{MEMBER_NUM

**INTERVIEWER: FOR ALL RESPONDENTS IN MARRIAGE OR CONSENSUAL UNION TRY TO OBTAIN INFORMATION THAT WILL ALLOW US TO LINK THE INDIVIDUAL TO HER MARRIAGE OR CONSENSUAL PARTNERS WITHIN THE SAME HOUSEHOLD OR WITHIN THE SAME COMMUNITY. PAY ATTENTION TO CASES OF POLYGAMY WHERE WIVES MAY BE LIVING WITHIN ONE HOUSE OR IN DIFFERENT HOUSEHOLDS. USE ADDITIONAL PAGES IF >3 PARTNERS.**

**FOR SAME HOUSEHOLD PARTNERS, TRY TO FIND THE MEMBER # FROM THE HOUSEHOLD CENSUS LISTING. FOR PARTNERS IN DIFFERENT HOUSEHOLDS, THEIR INFORMATION WILL HAVE TO BE COMPLETED LATER.**

"You stated previously that you are currently married or in a consensual union"

(If Yes),

**Partner 1**

I would like to clearly know, do you have a wife that you are staying with in this household?

**(If Yes,)** Tell me all the possible names by which she is called.

_______________________________________________________________

Current ID # for partner 1: **|__|__|/|__|__|__|/|__|__|__|_|/|__|__|**

(supercluster/community/household/member)

Does she/he normally stay in this community?

Yes .....1 No.....2 Outside spouse ...3

What are her names?

_________________________________________________________________

**SUPERVISOR: TRY TO LOCATE ID FROM HOUSEHOLD CENSUSES IN COMMUNITY**.

Current ID # for partner 1: **|__|__|/|__|__|__|/|__|__|__|_|/|__|__|**

(supercluster/community/household/member)

**(If not)** Can you tell me where she stays?

_________________________________________________________________

_________________________________________________________________

**Partner 2**

**(If Yes,)** Tell me all the possible names by which she is called.

_______________________________________________________________

Current ID # for partner 2: **|__|__|/|__|__|__|/|__|__|__|_|/|__|__|**

(supercluster/community/household/member)

Does she normally stay in this community?

Yes .....1 No.....2 Outside spouse.....3

What are her names?

_________________________________________________________________

**SUPERVISOR: TRY TO LOCATE ID FROM HOUSEHOLD CENSUSES IN COMMUNITY**.

Current ID # for partner 2: **|__|__|/|__|__|__|/|__|__|__|_|/|__|__|**

(supercluster/community/household/member)

**(If not)** Can you tell me where she stays?

________________________________________________________________

**Partner 3**

**(If Yes,)** Tell me all the possible names by which she is called.

_______________________________________________________________

Current ID # for partner 3: **|__|__|/|__|__|__|/|__|__|__|_|/|__|__|**

(supercluster/community/household/member)

Does she normally stay in this community?

Yes .....1 No.....2 Outside Spouse .....3

What are her names?

_________________________________________________________________

**SUPERVISOR: TRY TO LOCATE ID FROM HOUSEHOLD CENSUSES IN COMMUNITY**.

Current ID # for partner 3: **|__|__|/|__|__|__|/|__|__|__|_|/|__|__|**

(supercluster/community/household/member)

**(If not)** Can you tell me where she stays?

_________________________________________________________________

**Partner 4**

**(If Yes,)** Tell me all the possible names by which she is called.

_______________________________________________________________

Current ID # for partner 4: **|__|__|/|__|__|__|/|__|__|__|_|/|__|__|**

(supercluster/community/household/member)

Does she normally stay in this community?

Yes .....1 No.....2 Outside Spouse .....3

What are her names?

_________________________________________________________________

**SUPERVISOR: TRY TO LOCATE ID FROM HOUSEHOLD CENSUSES IN COMMUNITY**.

Current ID # for partner 4: **|__|__|/|__|__|__|/|__|__|__|_|/|__|__|**

(supercluster/community/household/member)

**(If not)** Can you tell me where she stays?

_________________________________________________________________

**APPENDIX 8**

**ORIGINAL PROTOCOL: A Randomized Clinical Trial of Male Circumcision for HIV Prevention - Rakai, Uganda (U01-AI051171-01A2) - Version 3.0 FINAL, April 10, 2007**

#### TABLE OF CONTENTS

**1.0 INTRODUCTION**

1.1 Background

1.2 Rationale

1.3 Study Design

**2.0 STUDY STAGE 2 OBJECTIVES**

2.1 Stage 2 Objectives

2.2 Time Line

**3.0 SELECTION AND ENROLLMENT OF SUBJECTS**

3.1 Population

3.2 Inclusion Criteria

3.3 Exclusion Criteria

3.4 Enrollment/Randomization Procedures

3.4.a Community Mobilization

3.4.b Screening and Enrollment, Informed Consent

3.4.c Procedure at Enrollment

3.4.d Randomization

**4.0 STUDY TREATMENTS AND PROCEDURES AFTER CLOSURE OF ENROLLMENT**

4.1 Surgery

4.1.1 Trial

4.1.2 Procedures after Closure of Enrollment

4.1.2.a Pre-operative history and examination

4.1.2.b Surgical Procedures

4.1.2c Postoperative care

4.1.2.d Asepsis

4.1.2.e Emergency procedures

4.2 Concomitant Medications

4.2.a Analgesia

4.2.b Treatment of Infections

4.2.c Treatment of Anemia

**5.0 CLINICAL AND LABORATORY EVALUATIONS**

5.1 Screening Evaluations

5.1.a Screening Interview and Exam

5.1.b Eligibility

5.1.c Enrollment and Randomization

5.1.d Sociodemographic and Behavioral Interview

5.2 Evaluations Related to Surgery

5.2.a Preoperative Assessment

5.2.b Surgery

5.3 Post-Surgical Evaluations for Intervention Arm, and Post-Enrollment

Evaluations for Control Arm Participants in stage 2

5.3.a Post-operative Evaluations

5.3.b Scheduled Follow up- Both arms

5.3.c Evaluations at Time of Premature Discontinuation

5.4 Laboratory Methods for Evaluation

5.4.1 HIV Testing

5.4.1.a Screening

5.4.1.b Follow up

5.4.1.c Rapid tests

5.4.2 STD Testing

5.4.3 Hemoglobin

5.5 Behavioral Research Methods for Evaluation

**6.0 DATA COLLECTION, SITE MONITORING AND ADVERSE**

**EXPERIENCE REPORTING**

6.1. Records to Be Kept

6.2 Data Management

6.3 Clinical Site Monitoring and Record Availability

6.4 Procedures at each study visit

6.5 Serious Adverse Event Reporting

6.5.a General Definitions

6.5.b Severity of Adverse Events

6.5.c Relationship of AEs to Intervention

6.5.d Study Definitions of Adverse Events

6.5.e Management of Adverse Events

6.5.f Reporting of AEs

6.5.g Tabulations of Adverse Events

6.5.h Source Documentation

**7.0 STATISTICAL CONSIDERATIONS**

7.1 Stage 2 Study Endpoints

7.1.a Primary Endpoint: Male HIV Incidence

7.1.b Male STDs and STD Symptoms

7.1.c Safety of Circumcision

7.1.d Behavioral Disinhibition

7.2 Accrual and Feasibility

7.3 Randomization and Stratification

7.4 Analysis Plan

7.4.a Stage 2 Analysis Plan

7.5 Interim Data Safety and Monitoring

**8.0 HUMAN SUBJECTS**

8.1. Services of relevance to Human Subjects Considerations

8.1.a Community Advisory Board

8.1.b Condom Promotion

8.1.c Voluntary HIV counseling and Testing (VCT)

8.1.d General Medical Care and Health Education

8.2 Institutional Review Boards

8.3 Training in Research Ethics

8.4 Proposed Involvement of Human Subjects

8.5 Recruitment and Consent

8.6 Ethnic Groups and Minors

8.7 Potential Risk to Participants

8.7.a Risks of circumcision

8.7.b Confidentiality and social harm

8.7.c Disinhibition

8.7.d Risk related to biological specimen collection

8.8 Procedures for Protecting Against Risk

8.8.a Risks of circumcision

8.8.b Confidentiality and social harm

8.8.c HIV Voluntary Counseling and Testing (VCT)

8.8.d Disinhibition

8.8.e Risk of biological sample collection

8.9 Study Benefits to participants

8.9.a Health Care

8.9.b STD testing and treatment provided to participants

8.9.c General health care

8.9.d Treatment of opportunistic infections and ARVs

8.9.e Provision of free circumcision

8.9.f Condoms

8.10 Compensation for Participants

8.11 Benefit-Risk Ratio

**9.0 PUBLICATION OF RESEARCH FINDINGS**

**10.0 SUMMARY OF CASE REPORT FORMS, SUMMARY OF INFORMED CONSENT DOCUMENTS** TABLES & FIGURE: RANDOMIZED CLINICAL TRIAL PROTOCOL

**PROTOCOL FOR THE RANDOMIZED TRIAL OF MALE CIRCUMCISION AFTER TRIAL UNBLINDING**

Circumcision in HIV-Negative Men and Risks of HIV/STD Acquisition

Circumcision and HIV risks in HIV-negative men:

Three randomized trials have shown that circumcision reduces male HIV incidence by 50-60%. Many, but not all cross-sectional epidemiologic studies, particularly from Africa, show lower HIV prevalence rates among circumcised compared with uncircumcised heterosexual men.^1-8, 23-27^ In addition, ecologic analyses suggest that the distribution of prevalent HIV is inversely correlated with the prevalence of male circumcision.^28-29b^ A meta-analysis which included 18 cross-sectional or case-control studies of prevalent HIV and 3 cohort studies (two from the Rakai Health Sciences Program), estimated an adjusted relative risk of HIV infection in circumcised relative to uncircumcised men of 0.42 (CI 0.34-0.52).^3^ The reduction in risk was even more pronounced in men with high HIV exposure (RR=0.29, CI 0.20-0.41).^3^ In Rakai, HIV risks were lower in circumcised men both for incident infection (RR=0.53, CI0.3-0.9)^1^ and prevalent HIV (OR=0.41, CI 0.3-0.5).^2^ The protective effects were most marked in highly exposed men in HIV-discordant couples: within this subgroup, there were no seroconversion in circumcised HIV-negative men, compared with an incidence of 16.7 per 100py in uncircumcised men (p = 0.0004).^1,30^ A study of high risk trucking company employees in Kenya also found reduced HIV acquisition in circumcised men (RR= 0.25, CI 0.1-0.53).^5,31^ In Kenyan men with GUD, HIV incidence was lower among those who were circumcised (RR=0.41, CI 0.23-0.93).^5a^ Circumcision is also associated with a lower risk of HIV in homosexual men.^32^

In Rakai, the protective effects of circumcision are most pronounced among men in whom the procedure was performed before puberty. Compared with uncircumcised men, pre-pubertal circumcision ( 12 years) was associated with significantly reduced risks of prevalent HIV (OR = 0.39, CI 0.29-0.53), as was circumcision performed between the ages 13-20 years (OR = 0.46, CI 0.28-0.77). However, risks of prevalent infection were not significantly reduced with circumcision after age 20 (OR= 0.78 (CI 0.42-1.43).^2^ Circumcision before puberty was also associated with a significantly lower rate ratio of incident HIV (RR = 0.49, CI 0.3-0.8), compared with uncircumcised men, whereas circumcisions after age 12 years was not significantly protective (RR = 0.70, CI 0.3-1.6).^1^ The apparent attenuation of protective effects with older age of circumcision probably reflects confounding by medical indication. In Rakai, 75% of post-pubertal circumcisions were performed for health indications (e.g., phimosis), and it is likely that such pathology is, in part, a consequence of prior STD infections, which are markers for high risk behaviors.^1^ However, it is also possible that scar formation following adult circumcision may result in more friable tissue and less keratinization than with pre-pubertal circumcision, and a friable scar might increase vulnerability to HIV infection among men circumcised in adulthood, particularly if intercourse is resumed before complete wound healing. It is also possible that preexisting infections, which are indications for adult circumcision, may retard healing. We do not know of other data on HIV risk in relation to age of circumcision or indications for the procedure, nor are we aware of information on scar formation or whether healing is delayed by preexisting infections.^9^ Thus, although adolescent or adult circumcision is likely to be protective against HIV, observational data cannot quantify the magnitude of this effect, due to probable confounding by indication and factors affecting postoperative tissue repair. Data are urgently needed to determine and optimize safety of post-pubertal circumcision, and to assess whether preceding pathology may affect HIV acquisition or wound healing.

Circumcision and risks of STDs and STD symptoms in HIV-negative men

Circumcision is associated with reduced male risks of viral and bacterial STDs,^24,25,33-38^ genital ulceration,^2,924,37,39^ urinary tract infections^41-43^ and balanitis.^45,46^ In Rakai, GUD is significantly less frequent in circumcised compared with uncircumcised men (RR=0.70, CI 0.54-0.93),^2^ and symptoms of penile inflammation or infection consistent with balanitis are also lower among circumcised men (RR= 0.51, CI0.33-0.77 , unpublished). Circumcision is also associated with reduced risk of penile cancer due to HPV.^44,47^ Thus, the benefits of circumcision may extend to a wide range of STDs and other genital tract infections. This is important because the circumcision effect on HIV may be mediated by protection from STDs.

**1.2 Rationale**

Observational data and three randomized trials show that male circumcision reduces the rate of men acquiring HIV infection by approximately 50-60%. Thus circumcision is an important public health measure because, despite initial costs, it could provide long-term risk reduction in males and secondary protection against infection in their female partners, without incurring recurrent costs.

1.3 Study Design

We have conducted an individually randomized, unblinded, two armed trial to assess the efficacy of circumcision in HIV-negative men for prevention of HIV and STD acquisition. The study was conducted in rural communities of Rakai District, Ugandan and enrolled 5,000 HIV-negative men who accepted voluntary counseling and testing (VCT). The trial was conducted in two stages which represent a continuum. In the first stage, 200 men were enrolled into a study of the acceptability, feasibility and safety of circumcision, with frequent postoperative follow up to determine rates of wound healing and complications. After assessment of the first 200 men enrolled in stage 1, and in consultation with NIAID Program and the DSMB, we embarked on the main trial (Stage 2) to determine the efficacy of circumcision for HIV prevention. In both stages, we individually randomize consenting HIV-negative men to an early circumcision Intervention Arm or delayed circumcision Control Arm. Follow up is for 24 months. We promised to offer circumcision to the Control Arm participants after completion of two years follow up, if there was evidence of safety at that time. However, we informed control arm participants that the efficacy of circumcision for HIV prevention was not be known until the 5^th^ project year, and that they may elect to wait until completion of the trial before deciding on circumcision.

A complementary but separate Gates Foundation supported trial enrolled HIV+ men and men who decline VCT was conducted concurrently in the same communities as the NIH funded trial.

**2.0 STAGE 2 STUDY OBJECTIVES**

The NIH funded trial enrolled 5,000 HIV- men.

**2.2 Stage 2 Objectives:**

Stage 2 followed Stage 1 and was a two armed individually randomized trial of circumcision versus a control condition, in HIV-negative men (n = 2,400 men enrolled per arm, additional to those enrolled in Stage 1, for a total of 2,500 men per arm). The objectives are:

Primary Objective:

1. To determine whether HIV incidence will be significantly different among HIV-negative male participants in the Circumcision Intervention Arm, compared to the Control Arm.

Secondary Objectives:

To determine:

1. Whether circumcision is acceptable to men in Rakai.

2. If circumcision is safe among HIV-negative men, with and without preexisting penile pathology (e.g., STDs, balanitis, phimosis).

3. Whether STD symptoms, prevalence and incidence are reduced among male participants in the circumcision intervention arm, compared with the control arm.

4. To assess whether men in the circumcision arm adopt higher risk behaviors compared with men in the control arm (i.e., whether the intervention engenders behavioral disinhibition).

2.3 Time Line

The time line is given in Table 1.

3.0 SELECTION AND ENROLLMENT OF SUBJECTS

3.1 Population

Stage 1 enrolled 200 HIV-negative men, with 100 randomized to immediate circumcision and 100 to delayed circumcision (control). Men were enrolled from two populations:

1. Men currently, enrolled in the ongoing Rakai Community Cohort study (RCCS).

2. Men who are not cohort participants but reside in communities accessible to the Kalisizo field station were also invited to participate and enrolled in the Kalisizo Field Station or satellite facilities (called “hubs”). These men were termed “walk in” participants.

3.2 Inclusion Criteria:

Male eligibility criteria for the NIH trial were:

- HIV-negative uncircumcised men who accepted to receive their HIV results
- aged 15 - 49 at screening
- who accepted circumcision and agree to randomization of timing of circumcision,
- were capable of and provided full informed consent,
- had no anatomical abnormality of the penis (e.g., hypospadias, severe phimosis) which would preclude safe circumcision, or which constituted a medical indication for therapeutic circumcision
- had no general medical condition that was a contraindication to surgery or use of local anesthesia
- hemoglobin  8 grams/dL
- intended to stay in Rakai District for at least one year and/or available for follow up

3.3 Exclusion Criteria

Men were excluded from the NIH trial if they were:

- HIV- positive
- refused receipt of HIV results
- aged <15 or > 49 at screening
- were circumcised or partially circumcised
- refused to accept randomization
- refusedconsent
- had anatomical abnormalities which were contraindications for circumcision or medical

conditions which required therapeutic circumcision

- had medical conditions which were contraindications for surgery or use of local anesthesia
- hemoglobin < 8 grams/dL which was not rectified by treatment ( section 4.2.c)
- did not intend to remain within Rakai for at least one year

3.4 Enrolment and Randomization Procedures

Stage 2 enrollment and follow up are summarized in figures 1 and 2 ( page 45-46).

**3.4.a Community Mobilization**

All target communities were informed of the study and men were invited to participate. The community mobilization messages emphasized:

- That the Rakai Health Sciences Program was conducting a study to assess the acceptability and safety of circumcision, in collaboration with the NIH.
- That there was preliminary evidence from Rakai and other studies that circumcision may reduce the risk of male HIV and STD acquisition, but this had not been proven. Therefore, a randomized trial was needed to assess whether circumcision is truly protective against HIV and STDs.
- That circumcision would be offered free of charge in fully equipped surgical theaters, and would be conducted by trained physicians under local anesthesia, in conditions of careful asepsis. Pain management and careful follow up would be provided.
- Men eligible for the study were HIV- negative, aged 15-49, willing to accept randomization, and willing to be followed up in the home/hub for at least two years post enrollment.

**3.4.b. Screening and Enrollment, Informed Consent**

- Screening and enrollment was conducted by Circumcision Enrollment Teams. Medical Officers supervised enrollment in the field and clinics. Enrollment teams generally consisted of a team supervisor, 4-5 research assistants, a data editor and a laboratory technician.
- Cohort participants were enrolled from ongoing cohort surveys. The non-cohort participants were enrolled at the Kalisizo Field Station or “hubs”.
- All men were offered VCT. Pre-result (i.e. pre-test) counseling was done in groups or individually and also included a video. Receipt of results and post-test counseling was done individually and in private.
- All men were interviewed to ascertain genital symptomatology (GUD, discharge, urethritis/dysuria, balanitis, phimosis and other relevant general medical conditions (Form 01 )..
- Men were examined to confirm that they were uncircumcised, and to detect pathology (infections, phimosis, penile abnormalities and GUD), and samples collected from under the foreskin (PCR and HPV swabs, to be assessed with separate funding.)
- Blood, swabs and urine samples were obtained for HIV and STD testing including serologic syphilis.

HIV and syphilis testing was conducted in real time, and results provided if the participant agreed to receipt of results. Men with serologic syphilis received treatment. Men with serologic syphilis who did not have genital lesions were eligible for enrollment (i.e., latent syphilis). Men with serologic syphilis and concurrent GUD (i.e., primary or secondary syphilis), were treated and enrolled if and when the genital lesions were resolved.

- Treatment was provided for infections and referral provided for penile abnormalities or intractable infections prior to enrollment. STD treatment was made available for partners.
- Finger stick blood was collected for hemoglobin measurement using a portable hemoglobinometers. Men with Hgb < 8.0g/dL were treated for anemia, but not enrolled in the NIH trial unless and until their Hgb  8.0g/dL
- Informed consent for screening was obtained. Men eligible for the study completed another consent document prior to enrollment and randomization (Consent Forms I and Ia).
- After randomization men were informed of their allocation and provided with a post-randomization information sheet describing immediate or delayed circumcision procedures (Form II).
- Men who were initially found ineligible due to treatable genital conditions, anaemia, or men reporting more than 30 days since last screening were re-assessed for eligibility using the eligibility form.
- Men were provided with a circumcision study ID# , as well as a Rakai alphanumeric study ID#. These were preprinted labels attached to questionnaires and samples. In addition, a photo ID allowed tracking and correct identification of subjects.
- Those men randomized to immediate circumcision were scheduled for surgery and given tetanus toxoid immunization. Arrangements were made for transport if needed.
- Men randomized to the control arm were informed that they would be re-contacted for follow up and were offered free circumcision, contingent on Program and DSMB approval, after 24 months
- Eligibility for enrollment into the NIH trial was recorded on the NIH Eligibility verification Form 22.

**3.4.c. Procedures at Enrollment**

- Cohort participants who had completed the baseline survey less than six months prior to enrollment, did not require a baseline re-interview.
- Cohort participants recruited from the ongoing RCCS cohort study who had completed a follow up interview on sociodemographics, risk behaviors and health more than six months before enrollment were administered the baseline survey (Form 02a).
- Non-cohort participants were asked to provide a baseline interview and samples using the current cohort questionnaires (Form 02a).
- All men received soap, and men scheduled for surgery were asked to wash their penis daily until surgery

**3.4.d. Randomization**

- Johns Hopkins University generated a list of randomized numbers in blocks of 20 to insure comparability of men randomized to intervention and control arms, within cohort communities or groups of “walk in” participants”.
- A random assignment sheet was placed in an opaque sealed envelope. The envelopes in blocks of 20, were generated by Rakai health sciences Program Data management using a computerized list provided by JHU, and was retained securely by the Circumcision Data Management Team.
- After enrollment and consent, men were asked to select an envelope from the block of 20 envelopes being used at that time. The envelope contained the assignment sheet showing the randomization number and random assignment to treatment or control arm. The Enrollment Team then affixed the label with the individual’s study ID number to the sheet. (Form 18).
- The assignment sheet with the study ID# labeled was entered into a data base to record study arm allocation.
- The list of random allocation numbers linked to participants was retained at JHU in locked files, and in password protected computers.
- All randomization envelopes were logged out and in daily. A data base monitored used and unused allocation numbers, and track disposition of all envelopes.
- Men were randomized at the enrollment visit. If participants change their minds and withdraw, or at a subsequent preoperative visit, the surgeon determines that circumcision is contraindicated for medical reasons, the men would still be considered enrolled and randomized.
- After randomization, men completed a consent comprehension test to ensure that they understood the trial and to correct any misunderstandings.

**4.0 STUDY TREATMENTS** AND PROCEDURES AFTER CLOSURE OF ENROLLMENT

4.1. Surgery

4.1.1 Trial

On the scheduled day of surgery transport was provided if needed. Men who previously consented but changed their minds and were contactable were interviewed to ascertain why they declined surgery. Repeat visits to contact absentees were conducted for a period of approximately one month, and if not contacted, their location and reason for absence were obtained from household members. Men who reported for surgery more than 30 days since last hemoglobin test had a repeat hemoglobin test.

Men who changed their minds: a) if at 6 months a man randomized to circumcision decides not to have surgery, he was classified as a cross-over and followed over two years**. b)** For men who receive delayed surgery between 1-6 months, the subsequent 6, 12 and 24 months visits were scheduled from time of surgery rather than from time of enrollment. For men with surgery delayed for less than 1 month (<1month), subsequent 6, 12, and 24 month follow up occured from time of enrollment.

Men who had a medical contraindication resulting in delayed surgery could come for surgery within 6 months post enrollment. If a man had a contraindication that did not resolved by 6 months, he was **classified as not eligible and withdrawn from the study.**

Men were re-consented for surgery (using the revised Surgical Consent Form III), and if they agreed to surgery they were briefly interviewed and examined by a clinical assistant, and by a physician who would certify fitness for surgery (Preoperative Interview/Exam 03).

Surgery was performed per protocol as indicated below:

**4.1.2 PROCEDURES AFTER CLOSURE OF ENROLLMENT**

All control participants will be informed of the trial findings and will be offered surgery. Control participants will be contacted in the chronological order of their enrollment, so that controls who enrolled earlier and completed 24 months follow up will be given initial priority, and after completion of surgery for these men, priority will then be accorded to men by date of enrollment. Controls who present at the the Kalisizo facility for unscheduled visits and who request surgery will also be provided with circumcision at time of presentation. Incident cases in the control arm who have completed 24 months follow up will be offered surgery. In addition, men who have seroconverted during the trial will be offered the opportunity to enroll in the Gates funded protocol for HIV-infected men. The Gates protocol involves an increased number follow-up visits for a more detailed documentation of healing. Men initially randomized to the intervention arm who failed to return for surgery within six months of enrollment and were classified as crossovers, will also be offered surgery.

All participants and the community at large will be informed of the trial results and of the availability of surgery for trial participants who remain uncircumcised.

4.1.a Pre-operative history and examination

- All participants who have not received tetanus toxoid will be offered immunization.
- The genitalia will be inspected

The following additional samples will be collected: urine and swabs from under the foreskin for future testing, such as gonorrhea/chlamydia/HSV-2 and HPV, with separate funding. During surgery a filter paper blood spot will be collected for archival purposes.

- If there is clinical evidence of current infection (e.g., discharge, GUD), that might increase the risk of surgery, circumcision will be deferred and the patient will be treated and given penile hygiene instructions.
- If there are anatomic abnormalities (e.g., hypospadias, severe phimosis, urinary retention), the patient will be referred for evaluation to the consultant urologist, who will visit the clinic approximately at 2-4 week intervals to review these cases.
- For men who live a long distance from the Kalisizo clinic, a pre-operative overnight stay will be made available, if they choose to use this service.

**4.1.b Surgical Procedure:**

Sleeve circumcision will be performed as follows:

- The penis and both surfaces of the foreskin are preped.
- Local anaesthesia (a mixture of Lignocaine and Marcaine) will be used to block the dorsal penile nerves at the base of the penis and to infiltrate the frenulum.
- With the foreskin in place and the skin lying undistorted on the shaft, a line is drawn following the outlines of the corona and the V of the frenulum. The foreskin is then retracted and a line marked 0.5 - 1 centimeter proximal to the corona, straight across the base of the frenulum.
- With the foreskin retracted the first incision follows the line marked on the inner surface, and bleeding is controlled by bipolar cautery. The incision is deepened to mobilize the skin edge. The prepuce is then extended over the glans and the marked proximal incision is made on the external surface.
- The distal skin edge of the foreskin will be retracted using skin hooks and/or artery or Allis clips, or sutures to permit dissection to free the dartos fascia from the skin.
- The sleeve of prepuce is then removed and will be retained for pathophysiologic studies.
- The foreskin will be laid out flat and measurements taken of the margins for estimates of surface area. The foreskin will then be cut into four sections placed in safe-fix ^TM^ or other appropriate fixative for archiving in Uganda and the US for histopathology.
- All bleeders are controlled,
- Skin edges re-approximated with 4-O or 5-O absorbable sutures. The first stitch is a simple suture at the apex of the frenulum followed by a horizontal mattress suture at the base of the frenulum, and then three vertical mattress sutures of each of the remaining three points of the quadrants, and a minimum of two simple sutures between each principle mattress suture (a total of 13 or more stitches) .
- The wound is cleaned with normal saline
- Medicated, non-adhesive gauze is applied and knotted on the four principle sutures, another free medicated, non-adhesive gauze is wrapped around the first, and a regular gauze dressing applied, followed by an elastic bandage, held in place with strapping
- The procedure usually takes 45-60 minutes.
- The patients will recuperate for approximately 2-4 hours in a post-operative recovery room or until they are ready to go home.
- The surgical procedure and postoperative recovery will be recorded on Form 04 .
- . Men will be given well fitting underwear for comfort and to prevent wound disruption, if needed.

Other methods of circumcision such as forceps or dorsal slit may be used

**4.1.c Postoperative care**

- Analgesics will be provided (see 4.2)
- Men will not be discharged if they experience severe pain, cannot walk independently or have evidence of bleeding through the dressing
- Men will be instructed not to share their pain medications. The postoperative visit will be used to monitor analgesia use.
- Men will rest for a minimum of 30 minutes or longer until they feel able to return home All men will be examined by a physician or health worker prior to discharge and a Form 04 completed.
- Men will be provided with instructions on wound care and analgesia, and this will be reviewed with them before discharge (Postoperative patient Information Sheet 46).
- At time of discharge, men will be offered free transport. Men who decline or do not require transport (residence close to the Rakai Health Sciences Program clinic) will be advised to be accompanied by a family member, friend or Rakai Health Sciences Program staff.

• Free postoperative overnight stay facilities will be made available, if men choose to avail themselves of this service.

**4.1.d Asepsis**

- All theater staff will be trained in aseptic procedures, including documentation.
- Steam Indicators, Biological Indicators, autoclave tracking labels and instrument lists will be used to insure sterility, tracing of instruments and QC.

**4.1.e Emergency procedures:**

- A crash cart, IV and oxygen will be available for emergencies.
- The main potential emergency is excessive bleeding, which will be controlled by ligation of vessels and by pressure. If uncontrolled bleeding persists, a pressure dressing will be applied, and a catheter wrapped around the penile shaft as a tourniquet. If these measures do not control bleeding, an IV will be inserted and oxygen provided if needed, and the patient will be transported to hospital (Kalisizo Hospital < 5 minutes away; larger hospitals in Masaka (Masaka Hospital or Kitovu, a mission hospital ~30 minutes) away, if needed.
- Anaphylactic reaction to the local anaesthetic will be managed with epinephrine, oxygen, IV and transport to the Hospital, if needed.

4.2 Concomitant Medications

**4.2.a. Analgesia**

- Experience has shown that postoperative Tramadol is not needed for pain control due to the long action of the local anaesthesia. Also, Tramadol can delay discharge due to drowsiness Therefore postoperative and subsequent analgesia will use Acetominophen 325 to 650 mg, every 4-6 hours, as needed.

Postoperative analgesia with Tramadol hydrochloride 50-100 mg stat will be provided if Acetominophen does not control postoperative pain.

Men will be evaluated within 48 hours, and if moderate to severe pain is reported, Tramadol hydrochloride 50 - 100mg- will be dispensed.

**4.2.b. Treatment of Infections**

1. Treatment of STDs and Balanitis.

- we will use symptom-based management of symptomatic infections at time of survey. In addition, syphilis treatment will be provided on the basis of serology.
- The regimen of treatment will, where possible, use directly observed, single oral therapies developed and evaluated in the STD Control Trial. The treatment algorithms include the following:

1) *GUD*. Therapy will cover *T. pallidum, H. ducreyi* and HSV-2: azithromycin 1 gram, ciprofloxacine 500 mg stat, IM benzathine benzylpenicillin 2.4 million units if there is serologic evidence of syphilis, and acycolvir 400mg t.d.s for five days for active herpes (per PDR 2003) ,other therapy may be provided as medically indicated

2) Urethral discharge/dysuria in men. Therapy will cover *N. gonorrohoeae*, and *C. trachomatis*: azithromycin 1 gram, ciprofloxacine 500 mg.

3) *Balanitis* treatment will consist of metrondizaole 2 grams stat for anaerobes, and topical clotrimazole for *C. albicans*.

*2. Pre- or post-operative penile infections* (other than balanitis) will be treated with azithromycin and ciprofloxacin. Indications for treatment are: a) clinical evidence of infection among men presenting for circumcision or after circumcision, and b) symptoms suggestive of current STDs (GUD, discharge, dysuria), reported by men during surveys or at post-circumcision visits, in both arms. Uganda MoH standard of care will be used for partner notification (MoH-STD Form 4) – Form 35 and provision of free treatment.

**4.2.c. Treatment of Anemia**

Men with hemoglobin < 8 grams/dL, will receive iron/folate supplements and presumptive treatment for malaria and helminths using standard MoH regimens for treatment of anemia in adults. Enrollment and randomization will be deferred until the hemoglobin is increased to  8 grams/dL. If the patient does not respond to therapy, they will be referred for investigation of persistent anemia. Anemia resulting from hemoglobinopathies such as sickle cell disease will be referred.

5.0 CLINICAL AND LABORATORY EVALUATIONS

5.1 Screening Evaluations

**5.1.a. Screening Interview and Exam**

- Consent for screening was obtained (Form I)
- An interview ascertained symptoms of penile pathology and other relevant medical conditions. (Form 01)
- All participants were asked to provide blood for HIV testing and hemoglobin determination (See Lab Methods 5.5.1 and 5.5.3) to assess eligibility prior to enrollment Blood and urine was collected and stored for future STD testing (HSV-2, gonorrhea and chlamydia PCR) with other funds (See Lab Methods 5.5.2 ).
- An exam was performed to verify circumcision status, and to diagnose infection, balanitis, phimosis and anatomical abnormalities (Form 01 ).
- At time of exam, swabs were collected for future assays such as Hybrid Capture and/or PCR for HPV, and for Chlamydia and gonorrhea, and M-PCR for GUD (under separate funds).

**5.1.b. Eligibility**

- Eligibility Screening consisted of willingness to participate, consent to circumcision and to randomization of timing of circumcision, confirmation of uncircumcised status, confirmation of HIV- negative status and acceptance of VCT, and confirmation of Hgb 8.0 g/dL. Thus, eligibility could only be determined after interview, exam and HIV/Hgb test which were recorded in the Eligibility Verification Form 22 .

**5.1.c. Enrollment and Randomization**

- After confirmation of eligibility men were consented for enrollment (Form I.a.), randomized and given a Post-Randomization Information Sheet (Form II) to inform them of their study arm allocation. Random allocation was recorded on Form 18.

5.1.d. Sociodemographic and Behavioral Interview

- Cohort participants routinely complete a sociodemographic, behavioral and health follow up interview during annual cohort visits (Form 48.). For cohort participants who completed this interview within six months of enrollment, no re-interview was needed. Cohort participants interviewed more than 6 months prior to enrollment were re-interviewed to up date behavioral information.
- Non-cohort participants were interviewed using the RCCS baseline interview Form 02a

5.2. Evaluations Related to Surgery

**5.2.a. Preoperative Assessment**

- Prior to circumcision, men were asked to consent to surgery (Form III), assessed by a clinical health worker or a physician. The clinical health worker took a general medical history, conducted a general systems exam and a genital exam (Form 03 ). The assessment included general medical history and general systems exam and a genital exam (Form 03 ). The clinical health worker or physician examined the penis and ensured that the patient is fit for surgery. Any infection or other abnormality which might be a contraindication for surgery were treated per protocol or by referral, and surgery was rescheduled.
- Swabs were taken for future assays such as PCR, HPV and culture

**5.2.b Surgery**

- The foreskin with the frenulum facing upwards, is placed on a board. The width and length is measured in centimeters and recorded.
- Four separate quadrants with full thickness sections of the foreskin is placed in safe-fix or other appropriate fixative and labeled with the ID#.
- A Surgical Record form (Form 04 ), records the surgery and post-operative status prior to discharge.

5.3 Post-Surgical Evaluations for Intervention Arm, and Post-Enrollment Evaluations for Control Arm Participants in stage 2

**5.3.a. Post-operative Evaluations**

The follow up schedule for control surgeries is as follows:

The schedule of postoperative evaluations for stage 2 of the trial is given in Figures 2

1^st^ post operative visit <48 hrs of surgery.

2^nd^ post operative visit at 7± 2 days following surgery.

3^rd^ post operative visit 3-6 weeks following surgery

Unscheduled visits can occur at any time if a participant experiences problems. Participants can contact the project via community counselors who are equipped with cell phones. If wound healing is incomplete at visit 3, men will be followed weekly until wound healing is certified.

These visits, which can take place in the participant’s home or at a convenient central location (“hub”), will record AEs, symptoms, resumption of intercourse, assessment of condom use, examination of the wound and collection of a culture swab if there is evidence of infection, evaluation of wound healing and certification if healing is complete. Blood, urine and penile swabs will not be routinely collected at these postoperative visits, unless needed for patient care.

**5.3.b Scheduled follow up - Both arms**

All men were seen 4-8 weeks post-Enrollment. For the intervention arm, this coincided with postoperative visit 3 to certify healing.

All participants are followed up at 6, 12 and 24 months to ascertain study end points, including HIV status (Form 12). At each 6, 12 and 24 month follow up contact, blood is collected for real time HIV and syphilis serology, and urine and penile swab samples are obtained for future STD testing (see 5.5.3)..

**5.3.c Evaluations at the time of premature discontinuation**

If participants withdraw from the study, this will be recorded on a withdrawal form (Form 14).

5.4. Laboratory Methods for Evaluations

**5.4.1.HIV Testing**

**5.4.1.a. Screening**

At screening, all samples were assessed by two separate EIAs, and if both EIAs were negative, the participant was informed that he was HIV-uninfected and enrolled. . If both EIAs were positive the participant was informed that he was HIV infected. Men who failed to return for enrollment were followed up and attempted contacts were documented.

If the EIAs were discordant, HIV status was determined by western blot (WB), and only confirmed WB negative or positive participants were provided with results and

VCT. Indeterminate WB samples were subject to qualitative or quantitative PCR on plasma or serum prior to provision of HIV results. EIA/Western blot could be run in the field laboratory in Kalisizo, and thus results could be provided in a timely manner. Only participants with confirmed negative results were enrolled.

**5.4.1.b Follow up**

At follow up (6, 12 and 24 months) we use the two EIA/WB algorithm as described above. In addition, all seroconverters detected by two EIAs, are confirmed by WB. Indeterminate WBs are subjected to qualitative or quantitative PCR on plasma or serum prior to provision of HIV results. Seroconversions during the first 0-6 month follow up are evaluated, and if need be, the baseline and/or 4 week blood samples are tested by PCR to assess whether participants may have been in the window period at time of enrollment, or are early seroconverters by the 4 weeks follow up. However, the 4 weeks blood sample was not tested in real-time.

**5.4.1.c Rapid tests**

Rapid tests were not routinely used for screening. However, if shortages of EIA kits became a problem, the following procedure was to be used: Two separate and sensitive

rapid tests (such as Determine and Stat-Pak) were to be used for screening. We have shown that the negative predictive value is ~99.8%. Both rapid tests must have been negative for

persons to receive their HIV results and be enrolled. If either rapid test was positive (with a weak or strong band), or equivocal, the sample would be subjected to EIA/WB and no

results would be provided to the participant until the latter tests were completed, as described above. All rapid tests would be confirmed by two EIAs and WB as soon as these

became available.

Tests would be run in batches and technicians blinded. If a participant refused to provide blood during follow up, we requested a urine sample for HIV testing using a urinary EIA and Western blot

**5.4.2 STD Testing.** Serology is performed for syphilis and RPR-positive samples are confirmed by treponemal-specific tests such as TPHA, TPPA, or FTA-ABS.

Using other funding and separate protocols, stored samples will be tested in future for infections such as herpes simplex virus type-2 (HSV-2) infection using Focus EIA. *Neisseria gonorroheae* and *Chlamydia trachomatis* will be detected by PCR on male urine samples Since GUD is a cofactor for HIV acquisition, all ulcers are swabbed for future testing by PCR for syphilis, *H. ducreyi* and HSV-2 in U.S.A or in Uganda once assays are established in our laboratory. (At this time urinary PCR for chlamydia and gonorrhea are available and HIV PCR viral load is being validated). .

5.4.3. Hemoglobin

Hemoglobin was measured at screening in order to determine eligibility for enrollment (see eligibility 3.2). Hemoglobin is determined using portable hemoglobinometers (e.g., HemoCue). A finger stick fresh blood sample is collected in a cuvette inserted into the machine and read within 10 minutes. The machines are calibrated each morning using standard controls.

5.5. Behavioral Research Methods for Evaluations.

Behavioral studies have already been conducted on circumcision acceptability and perceptions of risk and benefit with other funds. Behavioral research was conducted concurrently with the trial to assess participant and community beliefs and attitudes towards circumcision, to evaluate the experience of men receiving surgery and the attitudes of control subjects allocated to delayed circumcision. These studies provided qualitative information on participant and community beliefs about possible protective effects of circumcision which might affect behaviors, including disinhibition. Separate informed consents were developed for these behavioral study participants. Subjects included male trial participants and non-trial participants, and females (irrespective of whether their partners are trial participants or not). The methods include:

*Structured Interviews* were conducted with male trial participants at enrollment and in follow up surveys to ascertain risk behaviors (number and types of partners, coital frequency, condom use, alcohol use etc.), self-perceived HIV risk and use of VCT.

*Rapid Ethnographic Assessment (REA).* For exploration of norms, values and meanings regarding circumcision, we conducted in-depth interviews and focus group discussions with men and women aged 15-49 . This information will assist in the development of education messages on the benefits and risks of circumcision, and will facilitate interpretation of information obtained by the structured interviews. Application of multiple data collection methods permits “triangulation”, and validation of findings. The domains to be specifically explored include perceptions, facilitators and barriers to circumcision, the relationship of circumcision to sexuality, religion, and risk for HIV/STDs. Behavioral data was collected throughout the study to adjust our education and community motivation programs as needed.

*In-depth interviews* were conducted with male and female key informants from different age groups (15-19, 20-29, 30-39, 40-49) to provide insights into the experiences, attitudes, perceptions and opinions of individuals. A total of approximately 40 informants (~5 male and ~5 female from each of the age groups), were identified through purposive sampling, based on informant’s enculturation and involvement in their respective communities. The sample included representation by residence, ethnicity, religion, economic activities, and education. Open-ended questions werbe used to encourage conversation and enable probing, to elicit information on experiences and on disinhibition. Although females were not enrolled in the trial, we believe it was crucial to enroll them in the REA, since their attitudes and support will be critical for male circumcision acceptance.

*Focus group discussions*. We conducted approximately 32 focus groups each stratified by marital status and gender in age groups 15-19, 20-29, 30-39 and 40-49 to elicit information on shared beliefs, opinions, group concerns and expectations, group knowledge and awareness, and other normative perspectives. The primary objective was to elicit normative perceptions of circumcision, terminology used locally in discussing circumcision, and opinions regarding its acceptability as an HIV/STD preventive measure. Focus group discussions with community gatekeepers, stakeholders as well as community members were held.

*Qualitative data management and analyses:* The ethnographic data was transcribed and translated by experienced Rakai Health Sciences Program personnel. The word processed text was then coded and organized using NVIVO. Further editing was done in Uganda and the US. Data analysis/summaries will be completed in the US and will focus on barriers to circumcision, means for overcoming barriers, incentives that might be acceptable, and perceptions or beliefs which might lead to behavioral disinhibition.

**6.0	DATA COLLECTION, SITE MONITORING AND ADVERSE EXPERIENCE REPORTING**

6.1	Records to Be Kept

The records kept for each subject are consents and record forms. These are summarized in Tables 2. Files are maintained on each participant to provide a chronological record of all forms used. Files are organized by study number and maintained in locked secure storage rooms.

6.2	Data Management

- Data management is conducted by the Rakai Health Sciences Program and JHU. We have extensive experience managing large data sets. We use existing Rakai Health Sciences Program census and survey forms, with supplementary record forms specific to the trial. Forms were translated into Luganda and printed in Uganda.
- All participants have a household census enumeration. Census files are used to generate updated data to provide computer generated tracking forms, and pre-printed labels for all questionnaires and samples.
- Data collection is conducted by highly trained and experienced full-time, same-sex interviewers. Study editors check all completed questionnaires and query inconsistencies or errors in the field or hub so that interviewers can return to re-interview subjects and correct errors immediately.
- Subjects are identified by photo IDs and have permanent, unique alphanumeric check digit study ID numbers which prevent keying errors. Subjects enrolled from the cohort also have location numbers for community, household and census resident numbers to facilitate tracing.
- Data entry is done in Rakai Offices using Foxpro screens with programmed range and consistency checks. Lab data and questionnaire are double entered. The data entry system is adapted for this trial by experienced data managers in Uganda and JHU, and relational data bases will be constructed.
- The data is sent by FTP or zip files to JHU. Further data editing and cleaning are conducted at JHU and queries sent to Uganda for clarification and error correction.
- Security is maintained by storing all data files on codeword protected machines accessible only to senior data managers and analysts.
- Standard operating procedures are written and updated.
- Adherence to protocol is assessed by periodic checks on consent forms and hard copystudy instruments, as well as data sets.

6.3.	Clinical Site Monitoring and Record Availability

Monitoring is conducted by Westat or other monitors at intervals to be determined by NIAID.

All study hard copy records will be available to site monitors and will be maintained in individual chronological files on all study participants. Computer files may be accessed on site during a monitoring visit.

6.4.	Procedures at each study visit.


| Procedure/Tests | Screening/  Enrolment | Post-operative, Intervention | | 4 weeks | 6 months | 12 months | 24 months |
| --- | --- | --- | --- | --- | --- | --- | --- |
|  |  | < 48 hrs | 5-9 days |  |  |  |  |
| Sample collection | X |  |  | X | X | X | X |
| Blood | X |  |  | X | X | X | X |
| Urine | X |  |  |  | X | X | X |
| Swabs | X |  |  |  | X | X | X |
| GUD swabs | X |  |  | X | X | X | X |
| Tests in real time |  |  |  |  |  |  |  |
| Hgb | X |  |  |  |  |  |  |
| HIV | X |  |  |  | X | X | X |
| Syphilis | X |  |  |  | X | X | X |
| Exam | X | X | X | X | X | X | X |
| VCT | X |  |  |  | X | X | X |

**6.5.	Serious Adverse Event Reporting**

The occurrence of severe adverse events (SAEs) is recorded at every post-randomization visit.

6.5.a General Definitions

An AE is defined, as “any untoward medical occurrence in a participant and that does not necessarily have a causal relationship with the treatment. An AE can be any unfavorable and unintended sign (including an abnormal laboratory finding), symptom or disease temporarily associated with the beginning of the treatment (i.e., following randomization).**”** Adverse events during screening and prior to randomization that result from study participation were also reported.

- The adverse events are defined in Table 3 and AE notification is given in form 11a.

**6.5.b Severity of AEs**

- The severity of AEs is characterized as:

. *mild* (transient and not affecting activity),

*. moderate* (requiring modification of activity or additional medical/minor surgical/intervention),

*. severe* (resulting in: death, life threatening condition, incapacitating symptoms which require hospitalization, significant medical/surgical interventions or which result in persistent significant disability/incapacity).

- All severe occurrences are considered serious adverse events (SAEs) for reporting purposes, consistent with FDA definitions as follows:

. Life threatening: substantial risk of dying at the time of the AE

. Hospitalization (initial or prolonged): if admission to hospital or prolongation of hospital stay results from the AE.

. Disability: if the AE resulted in a significant, persistent or permanent change, impairment, damage or disruption in the patient’s body function/structure, physical activities or quality of life.

. Intervention required to prevent permanent impairment or damage: if it is suspected that the treatment may result in a condition which required medical or surgical intervention to preclude permanent impairment or damage to the patient.

**6.5.c Relationship of AEs to the intervention**

The relationship of AEs to study participation, particularly surgery or medications will in most cases be clear. We use the following classification.

. *definitely related* to study procedures such as surgery or anesthesia/analgesia (occurred during surgery or postoperatively),

. *probably related* (occurred during or after study procedures such as surgery, most likely to be due to the study procedure but could arise from other causes,

. *possibly related* (occurred during participation, but could equally or more plausibly be ascribed to other causes), and

. *unrelated* (an event clearly explained by causes other than the trial participation

- The timing of AEs is divided into screening/enrolment/randomization, operative, postoperative and during longer-term follow up.

**6.5.d Study Definition of Adverse Events**

The anticipated adverse events are largely those associated with surgery or study medications. The description of anticipated potential AEs is given in Table 3. (This is in part based on the AE classification used for the Kisumu trial of male circumcision).	All medical officers were trained in the recognition and classification of the adverse events, and in the procedures required for reporting. The senior Medical Officers, will be responsible for AE reports.

Any other serious events not defined in Table 3 will be reported as SAEs.

Table 3. Study Definition of Adverse Events

| Adverse Event Type | Description of Adverse Event Type | Severity  and Codes |
| --- | --- | --- |

| A. During Surgery |  |  |
| --- | --- | --- |

| Pain | Minor, not requiring additional anaesthesia | Mild (APS1) |
| --- | --- | --- |

|  | Moderate/severe controlled with additional anaesthesia* | Moderate (APS2) |
| --- | --- | --- |

|  | Severe, not controlled by additional anaesthesia | Severe (APS3) |
| --- | --- | --- |

| Excessive bleeding | More bleeding than usual, but easily controlled | Mild (ABL1) |
| --- | --- | --- |

|  | Bleeding that requires pressure dressing to control | Moderate (ABL2) |
| --- | --- | --- |

|  | Blood transfusion or transfer to another facility for management required | Severe (ABL3) |
| --- | --- | --- |

| Anesthetic-related event | Palpitations, vaso-vagal reaction or emesis | Mild (AAN1) |
| --- | --- | --- |

|  | Reaction to anesthetic requiring medical treatment in study clinic but not transfer to another facility | Moderate (AAN2) |
| --- | --- | --- |

|  | Anaphylaxis or any reaction requiring transfer to another facility | Severe (AAN3) |
| --- | --- | --- |

| Excessive skin removed | Adds time or material needs to the procedure, but does not result in any discernable adverse condition | Mild (AES1) |
| --- | --- | --- |
|  | Skin is tight, but additional operative work not necessary | Moderate (AES2) |

|  | Requires re-operation or transfer to another facility to correct the problem | Severe (AES3) |
| --- | --- | --- |

| Damage to the penis | Mild bruising or abrasion, not requiring treatment | Mild (ADP1) |
| --- | --- | --- |

|  | Bruise or abrasion to the glans or shaft of the penis requiring pressure dressing or additional surgery to control | Moderate (ADP2) |
| --- | --- | --- |

|  | Portion or all of the glans or shaft of the penis severed or burned by electrocautery | Severe (ADP3) |
| --- | --- | --- |

| B. First Month Post-Surgery |  |  |
| --- | --- | --- |

| Pain | Symptoms of pain requiring bed rest for less than half the day | Mild (BPA1) |
| --- | --- | --- |

|  | Pain requiring bed rest for more than half day | Moderate (BPA2) |
| --- | --- | --- |

|  | Excruciating pain requiring total bed rest | Severe |
| --- | --- | --- |

| Excessive bleeding | Dressing or other materials (for example underwear) spotted but dry | Mild (BBA1) |
| --- | --- | --- |

|  | Dressing or other materials wet with clotted blood requiring change of dressing | Moderate (BBA2) |
| --- | --- | --- |

|  | Dressing or other materials soaked with blood with obvious active bleeding requiring surgical exploration or transfusion | Severe (BBA3) |
| --- | --- | --- |

| Excessive skin removed | Client concerned but no discomfort on erection | Mild (BES1) |
| --- | --- | --- |

|  | Causes slight disconfort on erection but surgical correction not necessary. | Moderate (BES2) |
| --- | --- | --- |

|  | Interferes with life and surgical correction necessary | Severe (BES3) |
| --- | --- | --- |

| Insufficient skin removed | Prepuce partially covers the glans only when extended | Mild (BIS1) |
| --- | --- | --- |

|  | Prepuce still partially covers the glans and re-operation is required to correct | Moderate (BIS2) |
| --- | --- | --- |

|  | Not applicable |  |
| --- | --- | --- |

| Swelling/Hematoma | More swelling than usual, but no treatment needed | Mild (BSH1) |
| --- | --- | --- |

|  | swelling requiring surgical exploration but no evidence of active bleeding | Moderate (BSH2) |
| --- | --- | --- |

|  | Rapidly expanding Heamatoma suggesting active bleeding requiring surgical exploration or referral | Severe (BSH3) |
| --- | --- | --- |

| Damage to the penis | Mild bruising or abrasion, not requiring treatment | Mild (BDP1) |
| --- | --- | --- |

|  | Bruise or abrasion to the glans or shaft of the penis requiring pressure dressing or additional surgery to control | Moderate (BDP2) |
| --- | --- | --- |

|  | Portion or all of the glans or shaft of the penis severed | Severe (BDP3) |
| --- | --- | --- |

| Infection | Pain and erythema with no obvious swelling | Mild (BIN1) |
| --- | --- | --- |

|  | Painful swelling with erythema or elevated temperature or purulent wound discharge | Moderate (BIN2) |
| --- | --- | --- |

|  | Cellulitis or wound necrosis | Severe (BIN3) |
| --- | --- | --- |

| Delayed wound healing | Healing takes longer than usual, but no extra treatment necessary | Mild (BDW1) |
| --- | --- | --- |

|  | Additional non-operative treatment required | Moderate (BDW2) |
| --- | --- | --- |

|  | Requires re-operation to correct | Severe (BDW3) |
| --- | --- | --- |

| Appearance | Client concerned, but no discernable deformity | Mild (BAP1) |
| --- | --- | --- |

|  | Minimal deformity does not require re-operation | Moderate (BAP2) |
| --- | --- | --- |

|  | Significant deformity requires re-operation to correct | Severe (BAP3) |
| --- | --- | --- |

| Problems with voiding | Transient complaint that resolves without treatment | Mild (BVO1) |
| --- | --- | --- |

|  | Requires a special return to the clinic, but no additional treatment required | Moderate (BVO2) |
| --- | --- | --- |

|  | Requires referral to another facility for management | Severe (BVO3) |
| --- | --- | --- |

| Wound dehiscence | Wound disruption involving no more than one principal suture. | Mild (BWD1) |
| --- | --- | --- |
|  | Wound disruption and involving two or more principal sutures. No surgical intervention required. | Modarete (BWD2) |
|  | Significant wound disruption requiring surgical correction | Severe (BWD3) |
|  |  |  |

| **C. One Month or More Post-Surgery** |  |  |
| --- | --- | --- |

| Infection** | Pain and erythema no obvious swelling | Mild (CIN1) |
| --- | --- | --- |

|  | Painful swelling/purulent wound discharge | Moderate (CIN2) |
| --- | --- | --- |

|  | Cellulitis or wound necrosis | Severe (CIN3) |
| --- | --- | --- |

| Delayed wound healing | Healing takes longer than usual, but no extra treatment necessary | Mild (CDW1) |
| --- | --- | --- |

|  | Additional non-operative treatment required | Moderate (CDW2) |
| --- | --- | --- |

|  | Requires re-operation to correct | Severe(CDW3) |
| --- | --- | --- |

| Appearance | Client concerned, but no discernable deformity | Mild (CAP1) |
| --- | --- | --- |

|  | Significant scarring or other cosmetic problem, but does not require re-operation | Moderate (CAP2) |
| --- | --- | --- |

|  | Requires re-operation to correct | Severe (CAP3) |
| --- | --- | --- |

| Excessive skin removed | Client concerned, but there is no deformity on erection | Mild (CES1) |
| --- | --- | --- |

|  | Causes slight discomfort on erection but surgical correction not necessary | Moderate (CES2) |
| --- | --- | --- |

|  | Interferes with sexual life and surgical correction is necessary | Severe (CES3) |
| --- | --- | --- |

| Insufficient skin removed | Prepuce partially covers the glans only when extended | Mild (CIS1) |
| --- | --- | --- |

|  | Prepuce still partially covers the glans and re-operation is required to correct | Moderate (CIS2) |
| --- | --- | --- |

|  | Not applicable |  |
| --- | --- | --- |

| Torsion of penis | Torsion is observable, but does not cause pain or discomfort. | Mild (CTP1) |
| --- | --- | --- |

|  | Causes mild pain or discomfort on erection, but additional operative work not necessary | Moderate (CTP2) |
| --- | --- | --- |

|  | Requires re-operation or transfer to another facility to correct the problem | Severe (CTP3) |
| --- | --- | --- |

| Erectile dysfunction | Client reports occasional inability to have an erection | Mild (CED1) |
| --- | --- | --- |

|  | Client reports frequent inability to have an erection | Moderate (CED2) |
| --- | --- | --- |

|  | Client reports complete or near complete inability to have erections | Severe (CED3) |
| --- | --- | --- |

| Psycho-behavioral problems | Client reports mild sexual dissatisfaction attributed to circumcision, but no significant psycho-behavioural consequences | Mild (CPB1) |
| --- | --- | --- |

|  | Client reports significant sexual dissatisfaction attributed to circumcision, but no significant psycho-behavioural consequences | Moderate (CPB2) |
| --- | --- | --- |

|  | Significant depression or other psychological problems attributed by the participant to the circumcision | Severe (CPB3) |
| --- | --- | --- |
| Other AES | Other AEs are described below*** |  |

### COMMENTS:

•	Additional anesthesia implies a volume of local anesthetic in excess of initial amount drawn up in the syringe and calculated to be sufficient for patient’s weight before surgery.

** Infection is indicated by swelling, erythema, pain, elevated temperature ( locally or systemically, or purulent discharge)

*** Other AEs

New AE codes

During the February 2006 review, it was decided that specificity could be improved by addition of new codes. This did not affect the diagnosis of AEs, but added more detail for future analyses. The additional codes were:

1.	A new code for joint dehiscence (BWD) and infection (BIN) AEs, indicated by BWD/BIN. Degree of severity is per individual event and the first event to occur is reported first.

2.	New codes for stich sinus: SS = Sinus at Suture site SS1 = Sinus with non-puss discharge SS2 = Sinus with purulent discharge. This would be included in infectious AEs, but adds specificity.

3.	New code for anatomical site of lesion (F = Frenulum), because many wound dehiscence, poor skin apposition and infection involved the frenulum.

4.	New codes for pain on intercourse: PI = Pain on Intercourse PI1 = No interference with sex, PI2 = Reduced sexual frequency, PI3 = Refrain from intercourse due to pain

5.	A new code was added for allergic reaction to the iodine prep, since we observed cases of blisters following surgical site preparation

PREP REACTION	APR 1 = MILD BLISTERS RESOLVED

APR 2 = MORE SEVERE BLISTERS REQUIRING TREATMENT

6.	A number of cases of pain or wound dehiscence occurred after intercourse, so we coded intercourse related AEs: I = RELATIONSHIP TO INTERCOURSE

7.	A number of cases of wound dehiscence were due to external trauma (e.g., heavy lifting, bicycle accidents) so this was coded as EX =	EXTERNAL CAUSE

8.	Code for stitch granuloma: SG=Stitch granuloma. CSG 1=Small capable of resolving,no need for surgical removal. CSG 2=Not capable of resolving, requires surgical intervention. CSG 3=Not applicable

No changes were made to the AE definitions, although with hindsight, we believe these could be improved for monitoring of control surgery related AEs.

All other SAEs resulting in hospitalization or death are reported to DAIDS and the IRBs of record in real time (i.e., within 10 days of PI notification of the event).

Genitourinary AEs other than those listed in the above tables are reported as cumulative aggregate events to the DSMB and IRBs at annual intervals. “Other AEs” that are not included in the above list and do not affect the genitourinary system, are reported to the DSMB in annual aggregate tabulations, for circumcised men during the first 90 days following surgery. These “other AEs” are also reported for controls within 90 days of enrollment. The Medical Officer will be informed of “other AEs” within 10 days of notification.

The “other AEs” reportable with in 90 days of enrollment will include:

1)	Liver or pancreatic abnormalities

2)	Neurologic conditions of the CNS (e.g., meningitis, encephalitis, convulsions and headaches, visual and auditory disturbances, strokes), and peripheral neurologic conditions (e.g., peripheral neuropathies, motor weakness etc.).

3)	Myocarditis and pericarditis

4)	Dermatologic condition affecting the genital, pubic, perianal or groin

5)	Injuries and accidents

Common illnesses unrelated to circumcision will be excluded from the “other AE” category to avoid unnecessary reporting of irrelevant events. The excluded AEs are as follows:

1. Malaria and other common parasitic infections.
2. Gastrointestinal tract (GIT) diseases, including diarrhea, gastroenteritis (bacterial, viral or parasitic), and other GIT conditions, and oropharangeal infections
3. Respiratory illnesses (URTI, ALRI, pleurisy), including TB, influenza or other respiratory infections.
4. Angina, myocardial infarction, congestive cardiac failure,
5. Dermatologic conditions (e.g., scabies, infected and uninfected rashes, pruritis, fungal infection), except those affecting the genital, pubic, perianal or groin
6. Neoplasms not affecting the genitourinary tract
7. Surgical conditions not related to the intervention (eg., hernias) and which do not require hospitalization

6.5.e Management of AEs

- Free medical care is provided for AEs directly related to participation including surgical care if needed. If referral for medical or surgical complications is required, participants are referred to the Kalisizo Hospital (2-3 minutes driving time) from the surgical facility the Rakai Hospital or hospitals in the neighboring District of Masaka (approximately 30 minutes driving time) from the surgical facility**.**

**6.5.f Reporting of AEs**

- All AEs are ascertained and documented by Project medical officers
- All deaths and grade 3 or higher SAEs are notified to the Ugandan and U.S. PIs within 48 hours of detection, by e-mail or fax.
- The U.S. PI then notifies the relevant IRBs (Johns Hopkins, Columbia University and Uganda Virus research Institute) and DAIDS Medical Officer in writing as soon as possible, and not later than 10 days after identification of the SAE. The nature, severity, timing and relationship of the AE to circumcision or study medications is specified by the medical officer. A summary of how the severity and relationship to study participation was determined is provided by the PI, and clarifications made by phone with the medical officer as needed.

**6.5.g Tabulations of AEs**

- The PI submits aggregate reports of all AEs and tabulations to the DSMB. AEs are described by type, time since surgery or medications, severity and relationship to study procedures.

Dates of AEs are also tabulated to assess temporal trends or patterns.

- AEs are tabulated by total number, and by number of AEs per individual for those participants experiencing more than one AE.

**6.5.h Source documentation**

- Operative and postoperative AEs use Rakai Health Sciences Program documentation (Forms 11 )
- Medical visits to health centers or other facilities outside of the Rakai Health Sciences Program cannot be completely documented due to poor records. We will endeavor to obtain copies of hospital medical records, after obtaining permission from the index person. Rakai Health Sciences Program reports (such as questionnaires), are also used to provide information.
- To maximize documentation, Rakai Health Sciences Program offesr free medical care or referral for injury or illnesses as a result of study participation. Phone numbers of medical officers are provided on the Postoperative patient Information Sheet (Form 46 ), we provide transport if needed and reimburse participants for transport costs. In addition, we arrange community health workers to provide a contact person in each village who can facilitate contact with the Project. Records are maintained for unscheduled visits (Form 16), and an AE report completed if indicated**.**

**7.0	STATISTICAL CONSIDERATIONS**

As noted previously this is an individually randomized, un-blinded trial of male circumcision to be conducted in two stages. The sample size estimates, by study stage are provided for each study endpoint, end points are defined, and an analysis plan is presented.

**7.1.	Stage 2 Sample Size for Study Endpoints**

**7.1.a. Primary Endpoint: Male HIV Incidence.**

###### Efficacy:

The Rakai STD Control Study^78^ found an incidence of 1.8/100 PY among the uncircumcised men and 0.9/100 PY among the circumcised men, with a Poisson adjusted RR = 0.53.^1^ We base our sample size estimates on this efficacy, and use a two-sided test with  = 0.05 and 1- = 0.80.

###### Assumptions:

We assume two years follow up, with participants enrolled between 9-26 months of the study and follow up completed by study month 50, and an annual loss to follow up of 15% (please see feasibility). We also estimate the loss of power due to possible contamination (i.e., cross-over) of 7.5-10%.

###### Allowance for contamination or reduced magnitude of anticipated efficacy:

We previously observed a RR = 0.53 for HIV incidence in circumcised relative to uncircumcised men, and have powered the study to be able to detect a lower efficacy of RR = 0.57. We have also assessed potential effects of contamination as follows: Let RR* be the observed rate ratio in the presence of cross-over (p), and let RR be the true rate ratio in the absence of contamination. Then RR* = (p + [1-p] x RR)/(p x RR + [1-p]) = RR/([p x RR] + [1-p]) (J. Hughes , pc). With p = 0.075 (i.e., 7.5% contamination), the observed RR* = 0.55. The sample size needs to be increased to account for this cross-over effect. With 2,500 men enrolled per arm we would accumulate 2,125 py in the 1^st^ follow up year and 1,806 py in the 2^nd^ follow up year, with a total over two years follow up = 3,932 py, which could detect a RR* ~ 0.57 with 80% power if the true RR = 0.53. (Please note this is 18.4% larger than the 3,209 py needed in the absence of contamination). Therefore, we will enroll 2,500 men per arm providing 3,932 py per arm.

###### Allowance for data dependent stopping:

We propose using formal interim analysis methods for monitoring the primary outcome (incidence of HIV infection) as well as the safety aspect of the study. Lan-DeMets group sequential methods with O’Brien-Fleming bounds ^117,118^ will be utilized to ensure the maintenance of an overall Type I error rate of 5%. For monitoring HIV incidence, we anticipate one interim analysis to allow DSMB to decide whether we can commence circumcisions in the control arm, and allow sufficient time for completion of control surgeries before the end of the project. Assuming an interim analysis at project month 40, we will accrue a total of 3,028 py observation per arm, which represents 77% (3028 * 2 / 3932 * 2) of the information to be accumulated during the trial. This information fraction, for example, will give sequential O’Brien-Fleming boundaries equal to 2.303 for the interim analysis and 2.017 for the final analysis. Without using these boundaries, we estimate (via simulation with 10,000 runs) that the overall Type I error rate wouldl be inflated to approximately 7.7%. Using these boundaries, we estimate that the Type I error rate is maintained at 0.05 and that the power for a RR of 0.53 is equal to 89.5%. Furthermore, with this RR, we estimate that there is approximately a 70% chance of crossing the boundary at the interim analysis. Sequential confidence intervals ^118a^ will be used when reporting results. The investigators will be blinded for the interim analysis. Monitoring of safety will be done similarly, except that it will occur on a continuous basis, and will use the methods of Lan *et al.*

###### Age criteria and selection of high risk groups:

Rakai has is a mature generalized epidemic setting with relatively high incidence throughout the population. Table 5 shows male age-specific HIV incidence. Incidence is lowest among adolescents, high and sustained among men aged 20-29, and declines modestly at older ages. Exclusion of adolescent men 15-19, would exclude a group with considerable HIV incidence and which is of great importance in future programs. Restricting enrollment to the highest risk age group (20-29 years) would marginally increase incidence relative to the total male population 15-49, but would only include 39.5% of all men. Enrollment restricted to men aged 20-29 would require adding many more communities in order to maintain power, but this complicates logistics and inflates costs. Moreover, acceptability was highest (65% in ages 20-29), so self-selective acceptability will automatically over sample these highest risk age groups. We therefore propose to enroll all consenting men aged 15-49.

**7.1.b.	Male STDs and STD Symptoms:**

###### STDs:

All participants will be tested for HIV and syphilis at enrollment, 6, 12 and 24 months.. For budgetary reasons, additional specimens will be collected for selective STD testing with other funds. (Please see laboratory methods). From prior data the expected infection rates are: syphilis prevalence ~10% and incidence 2.2/100 py. The RR for syphilis in circumcised versus uncircumcised men was 0.71. With 2,500 HIV- men we expect 2,125 and 1,806 per arm, observed at the 1^st^ and 2^nd^ follow ups, respectively (a total of 3,931 observations per arm). We have >80% power to detect a RR of 0.70 for the effects of circumcision on syphilis prevalence, and >80% to detect a RR = 0.5 in syphilis incidence.

###### STD symptoms:

The RR for GUD in HIV- circumcised versus uncircumcised men was 0.70. With 2,500 men enrolled per arm and 85% follow up, we have 85% power to detect a difference of this magnitude. The RR of balanitis in all circumcised versus uncircumcised men was 0.53 (prevalence 11.6% in circumcised and 22.8% in uncircumcised), and we have 98% power to detect an effect of this magnitude. Therefore, these sample sizes are adequate, even with cross-over effects.

Thus, the study is adequately powered to detect reductions in STDs and STD symptoms in the circumcision relative to the control arm**.**

**7.1.c.	Safety of Circumcision**

The frequency of moderate to severe complications following adult circumcision in the Rakai trial to date is approximately 3.6%. However, surgical and postoperative complications were expected to be ~2%, and were monitored continuously to insure safety. Monitoring of the rate of complications associated with circumcision is done using similar techniques described for monitoring efficacy, except that analysis will occur on a continuous basis. To facilitate the proper analysis of this continuous safety data, we propose to use the methods of Lan, Rosenberger, and Lachin in which they demonstrate how to construct a sequential boundary (Wald boundary) for a Z-statistic used to continuously monitoring data that maintains an overall Type I error rate. The methods build upon the spending-function methods of Lan & DeMets, and can also be used when switching from occasional (e.g. group sequential) monitoring to continuous monitoring.

We proposed to conduct interim analyses if required for DSMB safety assessment at 20 and 32 months. We expected to have completed 1,250 circumcisions by 20 months and 2,500 by 32 months. Using StatXact (Cytel Software Corp), we estimated the smallest number of AEs that would give an exact 1-sided 95% lower confidence bound on the estimated rates of AEs (not adjusted for interim analyses). With 1,250 men circumcised by 20 months, if the total AE rate  2.64% (33 events), the lower 95% CI bound would significantly exceed the expected rate of 2.0% (25 events), and for moderate/serious AEs, a lower 95% CI bound of an observed rate of 0.88% (11 events) would significantly exceed the expected rate of 0.5% (7 events). Similarly, at 32 months with 2,500 surgeries completed, if the total AE rate  2.48% (62 events), the lower 95% CI would exceed the expected 2.0% rate (50 events), and for moderate/serious AEs, with an observed rate of 0.72% (20 events), the lower 95% CI would exceed the expected rate of 0.5% (13 events).

**7.1.d.	Behavioral disinhibition:**

From prior data we expect ~ 25% of men to have multiple sex partners, ~ 17.0% to use condoms inconsistently, ~ 5.0% to consistently use condoms, ~ 46.0% report alcohol consumption in the previous 7 days and ~42% report alcohol consumption with sex. These behaviors have been shown to be significantly associated with HIV risk in the Rakai population. Assuming these rates pertain in the control arm, we will have 80% power to detect the following markers of behavioral disinhibition in the intervention arm: a) an increase in multiple sex partners to 30%, b) a reduction of inconsistent condom use to 13.2%, and c) a reduction in consistent condom use to 3% (i.e., an overall reduction in condom use of - 5.8%), and d) and an increase in alcohol use within the past 7 days to 50.4%, or of alcohol consumption with sex to 46.4%.

The relevance of changes in behaviors of this magnitude are discussed in 7.5.b.

**7.2.	Accrual and Feasibility:**

The average number of men aged 15-49 per community is ~ 190, of whom we expected 137 to be uncircumcised and HIV-negative (84% uncircumcised and 86% HIV-).^2^ Assuming 50% acceptance of circumcision we expected an average of 69 subjects enrolled per community. With the required sample size of 2,500 men per arm we could enroll this many participants by surveillance in ~36 communities per arm, for a total of ~72 communities. It is feasible to expand coverage to additional communities. However, we would also allow non-cohort participants to enroll as “walk in subjects”. We believed that the current cohort surveillance participants and walk in participants would readily achieve the sample size goals. (Our previous trials enrolled over 12,000 subjects).

We estimated accrual of ~ 150-160 subjects per month. The accrual in relation to timing of the three main DSMB meetings is: ~ 2870 subjects by month 9; ~ 5000 subjects by month ~ 31 (with two year follow up of the 200 Stage 1 participants completed); and 5000 subjects by month 41 (with two year follow up of ~ 2069 volunteers completed).

###### Feasibility of follow up:

In the present trial, the eligibility criteria included intent to remain in the community for at least one year. We implemented additional procedures for active tracing. In brief, we a) asked relatives of absent subjects to contact them or to provide contact addresses, b) asked absentees to return for follow up and compensate them for time and travel expenses, and c) established peripheral hubs in urban centers such as Masaka and Kampala where most absentees go for work or education, in order to facilitate follow up outside the District. With eligibility based on intent to stay and active tracing, we believed that we could achieve a minimum of 85% or more follow up. In our maternal-infant trial we achieved 94% follow up of 4,036 persons, and in our MER study we have achieved 95.4% follow up on a sample of 383 subjects. We therefore believde follow up of 85% was feasible in the proposed trial.

**7.3	Randomization and Stratification**

- Johns Hopkins University generated a list of randomized numbers in blocks of 20 to insure comparability of men randomized to intervention and control arms, within cohort communities or groups of “walk in” participants”.
- A random assignment sheet was placed in an opaque sealed envelope. The envelopes in blocks of 20, were generated by Rakai Health Sciences Program Data management using a computerized list provided by JHU, and were retained securely by the Circumcision Data Management Team.
- After enrollment and consent, men were asked to select an envelope from the block of 20 envelopes being used at that time. The envelope contained the assignment sheet showing the randomization number and random assignment to treatment or control arm. The Enrollment Team then affixed the label with the individual’s study ID number to the sheet. (Form 18).
- The assignment sheet with the study ID# labeled was entered into a data base to record study arm allocation.
- The list of random allocation numbers linked to participants were retained at JHU in locked files, and in password protected computers.
- All randomization envelopes were logged out and in daily. A data base monitored used and unused allocation numbers, and track disposition of all envelopes.
- Men were randomized at the enrollment visit. If participants changde their minds and withdraw, or at a subsequent preoperative visit, the surgeon determined that circumcision is contraindicated for medical reasons, the men wer still considered enrolled and randomized.

**7.4	Analysis Plan**

**7.4.a	Stage 2 Analysis Plan**

Primary Endpoint, HIV incidence.

HIV incidence per 100 person-years (PY) will be estimated, assuming seroconversion occurred at the mid-point of each follow up interval. In both arms, time from enrollment will be accumulated up to 24 months of follow up. Person time will discount the postoperative period of sexual abstinence for intervention arm subjects. Baseline samples of subjects who seroconvert during the first follow up interval will be tested by detuned EIA and PCR to detect prevalent infections during the window period, and any suspected prevalent cases will be excluded from incidence analyses.

Exploratory data analysis will examine the populations in the two arms at enrollment to assess baseline comparability. HIV incidence will be determined by strata of covariates such as age, marital status, behaviors (e.g., number of partners, condom use), health status and other variables to identify potential confounders. We will also assess contamination (i.e., cross-over) among men allocated to circumcision who do not receive surgery, and among control subjects who opt to have circumcisions performed from non-Project services.

An intent-to-treat approach will be taken, and all men who enroll will be included, irrespective of whether intervention arm participants ultimately receive circumcision, or whether control arm participants crossover and receive circumcision prior to 24 months follow up. The rationale is that a proportion of enrolled men who initially accept circumcision may change their mind prior to receipt of surgery, and such men are likely to be self-selected. Because circumcision is delayed in the control arm, such self-selection could be differential between randomization groups. Therefore, in the circumcision arm, enrolled men who subsequently refuse circumcision, will be ascribed half the person time from enrollment to scheduled date of circumcision.

We will assess HIV incidence in enrolled HIV-negative men. The primary analytical model that will be used will be Poisson regression to compare the HIV incidence rates between the two study arms.^113^ The relative incidence of HIV in the circumcision arm versus the control arm, will be estimated using Poisson regression models with adjustment for individual-level variables found to differ between groups at enrollment, and for potential confounding variables. Potential confounders determined from prior risk analyses will include age, marital status, risk behaviors (i.e, number and nature of partners, condom use and alcohol use), or imbalance of covariates between study arms will be used for adjustment in the primary models. Secondary analyses will include any additional potential confounders identified during exploratory data analysis and will include covariates associated with HIV incidence (p < 0.15), or covariates with an incidence RR > 2.0. Poisson multivariate models will be fit for the whole population and for strata of particular interest (e.g., by age, sexual risk behaviors etc.). Although we are doing an individually randomized trial, the structure and logistics of field work requires that men be randomized within communities. Thus, we will also fit Poisson models with random effect terms for each community, to insure adjustment for clustering in HIV incidence.

We will also assess the associations between selected STDs and STD symptoms and incident HIV infection to determine whether effects of circumcision on HIV incidence may be mediated by STD cofactors. If, as expected from prior analyses, selected STDs and STD symptoms such as GUD are associated with incident HIV, we will assess the circumcision effects on HIV incidence using stratified **models or via interaction terms.**

###### Secondary Endpoints:

Male STD Prevalence and GUD.

Prevalent syphilis and STD symptoms (GUD, discharge/dysuria) at baseline will be compared between study arms. At 6, 12 and 24 months follow up visits we will determine the period prevalence of STD symptoms and cumulative prevalence of syphilis (the latter based on prevalence because of persistence in seropositivity). In persons free of initial serologic infection at baseline, incident syphilis will be derived, and covariate adjusted rate ratios of incident STD symptom endpoints will be estimated by Poisson regression modeling as described above.

Behavioral change (disinhibition).

To assess possible behavioral disinhibition we will determine the frequency and changes in reported frequency of risk behaviors (e.g., number, type of partners, condom use, alcohol use) by study Arm, at enrollment and at each follow up. Data will be displayed graphically for each follow up visit. We will estimate the adjusted odds ratios of risk behaviors in the intervention versus the control arms using multivariate logistic regression for each survey visit. We will assess intra-individual changes in risk behaviors as follows. For continuous variables (e.g., number of partners), the intra-individual change in risk behaviors from enrollment will be determined, and the mean within-individual behavioral change between study arms will be assessed using repeated measures ANOVA or paired t tests. For dichotomous variables, we will assess the change in risk behavior over time, and estimate adjusted odds ratios of risk behavioral change between study arms by logistic multivariate regression. For categorical behavioral outcomes (e.g., non use of condoms, inconsistent and consistent use) we will use multivariate polychotomous logistic regression. In addition, we will have data from ongoing cohort surveillance to measure behavioral trends among non-participants, and we will determine behavioral change in the non-trial population in order to assess general trends, and to compare such non-trial associated behaviors with those reported by participants in both arms. A composite risk score will be developed on the basis of these analyses.

The relevance of changes in risk behaviors with respect to HIV incidence will be determined from prior Rakai studies of risk factors for HIV acquisition and from epidemiologic data generated by the circumcision trial. The behaviors specified above have been shown to be associated with a risk (rate ratio) ~ 2.0 or more in our observational analyses. We will estimate the effect on HIV incidence by study arm, of observed behavioral disinhibition (should it occur), using stratified analyses and multivariate analyses and stochastic modeling.

**7.5	Interim Data and Safety Monitoring**

The DSMB halted the trial for efficacy on December 12, 2006 after an unscheduled interim analysis with 73% of total information demonstrated efficacy.

***Safety.*** The expected rate of total AEs following circumcision is expected to be ~ 2%. This will continuously monitored during the trial and specifically assessed by the DSMB after completion of Stage 1 (~ 4-7 months), and at 20 and 31 and 40 months. The DSMB will be blinded to any tabulations by study arm.

***Acceptability.*** Acceptability of circumcision should be  50% among eligible men. If acceptance is less, the DSMB will evaluate the data from the pilot study to determine whether **to** proceed with the full-scale randomized clinical trial, to change accrual methods or to propose an alternative design.

**Stopping rules:** The following stopping rules are suggested**:**

***Efficacy.*** We propose an analysis at 40 months. If HIV incidence in one arm significantly exceeds that of the other arm, it is proposed that the trial be discontinued following DSMB review and discussion with Program. We suggest that formal statistical monitoring methods such as the Lan-DeMets group sequential approach ^118^ with an O’Brien-Fleming type spending function. These methods minimize the chance of inappropriately terminating the trial in its early stages. If interim analyses suggest equivalence of HIV incidence in the two arms, conditional power methods will be used to estimate the chance that further data accumulation will result in significant differences. If the probability is low, consideration will be given to stopping the trial.

***Behavioral changes, STDs, and disinhibition.*** It is possible that, despite intensive health education, circumcision may lead to false expectations of protection, engendering increased risk behaviors. If there is evidence of increased risk behaviors we would intensify health education efforts. However, if such improved education is unsuccessful, we suggest establishing stopping rules using the following possible measures of disinhibition in the intervention versus the control arm: a) a significant decline in the frequency or consistency of condom use, b) a significant increase in the number of extramarital partners, c) a significant increase in use of alcohol with sex, d) a significant increase in HIV incidence or selected STDs which are markers for risk behaviors (e.g., incident syphilis).

**8.	HUMAN SUBJECTS**

We will first describe services provided by the Rakai Health Sciences Program and relevant actions as background to consideration of human subject issues.

**8.1.	Services of relevance to Human Subject Considerations**

**8.1.a. Community Advisory Board (CAB)**

The CAB reviewed the proposed trial and their letter of approval and list of members is attached. The CAB recommended that efforts be made to promote condom use among circumcised men, that well fitting garments and transport be provided to men immediately after circumcision if required, and that compensation be offered ($15.00) for lost time following surgery. These recommendations are incorporated into the proposal. The CAB recommended that men over age 49 be provided with circumcision if they request the procedure, and subject to DSMB approval, we will make this service available after completion of the trial. The CAB did not express reservations about the delayed circumcision provided in the control arm, nor about randomization. With assistance of the CAB and our community mobilization team we established liaison with Christians and animist religious leaders, to address any concerns about circumcision.

**8.1.b. Condom Promotion:**

Condoms were provided free to all enrolled men who were advised to practice consistent use, and to obtain re-supplies from community depots maintained by the Program. Circumcised men will were advised to only resume intercourse after certified wound healing and **to practice consistent** condom use. Current condom use has increased from 9.9% in 1995 to > 30% in 2001. Use with extramarital partners was 30.8% in 1995 and is currently over 60%. Thus, the Rakai Health Sciences Program has markedly increased condom use by the community-based education and distribution program. However, despite repeated intensive Rakai Health Sciences Program condom promotion via community meetings, counseling, and community distribution of supplies, consistent condom use remains low (~ 8 % in 2001), hence HIV incidence is stable at around 1.4/100py, and there is a need for innovative prevention interventions.

**8.1.c. Voluntary HIV Counseling and Testing (VCT):**

The Project strongly promotes and provides free VCT on request to individuals or couples, and VCT is encouraged at community meetings and community counseling sessions. Ready access to VCT is provided by trained full-time counselors resident in the communities and 2 counseling supervisors. VCT is provided in the home or at the counselors office, at the client’s discretion. Pre- and post-test counseling and long-term counseling support are available. The counseling messages include information on HIV/STD transmission and prevention including fidelity, abstinence, condom use and other safe sex behaviors, partner notification and couples counseling, and general health. Post-test counseling includes interpretation of HIV results, prevention of transmission/acquisition and support. We adhere to Ugandan Government policies on VCT^83b^ which explicitly require that VCT be voluntary and preclude disclosure of results to third parties, including sexual partners, without the written permission of the index individual.

**8.1.d. General medical Care and Health Education.**

All trial participants have access to free general health care provided by Rakai Health Sciences Program at time of scheduled contact. Between surveys, care is available via Government health services. The Project provides free health education, condom promotion and free condoms to all residents (participants and non-participants**).**

**8.2.	Institutional Review Board (IRB) Reviews.**

The study was reviewed and approved by the Scientific and Ethical Committee (SEC) of the Ugandan Virus Research Institute (FWA 00001254, expiry 8/30/04), Johns Hopkins University Committee for Human Research (CHR# H.32.02.05.10.B), and Columbia University Institutional Review Board (IRB# 14741).

We are collaborating with Dr. Bernard Lo, Bioethicist, who advises us regarding resolution of ethical questions throughout the trial. We get annual continuing review approvals from SEC and WIRB.

8.3.	Training in Research Ethics:

All Rakai Health Sciences Program senior investigators in the US and Uganda, have taken and passed courses in Research Ethics/Good Clinical Practice. All other Ugandan project personnel having contact with participants, participant data or laboratory samples, have been trained in Uganda, based on materials from Johns Hopkins and Columbia.

8.4.	Proposed involvement of Human Subjects:

The trial participants are 5,000 uncircumcised, HIV-negative adolescent and adult men aged 15-49, resident in Rakai communities in southwestern Uganda. We excluded uncircumcised boys under age 15, because the low HIV incidence in such young males would not add to the study power, and we wished to establish safety and efficacy at older ages, before assessing circumcision in boys under age 15.

Men were consented for screening and if found to be eligible, they were consented for enrollment. Consenting HIV-negative men were randomly assigned to an immediate circumcision arm (intervention arm) in which they were offered the service within approximately 1 month of enrolment, or to a delayed circumcision (control arm), in which they were offered circumcision approximately 24 months after enrolment. Circumcision is carried out by trained and supervised physicians. Participants are monitored after surgery to maximize care. Men in both arms were interviewed and asked to provide biological samples at enrolment and at 6,12 and 24 month visits

Interview information was obtained at baseline, time of surgery and at each follow up visit and included sociodemographic characteristics, risk behaviors (e.g., number and type of partners, condom use, sexual networks, alcohol use, coercive sex), health information including symptoms of STDs or penile pathology (e.g., discharge, dysuria, GUD, balanitis, post-operative infection or bleeding), and symptoms suggestive of OIs or AIDS (e.g., weight loss, chronic diarrhea, cough, KS etc). Most questions had already been tested in prior Rakai Health Sciences Program surveys.

Blood samples are collected from men for detection of HIV, STDs and hemoglobin as per protocol. Male urine samples are collected for detection of STDs with other funds. Prior to surgery, subpreputial swabs are obtained for assessment of STDs as per protocol and men reporting GUD are asked for an ulcer swab. The foreskin of circumcised men is retained for histopathology.

Subjects are identified by a permanent study ID number on all forms and samples, and a polaroid photograph is used to insure identity during follow up.

8.5	Recruitment and Consent

Participants were mobilised through community mobilization meetings and home visits. With the assistance of our Community Advisory Board (CAB), we informed the population of the research. Following contact with village leaders, we conducted open community meetings during which the community was informed of the nature of the trial, including randomization into one of two arms, and the possible risks and benefits of male circumcision. Following the community informational meetings, all age eligible men aged 15-49 residing in the community (identified from Rakai Health Sciences Program data files) or non-cohort participants (“walk ins”) were invited to go to the Kalisizo Clinic or central locations in the communities (“hubs”) for screening and enrollment. They were clearly informed that participation is entirely voluntary and that they would not forego any Rakai Health Sciences Program services (condoms, VCT, access to Rakai Health Sciences Program clinics) should they decline enrollment into the trial. The Screening and Enrollment Consents are given in Appendix 1, Forms I and I.a.

All prospective volunteers were offered VCT. After completing a screening consent and the screening procedures, HIV-negative men who indicated that they were willing to accept circumcision and randomization into either the intervention (immediate circumcision) or control (delayed circumcision) arms, and who agreed to VCT were asked to provide written consent for enrollment into the NIH trial. The consent process included oral group or individual presentations with a video (where possible) and an opportunity to ask questions during preliminary health education. Participants were given an opportunity to read the consent if they are literate, or to have the consents read to them if illiterate Then individual consent was then obtained. The consent included information regarding the design of the trial, requirements for participation including follow-up and data/sample collection, the risks and potential benefits of circumcision, and the freedom to refuse in part or in whole, or to withdraw from the trial, without loss of benefits and privileges. Contact names of physicians and phone numbers are provided for questions or complaints. The copy of the enrollment consent retained by the participants did not indicate HIV status or sponsor, except by a code (NIH form I.a., Gates Foundation form I..b), in order to avoid potential disinhibition among participants in the NIH trial, and to prevent breach of confidentiality and stigmatization of men enrolled in the Gates Foundation sponsored trial**.** After randomization, men completed a consent comprehension test to ensure that they understood the trial and to correct any misunderstandings

Men who were unsure about participation, but who did not refuse outright, were told that they should take time to consider their decision. They were re-contacted after 2-3 days to determine whether they decide or decline to participate.

As is standard operating procedure, consent forms were translated into Luganda, and back translated into English. A certificate of translation was obtained. Subjects were asked to sign or thumb print the consent document, and consent was independently witnessed. Enrolment of unemancipated minors aged 15-17 required both the assent of the parent/guardian and independent individual consent by the minor. A copy of the signed form was retained by the subjects, and original forms were maintained in safe, locked stores

Consent was obtained on screening, enrollment and prior to surgery.

8.6.	Ethnic Groups and Minors

Residents of Rakai District are all Black African, and belong to several tribal groups, of which the Baganda, Banyarwanda, Bakiga and Banyankole are the largest. Men were enrolled regardless of tribal affiliation.

As indicated above, the study included respondents aged 15-17. This is in keeping with recent NIH standards that minors be included in research related to conditions which affect their health. In Rakai, HIV, STDs and genital tract conditions represent health problems at young ages. Safety and healing following circumcision needed to be examined in adolescent males, since in countries such as Kenya, there was growing evidence that males in this age group were seeking out the procedure.

Emancipated adolescents, defined as persons under the age of 18 who are heads of households, or who live independently of their parents or guardians and are not in school, were asked to provide individual informed consent. Unemancipated minors aged 15-17 were likewise asked to provide individual informed assent, but their enrolment was also contingent on written consents by the parent or guardian. (Please note: if the parent or guardian consented but the minor did not assent, that minor was not enrolled.**)**

8.7	Potential Risks to Participants

**8.7.a Risks of circumcision:**

The rate of complications following circumcision reported in the clinical literature is less than 2% and include transient hyperesthesia, hematoma, bleeding and infection. Damage to the glans or shaft is extremely rare. Physical activity or intercourse early after surgery could exacerbate bleeding, bruising or infection. Intercourse before wound healing could increase the risk of HIV acquisition. Allergy, including anaphylaxis can occur with local anesthesia and antiseptics. Drowsiness or unsteadiness can occur with pain medications.

**8.7.b Confidentiality and social harm.**

There is a risk of breech of confidentiality if patient records, interviews or lab results are revealed to third parties. There is a risk that misidentification could cause an individual to be given an incorrect diagnosis or inappropriate treatment, or be inadvertently denied indicated treatment. There is a risk that knowledge of positive HIV results received via voluntary counseling and testing (VCT) could lead to distress, marital disruption or partner abuse.

**8.7.c Disinhibition:**

If circumcised men believe themselves to be at reduced risk of HIV or STD infections, they may adopt higher risk behaviors. If the copy of the consent form retained by HIV-negative men enrolled in the NIH trial documented HIV status, this “proof” of negative status could be used to negotiate unsafe sexual behaviors. To avoid this dilemma, the copy of the consent provided to participants will not indicate HIV status directly, or indirectly (e.g., by specifying NIH or Gates trial enrollment).

**8.7.d Risk related to biological specimen collection.**

Blood and urine collection is minimally invasive and entails minimal risk. Foreskins are saved for separate studies: the risk of collection is summarized under risks of circumcision.

8.8	Procedures for Protecting Against Risk.

**8.8.a Risks of circumcision:**

The risk of complications during or following circumcision is minimized by careful training and certification of physicians, supervision by an experienced urologist and referral/exclusion of men with anatomic abnormalities of the genitalia. A physician at the Kalisizo Hospital is available to assist with surgical complications. The hospital is only minutes away from the Rakai clinic facility. The risk of infection is minimized by: i) In men with evidence of current infection, surgery is deferred, and men are treated with antibiotics until infection has resolved. ii) Surgery is performed in a dedicated surgical facility, with strict adherence to aseptic procedures, autoclaving of all instruments, and careful dressing and monitoring of wound healing. iii) Any postoperative infection is treated with antibiotics and, if indicated, men are hospitalized at project expense for wound care. The risks of bleeding or bruising are minimized by: i) careful surgical technique and attention to bleeders, ii) use of an occlusive dressing, iii) postoperative bed rest and provision of well fitting garments and transport home, iii) encouraging participants to refrain from physical work and activity for approximately 3-5 days, and to refrain from intercourse until complete wound healing is certified. Men are provided with clear information on risks of premature resumption of physical work, and compensation is paid for work time lost (the equivalent of $15, to enable them to take the day of surgery, and the subsequent 3-5 days, off work). Potential risk of HIV is minimized by advising men to refrain from intercourse until the wound is healed, and promotion of condoms when intercourse is resumed. We provide detailed information and follow up for subjects to minimize risks. Postoperative pain is controlled by analgesia and limitation of activity. Men experiencing hyperesthesia are told this is usually transient, but if it does not resolve within a week, they are referred to the urologist for free care.

**8.8.b Confidentiality and social harm**

The Rakai Health Sciences Program has, for fourteen years, maintained full confidentiality of participant records. Informed consent documents are retained in locked filing cabinets and store rooms, accessible only to senior investigators or designated staff. Case report forms retain participant’s names until completion of analysis. All questionnaires are stored in secure, locked facilities. Only designated staff have access to these records. Photos used to identify persons are kept in locked cabinets and are under the control of the study supervisor. All computerized data bases are password protected. Electronic records containing confidential or sensitive information only contain the study ID # without the participant’s name. Files of lab results are maintained in a separate safe computer file, with study ID numbers, and contain no personal identifiers.

Misidentification is avoided by the photo ID and by use of unique, alphanumeric check digit ID numbers. The check digit number avoids data entry errors. All documents and samples are labeled with pre-printed study numbers to avoid transcription errors. Lab results are double entered to avoid data keying errors.

**8.8.c HIV Voluntary Counseling and Testing (VCT): protection of confidentiality**

Individuals and couples in the Rakai cohort are offered VCT, and those who accept are flagged for expeditious assay. HIV results are provided on the basis of two concordant EIAs (i.e., concordantly negative or positive). If EIAs are discordant, results are not provided until WB confirmation is available. The following procedures are followed to minimize risk of social or psychological harm that might result from breech of confidentiality. HIV result letter used by the counselor to inform the participant, contains the study ID# and the participant’s name to insure correct identification, but the HIV result is coded so as to be un-interpretable by a third party.

Participation in VCT (both individual and couples counseling), although strongly encouraged, is entirely voluntary. Counselors provide pre-results counseling for all participants and inform them of the potential for social harm, and advise them on how to mitigate such consequences. Participants may opt to learn their results and receive post-test counseling, or they may decline to learn their results. Only men who agree to learn their HIV results are enrolled in the NIH sponsored trial. Results are provided in private to individuals or couples (if both individuals have elected to participate in couples counseling). If recipients opt for individual counseling, they are encouraged to inform their partners of the test results but, in accord with Ugandan Government policy, no results are revealed to third parties, including spouses, without an individual’s express, written permission.

**8.8.d Disinhibition:**

To avoid false expectations of protection by circumcision we carefully informed participants that the efficacy of the interventions was unknown at that time, that monogamy with an HIV-negative partner or consistent use of condoms were the only proven methods of preventing infection, and that avoidance of high risk behaviors is imperative to reduce HIV risk. (Condoms are provided free of charge by the Project and we recommend that condoms be used by all men). Because we have designed the study within Rakai communities, all members of the community - men and women - are informed via town meetings of the need to continue other safe sex practices. The message is reiterated during each community town meeting for the duration of the study.

To minimize disinhibition among men in the NIH trial, and to prevent the use of copies of the consent form as “proof” of HIV-negative status, the consent copies do not indicate HIV status. Disinhibition is also minimized by intensive health education and monitoring of risk behaviors both via surveys and by qualitative research methods.

We monitored behaviors carefully during the trial and if an excess of risk behaviors was observed, the DSMB would determine whether this can be rectified, or whether the trial should be terminated.

8.8.e Risks of biological sample collection.

Venipunctures and genital ulcer swabs are collected by highly trained and experienced Rakai Health Sciences Program survey and clinical personnel. Over 80,000 serological specimens and 80,000 urine specimens were collected during the Rakai STD Control Trial for HIV Prevention, with no significant adverse event.

To avoid potential risks of incorrect HIV diagnoses, results are only provided on the basis of concordant EIAs with WB confirmation of discordant EIAs.

8.9	Study Benefits to Participants

**8.9.a Health Care:**

Residents of Rakai Health Sciences Program study communities, whether or not they have agreed to participate in any Rakai study, are provided with health education via town meetings and are offered condoms.

All residents also have access to Rakai Health Sciences Program mobile clinics for free general health care and symptom-based STD treatment at the time of our annual survey rounds.

All residents have access to the Rakai Health Sciences Program fixed clinic in Kalisizo for STD treatment; study participants are offered STD treatment for free in the fixed clinic.

All HIV Positive residents, who are study participants, are offered free health services and treatment for OIs through the Rakai Health Sciences Program fortnight HIV clinic in our fixed facility in Kalisizo, and in one mobile clinic which services distant communities.

In addition to the benefits above, study participants are offered free, confidential individual and couples voluntary counseling and testing.

**8.9.b STD testing and treatment provided to participants**

In addition to the symptom-based STD treatment offered to all study community residents through our mobile clinics at the time of each study round, circumcision trial participants in both arms are routinely tested for syphilis, and offered treatment on the basis of results. In addition, participants are examined and any pathology diagnosed clinically is treated. We use highly effective, single dose observed therapy, as we did in the STD control trial. Drugs include azithromycin, ciprofloxacin, cefixime, metronidazole and IM benzathine penicillin. Participants in both study arms are also provided with HSV-2 suppressive therapy, if there is evidence of active herpetic ulceration at the time of the survey or prior to circumcision.

**8.9.c General health care:**

At the time of each survey round, Rakai Health Sciences Program mobile clinics offer free general health care, including antimalarials and antibiotics as required, to all study community residents. Symptom-based care includes treatment for respiratory conditions, thrush, fever, skin conditions, pain and diarrhea.

**8.9.d Treatment for Opportunistic Infections and ARVs**

As indicated above, all HIV-positive residents of Rakai study villages are offered free care at fortnightly clinics held in our Kalisizo facility and in one mobile clinic, which services distant villages. We provide prophylaxis and treatment of OIs, and ARVs via the Presidential AIDS Initiative (PEPFAR). Care is provided when appropriate and feasible.

**8.9.e Provision of free circumcision:**

Men in the intervention arm were offered free circumcision within one month of enrolment.

Men in the control arm are provided with free circumcision after 24 months follow up as a service, contingent on DSMB assessment of safety. They were told that information on efficacy was not available then, and that they could defer circumcision to the end of the study when the last men enrolled into the two study arms complete 2 years of follow up. In December of 2006, the NIAID stopped the trial after interim analysis showed more that 50% efficacy. The NIAID now requires that all uncircumcised men in the control arm of the trial be offered surgery on an expedited schedule, without requiring completion of 2 years of follow-up. .)

**8.9.f Condoms**

Condoms are provided free of charge to study participants via the survey teams, health education team, condom sale agents. HIV counselors and our community mobilizers. The former reside permanently in the study communities.

8.10.	Compensation for Participants

We proposed to provide $30.00 (approximately 52,500/= Uganda shilling) as compensation for time lost due to surgery and the time required for screening/ enrollment and follow-up. All men received $3.00 compensation at time of enrollment, and for each scheduled visit at 3-6 weeks, 6, 12 and 24 months. Men in the immediate circumcision arm received $15.0 compensation distributed as $5.00 on day of surgery, and $5.00 at each of the 48 hour and 7 ±2 days visits. Control arm men will receive $15.00 compensation for time lost due to surgery. Based on discussions with our Community Advisory Board, the proposed amount was deemed to fairly recompense participants, without being a coercive inducement. Experience during stage one suggested that this distribution of compensation can maintain high compliance rates and avoid disinhibition that might occur with larger lump sum payments.

Men will be invited to attend focal points or “hubs” located in the communities and will be compensated for transport cost.

8.11	Benefit-Risk Ratio

The risks of the proposed Circumcision Trial are reasonable in relation to the benefits. Adult circumcision entails relatively minor surgery under local anaesthesia, with low rates of complications. The training and supervision of physicians performing the procedure and the conditions under which it is performed should minimize these risks. Stage 1 results showed that circumcision offered in the Rakai study clinic is safe . The individual benefits of the intervention are potential protection from HIV and STDs, and improved penile hygiene. There are substantial societal benefits. It is now known that circumcision is efficacious. This has provided critical information for policy and development of programs for HIV prevention. Because circumcision is a one time procedure, such benefits could be life long**.**

The risks of breech of confidentiality are minor, and the Rakai Health Sciences Program has put in place numerous safeguards to prevent such breeches and to avoid misidentification errors. The benefits also include access to HIV results and counseling through VCT, free condoms and health care.

The risk of disinhibition could not be determined prior to study initiation, but would be offset by careful education and patient instruction. We have neither observed disinhibition in our previous intervention trials nor in this trial.

**9.0 PUBLICATION OF RESEARCH FINDINGS**

Results of the research are communicated to the Ugandan Ministry of Health and to study communities.

A Publication Committee consisting of senior investigators will be formed to decide on topics for papers, and allocation of responsibility for manuscripts. First authorship will be given to the investigator who takes primary responsibility for specific analyses and writing of papers. Papers or abstracts will be submitted only after review by the Publication Committee.

**10.	SUMMARY OF CASE REPORT FORMS, SUMMARY OF INFORMED CONSENT DOCUMENTS, TABLES & FIGURE – RANDOMIZED CLINICAL TRIAL PROTOCOL**

SUMMARY OF CASE REPORT FORMS - FOR THE RCT (FORMS ARE NOT ATTACHED BUT ARE ON FILE)

00.Registration form.

01.Screening Interview and Exam.

02.a. Pre-baseline Interview

02.b. Enrolment Interview

03.Pre- operative Interview and Exam

04.Surgical and postoperative record

05.First day POP follow up Interview and Exam

07.First week POP follow up Interview and Exam

08.Second week POP follow up Interview and Exam

09.a. Third week POP follow up Interview and Exam

09.b. Fourth week POP follow up Interview and Exam-Immediate

09.c. Fourth week follow up Interview and Exam -Delayed

09.d. Fifth week POP follow up Interview and Exam

09.e. Sixth week POP follow up Interview and Exam.

10.Syphilis result form

11a. Adverse Event report form

11b. Adverse Event clinical monitoring form

12a. Sixth month follow up questionnaire

12b.12 Monthly Interview and Exam

12c. 24 Monthly Interview and Exam

13.Protocol deviation form

14.Withdrawal form

15. Missed visit/ Refusal.

16.Unscheduled visit form

17.Visit Track card

18.Randomization sheet

19.a. Screening verification flow sheet.

19.b. Enrolment verification flow sheet

19.c. Surgical verification flow sheet.

19.d. FUP verification flow sheet

20.Emergency Equipment inventory checklist.

21.a. NIH rejection form

22.Eligibility Verification form

23.Sterilizer QA Record

24.Circumcision instrument set

25.Communication Flag

27.Daily productivity log sheet

28.Linkage form.

29.VCT Result communication form

30.VCT Counselor’s checklist

31. Pre-test counseling communication form

32.VCT Result letter.

33.Mobilization/Health education guide.

34.Sterilizer Record log

35.MOH-STD FORM 4 (Partner notification)

36.Appointment card

37.Error detection sheet

38.Re-interview/Tape recording sheet

39.Certification assessment form

40.Verbal autopsy.

41.Verify biological monitoring card

41.a. Verify biological monitoring control card

42.Invitation to enroll in RCCS

43.Comprehension test interview

44.Spouse referral form

45.RCCS-Circumcision referral form.

46. Postoperative Patient information sheet

48.RCCS FUP Interview

Lab Form # 001	Specimen collection form.

Lab Form # 002	HemoCue Collection and Result Form

Lab Form # 003	Rapid HIV testing worksheet.

Lab Form # 004(a) Blood receipt form

Lab Form # 004(b) Urine receipt form.

Lab Form # 004© PCR swabs log form

Lab Form # 004(d) HPV shipper log form.

Lab Form # 005(a) RPR card test.

Lab Form # 005(b) TPPA confirmatory test.

Lab Form # 006	Wound infection culture and Gram stain results form

Lab Form # 007	Foreskin log form

Lab Form # 015	Participant result form.

#### SUMMARY OF INFORMED CONSENT DOCUMENTS - FOR THE RCT (DOCUMENTS ARE NOT ATTACHED BUT ARE ON FILE)

I. Screening consent

I.a. Enrollment consent NIH

II.a. Post randomization Information sheet- Immediate

II.b. Post randomization Information sheet- Delayed

III	Surgical consent Form

IV	Control subjects consent for surgery

V	RCCS Baseline Consent

VI	RCCS Follow-Up Consent

OTHER DOCUMENTS (DOCUMENTS ARE NOT ATTACHED BUT ARE ON FILE)

49.	Theater Logs and Procedures

50.	Literature Cited

51.	Study Site and Experience of Investigators

52.	Roster of Protocol Team Members

**Table 1.** Time Line – NIH-funded RCT

| **Activities (Project months)** | **Year 1** | **Year 2** | **Year 3** | **Year 4** | **Year 5** |
| --- | --- | --- | --- | --- | --- |
| **Stage 1**  **(~ mths 1-6)** | **xxxxxx** |  |  |  |  |
| **Assessment of acceptability/safety**  **(~ mth 6-7)** | **x** |  |  |  |  |
| **Finalize mobilization message etc.** | **x** |  |  |  |  |
| **Stage 2** |  |  |  |  |  |
| **Enrollment/Randomization**  **(~ mths 9-26)** | **xxxx** | **xxxxxxxxxxxx** | **xx** |  |  |
| **Intervention Circumcisions**  **(~ mths 9-27)** | **xxxx** | **xxxxxxxxxxxx** | **xxx** |  |  |
| **6 month follow up**  **(~ mths 14-32)** |  | **xxxxxxxxxx** | **xxxxxx** |  |  |
| **12 month follow up**  **(~ mths 20-38)** |  | **xxxx** | **xxxxxxxxxxxx** | **xx** |  |
| **24 month follow up**  **(~ mths 32-50)** |  |  | **xxxx** | **xxxxxxxxxxxx** | **xx** |
| **DSMB safety monitoring**  **(~ mths 19,31)** |  | **x** | **x** |  |  |
| **DSMB Interim Analyses**  **(~ mths 40, 56)** |  |  |  | **X** | **X** |
| **Circumcise Controls**  **(~ mths 24-56)** |  |  | **xxxxxxxxx** | **xxxxxxxxxxxx** | **xxxxxxxxxxxx** |
| **Analysis and reports**  **(~ mths 51-60)** |  |  |  |  | **xxxxxxxxxxxx** |

**Table 2.** Summary of Forms for Stage 2 of the Trial – NIH-funded RCT

| **Record Forms** | **Contacts** | | | | | | | |
| --- | --- | --- | --- | --- | --- | --- | --- | --- |
|  | **1** | **2** | **3** | **4** | **5** | **6** | **7** |  |
| **Pre-Enrollment interview for cohort participants interviewed within past 6 months ( 02a/48)** | **x** |  |  |  |  |  |  |  |
| **Screening Consent (I)** |  | **x** |  |  |  |  |  |  |
| **Enrolment Interview (02b)** |  | **x** |  |  |  |  |  |  |
| **Eligibility verification form ( 22)** |  | **x** |  |  |  |  |  |  |
| **Enrolment Consent (I.a.)** |  | **x** |  |  |  |  |  |  |
| **Interview for non-cohort participants^1^ and for cohort participants interviewed > 6 months previously ( 02a)** |  | **x** |  |  |  |  |  |  |
| **Randomization form ( 18)** |  | **x** |  |  |  |  |  |  |
| **Post-randomization Information sheet (II)** |  | **x** |  |  |  |  |  |  |
| **Surgical consent (III)** |  |  | **x** |  |  |  |  |  |
| **Pre-operative Interview/ Exam (03)** |  |  | **x** |  |  |  |  |  |
| **Surgical record (04)** |  |  | **x** |  |  |  |  |  |
| **Postoperative patient information sheet ( 46)** |  |  | **x** |  |  |  |  |  |
| **Post-operative visit forms** |  |  |  | **x...** |  |  |  |  |
| **Follow up Visits 6, 12, 24 months (12)** |  |  |  |  | **x** | **x** | **x** |  |
| **Adverse Events Reporting Form (11)** |  |  | **x** | **x** | **x** | **x** | **x** |  |
| **Protocol deviation form (13)** |  | **x** | **x** | **x** | **x** | **x** | **x** | **x** |
| **Withdrawal form (14)** |  | **x** | **x** | **x** | **x** | **x** | **x** | **x** |
| **Missed visit form (15)** |  |  | **x** | **x** | **x** | **x** | **x** | **x** |
| **Control subjects consent for surgery (IV)** |  |  |  |  |  |  |  | **x** |

**^1^Men who were not previously enrolled in the cohort or cohort participants last interviewed > 6 months before enrollment will complete the interview after enrollment into the trial**

**Table 3.** Number of AEs, exact confidence intervals and 1-sided p values for all AEs and for moderate/serious AEs.

| **Number of events /**  **Estimated rate (%)** | **Exact 95% Confidence intervals** | **Exact 1-sided p-value for testing vs. 2% rate** | **Exact 1-sided p-value for testing vs. 0.5% rate** |
| --- | --- | --- | --- |
| **0** | **0.00-3.52** | **.1326** | **.6058** |
| **1** | **0.05-4.92** | **.4033** | **.3942** |
| **2** | **0.36-6.60** | **.5967** | **.0898** |
| **3** | **0.82-7.99** | **.3233** | **.0141** |
| **4** | **1.38-9.43** | **.1410** | **.0017** |
| **5** | **1.99-11.03** | **.0508** | **.0002** |
| **6** | **2.65-12.16** | **.0155** | **<.0001** |
| **7** | **3.33-13.49** | **.0041** | **<.0001** |
| **8** | **3.52-14.84** | **.0009** | **<.0001** |

**Table 4.** Male HIV Incidence by Age.

| **Age groups** | **15-19** | **20-29** | **30-39** | **40-49** | **All** |
| --- | --- | --- | --- | --- | --- |
| **HIV Incidence/100 py in uncircumcised men (observed)** | **1.1** | **2.1** | **1.9** | **1.8** | **1.8** |
| **Estimated number of men enrolled per arm** | **435** | **988** | **582** | **495** | **2,500** |
